# Imbalance between the expression dosages of X-chromosome and autosomal genes in mammalian oocytes

**DOI:** 10.1038/srep14101

**Published:** 2015-09-15

**Authors:** Atsushi Fukuda, Motohiko Tanino, Ryo Matoba, Akihiro Umezawa, Hidenori Akutsu

**Affiliations:** 1 Center for Regenerative Medicine, National Research Institute for Child Health and Development, 2-10-1 Okura, Setagaya, Tokyo 157-8535, Japan; 2 DNA Chip Research Inc. 1-1-43 Suehiro-cho, Tsurumi-ku, Yokohama 230-0045, Japan; 3 Department of Stem Cell Research, Fukushima Medical University, 1 Hikarigaoka, Fukushima City, Fukushima 960-1295, Japan

## Abstract

Oocytes have unique characteristics compared with other cell types. In mouse and human oocytes, two X chromosomes are maintained in the active state. Previous microarray studies have shown that the balance of the expression state is maintained in haploid oocytes. Here, we investigated transcripts using RNA-sequence technology in mouse and human oocytes. The median expression ratio between X chromosome and autosomal genes (X:A) in immature mouse oocytes increased as the gene expression levels increased, reaching a value of 1. However, the ratio in mature oocytes was under 1 for all expression categories. Moreover, we observed a markedly low ratio resulting from the bimodal expression patterns of X–linked genes. The low X:A expression ratio in mature oocyte was independent of DNA methylation. While mature human oocytes exhibited a slightly low X:A expression ratio, this was the result of the skewed high frequency of lowly expressed X-linked genes rather than the bimodal state. We propose that this imbalance between the expression dosages of X-chromosome and autosomal genes is a feature of transcripts in mammalian oocytes lacking X-chromosome inactivation.

Of the 200 different types of mammalian cells, oocytes exhibit many unique characteristics. Oocytes contain many maternal factors that are essential for reprogramming of sperm and differentiated cells^1^. Most of these maternal factors are deposited during oogenesis^2^,^3^, and the dynamic transition of the epigenomic state can be observed in the maternal genome^4^,^5^,^6^. One of the unique features of oocytes is the X-chromosome state; different from most somatic cells in mice and humans, the two X chromosomes in oocytes are maintained in the active state by repressing Xist, a large non-coding RNA that is essential for X-chromosome inactivation (XCI)^7^,^8^,^9^. In mice, Xist repression begins with primordial germ cells and persists during oogenesis^8^. Thus, two X-chromosomes are maintained in the active state for a long period during development. Recent studies in mice have revealed the dynamic changes to DNA methylation that occur during oogenesis and the pre-implantation phase^6^,^10^,^11^,^12^. However, although transcriptional transitions during the pre-implantation phase have been extensively analyzed^13^,^14^, the dynamic transcriptome has not been studied during oogenesis.

Many studies have examined the transcriptional state of embryonic stem (ES) cells in mice and humans^15^. Like oocytes, both X chromosomes in mouse female ES cells are maintained in the active state, resulting in the upregulation of X-linked genes compared with the levels of autosomal genes^16^. These results suggested that Ohno’s hypothesis, which proposes that the levels of X-linked genes are increased to twice those of autosomal genes to establish X chromosome dosage compensation between males and females^17^,^18^,^19^,^20^, seemed to be applicable to ES cells. Interestingly, a previous study showed that the ratio of expressed genes from the X chromosome and autosomes is less than 1 in mature mouse oocytes, which would result from the increased frequency of lowly expressed genes on the X chromosome compared with the frequency of autosomal-expressed genes^19^. However, this study was based on microarray technology, which does not account for the whole transcriptome. Moreover, the effects of maintenance of two active X chromosomes during oogenesis on the transcriptional state are poorly understood, and the X-linked gene dosage of human oocytes remains unknown.

Here, we conducted transcriptome analysis using high-throughput RNA sequencing (RNA-Seq) in nongrowing and fully grown oocytes in mice and examined the transcriptional state in human oocytes using published data. Transcriptome analysis in mice revealed that specific epigenetic factors were expressed at different stages of oogenesis to control transcription. Moreover, in immature mouse oocytes with two active X chromosomes, the expression ratio of X-chromosome genes to autosomal genes (X:A) was close to 1 (0.75–1.14) for highly expressed genes, indicating that the expression states of X-linked genes were comparable to those of autosomal genes. However, in mature oocytes, although the expression frequency of X-linked genes was significantly lower than that of autosomal genes, the X:A expression ratios in various expression categories were less than 1 (0.28–0.85). These results provided evidence for the decline of X-linked gene expression dosages without XCI during oogenesis in mice. Knockout of *Dnmt3l*, which is essential for de novo DNA methylation in female germ line cells, demonstrated that low expression ratio in mature oocytes was independent of DNA methylation. In contrast, in human oocytes, we observed a low X:A expression ratio and found that this low expression ratio resulted from the skewed expression state rather than bimodal transcription states in X-linked genes. Our analysis showed that the imbalanced expression dosage of X-chromosome and autosomal genes was a transcriptional feature in mature mouse and human oocytes.

## Results

### Identification of specific transcripts in immature and mature mouse oocytes

**Figure 1:**
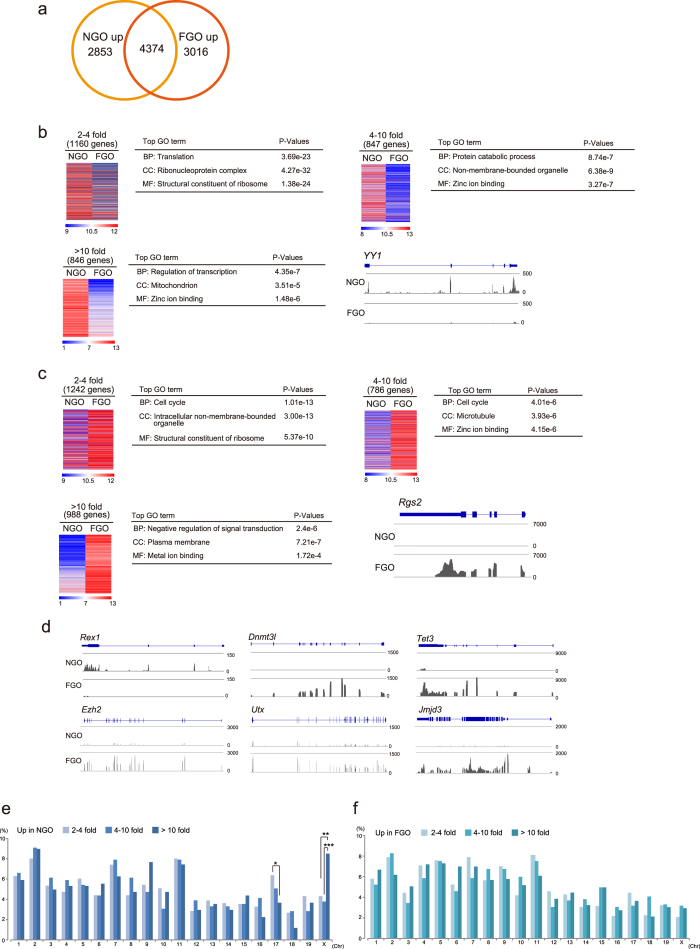
Identification of differentially expressed genes during oogenesis in mice. (**a**) Venn diagram showing the total number of differentially expressed genes based on TMM normalization values. Significant differences in FGO- and NGO-specific genes (3016 and 2853 genes, respectively) were observed (*P*-values: < 0.05 evaluated by t-test, and more than 2–fold change). (**b**,**c**) Heat maps showing differentially expressed genes that were categorized as highly expressed genes (>10 TMM values). Upregulated genes in NGO (**b**) and in FGO (**c**). Top Gene Ontology (GO) terms are shown in the heat map. RNA-Seq profiles indicated representative genes specific to NGO or FGO. (**d**) Expression state of representative transcription factors and epigenomic modifiers in NGO or FGO. (**e**,**f**) Chromosome distribution of upregulated genes in NGO (**e**) and FGO (**f**). Asterisks indicate significant differences by Fisher’s exacts test. *P < 0.01, **P < 0.002, ***P < 4.5e-5

During oocyte growth without cell division, dynamic epigenetic alterations are observed^6^,^11^. Thus, detailed transcriptomic analysis is important for defining the molecular features of oocytes. To identify the specific transcripts in nongrowing oocytes (NGO), which have no nuclear competency for development^21^, and fully grown oocytes (FGO), we conducted mRNA expression analysis combined with deep-sequence (RNA-seq) analysis. The samples were divided into groups based on their origins, and the correlation among samples in the same group was >0.9, indicating the existence of stage-specific transcripts (Supplementary Fig. 1). First, we investigated transcriptional changes in NGO, and FGO in mice. In this comparative analysis, we used trimmed mean of M-value normalization (TMM) to compare transcript transitions during oogenesis^22^. To identify representative genes at each stage, we focused on genes with expression levels greater than 10 TMM. The Venn diagram showed that 2853 and 3016 genes were specifically upregulated in NGO and FGO, respectively (Fig. 1a and Supplementary Table S1). The genes were divided into three groups based on fold changes: 2–4-fold (slight), 4–10-fold (moderate), and greater than 10-fold (high).

Gene ontology analysis revealed stage-specific transcriptional features. For example, in the biological process category of NGO upregulated genes, the transcriptional regulation-related genes such as *Yy1* were most dynamically changed^23^ (Fig. 1b). On the other hand, in FGO, the representative transcripts in the class of slight and moderate upregulated expression were ‘cell cycle’-related genes. Genes that are negatively associated with signal transduction such as *Rgs2* were identified as the most dynamically changed genes^24^ (Fig. 1c). These results suggest that transcriptional regulations might be greatly altered during oogenesis to gain nuclear competency.

We also identified differentially expressed transcription-, epigenomic-, and germ cell-related factors (Supplementary Fig. 2 and Fig. 1d). For example, *Rex1* was significantly upregulated in NGO (Fig. 1d and Supplementary Fig. 2b). During oogenesis, DNA methylation levels are dynamically increased^6^,^25^. Accordingly, *Dnmt3l* was specifically expressed in FGO (Fig. 1d and Supplementary Fig. 2e). Moreover, *Tet3*, which is essential for the DNA demethylation process after fertilization, was markedly upregulated in FGO (Fig. 1d and Supplementary Fig. 2f). Both histone H3 lysine 27 tri-methylation (H3K27me3) methyltransferease, *Ezh2*, and the demethylases *Utx* and *Jmjd3* were significantly upregulated in FGO (Fig. 1d and Supplementary Fig. 2g and 2j). Considering that H3K27me3 in zygotes is specifically imposed on the maternal genome, H3K27me3 demethylases might play an important role for epigenetic reprogramming of the paternal genome.

Next, we examined whether the differentially expressed genes are distributed among various chromosomes. Upregulated genes on the X chromosome in NGO were significantly concentrated in the >10-fold change category compared with the 2–4-fold and 4–10-fold categories (Fig. 1e). In contrast, the frequencies of each fold-change group were equally distributed among the chromosomes in FGO upregulated genes (Fig. 1f). These results indicated that dynamic alterations of X-linked genes occur during oocyte maturation.

### Decreased X-linked gene expression dosages during oogenesis in mice without XCI

**Figure 2:**
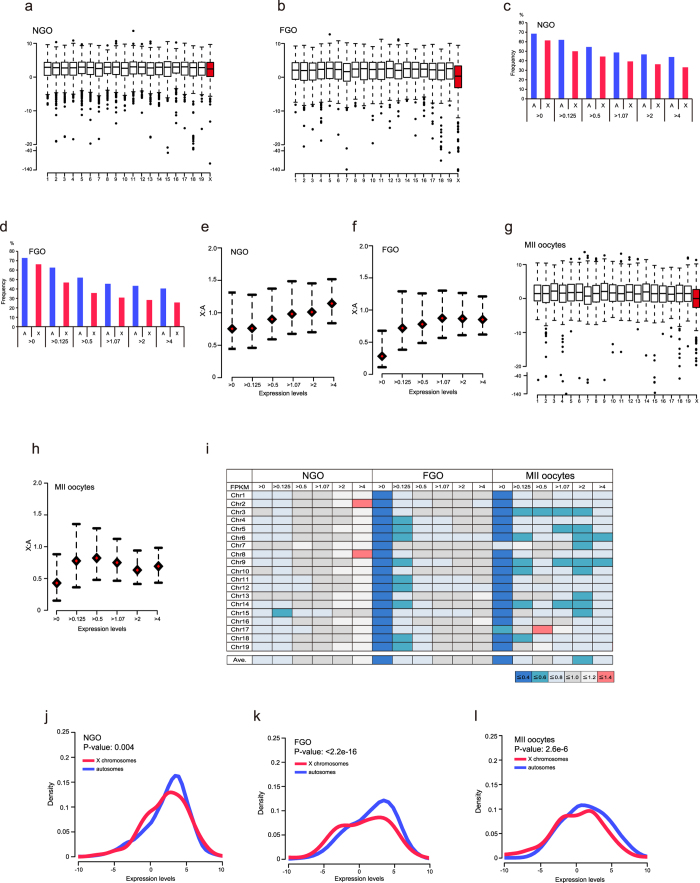
Expression ratios of X:A were lower in mature mouse oocyte compared with immature mouse oocyte. (**a**,**b**) Box plots of expression of genes with >0 FPKM in NGO (**a**) and FGO (**b**). (**c**,**d**) Expression frequencies of X-chromosome and autosomal genes in NGO (**c**) and FGO (**d**) based on the expression FPKM expression levels. The expression frequencies of X-chromosome and autosomal genes were significantly different for all expression categories (*P* < 0.01, Fisher’s exact test). (**e**,**f**) X:A expression ratios (all autosomes) by bootstrap analysis in NGO (**e**) and FGO (**f**). The red rhombus indicates the median. Error bars show 95% bootstrap confidence intervals. (**g**) Box plots of expression of genes with >0 FPKM in MII oocytes. (**h**) X:A expression ratio (all autosomes) by bootstrap analysis in MII oocytes. (**i**) X:A median expression ratio (each chromosome) of each FPKM category. Ave. means the average of each ratio according to the median expression levels. (**j**–**l**) Density plots of X-linked and autosomal genes with >0 FPKM in NGO (**j**), FGO (**k**), and MII oocytes (**l**). Expression levels are shown as log_2_ values. Distribution similarities between X-chromosome and autosomal genes were determined by the Kolmogorov-Smirnov test. The *P*-value was calculated by the Kolmogorov-Smirnov test.

*Xist* was repressed during oogenesis (Supplementary Fig. 3a and 3b). Sugimoto and Abe^8^ reported biallelic expression of X-linked genes in oocytes; therefore, these results indicate that both X chromosomes were in the active state in oocytes. In order to determine whether X-linked genes were upregulated, as is observed in female ES cells^16^, we investigated the expression states in autosomal and X-linked genes. In this assay, we used fragments per kilobase of exon per million mapped fragments (FPKM) as expression values to compare gene expression states within an identical sample^26^. We first examined the whole transcript level (>0 FPKM) in all chromosomes. The boxplot showed that the median expression levels of X chromosome genes in NGO were similar to those of each autosomal gene (Fig. 2a). However, interestingly, the expression levels of X chromosome genes in FGO were markedly different compared with those of each autosomal gene, and the median expression levels of X chromosomes were the lowest of all chromosomes (Fig. 2b). The frequencies of expressed genes in the X chromosome in each expression category were significantly low compared with those of autosomal genes in both the NGO and FGO, as well as in other tissues (Fig. 2c,d)^18^. These results indicated that the expression levels of X chromosome genes specifically declined during oogenesis.

We next investigated whether the X:A ratio in oocytes was more than 1. In NGO, the calculated X:A ratio by bootstrap analysis was different for each FPKM expression level (Fig. 2e). The X:A ratio was the lowest in genes including >0 FPKM expression levels (median: 0.75; Fig. 2e). However, the ratio increased as the gene expression levels increased, reaching nearly 1 in the group exhibiting the highest FPKM values (median values, 0.75–1.14; Fig. 2e). Therefore, in contrast to the results in female ES cells, the results of our analysis of gene expression states indicated that the expression dosage of X-linked genes in NGO was comparable to that of autosomal genes. While, in FGO, the ratio was dramatically decreased in genes in the >0 FPKM group (median value, 0.28; Fig. 2f). Interestingly, the ratio was always less than 1 (Fig. 2f). Although the ratio increased as the expression levels increased, the median ratio in FGO never reached 1 for all expression groups (median values, 0.28–0.85; Fig. 2f).

We also examined the gene expression states in ovulated haploid oocytes (MII oocytes) using published RNA-seq data. A previous report based on microarray analysis indicated that the median expression ratio of FGO was 0.7, whereas that of haploid oocytes was 0.9, suggesting that the expression states of X chromosome genes might change during oocyte maturation^19^. The RNA-seq data showed that the expression states of X chromosome genes in MII oocytes were similar to those of FGO, indicating that the median expression level of the X chromosome genes was the lowest of all chromosomes (Fig. 2g). The bootstrap analysis also showed that the X:A expression ratios were low in all expression categories (median values, 0.37–0.78; Fig. 2h). Comparison of X chromosome expression with each autosome, the X:A expression ratios (calculated by the median in the 0 > FPKM category) were 0.21–0.32 and 0.22–0.6 in FGO and MII oocytes, respectively, whereas they were 0.64–0.91 in NGO (Fig. 2i), thereby supporting the bootstrap analysis results (Fig. 2e,f: X chromosomes genes vs. all autosomal genes). These results indicated that the low X:A expression ratio is a feature of mature oocyte transcripts in mice.

Next, we examined whether the dramatically low ratio in FGO and MII oocyte was associated with the low expression levels of X-linked genes. Density plots in NGO, FGO, and MII oocytes using genes with >0 FPKM showed different distributions between NGO and mature oocytes (FGO and MII oocytes). In NGO, the distributions of X chromosome transcripts were similar to those of all autosomal transcripts (Fig. 2j). Statistical analysis using Kolmogorov-Smirnov tests to evaluate the distribution differences of expressed genes between the X chromosome and each autosome revealed that out of 19 autosomes, the transcript distributions of 13 autosomes were not significantly different (P > 0.01) to those of X chromosomes (Supplementary Fig. 4). However, in contrast to previous observations, density plots showed a clear bimodal distribution in X-linked gene expression in FGO and MII oocytes compared with that in NGO (Fig. 2k,l). The Kolmogorov-Smirnov tests indicated that the *p*-value was markedly lower in FGO and MII oocytes than in NGO (<2.2e-16 in FGO, 2.6e-6 in MII oocytes vs. 0.004 in NGO). Furthermore, all autosomes in FGO and all except for chromosome 7 in MII oocytes showed significantly different distributions compared with the distribution of X chromosome transcripts (Supplementary Figs 5 and 6).

In order to gain further insight into features of lowly expressed genes in FGO that shows markedly low expression in X chromosome transcripts, we further examined the chromosome distributions of lowly expressed X-linked genes. Genes with −5–0 (log_2_ values) FPKM showing a clear single peak in the density plot (Fig. 2k) were widely distributed on X chromosomes (Supplementary Fig. 7). Interestingly, genes exhibiting very low expression (under −5 FPKM) were concentrated on XqA2 and XqA3.1 regions (Supplementary Fig. 7). Although the functions of most of these genes are unknown (i.e., unannotated), they may have common features.

Taken together, these results suggest that in mature oocytes, the dramatically low X:A expression ratio resulted from bimodal transcriptional states in X-linked genes, and that the oocytes exhibited an imbalance between the expression dosages of X-chromosome and autosomal genes.

### DNA methylation was dispensable for the decrease in X-linked gene expression dosages

Our transcriptome analysis in NGO and FGO identified specific expression patterns for some epigenetic factors during different stages of oogenesis. Because DNA methylation is known to play an important role in transcription^27^,^28^, we next examined whether the observed changes in X-linked gene expression dosages were associated with DNA methylation using *Dnmt3l*-knockout (KO) oocytes (FGO) data^25^, which are deficient in DNA methylation. First, we examined the expression states in DNA methylation-deficient oocytes. The boxplot (>0 FPKM) showed no apparent differences between wild-type (WT) and mutant oocytes, and the median expression levels of X chromosomes were the lowest, consistent with the results shown in Fig. 2b (Supplementary Fig. 8a and 8b). Interestingly, the frequency of expressed genes with >0 FPKM in X chromosomes was not altered between wild-type (WT) and *Dnmt3l*-KO oocytes (WT: 800 genes [71%] vs. *Dnmt3l* KO: 806 genes [71%], *P*-value: 0.8; Supplementary Fig. 8c). In contrast, there was a significant increase in the frequency of expressed genes with >0 FPKM in autosomal genes (WT: 17638 genes [79%] vs. *Dnmt3l* KO: 17935 genes [80%], *P*-value: 0.0006; Supplementary Fig. 8c). These results indicated that loss of DNA methylation did not affect X-linked genes, but did affect autosomal genes.

We next examined the X:A expression ratio by bootstrap analysis. In WT FGO, the ratio was quite low (0.17, 0 < FPKM; Supplementary Fig. 8d). The ratio increased in genes with greater than 0.125 FPKM, exhibiting results similar to those of our experiment (Supplementary Fig. 8d). The X:A ratio in *Dnmt3l*-KO FGO was also low (0.18, >0 FPKM; Supplementary Fig. 8d). The ratio increased as the expression levels increased, as was observed for WT oocytes (Supplementary Fig. 8d).

In order to verify the expression states in DNA methylation-deficient oocytes, we examined the distributions of expressed genes in the X chromosome and autosomes. Density plots showed that the expression patterns of X-chromosome and autosomal genes in *Dnmt3l*-KO FGO were similar to those in WT FGO (Supplementary Fig. 8e and 8f). The *p*-values by Kolmogorov-Smirnov test against the distribution between WT and *Dnmt3l*-KO oocytes were 0.4 (X chromosome) and 0.2 (autosomes), respectively (Supplementary Fig. 8e and 8d). In contrast, the test against the distribution between X-chromosome and autosomal genes was significantly different (WT and *Dnmt3l*-KO: <2.2e-16, respectively, data not shown). These results demonstrated that loss of DNA methylation was independent of the decreased X-linked gene expression dosages in mature oocytes.

### XCI-related gene expression states in human oocytes

Previous studies have indicated that imprinted XCI does not occur in human pre-implantation embryos, implying that the regulatory mechanisms of *XIST* activation may differ from those of mice^29^,^30^. Using published RNA-Seq data from human MII oocytes^13^, we examined the expression states of XCI-associated genes. Similar to the results in mice, *XIST* was repressed (Supplementary Fig. 9a). Moreover, *XACT*, which has recently been identified as a marker of the active X chromosome marker in pluripotent stem cells^31^, was not expressed in oocytes (Supplementary Fig. 9a). These results suggested that like mouse oocytes, the *XIST* repression mechanism in human oocytes differs from that in pluripotent stem cells.

Next, we examined the expression of *RLIM/RNF12*, a *Xist* activator identified in mice, in human oocytes^32^,^33^. Interestingly, the expression level of maternal *RNF12* was extremely low (Supplementary Fig. 9a), even though the gene was abundantly expressed in immature and mature mouse oocytes (Supplementary Fig. 9b). Because *XIST* expression in human pre-implantation embryos is delayed compared with that in mouse embryos^30^, *RNF12* may be required during the late pre-implantation stage in humans.

### RNA-Seq data in human mature oocytes confirmed the decrease in X-linked gene expression dosages without XCI

**Figure 3:**
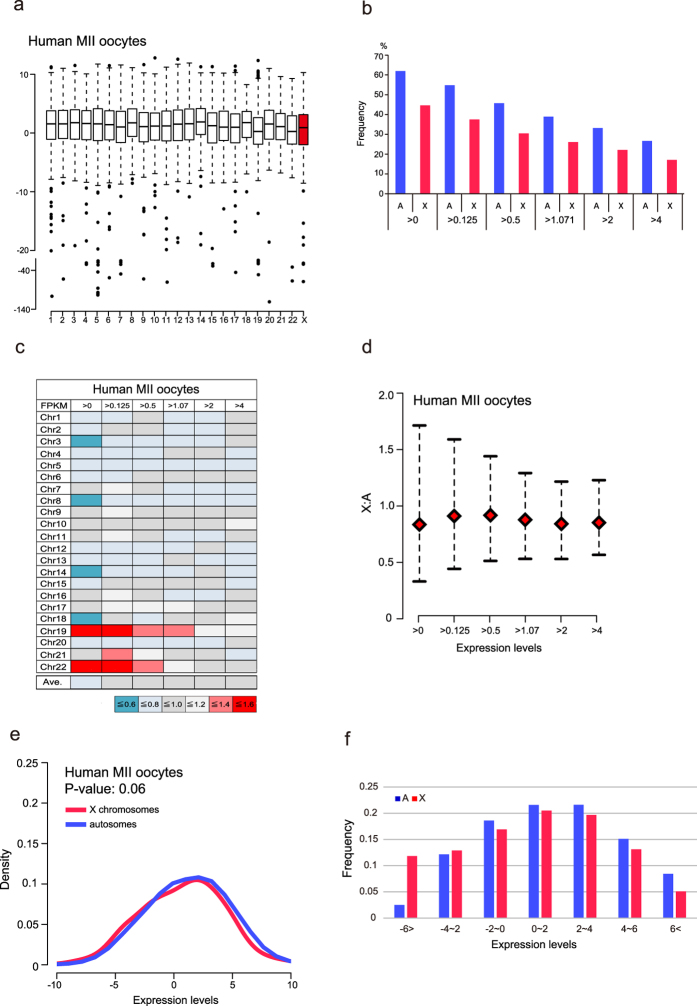
Low X:A expression ratios in human oocytes. (**a**) Box plots of expression of genes with >0 FPKM in human MII oocytes. (**b**) The frequencies of expressed genes based on FPKM expression levels. The frequencies of X-linked and autosomal genes were significantly different for all categories (Fisher’s exact test, *P*-value < 0.01). (**c**) X:A median expression ratios (each chromosome) determined of each FPKM categories. Ave. means the average of each ratio according to the median expression levels. (**d**) X:A expression ratios (all autosomes) by bootstrap analysis in human MII oocytes. The red rhombus indicates the median. Error bars show 95% bootstrap confidence intervals. (**e**) Density plots of X-linked and autosomal genes with >0 FPKM. The *P*-value was calculated by the Kolmogorov-Smirnov test. (**f**) Frequencies based on log_2_ expression levels. The frequency on X chromosomes was higher than that on autosomes for genes exhibiting low expression levels, while the opposite was true for genes exhibiting high expression levels.

Our analysis of mouse oocytes using RNA-Seq revealed that the expression states of X-linked genes in mature oocytes of mice showed bimodal transcription, resulting in a low X:A ratio without XCI. Therefore, we asked whether the X-linked gene expression dosage was similarly imbalanced in human oocytes. We first examined whole transcript (FPKM > 0) levels in all chromosomes. In contrast to the results for mice, the median expression levels of X chromosomes were not the lowest (Fig. 3a). However, as seen in mouse oocytes, the number of expressed X-linked genes was significantly lower than that of autosomal genes for all FPKM expression categories (Fig. 3b). These results suggest that the X chromosome expression states of human oocytes might be different from those of mice.

Next, we asked whether the imbalanced X:A expression ratio in mice is also observed in human oocytes. First, we compared the median expression levels between the X chromosome and each autosome. As shown in Fig. 3c, there was high variation in the median expression ratios in each chromosome comparisons (0.51–1.6) in the >0 FPKM category, and the average ratio of the medians in the >0 FPKM category was 0.8 (Fig. 3c), showing a tendency of a slightly low expression ratio. Next, we examined the X:A expression ratio by bootstrap analysis (X chromosomes genes vs. all autosomes genes). Although we observed an increase in the X:A ratio when genes with high expression were used for analysis, the median ratios were less than 1 for all categories (0.74–0.87; Fig. 3d). These results indicated that the X-linked gene dosage slightly decreased without XCI in human oocytes.

In human MII oocytes, the median expression ratio of genes with >0 FPKM determined by bootstrap analysis was not as dramatically decreased as that in mice MII oocytes (mice: 0.37, humans: 0.74). In order to explain the low X:A expression ratio, we created density plots for human oocytes. Interestingly, bimodal transcription was not found in X-linked genes in humans (Fig. 3e). The Kolmogorov-Smirnov test showed the similarity of expressed gene distribution patterns between the X chromosome and autosomes (*p*-value: 0.06, Fig. 3e). However, a slightly high density was observed in autosomal genes in high expression-level categories (Fig. 3e). Thus, we examined the expression frequencies based on the expression ranges from <−6 FPKM to >6 FPKM. The analysis showed that the frequency of X-linked genes in low-expression categories was higher than that of autosomal genes, while the opposite was true in high-expression categories (Fig. 3f). These results indicated that the skewed expression states between X-chromosome and autosomal genes caused the imbalanced X:A expression ratio without XCI.

## Discussion

In most of the somatic cells in female mammals, one of the two X chromosomes is inactivated to balance the expression dosage against their male counterparts. However, *in vivo*, oocytes have two active X chromosomes, and this activation state persists from the embryo stage through adulthood. Interestingly, in our study, we found that the median X:A expression ratio was low in both humans and mice. In mice, the expression of X-linked genes exhibited a bimodal pattern, while skewed, but not bimodal, expression states were observed in human oocytes. Therefore, these data demonstrated that the imbalance in expression states of X-linked gene dosages in mammalian oocytes is a feature of oocytes. However, these results also raise some interesting questions that remain to be addressed.

In mature mouse oocytes, the X:A expression ratio in the genes with 0 > FPKM was substantially low, whereas this ratio was only slightly low in human oocytes. Although the specific reason for the extremely low expression ratio in mature mouse oocytes is still unknown, consideration of the X chromosome state after fertilization in both species might help to resolve this. In mice, the maternal X chromosome is maintained in an active state by repression of *Xist*. On the other hand, maternal *XIST* is expressed and not imprinted in human preimplantation embryos. Thus, to robustly repress maternal *Xist*, chromatin of the X chromosome in mice might be more condensed than that in humans.

In human oocytes, we found that the X:chr19 expression ratios were high in all of the FPKM categories (Fig. 3c). In the light of the expression ratios of the other autosomes, chr19 transcriptional activity is specifically low. However, the gene density of chr19 is the highest among all chromosomes, and transcription factors are enriched on the chromosome^34^. Considering that human oocytes cannot immediately reprogram the somatic cell transcription pattern after nuclear transfer^35^, the maternal factors required for nuclear reprogramming of the differentiated genome might not be abundantly deposited.

The epigenetic modification states are dynamically altered during oogenesis. In this study, we demonstrated that DNA methylation is independent of the low X:A expression ratio in mature mouse oocytes. During oogenesis, histone modifications were also dramatically changed. Therefore, there might be multiple machineries that function together to attenuate X-linked gene transcript levels. Another interesting question is whether the observed low expression state on X chromosomes in mice results from an allelic imbalance. Sugimoto and Abe^8^ demonstrated that the expression states of X-linked genes in oocytes were biallelic by RT-PCR analysis. However, the RT-PCR analysis using single cell was not quantitative assay. Thus, allele-specific quantitative expression analysis should help to elucidate this question.

The biological meaning of the decrease in X-linked gene expression levels in mature oocyte is unknown. However, the imbalance in X-linked gene expression dosages may be a useful marker for evaluating oocyte quality with nuclear competency. Therefore, massive analysis using RNA-Seq from individual oocytes collected from humans will provide critical information in the management of reproductive disorders.

## Methods

### Oocyte collection

All mice were maintained and used in accordance with the Guidelines for the Care and Use of Laboratory Animals of the Japanese Association for Laboratory Animal Science and the National Research Institute for Child Health and Development (NRICHD) of Japan. All animal experiments were performed according to protocols approved by the Institutional Animal Care and Use Committee of the NRICHD (Permit Number: A2006-009). Adult female and male C57BL/6N mice were purchased from Clea Japan. NGO were collected from the ovaries of newborn female mice at 3 days of age. The ovaries were immersed in M2 medium containing collagenase (final concentration: 1 mg/mL; WAKO, Tokyo, Japan) and incubated for 10 min at 37 °C. Ovaries were washed with phosphate-buffered saline (PBS) and then incubated in 0.05% trypsin-EDTA for 5 min at 37 °C. After pipetting of ovaries, NGO (diameter: 15–30 μm) were collected using a micromanipulator. Before FGO collection, mice were treated with 5 IU pregnant mare serum gonadotropin (PMSG). At 44–48 h after PMSG injection, FGO were collected in M2 medium containing 2 mM dbcAMP (Sigma; St. Louis, MO, USA). The 30 FGO and 200 NGO were considered as one biological replicate, respectively, and a total of three replicates were analyzed per group.

### Immunofluorescence combined with *Xist* RNA fluorescence *in situ* hybridization (FISH)

NGO and somatic cells from ovaries were placed on glass slides. The samples were fixed with 2% paraformaldehyde (PFA) containing 0.25% Triton X-100 in PBS for 5 min at room temperature. After washing with PBS, the samples were blocked in 1% bovine serum albumin (BSA) in PBS containing 1.3 U/mL RNaseOUT (Life Technologies, Carlsbad, CA, USA) for 30 min at room temperature (RT). After washing, the samples were incubated with primary antibodies (anti-MVH, Abcam ab13840, diluted 1:100 in blocking buffer containing 1.3 U/mL RNaseOUT) for 1 h at RT. After washing in PBS, the samples were incubated for 1 h at RT with Alexa Flour 488-conjugated anti-rabbit IgG secondary antibodies (1:300, Life Technologies). After washing in PBS, the samples were dehydrated sequentially in 70% and 100% ethanol and then air-dried. The samples were then subjected to Xist FISH. Hybridization buffer containing an Xist probe (provided by T. Sado, Kinki University) was prepared using a Nick Translation Kit (Abbott; Abbott Park, IL, USA) and Cy3-dUTP (GE Healthcare Life Sciences; Fairfield, CT, USA) and was then applied to the slides. The nuclei were stained with 1 μg/mL 4′,6-diamidino-2-phenylindole (DAPI), and the embryos were placed on a glass slides and observed with an LSM510 laser-scanning confocal microscope (Carl Zeiss; Oberkochen, Germany).

### Sequencing library preparation

Total RNA pooled from each sample was extracted using a Qiagen RNeasy Micro Kit (Qiagen; Valencia, CA, USA), and the remaining DNAs were degraded by DNase treatment. For construction of sequencing libraries, we used an Ovation Single Cell RNA-Seq System (NuGEN, West Cumbria, UK). Briefly, first-strand cDNAs were selectively synthesized from the purified RNAs using proprietary whole-transcriptome primers for nonribosomal RNAs. Second-strand cDNAs were then synthesized from the first-strand cDNAs using the second-strand primer. After ligation of the reverse adaptor, libraries were enriched by polymerase chain reaction (PCR). Quality checks of the libraries were carried out using a Bioanalyzer (Agilent Technologies; Santa Clara, CA, USA).

### Sequencing

Strand-specific, paired-end sequencing (length: 100 bp) was carried out using a Hiseq system (Illumina, Inc.; San Diego, CA, USA), with six samples per lane.

### Quality check of raw reads

Output raw reads in fastq format were subjected to quality check using the FastQC program.

### Trimming of remaining sequencing adaptors and filtering out low-quality reads

Before aligning read sequences onto the reference genome (mm10), the sequencing adaptors (Illumina), which were used in the sequencing library preparation, and the low-quality read sequences evaluated by Quality Value (QV) Score were eliminated using Trimmomatic-0.30 (Ref-1). In this case, the average QV Score was calculated by the sliding window method (window size: 4 bp) from both ends of each read sequence. When the average score was 15 (error rate: approximately 3.2%) or less than 15, the four successive nucleotide sequences were discarded from the read. In addition, error-prone short reads that were shorter than 36 bp (by default) were also discarded. Read quality data for samples after trimming were also reported and summarized by FastQC.

### Alignment of the filtered reads onto the reference genome and transcripts

For alignment of trimmed-FASTQ data onto mm10, we used Tophat 2.0.11 (bowtie2-2.2.1)^36^, which can adequately align sequence reads onto the location including splice sites in the genomic sequence. For the Tophat-Cufflinks strategy, reference sequences and annotations (mm10) were downloaded from the iGenome site (Illumina, http://support.illumina.com/sequencing/sequencing_software/igenome.html). For the analysis using AvadisNGS, UCSC Gene and Transcripts version 2013.03.06 was used as described below.

### Quantifying the gene expression level

BAM format data yielded by Tohat 2.0.11 were subjected to successive analyses using Cufflinks-2.2.1 or AvadisNGS 1.6 (Agilent). The counts of raw reads allocated for each gene/transcript, which links to UCSC transcripts, were normalized to the FPKM value (Cufflinks-2.2.1)^26^ or using the TMM method (AvadisNGS 1.6)^37^. Normalized values were described as log_2_ values.

### Characterization of expressed genes and estimating the X:A ratio

Genes that were expressed in all replicates in each group were analyzed. The genes from triplicate data for each cell type (FGO and NGO) were averaged. The X:A ratio was calculated according to the following expression level cutoffs (>−∞, >−3, >−1, >0.1, >0.5, >1, >2, and >3 in log_2_ FPKM values from Cufflinks-2.2.1). In the case of TMM, negative log_2_ values are automatically collapsed to 0 using the AvadisNGS 1.6 software. The cutoffs for TMM were thus defined as follows: >0, >1, >3, >5, >7, >9, and >11. Median X:A ratios were calculated by bootstrapping with 2000 replications, and 100 expression values were randomly selected for each time from X-linked and autosomal genes using the ‘sample’ function of R. Ninety-five percent bootstrap intervals were also calculated using the ‘quantile’ function of R.

### Distribution of gene expression

We depicted distributions of gene expression of normalized values (>−∞), using the ‘density’ function in R. Differences of expression distributions between X-linked and autosomal genes were evaluated by Kolmogorov-Smirnov tests using the ‘ks.test’ function in R.

### Functional annotation using the Gene Ontology terms

In order to investigate the biological features underlying the differential expression patterns between NGO and FGO, differentially expressed gene sets were selected according to 2-fold up- or downregulation of genes (*p*-value < 0.05 using t-tests) between NGO and FGOamples. The gene sets were subjected to DAVID bioinformatics analysis. All the procedures were performed using TMM data in AvadisNGS 1.6.

### Analyses using publicly available data

For comparison of the X:A ratios between mice and humans, we obtained publicly available RNA-Seq data and processed these data according to the library layout and reference genomes. Dnmt3l-deficient mouse oocyte (DRX001170) and WT mouse oocyte (DRX001169) data were downloaded from the following sites: https://trace.ddbj.nig.ac.jp/DRASearch/experiment?acc=DRX001169 and https://trace.ddbj.nig.ac.jp/DRASearch/experiment?acc=DRX001170, respectively. Mouse data for MII oocytes were downloaded from http://www.ncbi.nlm.nih.gov/geo/query/acc.cgi?acc=GSE44183. In addition, we analyzed human oocyte data (http://www.ncbi.nlm.nih.gov/geo/query/acc.cgi?acc=GSE36552), using hg19 as the reference.

## Additional Information

**Accession code**: The original data of RNA-seq from NGO and FGO have been deposited in DDBJ at http://cibex.nig.ac.jp/index.jsp with accession number (submission ID): fukuda911-0001.

**How to cite this article**: Fukuda, A. *et al.* Imbalance between the expression dosages of X-chromosome and autosomal genes in mammalian oocytes. *Sci. Rep.* **5**, 14101; doi: 10.1038/srep14101 (2015).

## Supplementary Material

Supplementary Information
