# Supplementary material for: Imbalance between the expression dosages of X-chromosome and autosomal genes in mammalian oocytes
Source: Sci Rep. 2015 Sep 15;5:14101. doi: 10.1038/srep14101 (PMC4572927; doi:10.1038/srep14101)
Supplement: Supplementary Information [file srep14101-s1.pdf]

Imbalance between the expression dosages of X-chromosome and autosomal genes in mammalian oocytes

(Supplementary data)

Atsushi Fukuda, Motohiko Tani, Ryo Matoba, Akihiro Umezawa, Hidenori Akutsu

Supplementary Figures

Fig. 1. RNA-seq quality among biological replicates.

(a) Unsupervised clustering analysis by raw reads. Each sample was categorized according to their origin. (b) The correlation coefficient based on the raw reads in each group.

Fig. 2. Expression states of transcription-, pluripotency-, and epigenetics-related genes during mouse oogenesis. (a and b) Transcription factors upregulated in FGO (a) and NGO (b). (c) Germ cell factors. (d) Histone deacetylase/acetylase. (e) DNA methyltransferase. (f) DNA demethylation-associated genes. (g and h) Histone demethylase upregulated in FGO (g) and NGO (h). (i) Histone methylase. (j) Polycomb repressive group (PRC)-1 and -2-associated genes. The expression levels were based on TMM normalization.

Fig. 3. *Xist* expression analysis by RNA FISH combined with immunofluorescence in NGO and RNA-seq. (a) Cells positive for the germ cell marker MVH (green) were identified as NGOs. DNA was stained with DAPI (blue), and Xist is shown in red. Scale bar: 10 µm. Total 18 NGO were analyzed. (b) Expression state of *Xist* in NGO and FGO.

Fig. 4. Density plot of expressed genes in X chromosomes and each autosome in NGO. Density plots of X-linked and autosomal genes with > 0 FPKM. The P-value was calculated by the Kolmogorov-Smirnov test.

Fig. 5. Density plot of expressed genes in X chromosomes and each autosome in FGO.

Density plots of X-linked and autosomal genes with > 0 FPKM. The P-value was calculated by the Kolmogorov-Smirnov test.

Fig. 6. Density plot of expressed genes in X chromosomes and each autosome in MII oocytes in mice.

Density plots of X-linked and autosomal genes with > 0 FPKM. The P-value was calculated by the Kolmogorov-Smirnov test.

Fig. 7. Distributions of lowly expressed X-linked genes in FGO. X-linked genes showing low expression states by density plot analysis were mapped on the X chromosome.

Fig. 8. Effects of DNA methylation on the X:A expression ratio in FGO. (a) Box plots of expression of genes with > 0 FPKM in wild type (WT) (a) and *Dnmt3l* KO (b) mice. (c) Frequency of expressed genes with > 0 FPKM. *P*-values were calculated by Fisher's exact test. (d) X:A expression ratios (all autosomes) by bootstrap analysis in WT and Dnmt3l-KO FGO. Red and green rhombi indicate medians. Error bars show 95% bootstrap confidence intervals. (e and f) Density plot analysis of X-linked genes (e) and autosomal genes (f) between samples. The *P*-values were calculated by the Kolmogorov-Smirnov test.

Fig. 9. Expression states of *Xist* regulators in human and mouse oocytes. (a) Expression states of *PGK1*, *RNF12*, *XACT* and *XIST*. (b) Mouse *Rnf12* expression states in FGO and NGO.

Supplementary Fig. 1

a

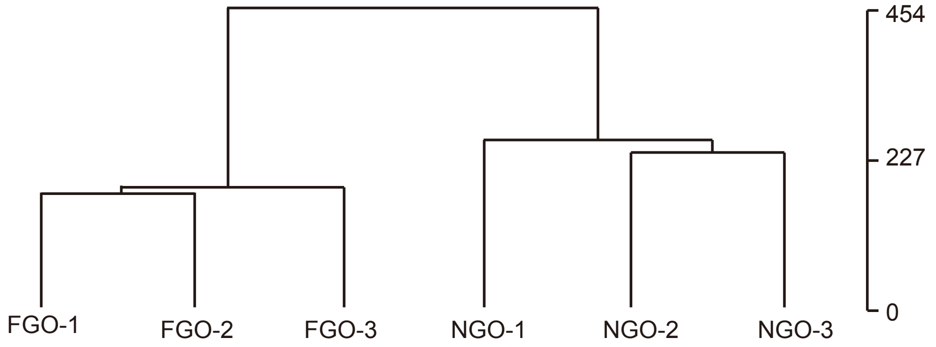

b

	FGO-1	FGO-2
FGO-1		
FGO-2	0.97	
FGO-3	0.97	0.96

	NGO-1	NGO-2
NGO-1		
NGO-2	0.92	
NGO-3	0.94	0.91

Supplementary Fig. 2

a

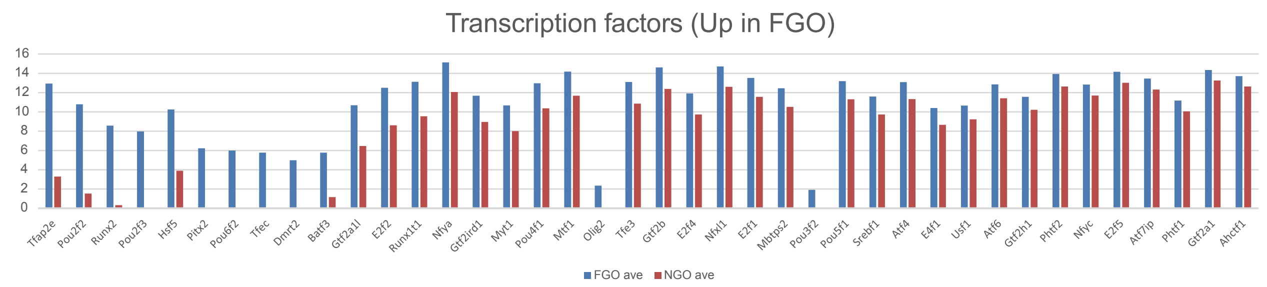

b

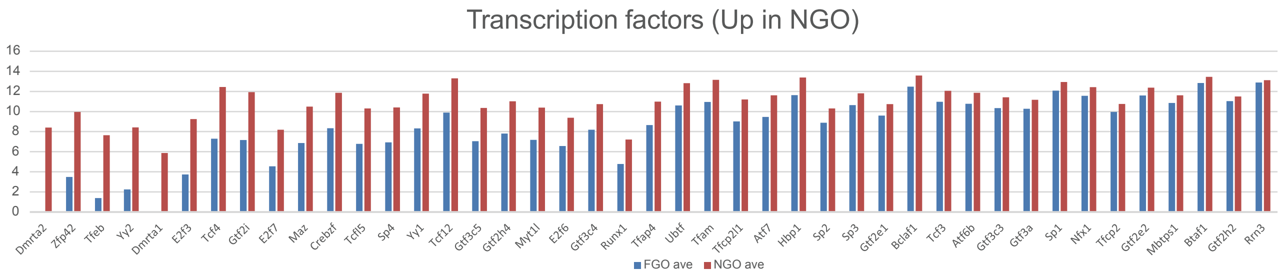

c

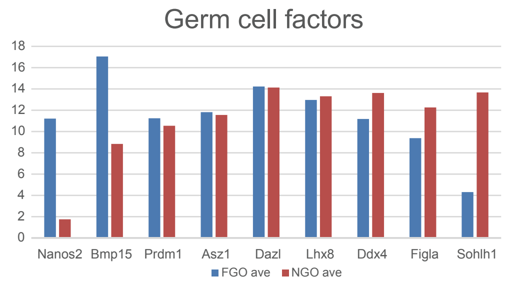

d

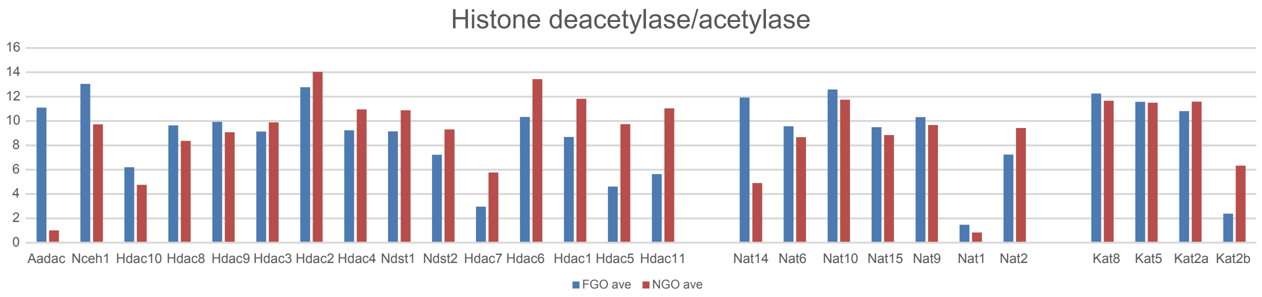

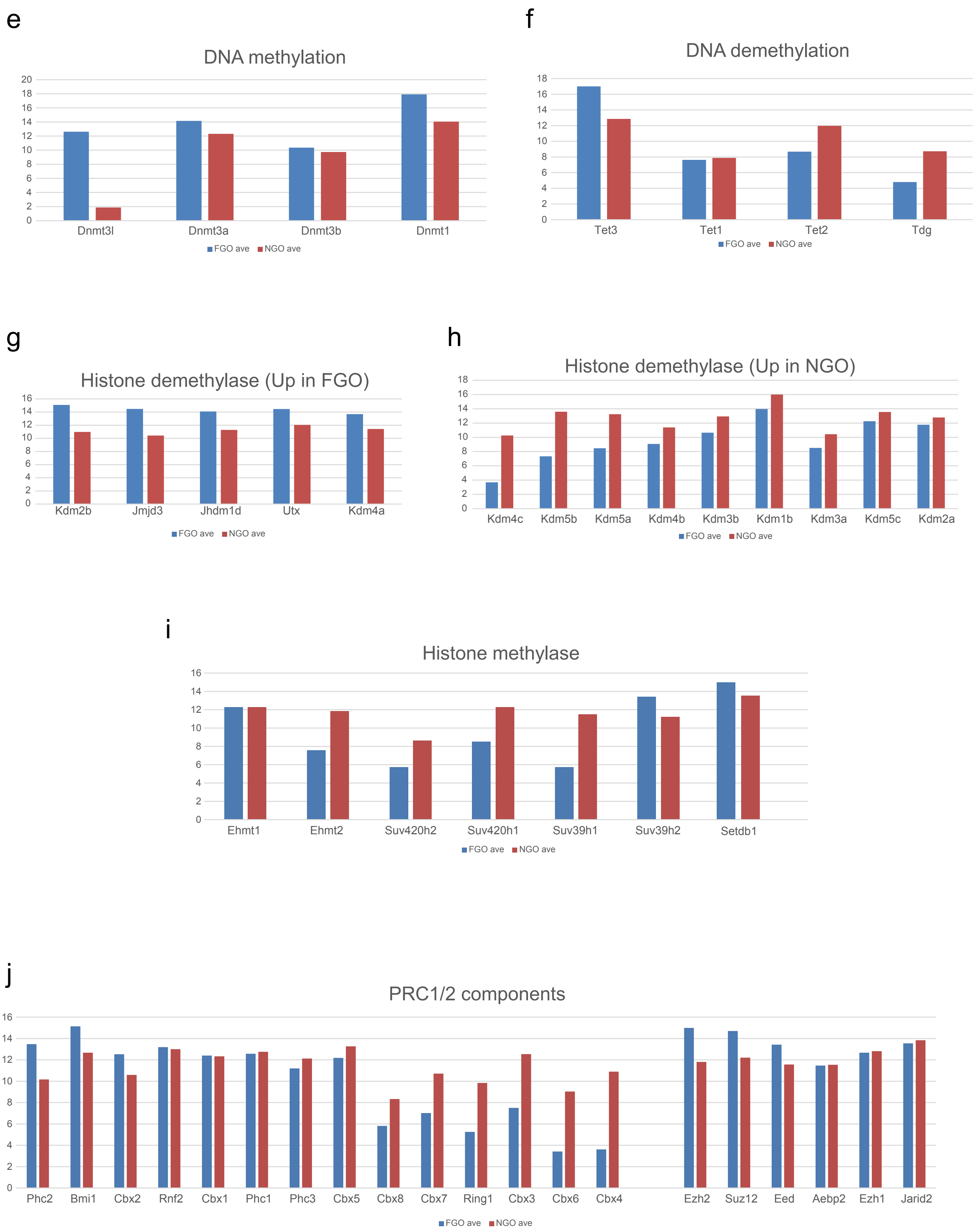
e
DNA methylation
Dnmt3l
Dnmt3a
Dnmt3b
Dnmt1
FGO ave
NGO ave
f
DNA demethylation
Tet3
Tet1
Tet2
Tdg
FGO ave
NGO ave
g
Histone demethylase (Up in FGO)
Kdm2b
Jmjd3
Jhdm1d
Utx
Kdm4a
FGO ave
NGO ave
h
Histone demethylase (Up in NGO)
Kdm4c
Kdm5b
Kdm5a
Kdm4b
Kdm3b
Kdm1b
Kdm3a
Kdm5c
Kdm2a
FGO ave
NGO ave
i
Histone methylase
Ehmt1
Ehmt2
Suv420h2
Suv420h1
Suv39h1
Suv39h2
Setdb1
FGO ave
NGO ave
j
PRC1/2 components
Phc2
Bmi1
Cbx2
Rnf2
Cbx1
Phc1
Phc3
Cbx5
Cbx8
Cbx7
Ring1
Cbx3
Cbx6
Cbx4
Ezh2
Suz12
Eed
Aebp2
Ezh1
Jarid2
FGO ave
NGO ave

Supplementary Fig. 3

a

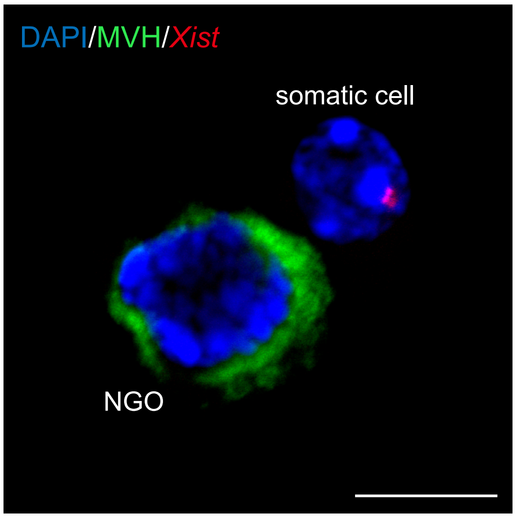

b

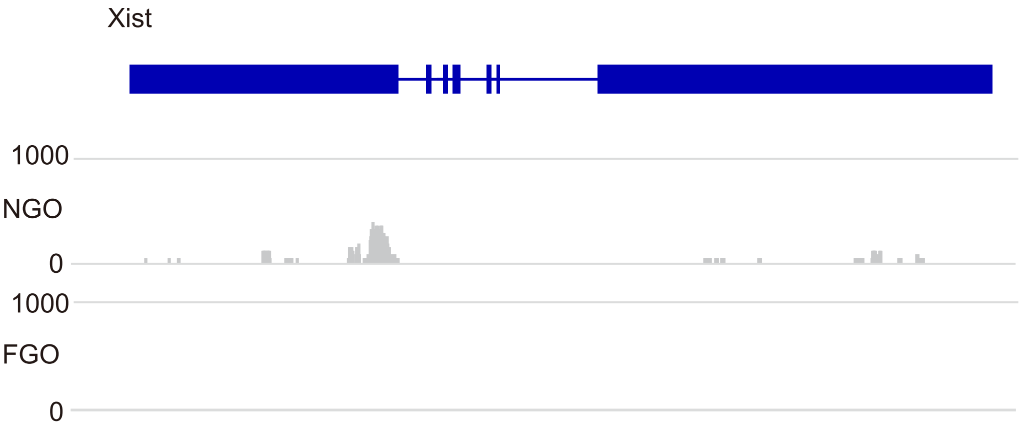

Supplementary Fig. 4

NGO

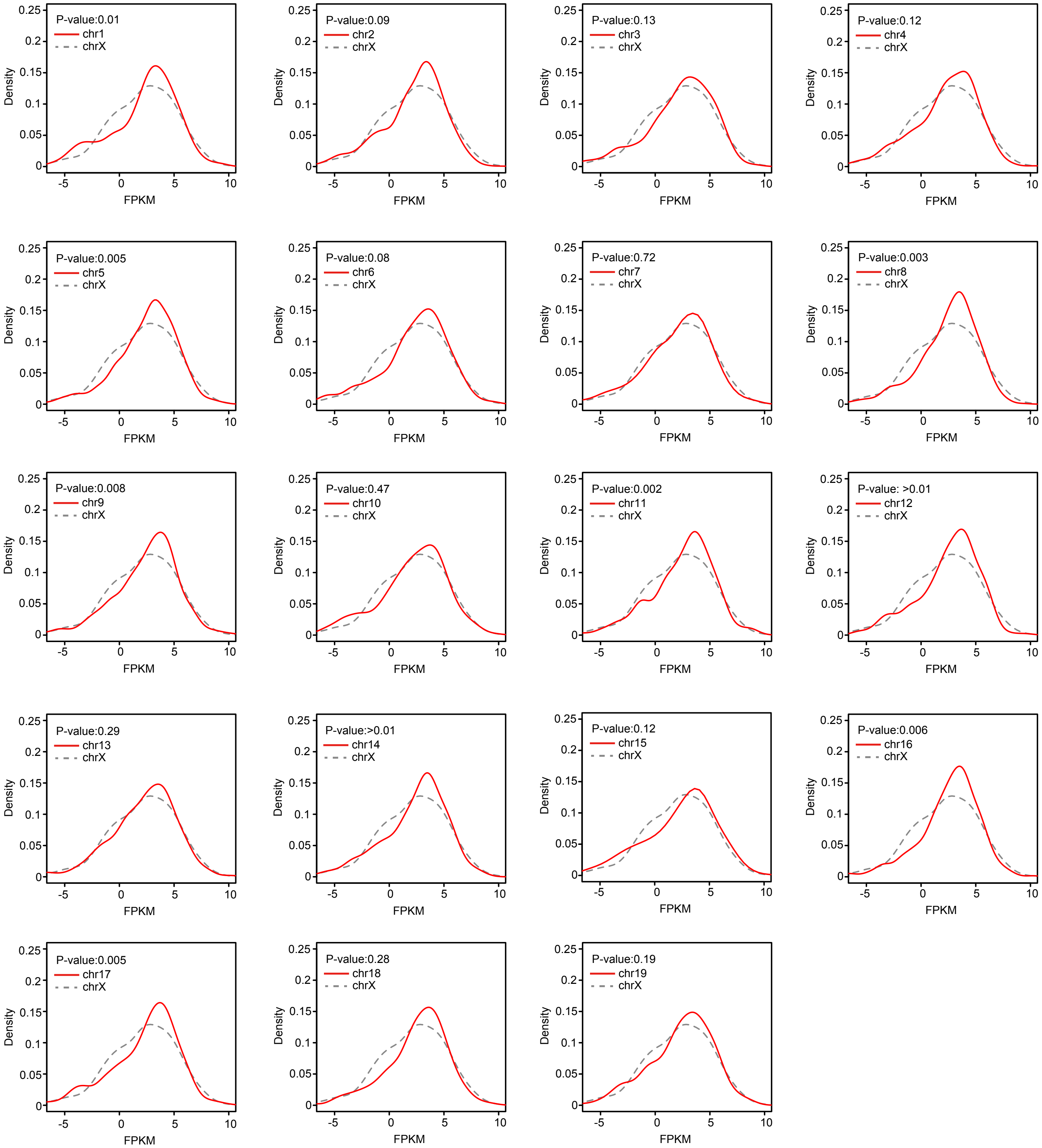

Supplementary Fig. 5

FGO

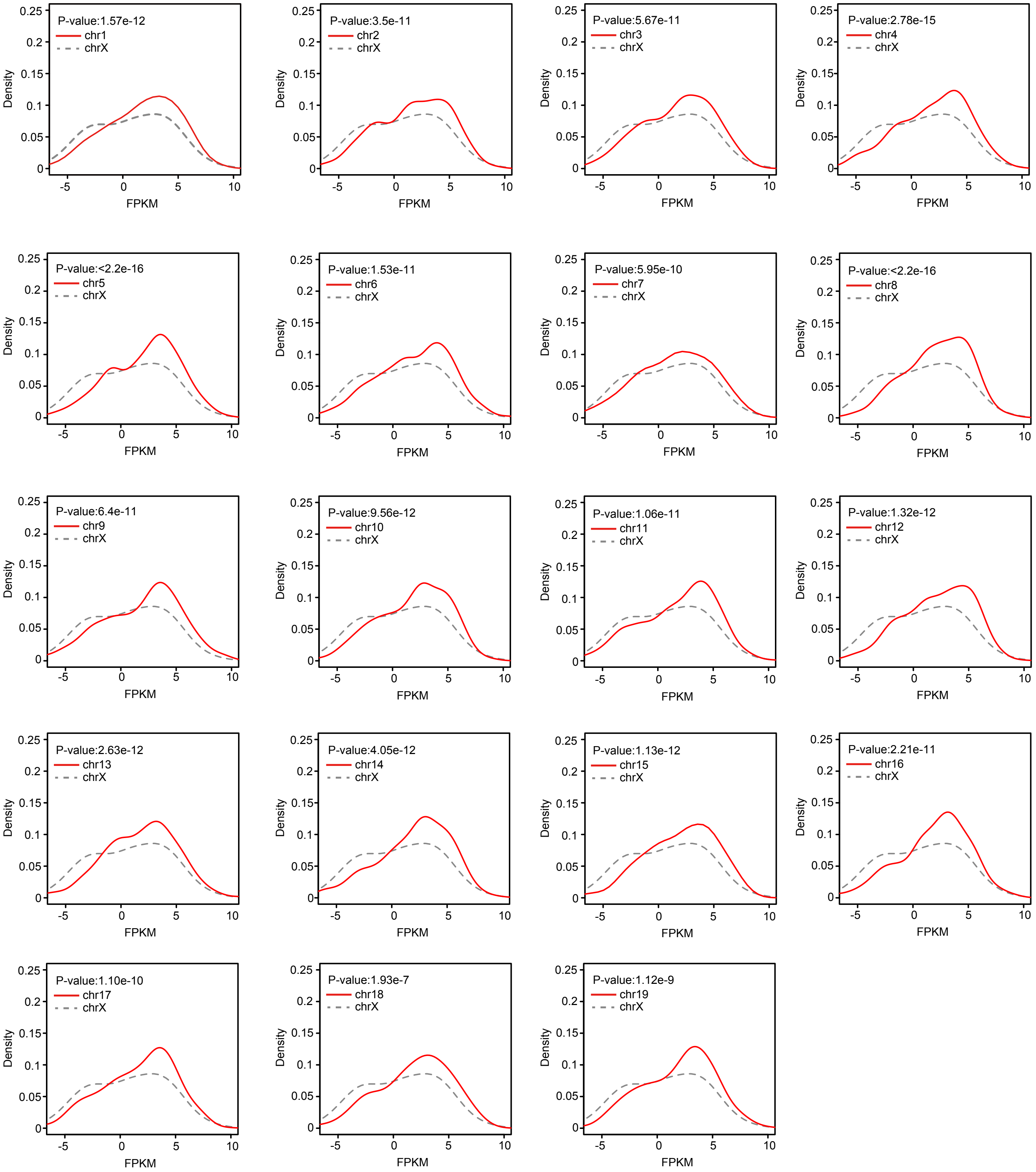

Supplementary Fig. 6

MII oocytes

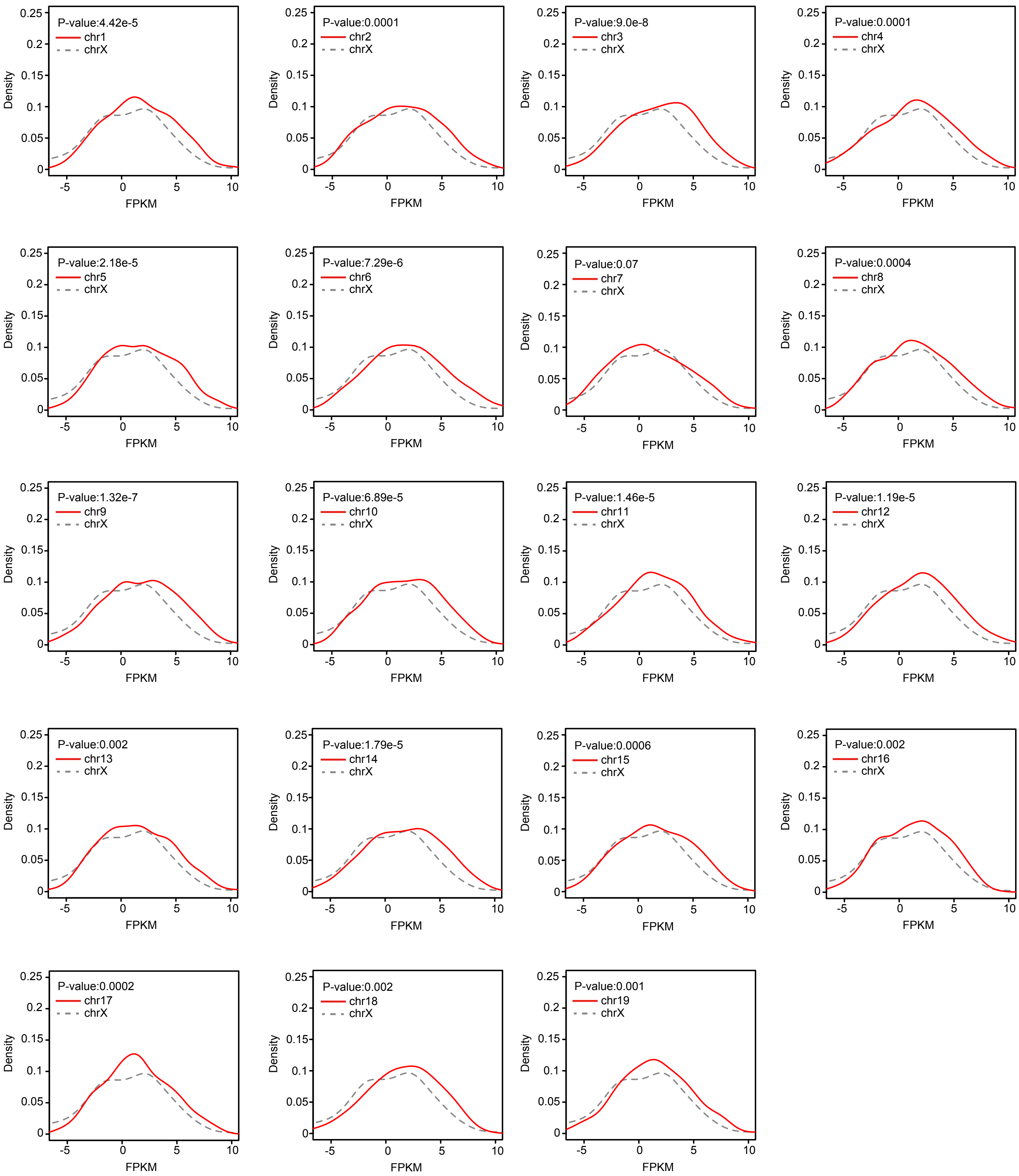

Supplementary Fig. 7

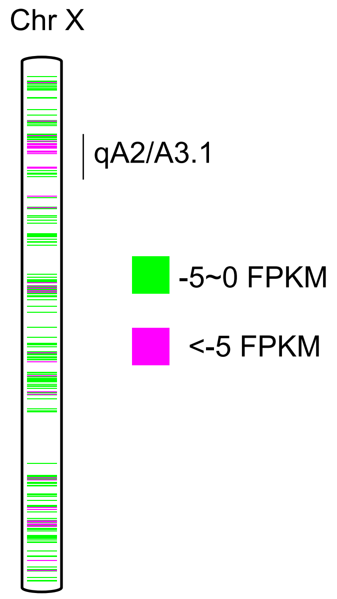

Supplementary Fig. 8

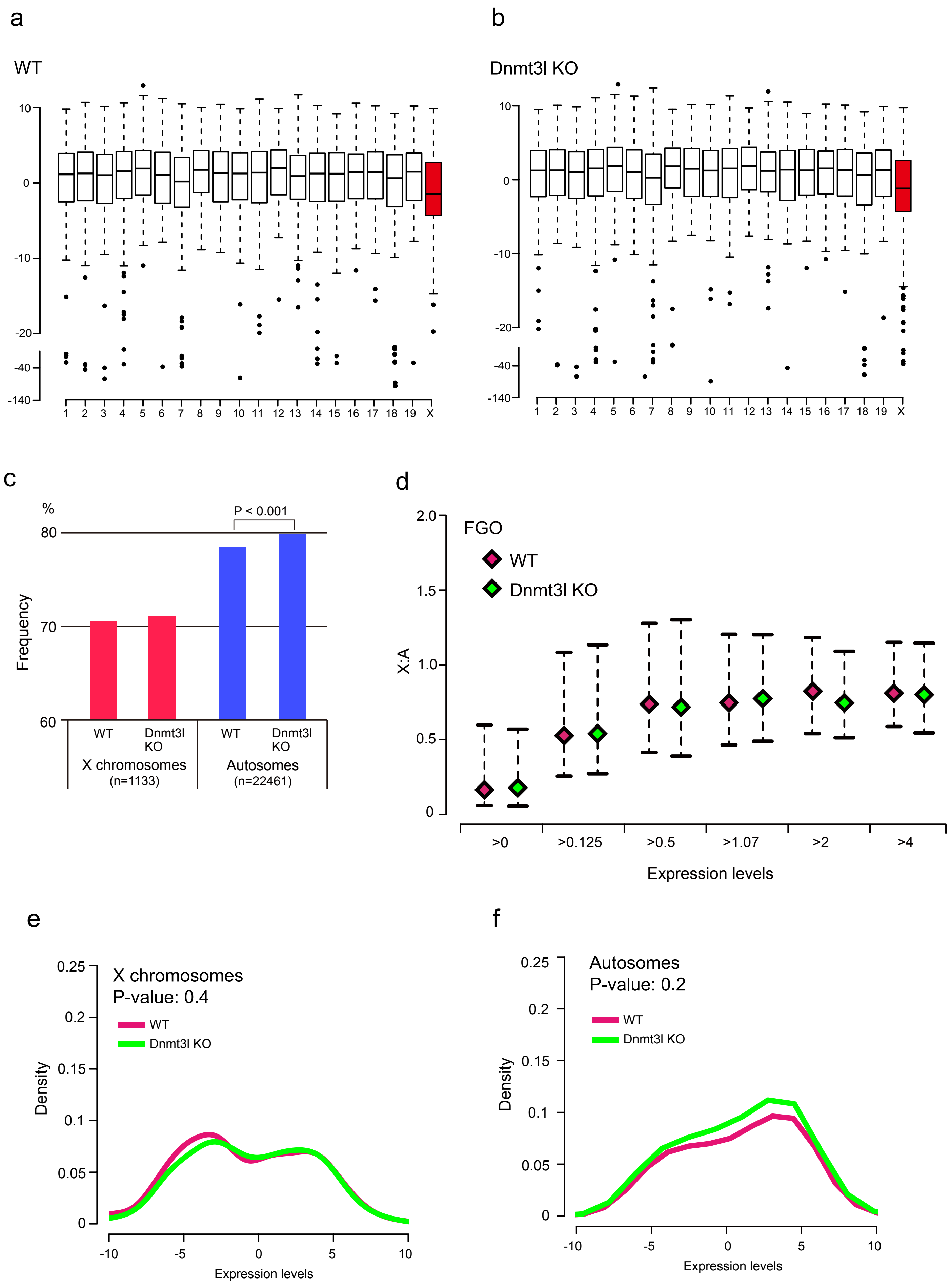

Supplementary Fig. 9

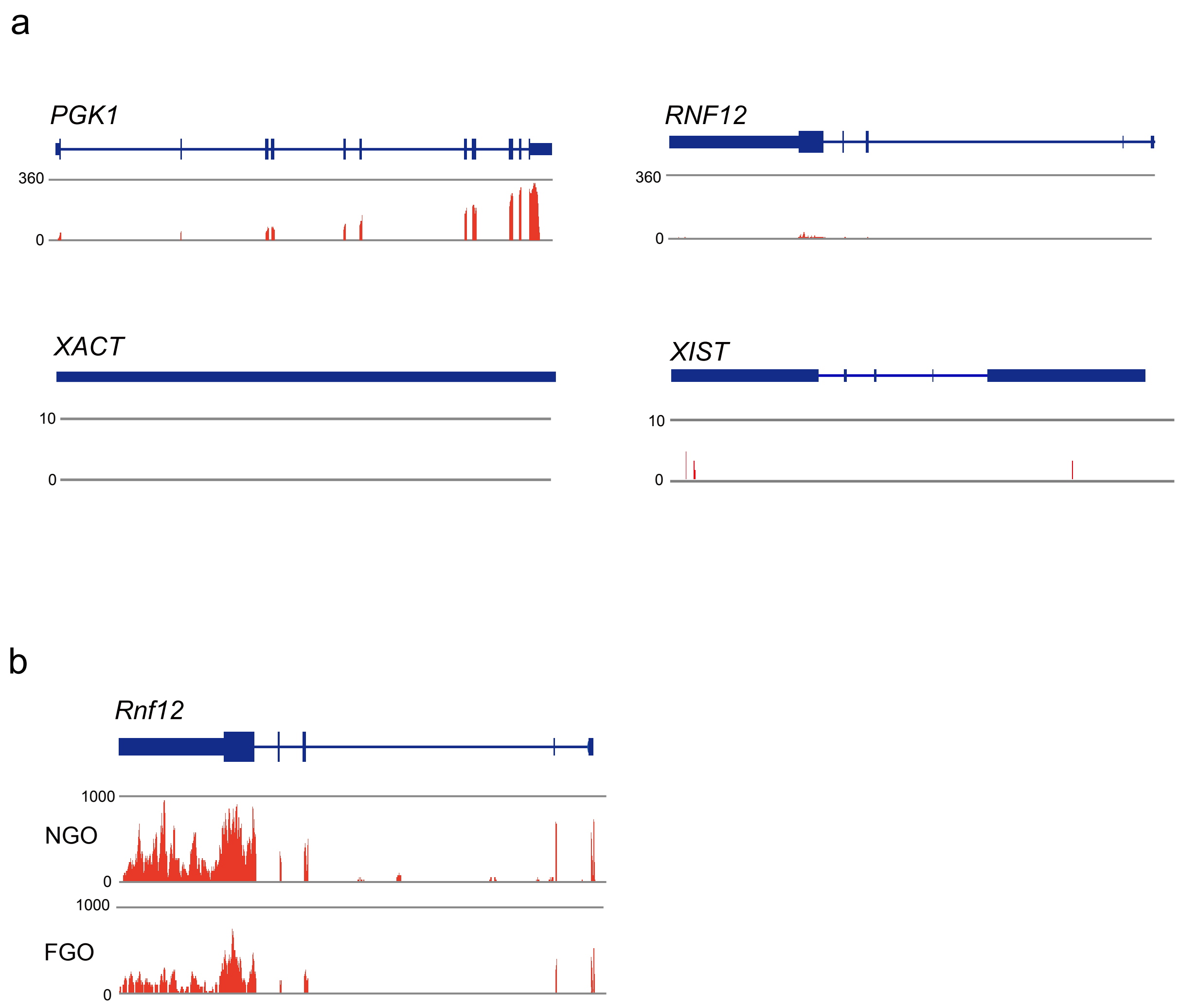

Supplementary Table. 1

Up in NGO					
**Gene Symbol**	**Chr**	**GV ave**	**ng ave**	**fold changes**	**t-test**
Trap1a	chrX	0.63654	12.0368	-11.400	0.00241
Mageb4	chrX	2.43694	13.129	-10.692	0.00693
Crabp1	chr9	0.30321	10.5766	-10.273	1.7E-05
Lrch4	chr5	0	10.1766	-10.177	0.00031
Il13ra1	chrX	0.89186	11.0294	-10.138	0.00622
Dlk1	chr12	0.30321	10.1071	-9.804	2E-05
2410016O06Rik	chr12	0	9.73428	-9.734	0.00094
Slc36a4	chr9	0.36354	10.071	-9.707	0.00066
Crebl2	chr6	1.22519	10.8784	-9.653	0.01553
Xpot	chr10	3.12218	12.7004	-9.578	0.02564
Scn5a	chr9	0.55852	10.1012	-9.543	0.00293
Tex13	chrX	0.72029	10.2447	-9.524	0.00031
Alg6	chr4	0.88508	10.3978	-9.513	0.00608
Urgcp	chr11	0	9.46358	-9.464	0.00043
Podxl	chr6	0.69009	10.1334	-9.443	0.00398
Lrrc3	chr10	0	9.41381	-9.414	0.00083
4930447C04Rik	chr12	0	9.41168	-9.412	0.00022
Gucy2f	chrX	0	9.40303	-9.403	0.00137
Rorc	chr3	1.24861	10.6499	-9.401	0.0037
Gm5128	chrX	0	9.39872	-9.399	5.7E-05
Taf7l	chrX	0	9.38215	-9.382	0.00081
Sohlh1	chr2	4.31014	13.6733	-9.363	0.00799
Cln5	chr14	1.07718	10.3764	-9.299	0.01154
Alg5	chr3	1.4134	10.5348	-9.121	0.02187
Psat1	chr19	2.82094	11.8831	-9.062	0.00922
Gcnt1	chr19	1.36354	10.3248	-8.961	0.0211
Nop16	chr13	1.81311	10.7738	-8.961	0.02289
Tab1	chr15	0.66674	9.57166	-8.905	0.00016
Nup62cl	chrX	0.30321	9.20145	-8.898	0.00013
Irgq	chr7	0.89186	9.77822	-8.886	0.00928
Btbd6	chr12	0.36354	9.1753	-8.812	0.00016
Trabd	chr15	1.66569	10.4691	-8.803	0.03397
Haghl	chr17	0.38696	9.1587	-8.772	5.6E-05
Hlf	chr11	1.35975	10.1228	-8.763	0.02264
Rbm46	chr3	1.91528	10.6587	-8.743	0.02311
Nxf2	chrX	0	8.72032	-8.720	0.00013
Hars2	chr18	1.00008	9.71641	-8.716	0.00075
Nckipsd	chr9	0.36354	9.05439	-8.691	7.5E-05
Itm2a	chrX	0.69009	9.3765	-8.686	0.00228
C77080	chr4	0	8.67683	-8.677	0.00067
Mrm1	chr11	0.36354	9.03183	-8.668	8.1E-05
Psma8	chr18	1.51668	10.1795	-8.663	0.02763
Ifngr1	chr10	0.9933	9.65586	-8.663	0.0035
Atg4d	chr9	0.69687	9.29478	-8.598	0.00338
Slc13a4	chr6	0	8.53191	-8.532	0.00026
Ehhadh	chr16	0	8.50568	-8.506	0.00039
Ctsf	chr19	2.63859	11.0847	-8.446	0.02119
Eif5a2	chr3	4.0491	12.4689	-8.420	0.00234
Mum1l1	chrX	1.72029	10.1302	-8.410	0.01055
Dmrta2	chr4	0	8.40629	-8.406	8.5E-05
Ndrg1	chr15	2.36972	10.7667	-8.397	0.04885
H19	chr7	3.47012	11.8224	-8.352	0.0009
Kank3	chr17	2.57145	10.9207	-8.349	0.0069

Lacc1	chr14	1.5099	9.82542	-8.316	0.03086
1810030O07Rik	chrX	3.44072	11.7514	-8.311	0.04019
Col1a2	chr6	0.88508	9.16344	-8.278	0.0098
Fam109a	chr5	0.69687	8.95998	-8.263	0.00302
Mpdu1	chr11	1.65608	9.85063	-8.195	0.01566
Phf17	chr3	3.06842	11.2546	-8.186	0.03121
Tnfrsf10b	chr14	1.19506	9.37098	-8.176	0.00324
Kdelc2	chr9	1.76727	9.90778	-8.141	0.0096
Zfp747	chr7	1.35676	9.43661	-8.080	0.02697
4933406J08Rik	chr2	0.30321	8.37222	-8.069	4.3E-05
Smtnl2	chr11	0.36354	8.41573	-8.052	0.00075
Rsad1	chr11	0.89141	8.89693	-8.006	0.00935
Tmem177	chr1	0.36354	8.34082	-7.977	0.00018
Zc3hav1l	chr6	3.26158	11.2245	-7.963	0.01416
Slc25a42	chr8	1.00008	8.92747	-7.927	0.00121
Lysmd1	chr3	0.35676	8.28173	-7.925	0.00138
Piwil4	chr9	0.69009	8.60846	-7.918	0.00338
Ppp1r16a	chr15	1.42018	9.32856	-7.908	0.0299
Zfp783	chr6	1.33708	9.2399	-7.903	0.02526
Irs2	chr8	1.88515	9.73988	-7.855	0.00117
Fzd5	chr1	2.82155	10.6729	-7.851	0.04205
Abca1	chr4	4.75426	12.5772	-7.823	0.02128
Armc6	chr8	0.9933	8.80372	-7.810	0.00325
Col6a2	chr10	1.46407	9.26614	-7.802	0.03038
Zscan22	chr7	0.35676	8.14481	-7.788	0.00147
Id1	chr2	2.2147	9.99666	-7.782	0.01965
Ikbkg	chrX	4.86697	12.6469	-7.780	0.00504
Fggy	chr4	1.4134	9.18389	-7.770	0.03116
Hexb	chr13	3.34926	11.0913	-7.742	0.00235
Vps18	chr2	2.4436	10.1814	-7.738	0.02879
D7Ertd715e	chr7	0.72029	8.44558	-7.725	0.00511
Unc45a	chr7	2.85412	10.5675	-7.713	0.00018
Eda2r	chrX	1.88515	9.57528	-7.690	0.00138
Pramel1	chr4	1.95928	9.64409	-7.685	0.01113
Igdcc4	chr9	5.10632	12.7576	-7.651	6E-05
Cgrrf1	chr14	1.56319	9.21349	-7.650	0.01066
Tyro3	chr2	3.22745	10.8723	-7.645	0.01716
Adrbk2	chr5	2.13081	9.76889	-7.638	0.00189
Aifm3	chr16	4.4532	12.0784	-7.625	0.00792
Atxn7l3	chr11	2.95203	10.5767	-7.625	0.00156
Pkhd1l1	chr15	1.5099	9.10213	-7.592	0.0359
Zmat1	chrX	2.08272	9.64837	-7.566	0.0238
Wipf3	chr6	3.55928	11.1207	-7.561	0.00405
Tcp11l2	chr10	2.15322	9.71455	-7.561	0.02779
2610008E11Rik	chr10	3.09006	10.6503	-7.560	0.02766
Abcg2	chr6	2.72338	10.2805	-7.557	0.03222
1600012H06Rik	chr17	2.0235	9.56327	-7.540	0.00145
Zkscan3	chr13	3.31362	10.8518	-7.538	0.0212
Entpd7	chr19	2.5284	10.0467	-7.518	0.02445
Stk17b	chr1	2.21849	9.73268	-7.514	0.00427
Fam19a1	chr6	2.29559	9.80795	-7.512	0.01982
Nr2f6	chr8	1.39005	8.88073	-7.491	0.00689
Retsat	chr6	1.59702	9.0848	-7.488	0.03196
Col4a5	chrX	1.21841	8.66268	-7.444	0.0235
D3Ertd254e	chr3	1.67695	9.11577	-7.439	0.00616
Slc27a2	chr2	4.38599	11.8242	-7.438	0.00713
Atp8b2	chr3	2.88515	10.3085	-7.423	1.9E-06

Col1a1	chr11	1.92908	9.34511	-7.416	0.0168
Osgin2	chr4	1.53918	8.90337	-7.364	0.03786
Zfp202	chr9	2.05827	9.41465	-7.356	0.02673
Hormad1	chr3	1.89344	9.24343	-7.350	0.03133
Tmub2	chr11	2.6016	9.93874	-7.337	0.00813
Frs3	chr17	2.29559	9.6326	-7.337	0.03362
Sccpdh	chr1	2.95377	10.2754	-7.322	0.04574
Gorasp1	chr9	1.5284	8.83052	-7.302	0.01388
Dolpp1	chr2	3.10614	10.4029	-7.297	0.02798
Cbx4	chr11	3.61068	10.8987	-7.288	0.03534
Arl4c	chr1	1.57233	8.85952	-7.287	0.03991
Fhl1	chrX	2.19506	9.45817	-7.263	0.02669
Ilvbl	chr10	1.18335	8.4458	-7.262	0.02108
Micall1	chr15	1.93586	9.18524	-7.249	0.03363
Tmsb4x	chrX	2.25698	9.50365	-7.247	0.02743
Plcl2	chr17	2.85297	10.0936	-7.241	0.01254
Fn3krp	chr11	3.06659	10.3031	-7.236	0.0171
Gm20300	chr10	2.00008	9.2065	-7.206	0.01842
Zfp871	chr17	5.98853	13.1746	-7.186	7.3E-05
Zfp622	chr15	4.75836	11.9442	-7.186	0.04024
Zbtb38	chr9	3.97408	11.117	-7.143	0.01947
Camk2b	chr11	2.43394	9.54304	-7.109	0.02845
Xbp1	chr11	3.96552	11.0635	-7.098	0.01032
Maged2	chrX	3.17573	10.2729	-7.097	0.04366
Spryd4	chr10	1.42018	8.51562	-7.095	0.03381
Pde7a	chr3	2.73326	9.82399	-7.091	0.01144
Acss2	chr2	2.4436	9.53392	-7.090	0.0338
6720401G13Rik	chrX	4.65913	11.7413	-7.082	0.00123
Myo15	chr11	3.56662	10.6269	-7.060	0.00942
Chchd4	chr6	4.03095	11.0891	-7.058	0.00239
Dpp4	chr2	2.29559	9.33601	-7.040	0.02136
Tef	chr15	5.11243	12.148	-7.036	0.0069
Sgpl1	chr10	4.78644	11.7938	-7.007	0.00285
Piwil2	chr14	8.56741	15.5536	-6.986	9.1E-07
Thbs3	chr3	1.38696	8.3389	-6.952	0.01482
Ybey	chr10	1.79982	8.74243	-6.943	0.00112
Prmt6	chr3	1.85495	8.79192	-6.937	0.01665
Qrsl1	chr10	3.70222	10.63	-6.928	0.04026
Acad8	chr9	1.9933	8.91223	-6.919	0.01973
Klhl5	chr5	3.89995	10.8173	-6.917	0.01223
Lrsam1	chr2	2.76727	9.68457	-6.917	0.03195
Plxna3	chrX	1.77693	8.68826	-6.911	0.02988
Fam195b	chr11	2.51773	9.42106	-6.903	0.02259
Slc9a6	chrX	6.82394	13.7136	-6.890	0.00201
Klc3	chr7	1.90022	8.77846	-6.878	0.03071
Gemin4	chr11	2.8461	9.68163	-6.836	0.04175
Bloc1s6	chr2	5.28504	12.1156	-6.831	0.0021
Tsr2	chrX	3.92825	10.7432	-6.815	0.00137
Olfr856-ps1	chr9	1.59575	8.40805	-6.812	0.01216
Zgpat	chr2	2.94693	9.74151	-6.795	0.00305
Smoc1	chr12	1.32663	8.10802	-6.781	0.01721
Rasd1	chr11	2.74681	9.48885	-6.742	0.03081
Tmem64	chr4	5.71073	12.4352	-6.724	0.02511
Adam19	chr11	4.3192	11.0361	-6.717	0.00083
Isg20l2	chr3	2.88515	9.59333	-6.708	0.00018
Zfp711	chrX	3.13654	9.81179	-6.675	0.04965
Cyb5r1	chr1	1.91473	8.58364	-6.669	0.01981

Kbtbd2	chr6	4.91194	11.5686	-6.657	0.00807
Sdr39u1	chr14	2.91837	9.55812	-6.640	0.04616
Dlg3	chrX	3.4876	10.1231	-6.635	0.0411
Tatdn1	chr15	2.85183	9.47922	-6.627	0.0447
Khnyn	chr14	2.55091	9.16966	-6.619	0.04121
Zpbp	chr11	2.13081	8.7428	-6.612	0.00355
Hspbp1	chr7	2.65608	9.25886	-6.603	0.03689
Sh3gl3	chr7	2.4542	9.04885	-6.595	0.03245
Bri3bp	chr5	2.93491	9.52825	-6.593	0.047
Col5a1	chr2	1.85495	8.44812	-6.593	0.01208
Hes1	chr16	1.72338	8.30739	-6.584	0.03522
Eid2	chr7	2.74681	9.32596	-6.579	0.01362
Epcam	chr17	5.87758	12.4535	-6.576	0.00015
5830433M19Rik	chr4	3.13081	9.70353	-6.573	0.00063
Kdm4c	chr4	3.69762	10.2621	-6.564	0.03236
Tbc1d24	chr17	3.23328	9.78026	-6.547	0.00416
Zfp292	chr4	6.46217	12.985	-6.523	9.8E-07
Fignl2	chr15	2.11027	8.59289	-6.483	0.03764
Cct6a	chr5	6.93243	13.4113	-6.479	0.00258
Slc35e1	chr8	5.07447	11.5479	-6.473	0.00226
Dner	chr1	3.96143	10.43	-6.469	0.00513
Zfp42	chr8	3.49046	9.95723	-6.467	0.04258
Churc1	chr12	3.43462	9.89533	-6.461	0.00139
Tspan7	chrX	3.91076	10.3707	-6.460	0.04534
Pin1	chr9	3.2284	9.6747	-6.446	0.02582
Smcr7	chr11	3.52078	9.95876	-6.438	0.0343
Kpna1	chr16	2.9678	9.40172	-6.434	0.02624
Gnl3l	chrX	6.24007	12.6656	-6.426	0.00324
Rnmtl1	chr11	2.59031	9.0107	-6.420	0.02797
E330020D12Rik	chr1	6.67803	13.075	-6.397	0.01262
Bend4	chr5	4.28395	10.6713	-6.387	0.00965
Mdn1	chr4	9.07804	15.458	-6.380	0.00022
Dhcr24	chr4	5.76092	12.0953	-6.334	0.01707
Map3k3	chr11	3.38244	9.69073	-6.308	0.01171
Sfmbt2	chr2	3.96143	10.2594	-6.298	0.01684
Fam73a	chr3	3.46414	9.75479	-6.291	0.01568
Rcn1	chr2	2.81989	9.07817	-6.258	0.03557
Jub	chr14	3.65913	9.91514	-6.256	0.01078
Slc35d1	chr4	2.55091	8.8047	-6.254	0.0374
Kdm5b	chr1	7.34219	13.5784	-6.236	0.00317
AI462493	chr19	3.06108	9.28105	-6.220	0.04733
Kpna4	chr3	5.66287	11.8659	-6.203	0.0044
Etv3	chr3	4.47522	10.6667	-6.191	0.00036
9430008C03Rik	chr2	2.4876	8.67153	-6.184	0.01637
Bhlhb9	chrX	4.91881	11.0843	-6.165	0.0025
Glis2	chr16	2.99246	9.15459	-6.162	0.00351
Yy2	chrX	2.25869	8.42041	-6.162	0.04793
Zfp446	chr7	3.13081	9.28772	-6.157	0.00413
Hspg2	chr4	4.80345	10.9526	-6.149	0.00458
Klrg2	chr6	2.10739	8.25456	-6.147	0.02784
Mbnl3	chrX	2.66742	8.8042	-6.137	0.04471
Sec61a2	chr2	5.16267	11.2886	-6.126	0.00629
Lias	chr5	5.3023	11.4205	-6.118	0.00765
Eif4ebp1	chr8	5.31695	11.4284	-6.111	0.0034
Cd24a	chr10	3.17664	9.27637	-6.100	0.02185
Maged1	chrX	8.33133	14.4204	-6.089	0.00417
Elac1	chr18	4.24409	10.3146	-6.070	0.00324

Ppp2cb	chr8	5.4716	11.5281	-6.057	2.7E-05
H47	chr7	3.79747	9.84592	-6.048	0.00416
Mtap7d2	chrX	7.66396	13.7089	-6.045	1.4E-06
Zfp105	chr9	4.32979	10.373	-6.043	0.01208
Edc3	chr9	5.09817	11.126	-6.028	0.00051
Gstm4	chr3	3.42144	9.44744	-6.026	0.03797
Ets1	chr9	2.54803	8.5634	-6.015	0.04066
Gabpa	chr16	6.08053	12.0889	-6.008	0.0003
Amt	chr9	2.68259	8.68417	-6.002	0.02552
Tmem41a	chr16	3.55182	9.53005	-5.978	0.00279
Prtg	chr9	4.39126	10.3692	-5.978	0.00475
Col18a1	chr10	2.62893	8.57297	-5.944	0.04696
Pepd	chr7	4.86612	10.7938	-5.928	0.0053
Zfp266	chr9	4.72108	10.6463	-5.925	0.00165
Slc39a1	chr3	5.60919	11.5303	-5.921	0.00913
Marcksl1	chr4	4.61509	10.5313	-5.916	0.03313
Syce1	chr7	6.92809	12.8219	-5.894	0.00075
Prdm15	chr16	4.41347	10.3015	-5.888	0.01324
D030056L22Rik	chr19	6.00851	11.8725	-5.864	0.00585
Tsen15	chr1	3.63866	9.4995	-5.861	0.02383
Adi1	chr12	5.4373	11.2971	-5.860	0.00262
Wdr6	chr9	4.7036	10.5627	-5.859	0.0083
Pofut1	chr2	3.97408	9.82413	-5.850	0.00025
Rab28	chr5	4.29502	10.1379	-5.843	0.01026
Ndufaf1	chr2	3.63835	9.46579	-5.827	0.00297
Gm962	chr19	3.7853	9.60739	-5.822	0.01667
Bbx	chr16	9.66151	15.4673	-5.806	3.6E-06
4930470H14Rik	chr17	3.74881	9.55093	-5.802	0.0284
Suv39h1	chrX	5.71744	11.5159	-5.798	0.00426
Klhl17	chr4	3.82804	9.62617	-5.798	0.03381
Zfp213	chr17	4.38775	10.1798	-5.792	0.00024
Trim25	chr11	5.04724	10.8325	-5.785	0.00123
Uqcrc1	chr9	7.23339	13.0051	-5.772	0.00151
Vhl	chr6	5.53776	11.3008	-5.763	0.01286
Rnf11	chr4	7.19557	12.956	-5.760	5.1E-05
Lemd3	chr10	4.40207	10.1122	-5.710	1.6E-05
Rap1gap	chr4	5.16907	10.8749	-5.706	0.01769
Wdr4	chr17	4.07257	9.77771	-5.705	0.00011
Dusp9	chrX	5.80308	11.5079	-5.705	0.00013
Lemd2	chr17	4.88858	10.5856	-5.697	0.00614
Zdhhc9	chrX	5.09006	10.7714	-5.681	0.00016
Kif21a	chr15	7.04771	12.7246	-5.677	0.00148
C1d	chr11	5.906	11.5772	-5.671	0.00473
Nell2	chr15	3.31362	8.97439	-5.661	0.03249
Zfp422	chr6	5.12514	10.78	-5.655	0.00152
Cog2	chr8	5.34277	10.9828	-5.640	0.01112
Fam76a	chr4	4.92453	10.5359	-5.611	0.00158
Gjc1	chr11	4.21282	9.82343	-5.611	0.02116
Cnnm3	chr1	4.77929	10.3722	-5.593	0.00801
Snx10	chr6	3.63446	9.21493	-5.580	0.00099
D830031N03Rik	chr4	8.42832	14.0004	-5.572	0.00058
Malt1	chr18	6.2205	11.783	-5.563	0.00332
Pitpnb	chr5	5.6539	11.2059	-5.552	0.00921
Clp1	chr2	4.35636	9.90626	-5.550	0.03014
Phf16	chrX	5.93561	11.4818	-5.546	0.00542
Ppp2r3a	chr9	6.98676	12.5307	-5.544	0.0004
Cntnap5a	chr1	4.36889	9.90655	-5.538	0.0097

Cct6a	chr5	3.82193	9.35661	-5.535	0.00054
Fam136a	chr6	4.96372	10.4978	-5.534	0.01256
Mgat2	chr12	4.46676	9.99994	-5.533	0.00032
Cpt1a	chr19	5.19045	10.7221	-5.532	0.00192
Sp140	chr1	7.02913	12.5581	-5.529	0.00113
Fam134b	chr15	6.07805	11.6048	-5.527	0.00143
Tomm5	chr4	2.88515	8.40766	-5.523	0.03199
Syap1	chrX	6.58069	12.1006	-5.520	0.00499
Grk6	chr13	4.29684	9.80667	-5.510	0.00421
Kifap3	chr1	7.84801	13.3509	-5.503	2.6E-06
Rbm10	chrX	6.37049	11.8722	-5.502	0.00342
Psmg2	chr18	4.74681	10.2481	-5.501	0.00036
E2f3	chr13	3.74681	9.24694	-5.500	0.00436
Slc35e3	chr10	3.96143	9.45339	-5.492	0.01641
Ints7	chr1	6.14966	11.6389	-5.489	0.0056
Chtf8	chr8	5.57977	11.0511	-5.471	6.8E-05
Polr3g	chr13	7.83093	13.2905	-5.460	0.00015
Zc3hc1	chr6	4.21091	9.66122	-5.450	0.0052
Maf1	chr15	6.25246	11.7005	-5.448	1.2E-05
Mettl13	chr1	5.40448	10.8523	-5.448	0.00161
Zfp507	chr7	6.84125	12.2831	-5.442	0.00011
Ccdc43	chr11	4.13678	9.57652	-5.440	0.00032
4932411L15	chr1	4.09516	9.5342	-5.439	0.01284
Mov10l1	chr15	9.44468	14.8677	-5.423	1.1E-05
Gm5113	chr7	3.67876	9.08502	-5.406	0.03207
Hdac11	chr6	5.64255	11.0374	-5.395	9.5E-05
Rbms1	chr2	5.64612	11.0306	-5.384	0.0388
BC003965	chr17	5.29073	10.6706	-5.380	0.0017
Cad	chr5	7.95039	13.3283	-5.378	0.00059
Sycp3	chr10	8.52459	13.8939	-5.369	4.8E-05
Ak3	chr19	4.37841	9.7154	-5.337	0.02455
Zfp668	chr7	4.95807	10.2914	-5.333	0.00679
Ogfr	chr2	5.08014	10.4076	-5.328	0.00928
Adck3	chr1	7.16286	12.4884	-5.326	0.00098
Cacybp	chr1	7.14089	12.4541	-5.313	1.5E-05
Jrk	chr15	3.96143	9.2655	-5.304	0.00045
Zfx	chrX	6.96591	12.2656	-5.300	0.02286
Zfp281	chr1	6.86422	12.1525	-5.288	0.00039
Rab6b	chr9	6.14281	11.428	-5.285	0.00068
Sord	chr2	4.73923	10.0136	-5.274	0.01802
Tmem115	chr9	3.4876	8.75956	-5.272	0.04094
Fam49b	chr15	6.08574	11.3431	-5.257	0.00014
Ptgr2	chr12	6.66246	11.9187	-5.256	0.00243
LOC101056086	chr8	3.76675	9.02175	-5.255	0.0024
Zfp488	chr14	3.71817	8.96694	-5.249	0.00323
Ddah1	chr3	5.19342	10.4301	-5.237	0.00035
Snrpb	chr2	7.27135	12.4968	-5.225	2.7E-05
Hexim1	chr11	3.3258	8.5453	-5.220	0.00043
Slc27a4	chr2	4.94647	10.1586	-5.212	0.00047
Mpv17	chr5	3.53588	8.74419	-5.208	0.01318
Mfge8	chr7	4.15427	9.35621	-5.202	0.03896
Slc39a11	chr11	5.4518	10.6516	-5.200	0.0039
Atxn10	chr15	7.27497	12.4743	-5.199	5.8E-06
Spcs3	chr8	7.67036	12.8639	-5.194	3.3E-05
Ttyh2	chr11	6.41875	11.5936	-5.175	0.00811
Hacl1	chr14	3.92825	9.09639	-5.168	0.01698
Prr12	chr7	6.61848	11.7847	-5.166	0.00555

Tktl1	chrX	6.37174	11.5366	-5.165	0.00097
Smc1b	chr15	10.3752	15.5236	-5.148	5.4E-05
Frat2	chr19	5.49691	10.6449	-5.148	0.00113
2310046O06Rik	chr9	5.9474	11.0944	-5.147	0.0005
Pnrc2	chr4	5.56327	10.7088	-5.146	0.01356
Hdac5	chr11	4.60511	9.74717	-5.142	0.00115
Osbpl6	chr2	7.43696	12.5695	-5.133	0.00016
Tcf4	chr18	7.30494	12.433	-5.128	0.00226
Tbcel	chr9	6.23001	11.3529	-5.123	0.00011
Zfp768	chr7	5.75401	10.8736	-5.120	0.00607
Tmem18	chr12	5.37163	10.4867	-5.115	0.0005
Nanp	chr2	4.4876	9.60045	-5.113	0.01827
Iars2	chr1	7.7825	12.8822	-5.100	2E-06
Rpl10l	chr12	5.71365	10.8007	-5.087	0.00015
Nek9	chr12	7.6611	12.7473	-5.086	0.00344
Tbl1x	chrX	5.46264	10.5479	-5.085	0.00026
Pam	chr1	4.786	9.84253	-5.057	0.00287
Tubgcp6	chr15	5.09857	10.1546	-5.056	0.00786
Cbx3	chr6	7.49693	12.5394	-5.042	0.01042
D1Pas1	chr1	3.92595	8.96621	-5.040	0.03243
Impdh2	chr9	7.68123	12.7201	-5.039	0.0038
Esyt3	chr9	5.52676	10.5562	-5.029	0.0072
Aldh16a1	chr7	5.76644	10.791	-5.025	0.02565
Ripk4	chr16	5.54757	10.5718	-5.024	6.7E-05
Tusc2	chr9	5.30741	10.3226	-5.015	0.02167
4932435O22Rik	chr11	4.65687	9.66458	-5.008	0.00553
Sycp1	chr3	8.55312	13.5523	-4.999	8E-06
Klf8	chrX	3.71577	8.71047	-4.995	0.0003
Bahcc1	chr11	7.89926	12.882	-4.983	0.00037
Tbc1d12	chr19	4.52378	9.49855	-4.975	0.00398
Prdx4	chrX	6.57659	11.5503	-4.974	0.00107
Fbln1	chr15	5.07257	10.0327	-4.960	0.00031
1700052N19Rik	chr10	4.87609	9.82466	-4.949	0.01402
Mark1	chr1	5.53639	10.4831	-4.947	0.00046
Ift27	chr15	7.44354	12.3812	-4.938	0.00044
Tmx2	chr2	5.74806	10.6817	-4.934	0.00047
Epm2aip1	chr9	4.78298	9.71416	-4.931	0.0097
Mtmr1	chrX	4.95511	9.88176	-4.927	0.0004
Fam173a	chr17	4.55696	9.48191	-4.925	0.00573
Chmp6	chr11	4.83182	9.75372	-4.922	0.00566
Fam171b	chr2	4.972	9.88448	-4.912	0.00191
Ormdl3	chr11	5.02561	9.93788	-4.912	0.00049
Gpn3	chr5	7.50162	12.411	-4.909	0.00297
Dpp8	chr9	6.97357	11.8774	-4.904	0.00569
Rpl9	chr5	8.82595	13.726	-4.900	5.7E-07
Haus2	chr2	5.23586	10.1269	-4.891	0.01711
Smarcd1	chr15	6.07883	10.9591	-4.880	0.00048
Spns1	chr7	6.21358	11.0846	-4.871	0.00294
Snapc2	chr8	4.65802	9.5258	-4.868	0.00014
Akirin1	chr4	6.60599	11.467	-4.861	0.01198
Bcl9l	chr9	4.53913	9.39924	-4.860	0.02626
Slc45a4	chr15	5.64686	10.5052	-4.858	0.00915
Traf6	chr2	5.23656	10.0925	-4.856	0.01387
Slc38a1	chr15	7.60138	12.4554	-4.854	0.00016
Ngfrap1	chrX	4.96046	9.8133	-4.853	0.0057
Rufy3	chr5	6.58385	11.4318	-4.848	0.00176
Zfp395	chr14	6.18658	11.03	-4.843	0.00128

Ado	chr10	6.72862	11.5663	-4.838	1.1E-05
Rnf149	chr1	6.45841	11.2928	-4.834	0.00097
Ptch1	chr13	6.31417	11.1437	-4.830	0.00541
Nalcn	chr14	6.00067	10.8299	-4.829	0.00255
Rprd1b	chr2	4.28914	9.11215	-4.823	0.01934
Mll1	chr9	6.21926	11.0326	-4.813	0.00654
Gsr	chr8	8.18178	12.9896	-4.808	0.00426
Thtpa	chr14	3.95062	8.75782	-4.807	0.03103
Dck	chr5	5.96859	10.746	-4.777	0.0019
8030474K03Rik	chrX	4.5261	9.30105	-4.775	0.02591
Zfp26	chr9	8.05699	12.8292	-4.772	0.00143
Kdm5a	chr6	8.46509	13.2315	-4.766	0.00159
9930021J03Rik	chr19	7.74698	12.511	-4.764	1.6E-05
Gtf2i	chr5	7.16847	11.9284	-4.760	0.00405
Zfp367	chr13	5.12802	9.88528	-4.757	0.02581
Mak16	chr8	6.18132	10.9328	-4.752	0.00546
Nol11	chr11	5.62492	10.3749	-4.750	0.0062
Snhg11	chr2	4.45657	9.20371	-4.747	0.01361
Clmn	chr12	7.02397	11.7683	-4.744	0.00492
Gpkow	chrX	7.20225	11.9445	-4.742	0.0038
Wdr77	chr3	5.83275	10.575	-4.742	0.00435
A830080D01Rik	chrX	6.70736	11.4443	-4.737	0.00072
Sepp1	chr15	6.30073	11.034	-4.733	0.00079
Dysf	chr6	6.83627	11.5678	-4.732	0.00153
Ctsl	chr13	8.89577	13.6272	-4.731	1.7E-07
Slc12a4	chr8	5.43908	10.1649	-4.726	0.00027
Tmx4	chr2	7.30724	12.0192	-4.712	0.00164
Gpr143	chrX	6.25027	10.9533	-4.703	0.00689
Wdr45l	chr11	6.77224	11.4665	-4.694	0.00323
Rpusd2	chr2	6.8298	11.5203	-4.690	2.8E-06
Erlin2	chr8	7.93058	12.6204	-4.690	7.2E-05
Ddx6	chr9	10.7309	15.4123	-4.681	6.5E-05
Zfp36l2	chr17	8.46319	13.1402	-4.677	4.3E-05
Gigyf1	chr5	8.29197	12.9685	-4.677	0.00109
Adnp	chr2	7.75554	12.4235	-4.668	5.3E-06
Oxsr1	chr9	7.46217	12.0984	-4.636	0.00629
Yeats2	chr16	6.682	11.3109	-4.629	0.01891
Prkag2	chr5	7.32407	11.9525	-4.628	0.00152
Cdc5l	chr17	7.75815	12.3861	-4.628	9.7E-06
Arl14epl	chr18	8.17796	12.8033	-4.625	0.00225
Ccdc46	chr11	6.29601	10.9191	-4.623	0.00054
Pdxk	chr10	3.99102	8.61324	-4.622	0.00967
Zdbf2	chr1	7.25821	11.88	-4.622	0.01619
2510039O18Rik	chr4	7.06069	11.679	-4.618	0.00777
Prps1	chrX	7.30938	11.9255	-4.616	9.2E-05
Hemk1	chr9	4.01925	8.62597	-4.607	0.00186
Etfdh	chr3	6.1871	10.791	-4.604	0.0022
Hsd17b10	chrX	7.72397	12.3229	-4.599	0.00592
Tia1	chr6	4.93708	9.52898	-4.592	0.02771
Prkacb	chr3	9.36567	13.9562	-4.591	6.5E-05
Kcnt1	chr2	4.71577	9.2985	-4.583	0.00016
Ring1	chr17	5.25772	9.83939	-4.582	0.00012
Camk1g	chr1	4.77596	9.34723	-4.571	0.01361
Crim1	chr17	4.28176	8.84805	-4.566	0.0472
Gpat2	chr2	7.40043	11.9631	-4.563	2.4E-05
Slc27a1	chr8	5.65985	10.2222	-4.562	0.01604
Klhl26	chr8	3.91076	8.47303	-4.562	0.03701

Tmeff1	chr4	4.50724	9.06699	-4.560	0.01238
Agphd1	chr9	7.75277	12.3122	-4.559	2E-06
Id2	chr12	4.73326	9.29241	-4.559	7.1E-05
Cd47	chr16	4.63446	9.18928	-4.555	0.04686
Hist1h2ao	chr13	4.90836	9.45106	-4.543	0.00034
Scrib	chr15	6.64399	11.1829	-4.539	0.00241
Kifc2	chr15	3.84057	8.37691	-4.536	0.01104
Ap3b2	chr7	8.422	12.9564	-4.534	0.00095
Sppl3	chr5	6.63394	11.15	-4.516	0.01491
Sh2b1	chr7	5.95265	10.461	-4.508	0.00038
Ckap4	chr10	5.50552	10.009	-4.503	0.00064
Trim45	chr3	5.56934	10.0521	-4.483	0.01757
Neb	chr2	11.4189	15.9013	-4.482	0.0006
Fyttd1	chr16	7.61763	12.0873	-4.470	3.7E-06
Glt25d1	chr8	5.75909	10.2231	-4.464	0.00352
Zfp187	chr13	6.10143	10.5574	-4.456	0.00112
Rrs1	chr1	6.15846	10.6124	-4.454	0.00262
Vamp2	chr11	5.46676	9.91645	-4.450	0.00105
Hist1h2ap	chr13	4.99635	9.4376	-4.441	8.7E-05
Cd93	chr2	4.58274	9.02196	-4.439	5.4E-05
Rc3h2	chr2	7.35902	11.798	-4.439	0.01088
Trp53bp2	chr1	6.15711	10.5828	-4.426	0.0029
Prosapip1	chr2	5.09369	9.51581	-4.422	0.00977
Timm22	chr11	5.25434	9.6732	-4.419	0.00397
Hsf2	chr10	6.7909	11.2079	-4.417	0.00621
Zfp59	chr7	4.80528	9.21476	-4.409	0.00016
Dr1	chr5	8.23635	12.6416	-4.405	0.00187
1700123O20Rik	chr14	5.21345	9.6179	-4.404	0.02347
Agrn	chr4	7.7775	12.1802	-4.403	0.00011
Emc1	chr4	8.27939	12.6795	-4.400	0.00065
Ahnak	chr19	6.48342	10.879	-4.396	0.01157
Zxdb	chrX	6.86678	11.2578	-4.391	0.00618
Gmppb	chr9	4.41159	8.80174	-4.390	0.02052
Mir17hg	chr14	4.60846	8.99711	-4.389	0.03327
Eid1	chr2	5.98489	10.3731	-4.388	0.00039
Stim1	chr7	6.38672	10.7531	-4.366	0.01059
Anxa11	chr14	6.86879	11.2348	-4.366	0.01043
Prrc2b	chr2	7.43511	11.7986	-4.364	2.1E-06
Nfia	chr4	7.11705	11.4759	-4.359	0.00011
Klhdc9	chr1	4.45657	8.80922	-4.353	0.00806
Grid2	chr6	5.47382	9.82161	-4.348	0.00247
Tmed8	chr12	6.02029	10.3663	-4.346	0.00319
Tecpr1	chr5	4.38344	8.72928	-4.346	0.03178
Arrdc4	chr7	5.17527	9.52111	-4.346	0.02858
Yars	chr4	5.0383	9.3822	-4.344	0.0004
2900097C17Rik	chr2	8.74812	13.0851	-4.337	1.7E-05
Mettl2	chr11	5.55843	9.89011	-4.332	0.00303
Pctp	chr11	6.07257	10.4037	-4.331	0.04584
Pex6	chr17	7.09103	11.4141	-4.323	0.00339
Zfyve27	chr19	7.01269	11.3313	-4.319	0.01231
Gba	chr3	5.33186	9.64919	-4.317	0.01649
Eif2c1	chr4	7.14754	11.4606	-4.313	0.00434
Ttf1	chr2	5.17953	9.4925	-4.313	0.04925
Zfp275	chrX	5.7191	10.0316	-4.313	0.00028
Prox1	chr1	6.99419	11.3015	-4.307	0.00327
Nmt2	chr2	8.99477	13.299	-4.304	0.00061
Mtm1	chrX	6.60701	10.9047	-4.298	0.00012

Tomm70a	chr16	8.41003	12.703	-4.293	0.00308
Gltscr2	chr7	8.4846	12.777	-4.292	0.00171
Ehmt2	chr17	7.56486	11.8365	-4.272	0.00063
Traf2	chr2	4.60971	8.88074	-4.271	0.00074
Stx3	chr19	5.79124	10.0606	-4.269	0.00311
Ptk7	chr17	5.01257	9.27811	-4.266	0.00076
Casd1	chr6	4.4876	8.74923	-4.262	0.0192
Pknox1	chr17	6.86697	11.1283	-4.261	0.0001
Casp7	chr19	4.49506	8.75339	-4.258	0.00116
Serinc5	chr13	9.18576	13.4301	-4.244	6.3E-05
Mlh3	chr12	7.12506	11.3674	-4.242	0.00036
Aar2	chr2	5.87254	10.1148	-4.242	0.00095
Tbl2	chr5	7.59603	11.8308	-4.235	4.6E-05
Gsto1	chr19	4.96674	9.19211	-4.225	0.04586
Mtx3	chr13	6.77061	10.9922	-4.222	0.00391
Slc39a13	chr2	5.00176	9.22122	-4.219	0.00394
Spag9	chr11	7.22601	11.443	-4.217	0.01705
Uaca	chr9	7.91523	12.1322	-4.217	1.2E-06
Eml3	chr19	6.75526	10.9716	-4.216	0.00627
Ip6k1	chr9	7.6298	11.8449	-4.215	0.00012
Hexa	chr9	5.69391	9.90763	-4.214	0.00553
Tbc1d17	chr7	5.64983	9.85945	-4.210	0.00044
Irf2bp2	chr8	8.98587	13.1936	-4.208	0.00229
Sox12	chr2	6.68438	10.8898	-4.205	0.00157
Klhdc8b	chr9	5.22594	9.42644	-4.201	0.03709
Syt9	chr7	6.6227	10.8221	-4.199	0.00917
Golga1	chr2	5.84271	10.0343	-4.192	0.00191
Sobp	chr10	6.08119	10.2569	-4.176	5.4E-05
Nck2	chr1	7.48712	11.6603	-4.173	0.00367
Syde2	chr3	6.64422	10.8161	-4.172	0.00087
Impad1	chr4	5.84908	10.0119	-4.163	0.00158
Cacna2d2	chr9	4.8326	8.98884	-4.156	0.01537
Gas2	chr7	5.41745	9.57069	-4.153	0.00045
Hdgfrp3	chr7	6.35633	10.5046	-4.148	0.00126
Nadkd1	chr15	8.87895	13.0271	-4.148	0.00036
Pfas	chr11	6.87823	11.0242	-4.146	0.0041
Gabarapl1	chr6	5.8241	9.96943	-4.145	0.0041
Sh3bp5l	chr11	5.71035	9.85004	-4.140	0.01044
Rps23	chr13	10.5479	14.6841	-4.136	0.00272
Nfic	chr10	6.82418	10.9598	-4.136	0.00254
Zfp386	chr12	5.78559	9.92042	-4.135	0.00986
Pcnp	chr16	8.3556	12.4869	-4.131	0.00036
Gnpda1	chr18	4.84806	8.97371	-4.126	0.02178
Plekhf2	chr4	6.47757	10.5976	-4.120	0.00015
Gm1141	chrX	4.52212	8.63877	-4.117	0.02042
Antxr1	chr6	8.59048	12.7047	-4.114	1.6E-05
Casp6	chr3	5.99032	10.1012	-4.111	0.003
Cuedc2	chr19	6.13815	10.2449	-4.107	0.00023
Gpsm1	chr2	5.2284	9.33196	-4.104	0.0099
Phactr2	chr10	7.50728	11.6108	-4.103	0.00157
Slc30a7	chr3	7.16573	11.2676	-4.102	0.00023
Foxred1	chr9	5.36889	9.46972	-4.101	0.00112
Cpsf7	chr19	7.63693	11.7369	-4.100	0.00105
D6Mm5e	chr6	4.71175	8.80429	-4.093	0.00998
Ppp1r12c	chr7	4.82094	8.9115	-4.091	0.00583
Cat	chr2	5.28258	9.37174	-4.089	0.00822
Syt14	chr1	6.20416	10.2921	-4.088	0.00585

Syncrip	chr9	8.51849	12.601	-4.082	1.4E-05
Lama3	chr18	6.73152	10.8077	-4.076	0.0097
Slc35f5	chr1	8.30791	12.3818	-4.074	0.00019
Rad23a	chr8	6.20564	10.2781	-4.072	8.2E-05
Ly6a	chr15	4.75426	8.82479	-4.071	0.00653
BC005537	chr13	9.4818	13.5513	-4.070	7.7E-05
Aifm2	chr10	6.08061	10.1458	-4.065	0.00959
1700019G17Rik	chr6	4.65451	8.71595	-4.061	0.00059
Prmt10	chr8	7.28747	11.3421	-4.055	0.00023
Nfat5	chr8	8.56487	12.6147	-4.050	0.00017
Dync1h1	chr12	11.703	15.7526	-4.050	0.00046
Ero1l	chr14	6.05035	10.0928	-4.042	0.01917
Baat1	chr5	5.75276	9.78791	-4.035	0.04407
Slc9a8	chr2	7.66342	11.6951	-4.032	0.00125
Klhl11	chr11	8.38035	12.4021	-4.022	3.3E-06
Stk16	chr1	5.77892	9.79314	-4.014	0.0064
Psd	chr19	4.6202	8.632	-4.012	0.00064
Gcsh	chr8	5.20225	9.21331	-4.011	0.02467
Tbcd	chr11	10.3468	14.3559	-4.009	5.2E-05
Ola1	chr2	8.51432	12.522	-4.008	3.7E-05
Tmem86a	chr7	6.24941	10.2548	-4.005	0.03946
Pmvk	chr3	6.23652	10.2404	-4.004	0.00555
Sdad1	chr5	10.0457	14.0446	-3.999	0.00044
Fkbp9	chr6	9.49543	13.4937	-3.998	0.00062
6820431F20Rik	chr8	5.13587	9.13281	-3.997	0.00068
Abca3	chr17	8.30471	12.2987	-3.994	0.00241
Guk1	chr11	5.71702	9.7	-3.983	0.00255
Mfsd7b	chr1	4.95156	8.93024	-3.979	0.00062
Osgep	chr14	6.65747	10.6345	-3.977	2.1E-05
St3gal2	chr8	5.81681	9.79307	-3.976	0.01349
Bcl2	chr1	5.27887	9.2547	-3.976	0.01389
Pmpca	chr2	7.84829	11.8211	-3.973	0.0017
Tmem205	chr9	4.81611	8.77289	-3.957	0.00757
Rab24	chr13	5.45197	9.40688	-3.955	0.00172
Psmd13	chr7	6.61592	10.5673	-3.951	0.00537
Gin1	chr1	6.78395	10.7282	-3.944	0.00039
Nt5dc2	chr14	6.94855	10.888	-3.939	0.00738
Lpcat3	chr6	6.84754	10.7843	-3.937	0.00045
N6amt2	chr14	6.44417	10.3699	-3.926	0.01413
Zfp651	chr9	6.97531	10.9004	-3.925	0.00011
Specc1	chr11	6.03283	9.95697	-3.924	0.01359
Pabpc1	chr15	10.5504	14.4707	-3.920	4.6E-05
Tmc3	chr7	7.03295	10.9525	-3.920	8.9E-05
Zyg11b	chr4	8.84514	12.7605	-3.915	4E-05
Wsb2	chr5	7.32842	11.2427	-3.914	0.00901
Bid	chr6	4.94567	8.85165	-3.906	0.01019
Zfp7	chr15	5.74317	9.64176	-3.899	0.00454
Foxo4	chrX	5.8875	9.78322	-3.896	0.01291
Tspan6	chrX	5.17988	9.07186	-3.892	0.00138
Zbtb5	chr4	6.38288	10.2738	-3.891	0.00041
Mier1	chr4	6.80007	10.6905	-3.890	0.00133
Oxr1	chr15	10.1562	14.0423	-3.886	0.00071
Mllt6	chr11	8.03887	11.9188	-3.880	0.00187
Fundc1	chrX	7.76498	11.6282	-3.863	0.00045
Ids	chrX	7.88414	11.7438	-3.860	0.00221
Ctsa	chr2	7.11468	10.9729	-3.858	0.00019
Zfp800	chr6	6.2121	10.0655	-3.853	0.00116

0610031J06Rik	chr3	7.16892	11.0196	-3.851	0.00726
Cyc1	chr15	8.15298	12.0028	-3.850	0.0002
Zfp1	chr8	4.89076	8.73976	-3.849	0.02374
2010106G01Rik	chr2	6.30708	10.1509	-3.844	0.00938
Chkb	chr15	5.89721	9.74077	-3.844	0.00379
Snx5	chr2	8.03554	11.8754	-3.840	0.00135
Lats2	chr14	7.4749	11.3137	-3.839	0.00056
Cep72	chr13	7.41284	11.2515	-3.839	0.00454
Tdg	chr10	4.79389	8.63152	-3.838	0.00474
Mrps34	chr17	4.82947	8.66708	-3.838	0.01508
Exog	chr9	5.0235	8.86107	-3.838	0.01081
Nmnat3	chr9	6.44635	10.2751	-3.829	0.00017
Efhc1	chr1	5.15427	8.98197	-3.828	0.03513
Jmjd4	chr11	5.48096	9.30365	-3.823	0.00082
Mef2a	chr7	6.51007	10.331	-3.821	0.00268
4833439L19Rik	chr13	9.32046	13.1369	-3.816	8.2E-07
Tnpo3	chr6	8.23232	12.0454	-3.813	0.0065
Mttp	chr3	5.51928	9.33186	-3.813	0.00081
Zfp346	chr13	6.96304	10.775	-3.812	0.00462
Fkbp6	chr5	8.59487	12.4065	-3.812	4.9E-05
Npnt	chr3	6.29776	10.1072	-3.809	0.01403
Zfp644	chr5	9.7978	13.606	-3.808	0.00036
Terf2ip	chr8	6.29141	10.0972	-3.806	0.00215
Dnpep	chr1	8.2033	12.0087	-3.805	0.00042
Usp40	chr1	7.51795	11.3212	-3.803	0.00456
Efcab5	chr11	4.5024	8.30178	-3.799	0.01193
Cyld	chr8	8.71155	12.5032	-3.792	0.00013
Rnf103	chr6	7.2501	11.0417	-3.792	0.0082
Wrb	chr16	6.73199	10.522	-3.790	0.00063
Mccc2	chr13	6.33758	10.1229	-3.785	0.00127
Tmc2	chr2	5.86688	9.65128	-3.784	0.02364
Klf6	chr13	6.30439	10.088	-3.784	0.0004
Sv2c	chr13	6.16245	9.9414	-3.779	0.00288
Cln6	chr9	7.69391	11.4675	-3.774	0.00062
Efha2	chr8	6.80464	10.5779	-3.773	0.00454
Vps33a	chr5	8.0097	11.7815	-3.772	0.0002
Tomm20	chr8	8.8327	12.6009	-3.768	1.9E-05
H2afy	chr13	8.82365	12.5906	-3.767	2.1E-05
Pet112l	chr3	6.13143	9.895	-3.764	0.00048
Dennd1b	chr1	7.72969	11.483	-3.753	5.7E-05
Lcorl	chr5	5.96693	9.71683	-3.750	0.00865
Ppm1f	chr16	7.24687	10.9959	-3.749	2.6E-05
Pwp1	chr10	7.75835	11.5056	-3.747	0.00446
2310008H09Rik	chr7	8.32893	12.0761	-3.747	0.002
Suv420h1	chr19	8.51407	12.2612	-3.747	0.00151
Proser1	chr3	5.38093	9.12379	-3.743	0.00298
Acaa1a	chr9	4.77463	8.51622	-3.742	0.02668
Dpf2	chr19	8.54698	12.2879	-3.741	7.9E-05
Glt28d2	chr3	5.43454	9.17097	-3.736	0.00058
App	chr16	5.74684	9.48271	-3.736	0.00786
Lnp	chr2	7.03114	10.7653	-3.734	0.0002
Sik3	chr9	7.95139	11.6811	-3.730	0.00225
Diap2	chrX	5.77525	9.50257	-3.727	0.00074
1110038D17Rik	chr10	5.06293	8.78968	-3.727	0.02831
Irak1	chrX	7.58054	11.3073	-3.727	1.5E-05
Eef1g	chr19	10.5216	14.2401	-3.719	6.3E-05
A730085A09Rik	chr4	6.33839	10.053	-3.715	0.03358

Arf4	chr14	7.05308	10.7643	-3.711	0.00123
Npepl1	chr2	4.85537	8.5639	-3.709	0.02245
Appl2	chr10	7.57823	11.2834	-3.705	0.00536
Asl	chr5	4.98651	8.68985	-3.703	0.03735
Cbx7	chr15	7.01155	10.7065	-3.695	0.0047
Mcl1	chr3	9.41754	13.1125	-3.695	9.3E-05
Eci2	chr13	6.73033	10.4245	-3.694	0.00036
Tprkb	chr6	6.36975	10.0569	-3.687	0.00111
Gpam	chr19	5.39528	9.07999	-3.685	0.00148
Abi2	chr1	9.23146	12.9156	-3.684	0.00092
Mcart6	chrX	5.82901	9.51234	-3.683	0.00043
4932415G12Rik	chr10	7.63662	11.3191	-3.683	1.8E-05
Nucks1	chr1	12.021	15.7017	-3.681	3.5E-05
Pus10	chr11	8.05194	11.7301	-3.678	0.00452
Commd4	chr9	8.27464	11.9516	-3.677	0.00103
Lonrf2	chr1	9.5627	13.2354	-3.673	0.00052
Cd81	chr7	5.97216	9.64424	-3.672	0.0018
Usp12	chr5	9.21143	12.8807	-3.669	1.2E-05
Lphn1	chr8	8.46445	12.1284	-3.664	3.9E-06
Myo15b	chr11	5.9861	9.64954	-3.663	0.00209
Slc18a1	chr8	4.81445	8.47765	-3.663	0.01436
Perp	chr10	7.57372	11.2363	-3.663	0.00046
Tm9sf1	chr14	6.98378	10.6463	-3.663	0.00034
2610005L07Rik	chr8	6.19972	9.86095	-3.661	0.0006
Pi4kb	chr3	7.71153	11.3715	-3.660	0.00035
Spg11	chr2	9.84642	13.5052	-3.659	1.6E-06
Man1b1	chr2	6.83569	10.4922	-3.657	0.00174
Tmem106b	chr6	7.23989	10.8961	-3.656	0.00149
Chm	chrX	6.88358	10.5385	-3.655	0.00174
Stard3	chr11	6.65401	10.3083	-3.654	0.01486
Mmachc	chr4	6.03072	9.68368	-3.653	0.0011
Sgpp1	chr12	7.31757	10.9649	-3.647	0.00051
Rab36	chr10	5.80329	9.44524	-3.642	0.00092
Eps8l1	chr7	4.76383	8.40191	-3.638	0.00049
Nucb2	chr7	5.32429	8.96201	-3.638	0.03319
Ncbp1	chr4	7.6198	11.2575	-3.638	0.00189
Vars	chr17	8.20641	11.8435	-3.637	0.00673
Ankrd10	chr8	7.39772	11.0337	-3.636	3.7E-05
Zfand1	chr3	5.82427	9.45914	-3.635	0.008
Maz	chr7	6.87208	10.4963	-3.624	0.00044
Unc119b	chr5	6.42598	10.0496	-3.624	0.00841
Zfp180	chr7	7.06279	10.6849	-3.622	0.00454
Phlpp1	chr1	8.64731	12.2664	-3.619	0.00045
Mthfsd	chr8	5.56532	9.18204	-3.617	0.0008
Pyroxd1	chr6	7.09434	10.7099	-3.616	0.00019
Wdr35	chr12	5.80275	9.41623	-3.613	0.00911
Actb	chr5	11.6067	15.2189	-3.612	8.4E-08
Dusp11	chr6	8.17433	11.7859	-3.612	0.00023
Ptpn4	chr1	7.33264	10.9363	-3.604	0.00089
Wdr61	chr9	7.8664	11.4656	-3.599	0.00685
Sparc	chr11	6.45757	10.0508	-3.593	0.00396
Vrk3	chr7	7.47608	11.0682	-3.592	0.00027
Rbmx	chrX	5.94192	9.533	-3.591	0.00083
Adam12	chr7	9.7805	13.3658	-3.585	0.00214
Ggnbp2	chr11	9.29932	12.8842	-3.585	0.00032
Zfp11	chr5	4.75672	8.341	-3.584	0.01226
Cspg5	chr9	8.84509	12.4217	-3.577	0.00363

Rbmx2	chrX	7.80275	11.3743	-3.572	0.00307
Arhgap5	chr12	8.75368	12.3249	-3.571	0.00031
Maoa	chrX	6.51835	10.0867	-3.568	0.00294
A530054K11Rik	chr13	7.01041	10.5683	-3.558	0.01084
Sike1	chr3	5.75657	9.31214	-3.556	0.00181
Tor1b	chr2	5.2879	8.84237	-3.554	0.00095
Gipc1	chr8	7.13391	10.6879	-3.554	0.00031
Dusp22	chr13	6.35377	9.90588	-3.552	0.00053
1600020E01Rik	chr6	5.19554	8.74231	-3.547	0.00493
Fig4	chr10	8.03093	11.5769	-3.546	0.00073
Ctbp1	chr5	9.05788	12.586	-3.528	0.00026
Crebzf	chr7	8.3416	11.868	-3.526	0.0005
Decr2	chr17	5.7342	9.25962	-3.525	0.00533
Rras	chr7	6.21252	9.73701	-3.524	0.00238
Chek2	chr5	7.20021	10.7237	-3.523	0.00142
Zfp763	chr17	6.5859	10.1082	-3.522	0.00035
Tcfl5	chr2	6.7872	10.3059	-3.519	0.00339
Bcl2l2	chr14	6.73455	10.2517	-3.517	0.03249
Mxi1	chr19	7.19584	10.7125	-3.517	0.00419
Kri1	chr9	6.81973	10.3356	-3.516	0.00114
Acad10	chr5	5.94071	9.45631	-3.516	0.0062
Pigv	chr4	5.70671	9.22198	-3.515	0.00088
1810019J16Rik	chr4	6.62677	10.137	-3.510	0.00729
Id3	chr4	7.41082	10.9186	-3.508	0.00679
Purb	chr11	7.55862	11.0639	-3.505	0.0013
Acadl	chr1	8.07025	11.5745	-3.504	0.00328
Trim71	chr9	9.6864	13.1899	-3.504	0.00094
Zfp518b	chr5	5.24007	8.74109	-3.501	0.02785
Trub1	chr19	6.16907	9.66909	-3.500	0.02065
Myl12b	chr17	8.50978	12.0086	-3.499	0.00242
9430020K01Rik	chr18	7.52012	11.0185	-3.498	0.00028
Dync1li1	chr9	8.33474	11.831	-3.496	0.00423
Pard3b	chr1	7.73561	11.2307	-3.495	1.4E-05
Hist1h1e	chr13	6.07773	9.57151	-3.494	0.00153
Ccdc53	chr10	6.84037	10.3261	-3.486	0.01816
Ostm1	chr10	7.8916	11.374	-3.482	0.00043
Tasp1	chr2	6.0835	9.56587	-3.482	0.03725
Aplf	chr6	11.7191	15.2005	-3.481	9.4E-06
Sp4	chr12	6.92552	10.4019	-3.476	0.00381
Faf2	chr13	8.45059	11.9229	-3.472	0.00021
A330021E22Rik	chr5	5.17037	8.63738	-3.467	0.04773
Sfxn2	chr19	6.00863	9.47326	-3.465	0.00144
Arih2	chr9	9.59027	13.0492	-3.459	0.00021
Zxda	chrX	5.39703	8.85449	-3.457	0.00106
Mgea5	chr19	11.5695	15.0254	-3.456	8.6E-07
Reck	chr4	5.10797	8.56213	-3.454	0.00652
Hmmr	chr11	8.77124	12.2245	-3.453	7.1E-06
Fnbp1	chr2	7.97709	11.4286	-3.452	0.00749
Yy1	chr12	8.32278	11.7729	-3.450	9.2E-05
2610029G23Rik	chrX	7.0406	10.4843	-3.444	0.00011
F2rl1	chr13	5.27325	8.71521	-3.442	0.00056
Ctnna2	chr6	5.17908	8.62018	-3.441	0.00314
Necap1	chr6	9.12052	12.5567	-3.436	0.00111
Ormdl2	chr10	6.19181	9.6247	-3.433	0.00932
Prkcz	chr4	6.13927	9.57069	-3.431	0.00129
Phka1	chrX	7.36423	10.7942	-3.430	0.00018
Mrpl19	chr6	7.07855	10.5067	-3.428	7.2E-06

Rxrb	chr17	7.85998	11.288	-3.428	0.00886
Epas1	chr17	7.65866	11.0864	-3.428	0.01571
Mark3	chr12	8.59694	12.0235	-3.427	0.00015
Atp4a	chr7	5.04205	8.46844	-3.426	0.00529
Ptgds	chr2	5.33565	8.76055	-3.425	0.04777
Itih2	chr2	5.14924	8.57209	-3.423	0.02662
Tcf12	chr9	9.88723	13.309	-3.422	4.4E-05
Trim12c	chr7	7.46368	10.882	-3.418	0.00055
Asah1	chr8	7.23097	10.6483	-3.417	0.00607
Urb2	chr8	7.7334	11.1487	-3.415	0.00067
Ccdc134	chr15	5.80215	9.21126	-3.409	0.02066
Akap11	chr14	11.5735	14.9819	-3.408	0.00069
Btbd3	chr2	6.29601	9.70366	-3.408	0.00362
Wdr81	chr11	8.32523	11.7319	-3.407	0.00594
Nop2	chr6	8.56012	11.9633	-3.403	0.00182
D930016D06Rik	chr5	7.09825	10.4989	-3.401	0.00125
Slc35e2	chr4	7.13994	10.5405	-3.401	0.00189
Pcgf5	chr19	8.91723	12.3161	-3.399	6.4E-05
Dnajb9	chr12	8.66381	12.0613	-3.397	0.00076
Ccng1	chr11	8.47039	11.8612	-3.391	1.4E-05
Set	chr2	10.2439	13.634	-3.390	0.00014
Sfi1	chr11	9.87623	13.2611	-3.385	0.0002
Rhox1	chrX	8.12282	11.5064	-3.384	0.00189
Arfgap2	chr2	7.94998	11.333	-3.383	0.00093
AI597468	chr10	7.45869	10.8397	-3.381	0.00075
Cttn	chr7	9.8148	13.1956	-3.381	0.00252
Nabp1	chr1	8.26193	11.6378	-3.376	0.00476
Cnksr2	chrX	6.38244	9.75752	-3.375	0.00269
Ech1	chr7	6.53979	9.91173	-3.372	0.00432
Tmem56	chr3	7.35886	10.7272	-3.368	0.00059
Atxn7l3b	chr10	9.50486	12.8678	-3.363	4.5E-06
1110004F10Rik	chr7	9.14818	12.5093	-3.361	0.00014
Sbno1	chr5	11.371	14.7305	-3.360	7.8E-05
Cutc	chr19	7.16869	10.5267	-3.358	0.00155
Senp2	chr16	9.20224	12.5575	-3.355	1.6E-05
Ifrd2	chr9	6.12286	9.47574	-3.353	0.02353
Rps3a	chr3	11.4504	14.8032	-3.353	0.00118
Abca2	chr2	10.0286	13.3784	-3.350	0.00062
Podxl2	chr6	6.06674	9.41575	-3.349	0.01776
Gabarap	chr11	8.52652	11.8749	-3.348	0.00035
Prdm9	chr17	7.87766	11.2202	-3.343	4.6E-05
Zfp715	chr7	8.71932	12.0609	-3.342	0.00329
Creld1	chr6	5.69292	9.0327	-3.340	0.02024
Fam102b	chr3	5.67122	9.00879	-3.338	0.02195
Cyth1	chr11	6.83304	10.17	-3.337	0.00053
Hccs	chrX	8.71956	12.0557	-3.336	0.00118
Sesn1	chr10	6.7947	10.1261	-3.331	0.02775
Ppat	chr5	7.4244	10.7518	-3.327	0.00056
Atm	chr9	9.86103	13.1882	-3.327	0.00172
Slc25a36	chr9	8.56373	11.8903	-3.327	0.00183
Ptpla	chr2	5.99086	9.31707	-3.326	0.00041
Bcl7a	chr5	7.83645	11.1598	-3.323	0.00153
Isca2	chr12	5.17758	8.49632	-3.319	0.00486
Fam101b	chr11	6.58522	9.90118	-3.316	0.00054
Abca5	chr11	8.21223	11.5267	-3.314	0.00023
Gtf3c5	chr2	7.04353	10.3558	-3.312	0.00042
Fam175b	chr7	8.69241	12.0007	-3.308	0.00141

Clec2l	chr6	5.58936	8.8942	-3.305	0.00196
Map2k5	chr9	7.18765	10.4909	-3.303	0.00617
Camk2d	chr3	5.61369	8.9157	-3.302	0.01345
Cryz	chr3	7.0764	10.378	-3.302	0.00647
Zfp317	chr9	6.56816	9.86922	-3.301	0.00146
Cox4i2	chr2	6.58924	9.88999	-3.301	0.00687
Fam63b	chr9	6.55018	9.84885	-3.299	0.00141
Pafah1b2	chr9	10.4568	13.7508	-3.294	4.3E-05
Aen	chr7	10.2476	13.5403	-3.293	9.2E-07
Snx30	chr4	7.974	11.2635	-3.290	0.00156
Macrod1	chr19	6.80978	10.0945	-3.285	0.00148
Ddhd2	chr8	9.69769	12.9819	-3.284	0.00035
Srsf9	chr5	7.68166	10.9646	-3.283	0.00327
Zmym5	chr14	8.40092	11.6829	-3.282	0.00036
Tet2	chr3	8.67706	11.958	-3.281	0.00507
AW549877	chr15	9.59887	12.8775	-3.279	2.3E-05
Zcchc11	chr4	7.25374	10.5281	-3.274	0.01025
Tmem167b	chr3	8.03921	11.311	-3.272	0.00065
Manbal	chr2	6.20225	9.47331	-3.271	0.01052
Ttc3	chr16	10.5549	13.8256	-3.271	0.00063
B230219D22Rik	chr13	7.33833	10.607	-3.269	0.00322
Sohlh2	chr3	8.89394	12.1616	-3.268	0.00476
A730017L22Rik	chr2	5.62159	8.88899	-3.267	0.01381
Pgls	chr8	5.18569	8.45266	-3.267	0.00169
Fmr1	chrX	10.4798	13.744	-3.264	9.9E-05
Chka	chr19	6.60184	9.86459	-3.263	0.0005
Sos1	chr17	9.84092	13.1014	-3.260	0.00056
Ipo11	chr13	9.43104	12.6904	-3.259	1.8E-06
Tmem192	chr8	6.31278	9.56876	-3.256	0.02653
5430440P10Rik	chr14	5.7946	9.04441	-3.250	0.00765
Mns1	chr9	7.64421	10.8938	-3.250	0.0117
Pak2	chr16	8.5301	11.7785	-3.248	0.00374
Mettl10	chr7	6.671	9.91876	-3.248	0.02098
Trp63	chr16	11.2082	14.453	-3.245	5.5E-05
Ralgapa1	chr12	9.37017	12.6139	-3.244	0.00022
Mcf2	chrX	6.41502	9.65862	-3.244	0.00488
Bcl10	chr3	5.39186	8.63387	-3.242	0.00918
Ppia	chr11	11.4781	14.7199	-3.242	0.00119
Pogk	chr1	7.62695	10.8674	-3.240	0.00334
Sltm	chr9	8.91524	12.15	-3.235	4.2E-05
Grpel2	chr18	9.02107	12.2504	-3.229	0.00028
Tmed7	chr18	8.50414	11.7331	-3.229	0.0003
Zfp945	chr17	7.18313	10.4069	-3.224	5.9E-05
Shmt1	chr11	6.88745	10.1098	-3.222	0.00227
Pycr1	chr11	7.05577	10.2755	-3.220	0.00179
Gtf2h4	chr17	7.80841	11.0249	-3.216	0.00081
Gmeb2	chr2	6.49744	9.71045	-3.213	0.0005
Zfp810	chr9	6.139	9.35152	-3.213	0.00412
Mbnl1	chr3	6.61255	9.82373	-3.211	0.00168
Ankrd11	chr8	8.96444	12.1691	-3.205	0.00227
Gabpb2	chr3	9.15855	12.3617	-3.203	0.00254
Pold1	chr7	9.01204	12.2141	-3.202	0.00024
D16Ertd472e	chr16	7.57977	10.7794	-3.200	0.00203
Myt1l	chr12	7.18296	10.3821	-3.199	0.00019
Bckdk	chr7	8.19143	11.3869	-3.196	0.00011
Gtdc2	chr9	7.60387	10.7991	-3.195	0.00672
Commd7	chr2	7.30423	10.4974	-3.193	0.00263

Creb1	chr1	7.81644	11.009	-3.193	0.00013
Nr1d2	chr14	9.22828	12.4159	-3.188	3.8E-05
Cxadr	chr16	5.70342	8.88799	-3.185	0.00152
Abce1	chr8	9.85655	13.0374	-3.181	0.00057
Slc5a3	chr16	8.4842	11.6572	-3.173	0.00159
Cox11	chr11	6.00221	9.16634	-3.164	0.00456
Fam171a1	chr2	7.85459	11.0187	-3.164	0.00036
2410089E03Rik	chr15	11.3497	14.513	-3.163	0.00442
Zfp362	chr4	6.00452	9.16574	-3.161	0.00347
AI480653	chr16	7.16334	10.3222	-3.159	0.00189
Iah1	chr12	6.17799	9.33365	-3.156	0.0054
Tgs1	chr4	7.14922	10.3029	-3.154	0.00296
Ccdc88c	chr12	11.1983	14.3511	-3.153	3.3E-05
Arhgef26	chr3	9.15038	12.303	-3.153	0.00012
Rbm44	chr1	8.90174	12.0528	-3.151	3.4E-05
Hadh	chr3	7.83226	10.9806	-3.148	0.00561
4732471D19Rik	chr13	7.2197	10.3676	-3.148	0.01915
Capn5	chr7	7.7162	10.8616	-3.145	0.00297
Pigh	chr12	6.87245	10.0131	-3.141	0.00121
Hdac1	chr4	8.68313	11.8213	-3.138	6.9E-05
Cblb	chr16	7.11113	10.2463	-3.135	0.00163
Impact	chr18	7.56402	10.6977	-3.134	0.00114
G2e3	chr12	7.49069	10.6222	-3.132	0.00207
Ccni	chr5	11.2503	14.3797	-3.129	0.00057
Hmgcl	chr4	6.07257	9.20195	-3.129	0.00804
Ilk	chr7	8.95117	12.08	-3.129	0.00138
Tmx3	chr18	8.03891	11.1671	-3.128	0.00385
Arpc5	chr1	8.76421	11.8923	-3.128	0.0003
Klhl7	chr5	6.07356	9.19981	-3.126	0.0075
Asb1	chr1	7.36227	10.488	-3.126	6.1E-05
Smarcc1	chr9	10.9182	14.0421	-3.124	4.4E-05
Rfc4	chr16	8.70761	11.8308	-3.123	0.00088
Mdp1	chr14	6.04297	9.16447	-3.121	0.00108
Tceb2	chr17	7.02927	10.1456	-3.116	0.00423
Cpne3	chr4	10.4085	13.5243	-3.116	0.00017
Tspan17	chr13	7.01134	10.1261	-3.115	0.00054
Esf1	chr2	8.98197	12.0944	-3.112	0.00067
Wdr7	chr18	8.93794	12.0499	-3.112	0.00015
Hdac6	chrX	10.3321	13.4405	-3.108	3E-05
C130026I21Rik	chr1	8.33487	11.4394	-3.105	7.2E-05
Zfp251	chr15	7.08878	10.19	-3.101	0.00546
Tubgcp4	chr2	8.02791	11.1281	-3.100	0.01922
Rab11fip2	chr19	8.8124	11.9122	-3.100	0.0001
Nampt	chr12	9.26638	12.3657	-3.099	3.1E-05
Stard10	chr7	7.65187	10.7469	-3.095	0.00272
Rnf7	chr9	9.61011	12.7028	-3.093	0.00621
Arrdc3	chr13	8.19711	11.2861	-3.089	0.00034
Ier3ip1	chr18	7.14813	10.2371	-3.089	0.00166
Fam116b	chr15	5.42129	8.50993	-3.089	0.00315
Uhrf2	chr19	7.18273	10.2706	-3.088	0.00025
Fbxo45	chr16	8.39016	11.4764	-3.086	0.00076
Bpnt1	chr1	8.98912	12.0716	-3.082	7.3E-05
Bcmo1	chr8	7.18119	10.2575	-3.076	0.00017
Nbea	chr3	9.37261	12.4452	-3.073	3.1E-05
Tmem101	chr11	7.05567	10.124	-3.068	0.03388
Mpp7	chr18	6.3512	9.4156	-3.064	0.01077
Dnahc17	chr11	9.58281	12.6445	-3.062	1.5E-05

Nubp1	chr16	7.63854	10.6957	-3.057	0.00166
Zfp36l1	chr12	6.8951	9.94463	-3.050	0.00706
Arf2	chr11	6.78704	9.83598	-3.049	0.00614
Gart	chr16	8.78835	11.8332	-3.045	0.0001
Hfm1	chr5	7.36186	10.4042	-3.042	0.00648
Ran	chr5	10.8867	13.9247	-3.038	0.00199
D15Ertd621e	chr15	11.0515	14.0886	-3.037	6.2E-06
Braf	chr6	9.98697	13.0233	-3.036	3E-05
Kctd21	chr7	7.25559	10.2912	-3.036	0.00873
Ttc12	chr9	6.92067	9.9547	-3.034	0.00059
Gprasp1	chrX	9.5354	12.5687	-3.033	2.5E-05
Spnb1	chr12	6.53124	9.56396	-3.033	0.00188
Plscr1	chr9	6.76418	9.79546	-3.031	0.01142
Etnk1	chr6	11.7743	14.8035	-3.029	4.9E-06
Sft2d2	chr1	9.85017	12.8768	-3.027	7.4E-05
Rbbp6	chr7	10.0188	13.0402	-3.021	1.7E-05
Tgoln1	chr6	11.5016	14.52	-3.018	7.1E-06
Tex11	chrX	9.1744	12.1836	-3.009	3.9E-05
Ccs	chr19	9.89768	12.9051	-3.007	0.00086
A130010J15Rik	chr1	7.92814	10.9316	-3.003	0.00246
Insr	chr8	9.18826	12.1851	-2.997	0.0005
Ppip5k1	chr2	7.56693	10.5624	-2.995	0.00105
Fam134a	chr1	8.28422	11.2796	-2.995	0.00717
Dhx37	chr5	7.29379	10.2865	-2.993	0.00059
2810021B07Rik	chr13	6.24446	9.23472	-2.990	0.01533
Herc4	chr10	8.59563	11.5815	-2.986	0.00284
Ndrg3	chr2	6.47757	9.46293	-2.985	0.00016
5730508B09Rik	chr3	5.41441	8.39876	-2.984	6.6E-05
Fnip2	chr3	9.24116	12.2244	-2.983	0.00063
Prkab2	chr3	9.58996	12.5722	-2.982	4E-05
Tle4	chr19	10.6356	13.6174	-2.982	0.00012
2010317E24Rik	chr2	6.4039	9.38406	-2.980	0.00195
Foxj2	chr6	9.30852	12.2875	-2.979	0.00018
Map3k2	chr18	7.59468	10.5721	-2.977	0.00049
Zfp217	chr2	11.3302	14.3042	-2.974	0.00068
6530401N04Rik	chr12	6.82415	9.79729	-2.973	0.00091
Rcbtb1	chr14	9.61233	12.5853	-2.973	0.00052
Ammecr1	chrX	10.002	12.9736	-2.972	0.00048
Tspan13	chr12	9.73473	12.7037	-2.969	2.7E-05
Zbtb26	chr2	8.74621	11.7127	-2.966	0.00336
Prickle2	chr6	9.89364	12.8579	-2.964	6E-05
Marcks	chr10	6.75818	9.7222	-2.964	0.01535
Cox15	chr19	7.5661	10.53	-2.964	0.00118
Celsr2	chr3	6.1677	9.12589	-2.958	0.00184
Snx27	chr3	9.52583	12.4827	-2.957	7.9E-05
Igf2	chr7	6.00309	8.95984	-2.957	0.03832
Rad9b	chr5	6.48284	9.43949	-2.957	0.0059
Hipk1	chr3	7.91296	10.8688	-2.956	0.00058
Ruvbl1	chr6	8.63359	11.5846	-2.951	7.8E-05
Zfp772	chr7	5.77439	8.71861	-2.944	0.0082
Zfp263	chr16	8.7864	11.7294	-2.943	0.00167
Fkbp3	chr12	8.07163	11.014	-2.942	0.00082
Mgmt	chr7	7.38157	10.3231	-2.942	0.01193
Eln	chr5	6.98888	9.92824	-2.939	0.00032
Zfp14	chr7	5.36728	8.30486	-2.938	0.00091
Vps26b	chr9	6.86524	9.80171	-2.936	0.00237
Tceanc	chrX	8.31774	11.2534	-2.936	0.00247

Fam120a	chr13	10.4927	13.4281	-2.935	0.00027
Crocc	chr4	6.25482	9.18716	-2.932	0.0004
Ankrd46	chr15	7.33457	10.262	-2.927	0.00051
Ddx10	chr9	10.8793	13.8058	-2.927	5.6E-06
Polr2m	chr9	10.3581	13.2841	-2.926	0.00093
Syne1	chr10	8.48017	11.4041	-2.924	0.00115
Irak1bp1	chr9	5.87758	8.80016	-2.923	0.00015
Hcfc2	chr10	8.21544	11.1368	-2.921	0.00028
D930020B18Rik	chr10	7.46947	10.3877	-2.918	0.0123
Prkd3	chr17	8.76288	11.6811	-2.918	0.00557
Dap	chr15	6.20558	9.12368	-2.918	0.00585
Myot	chr18	7.67455	10.5918	-2.917	0.00078
Cald1	chr6	5.838	8.7502	-2.912	0.00735
Srsf6	chr2	10.1321	13.0433	-2.911	0.00034
Prmt3	chr7	9.37229	12.2815	-2.909	5.9E-05
Afg3l1	chr8	9.60715	12.5152	-2.908	0.00139
Hnrnpm	chr17	11.153	14.0604	-2.907	0.00026
Szt2	chr4	7.26868	10.1744	-2.906	0.00943
Kif27	chr13	7.24581	10.1502	-2.904	0.00533
1700020I14Rik	chr2	7.58208	10.4833	-2.901	0.00536
Pigt	chr2	6.10907	9.00748	-2.898	0.01532
Cbwd1	chr19	8.04493	10.9416	-2.897	7.9E-05
Suv420h2	chr7	5.719	8.61521	-2.896	0.0262
Btg3	chr16	7.1733	10.0662	-2.893	0.00164
Man2b1	chr8	6.90541	9.7948	-2.889	0.00289
Alg13	chrX	13.9036	16.7878	-2.884	3.7E-08
Figla	chr6	9.37661	12.2606	-2.884	0.00564
Smg5	chr3	8.95403	11.8371	-2.883	0.00026
Tpp1	chr7	7.12926	10.0119	-2.883	0.01374
Endov	chr11	6.41233	9.28874	-2.876	0.0069
Mib1	chr18	10.7351	13.611	-2.876	0.00086
Tmem234	chr4	7.13277	10.0079	-2.875	0.00834
Shcbp1l	chr1	8.77808	11.6453	-2.867	0.00787
Zfp146	chr7	6.34434	9.21099	-2.867	0.00825
Nsl1	chr1	7.43573	10.3024	-2.867	0.0066
Elmo2	chr2	7.44849	10.3104	-2.862	0.01122
Rap2c	chrX	8.61632	11.4731	-2.857	0.00671
Pus1	chr5	6.3365	9.19118	-2.855	0.00983
Polr1c	chr17	8.6779	11.5316	-2.854	0.00237
Mllt1	chr17	7.10071	9.95225	-2.852	0.03863
Zdhhc21	chr4	7.27264	10.1235	-2.851	0.00197
Rsbn1l	chr5	8.83803	11.6887	-2.851	9.5E-05
Pbrm1	chr14	10.8956	13.7457	-2.850	0.00034
Pspc1	chr14	8.31546	11.1655	-2.850	0.00035
Ric8	chr7	6.83892	9.68814	-2.849	0.01434
Pgm2l1	chr7	11.0244	13.8709	-2.846	0.00195
Zyx	chr6	7.69376	10.5393	-2.846	0.01014
4932438A13Rik	chr3	12.8738	15.7151	-2.841	6.2E-05
Serp1	chr3	9.89226	12.733	-2.841	0.00082
Znf41-ps	chr4	6.21119	9.045	-2.834	0.02083
Ppil1	chr17	7.41966	10.2517	-2.832	7.2E-05
Klhdc5	chr6	8.24846	11.0773	-2.829	0.00107
Sephs1	chr2	7.90672	10.7347	-2.828	0.00585
Rab11b	chr17	10.2261	13.0539	-2.828	2.8E-05
Slc30a9	chr5	8.48576	11.3116	-2.826	0.00103
Lztr1	chr16	6.53929	9.36324	-2.824	0.01519
Pink1	chr4	8.46494	11.2858	-2.821	7.3E-05

Herc1	chr9	12.8096	15.63	-2.820	1.1E-05
Ppp1r8	chr4	8.45371	11.2737	-2.820	0.00278
E2f6	chr12	6.56836	9.38729	-2.819	0.0287
Rpl3	chr15	10.1133	12.9314	-2.818	0.0004
Rangap1	chr15	10.3308	13.1479	-2.817	0.00038
Kif15	chr9	8.13698	10.9516	-2.815	0.00239
Gan	chr8	7.64205	10.4548	-2.813	0.0138
Nisch	chr14	10.0786	12.8907	-2.812	0.00091
Tes	chr6	6.68381	9.49483	-2.811	0.00096
1110007C09Rik	chr13	6.44777	9.25197	-2.804	0.00435
Fam193b	chr13	7.55923	10.3633	-2.804	0.01568
Tmco7	chr8	7.38161	10.1854	-2.804	0.00141
Fastk	chr5	8.06845	10.8709	-2.802	0.00087
BC005561	chr5	8.44083	11.2419	-2.801	0.00371
Bzw1	chr1	11.5052	14.3053	-2.800	0.00012
Zfp825	chr13	5.86814	8.6672	-2.799	0.01115
Seh1l	chr18	11.3426	14.1379	-2.795	7E-06
As3mt	chr19	7.11934	9.91414	-2.795	0.00522
Zfp521	chr18	7.53401	10.328	-2.794	0.00111
Ube2r2	chr4	9.64483	12.437	-2.792	0.0005
Larp1	chr11	10.2804	13.0716	-2.791	7.2E-05
Stk11ip	chr1	6.8277	9.61359	-2.786	0.01325
Ift57	chr16	6.24648	9.02955	-2.783	0.00792
Polr2a	chr11	12.4863	15.269	-2.783	8.9E-05
Pmm2	chr16	7.68356	10.4635	-2.780	0.00321
Cnpy1	chr5	9.27528	12.0507	-2.775	0.00077
Rlf	chr4	10.0931	12.863	-2.770	0.00057
Chmp4b	chr2	9.18022	11.9483	-2.768	0.00089
Ap2a2	chr7	9.23684	12.0047	-2.768	2.4E-05
Serinc3	chr2	11.277	14.0438	-2.767	9.7E-06
Zfp326	chr5	7.76614	10.5292	-2.763	0.0036
Hist1h1c	chr13	7.89422	10.6555	-2.761	0.00293
Gfpt1	chr6	10.4249	13.1847	-2.760	3.6E-05
Coil	chr11	8.49168	11.2491	-2.757	0.00015
Fbn1	chr2	6.1136	8.87081	-2.757	0.00044
Fam20b	chr1	8.94035	11.6959	-2.756	0.00088
Setd5	chr6	10.307	13.0611	-2.754	0.00061
Ninl	chr2	7.16968	9.92256	-2.753	0.00215
Tgfbr1	chr4	10.6163	13.361	-2.745	8.9E-05
Tsc2	chr17	10.0317	12.7741	-2.742	0.00072
Ube2v2	chr16	8.81545	11.5573	-2.742	0.00015
Cep97	chr16	7.61014	10.3499	-2.740	0.00051
Stx11	chr10	6.28443	9.02407	-2.740	0.00325
Sema3c	chr5	6.72219	9.46041	-2.738	0.02261
Tgfa	chr6	7.19744	9.93539	-2.738	0.02239
2410002F23Rik	chr7	7.56319	10.3007	-2.737	0.00935
Pias3	chr3	6.23354	8.97084	-2.737	0.00245
Plekhb2	chr1	8.49561	11.2318	-2.736	0.00011
Pnpla7	chr2	8.46725	11.1985	-2.731	0.00032
Rptor	chr11	9.66293	12.3923	-2.729	0.00037
Ugp2	chr11	7.67701	10.4047	-2.728	0.00104
Spopl	chr2	6.79367	9.51973	-2.726	0.00246
Rint1	chr5	8.20978	10.9322	-2.722	0.00259
Smyd2	chr1	7.73954	10.4603	-2.721	0.00436
Dtwd2	chr18	8.06232	10.7821	-2.720	0.00096
Crot	chr5	8.42883	11.1485	-2.720	0.00223
D14Abb1e	chr14	10.9081	13.6253	-2.717	0.00164

Prpf4	chr4	7.98108	10.6982	-2.717	0.00925
Stx16	chr2	10.2865	13.0029	-2.716	0.00329
Strn3	chr12	9.32964	12.0456	-2.716	0.00035
Irf2bp1	chr7	6.29496	9.0105	-2.716	0.00063
Gab1	chr8	6.84823	9.56297	-2.715	0.00669
Birc2	chr9	8.77551	11.4893	-2.714	0.00772
Tom1	chr8	6.60292	9.31329	-2.710	0.00111
Dhrs7b	chr11	6.77099	9.48049	-2.709	0.00259
Igf1r	chr7	11.8828	14.5872	-2.704	0.00074
Arl5a	chr2	9.6477	12.3504	-2.703	0.00041
Mtch2	chr2	7.62137	10.3239	-2.703	0.0082
9030025P20Rik	chr17	6.55722	9.25815	-2.701	0.00078
Fbxw7	chr3	10.2351	12.9347	-2.700	0.0001
Zbtb43	chr2	8.69004	11.3807	-2.691	0.00012
Pddc1	chr7	6.73923	9.42962	-2.690	0.00228
Prss53	chr7	6.43049	9.1188	-2.688	0.00248
Cstf2	chrX	10.498	13.185	-2.687	0.00048
Carf	chr1	6.22755	8.91246	-2.685	0.00089
Slc25a33	chr4	6.25448	8.93866	-2.684	0.00014
Zfp609	chr9	11.1857	13.8662	-2.680	7.1E-05
Zfp953	chr13	6.87035	9.55062	-2.680	0.00565
Ovol1	chr19	7.71523	10.3954	-2.680	0.00561
Hmbs	chr9	7.91391	10.5903	-2.676	0.00284
Nme1	chr11	8.35099	11.0267	-2.676	0.00369
Rab5a	chr17	8.51133	11.1854	-2.674	0.00067
Pik3r2	chr8	8.2649	10.9358	-2.671	0.00524
Usp22	chr11	9.70591	12.3739	-2.668	0.00038
Rpl37a	chr1	10.3206	12.9885	-2.668	0.00275
Klhl21	chr4	9.16651	11.834	-2.668	0.00142
B230118H07Rik	chr2	8.83584	11.5023	-2.666	0.01017
Tti2	chr8	8.13546	10.8014	-2.666	0.00737
Depdc1b	chr13	6.51751	9.18335	-2.666	0.0061
Cspp1	chr1	8.31991	10.9843	-2.664	0.003
Etohi1	chr2	7.30219	9.96615	-2.664	0.00128
Gstk1	chr6	8.07798	10.7417	-2.664	0.00131
Ppp3cb	chr14	10.6971	13.3598	-2.663	0.00057
Nup210	chr6	8.54051	11.203	-2.662	0.0022
Necap2	chr4	10.4904	13.1524	-2.662	0.00212
Rps27	chr3	8.92346	11.5854	-2.662	0.00763
Flywch1	chr17	6.81224	9.47334	-2.661	0.00165
Cndp2	chr18	7.2426	9.90164	-2.659	0.00376
Bin1	chr18	7.04128	9.70031	-2.659	0.00074
Otud7b	chr3	9.698	12.3502	-2.652	1.6E-05
Polr3c	chr3	9.32954	11.9799	-2.650	0.00319
Tubgcp3	chr8	9.00936	11.6574	-2.648	0.00136
Gm3435	chr17	6.2294	8.87713	-2.648	0.00578
Zfp12	chr5	7.33477	9.98166	-2.647	0.00829
Rpl7	chr1	11.3777	14.0242	-2.647	0.00026
Gpbp1l1	chr4	11.5849	14.229	-2.644	5.2E-05
Ptplad1	chr9	10.6706	13.3142	-2.644	0.00188
Araf	chrX	9.16633	11.8093	-2.643	0.00037
Efcab2	chr1	7.11659	9.75934	-2.643	0.00051
B4galt1	chr4	6.49848	9.1412	-2.643	0.00255
Nfatc3	chr8	7.87672	10.5191	-2.642	0.00069
Sh3gl2	chr4	8.24729	10.8893	-2.642	0.03329
Cyp20a1	chr1	8.05747	10.6993	-2.642	0.0006
Jmy	chr13	9.04411	11.6842	-2.640	0.00016

Atg4c	chr4	6.88746	9.52745	-2.640	0.00032
Pnpla6	chr8	10.7401	13.3789	-2.639	4.4E-05
Fbxw2	chr2	8.94127	11.5798	-2.639	0.0014
Mfsd5	chr15	7.45342	10.0919	-2.638	0.00072
Yme1l1	chr2	10.2072	12.8441	-2.637	0.0005
Ptges	chr2	6.54478	9.18157	-2.637	0.00779
Atp5g3	chr2	8.25838	10.8942	-2.636	0.00232
Ppp2r2c	chr5	6.74283	9.37755	-2.635	0.01238
Arl5b	chr2	7.8867	10.5194	-2.633	0.01369
Saal1	chr7	7.72465	10.3571	-2.632	0.0044
LOC100503956	chr8	7.1207	9.7506	-2.630	0.00351
Uevld	chr7	8.1407	10.7681	-2.627	0.00333
H2-K1	chr17	10.0301	12.6571	-2.627	6.6E-06
Nsg2	chr11	7.62081	10.246	-2.625	0.0009
Srsf11	chr3	9.41508	12.0381	-2.623	0.00051
Gemin5	chr11	9.3389	11.961	-2.622	0.0007
Hmgn2	chr4	9.11768	11.7384	-2.621	0.0006
Dcxr	chr11	6.06092	8.68094	-2.620	0.03636
Evc	chr5	6.83444	9.45111	-2.617	0.00697
0610010F05Rik	chr11	8.51758	11.1322	-2.615	0.00118
Ap2a1	chr7	9.36173	11.9762	-2.614	0.00278
Oscp1	chr4	6.05845	8.66875	-2.610	0.00791
Tmem55b	chr14	8.4884	11.0982	-2.610	0.00498
Fkbp4	chr6	10.4449	13.0543	-2.609	1.9E-05
Fto	chr8	9.06508	11.6745	-2.609	0.00121
Huwe1	chrX	14.618	17.2273	-2.609	0.00064
Mrpl20	chr4	6.29771	8.90697	-2.609	4E-05
Trim8	chr19	7.12855	9.73778	-2.609	0.00186
Slc25a3	chr10	11.1371	13.746	-2.609	0.00226
Xpnpep1	chr19	6.78061	9.38359	-2.603	0.01941
Stag3	chr5	8.38714	10.9897	-2.603	0.00048
Chd2	chr7	8.8045	11.403	-2.598	0.00068
Plec	chr15	10.1518	12.7497	-2.598	0.00021
Ddx1	chr12	11.4355	14.0293	-2.594	7E-05
Asb7	chr7	7.00249	9.59523	-2.593	0.00358
Prrc1	chr18	9.38588	11.9775	-2.592	0.00014
Itgav	chr2	8.3904	10.981	-2.591	0.00031
Trrap	chr5	11.8416	14.4314	-2.590	0.00016
Marveld2	chr13	10.1248	12.7146	-2.590	0.00044
2210013O21Rik	chrX	5.71577	8.30291	-2.587	0.00446
Wnk3-ps	chrX	8.195	10.7816	-2.587	0.0311
2310067B10Rik	chr11	8.83954	11.4237	-2.584	0.00048
Mrps10	chr17	7.32795	9.91051	-2.583	0.01445
Taf1c	chr8	7.40844	9.98949	-2.581	0.00327
Xk	chrX	7.30413	9.88509	-2.581	0.03081
Mta2	chr19	6.81126	9.38961	-2.578	0.00164
Nek4	chr14	8.51422	11.0909	-2.577	0.00036
Rasd2	chr8	9.37517	11.9518	-2.577	0.0007
Vps51	chr19	8.78443	11.3598	-2.575	0.00164
4930562C15Rik	chr16	11.2095	13.7832	-2.574	0.00093
Arglu1	chr8	9.3664	11.9365	-2.570	0.00222
Hip1	chr5	9.20169	11.7709	-2.569	0.00032
Slit2	chr5	5.93754	8.50554	-2.568	0.02193
Dock8	chr19	8.23691	10.8028	-2.566	0.00184
Rock2	chr12	10.6737	13.2371	-2.563	0.00216
Samm50	chr15	10.4348	12.9963	-2.562	0.00138
Stam2	chr2	9.38412	11.9453	-2.561	0.00022

Ftl1	chr7	10.4282	12.9891	-2.561	0.00013
Impdh1	chr6	7.04717	9.60472	-2.558	0.0326
Itpr3	chr17	8.96894	11.5252	-2.556	0.00148
Cdadc1	chr14	7.54647	10.1017	-2.555	0.00297
Gtf3c4	chr2	8.1826	10.7354	-2.553	0.00891
4632415K11Rik	chr8	9.46584	12.0159	-2.550	0.00159
Slc12a6	chr2	11.7335	14.2823	-2.549	0.0003
Tmed4	chr11	7.11838	9.66594	-2.548	0.00673
Myh14	chr7	8.32914	10.8749	-2.546	0.00107
Erbb4	chr1	6.08571	8.63101	-2.545	0.01055
Nudt16	chr9	6.08347	8.62639	-2.543	0.02936
Parp1	chr1	12.0164	14.5585	-2.542	6.9E-07
Scai	chr2	9.13096	11.6726	-2.542	0.00973
R3hcc1	chr14	7.45942	9.99903	-2.540	0.00153
Pet2	chrX	7.50001	10.0371	-2.537	0.02296
Chml	chr1	8.41696	10.9538	-2.537	0.00401
Mink1	chr11	11.2576	13.7923	-2.535	0.00067
Gosr1	chr11	8.76523	11.299	-2.534	0.00033
4930444A02Rik	chr8	7.85556	10.3869	-2.531	0.00416
Tenm4	chr7	8.51085	11.0406	-2.530	0.00169
Dpp3	chr19	9.11272	11.637	-2.524	3.2E-05
Coa3	chr11	6.73566	9.25936	-2.524	0.00075
Hspa13	chr16	8.24752	10.7698	-2.522	0.00489
Gpaa1	chr15	8.20813	10.7298	-2.522	0.00064
A930001N09Rik	chr17	9.80111	12.3191	-2.518	0.0006
Klf10	chr15	8.55792	11.0754	-2.518	0.00048
Nudt21	chr8	8.73785	11.252	-2.514	0.00504
Eif2b5	chr16	8.48346	10.9975	-2.514	0.00208
Nxt2	chrX	7.50616	10.02	-2.514	0.00061
Pkp2	chr16	9.11658	11.63	-2.513	0.00127
Cbx8	chr11	5.81001	8.32338	-2.513	0.03764
Isoc1	chr18	8.91284	11.424	-2.511	0.00217
Zfp868	chr8	6.53106	9.04134	-2.510	0.00187
Smc2	chr4	10.9588	13.4689	-2.510	0.00033
Ift172	chr5	9.9611	12.4695	-2.508	0.00034
Ttc14	chr3	8.47578	10.984	-2.508	0.00399
Anapc13	chr9	9.22346	11.731	-2.508	0.00984
Pds5a	chr5	10.0114	12.5179	-2.506	0.00223
Dnajc27	chr12	6.52648	9.03127	-2.505	0.03933
Atp5d	chr10	8.42955	10.9339	-2.504	0.00176
Glod4	chr11	7.76961	10.2736	-2.504	0.04687
Phka2	chrX	11.2317	13.7346	-2.503	0.00027
Frs2	chr10	10.696	13.1966	-2.501	0.00012
Lgals8	chr13	7.86303	10.3625	-2.499	0.02848
Fam96a	chr9	7.73838	10.2355	-2.497	0.03388
1700037H04Rik	chr2	6.39842	8.89404	-2.496	0.00857
Tdrd9	chr12	11.3698	13.8638	-2.494	0.00059
Unkl	chr17	6.72368	9.21769	-2.494	0.04173
Alkbh4	chr5	6.46942	8.96329	-2.494	0.00247
Zfp704	chr3	9.8597	12.3532	-2.493	0.0003
Dars2	chr1	8.37061	10.863	-2.492	0.00057
Zfp192	chr13	11.2862	13.7776	-2.491	0.00146
Rpsa	chr9	12.1987	14.69	-2.491	0.00082
Prkcb	chr7	7.16019	9.64908	-2.489	0.00762
Rnf167	chr11	9.88178	12.3665	-2.485	0.00056
Myom1	chr17	7.02832	9.51135	-2.483	0.0141
AW209491	chr13	8.18296	10.6649	-2.482	0.00488

St6galnac2	chr11	7.9772	10.4586	-2.481	8E-05
Wipi2	chr5	10.1002	12.5801	-2.480	0.00021
Alad	chr4	7.58522	10.0639	-2.479	0.00693
Cpe	chr8	6.69942	9.17738	-2.478	0.00935
Fam100b	chr11	6.13343	8.60874	-2.475	0.00028
Npepps	chr11	9.35411	11.8293	-2.475	0.00022
Actr1b	chr1	7.88771	10.3615	-2.474	0.0132
Uggt2	chr14	7.12826	9.60057	-2.472	0.002
Zfp384	chr6	8.84327	11.3147	-2.471	8E-05
Nes	chr3	8.58079	11.0519	-2.471	1.5E-05
Paqr3	chr5	7.24542	9.71641	-2.471	0.00454
Tex15	chr8	13.2075	15.6784	-2.471	0.00394
Dnajc24	chr2	6.15966	8.63031	-2.471	0.00793
Dhx9	chr1	11.8044	14.2734	-2.469	8.8E-05
Emc4	chr2	8.44592	10.9139	-2.468	0.00245
Pdss1	chr2	6.22348	8.69118	-2.468	0.00565
Cep128	chr12	9.67704	12.1446	-2.468	0.00117
Ube2l3	chr16	9.10799	11.5751	-2.467	0.00101
Actg1	chr11	13.1952	15.6591	-2.464	4.5E-05
Ints2	chr11	10.4481	12.9116	-2.463	1.4E-05
Vmn2r-ps16	chr4	6.90721	9.37062	-2.463	0.0013
Mien1	chr11	9.41015	11.8728	-2.463	0.00032
BC002230	chr12	8.01398	10.4754	-2.461	0.00347
Cry2	chr2	7.41401	9.87503	-2.461	0.00745
Bag5	chr12	8.6283	11.0875	-2.459	0.00116
41706	chr6	10.1409	12.598	-2.457	0.00027
Zfp869	chr8	8.39336	10.8497	-2.456	0.00253
Fbl	chr7	10.0293	12.4855	-2.456	0.00192
Rgs9	chr11	8.337	10.7924	-2.455	0.00699
Slc38a10	chr11	9.49526	11.9505	-2.455	0.00044
Oip5	chr2	6.50103	8.95618	-2.455	0.01955
Vps36	chr8	7.25743	9.71141	-2.454	0.00368
Atr	chr9	12.3565	14.809	-2.453	1.8E-05
Ddx4	chr13	11.1777	13.6282	-2.451	2.4E-05
Ttll12	chr15	8.51513	10.9646	-2.449	0.00214
Suclg2	chr6	10.606	13.0551	-2.449	0.0006
Aup1	chr6	6.55037	8.99928	-2.449	0.04403
Rufy2	chr10	8.68267	11.1316	-2.449	4.3E-05
Rab1	chr11	10.7133	13.1613	-2.448	2.8E-05
Ahi1	chr10	6.48003	8.92711	-2.447	0.00732
Mrpl23	chr7	8.11299	10.5599	-2.447	0.00939
Ift88	chr14	9.11335	11.5579	-2.445	0.00171
1110057K04Rik	chr12	7.57116	10.013	-2.442	0.00535
Pja2	chr17	10.8644	13.3041	-2.440	0.00132
Zcwpw1	chr5	9.89503	12.3329	-2.438	0.00506
Peg3	chr7	10.0881	12.5251	-2.437	0.00026
Spg7	chr8	8.51691	10.9467	-2.430	0.00414
Hars	chr18	9.36582	11.7936	-2.428	0.00037
Ciz1	chr2	7.99551	10.4211	-2.426	0.00086
Ttc13	chr8	8.74307	11.1685	-2.425	0.00334
Clpp	chr17	8.63517	11.0603	-2.425	0.003
Ctu2	chr8	8.26055	10.6831	-2.423	0.00452
BC027231	chr16	8.64742	11.0698	-2.422	0.00028
Ccbl2	chr3	9.47384	11.8955	-2.422	0.00117
2700094K13Rik	chr2	9.22119	11.6406	-2.419	0.00104
Hexdc	chr11	10.4437	12.8627	-2.419	0.00185
Plk1s1	chr2	8.77917	11.1976	-2.418	0.01053

Enpp4	chr17	9.17056	11.5888	-2.418	0.00107
Snap91	chr9	9.3492	11.7659	-2.417	0.001
Fam120c	chrX	8.79201	11.2085	-2.416	0.00327
Chst10	chr1	6.30982	8.72583	-2.416	0.01883
Exd2	chr12	8.62736	11.0421	-2.415	0.0018
Vps54	chr11	10.0738	12.4858	-2.412	0.00254
Mettl5	chr2	8.51794	10.9266	-2.409	0.00399
Prmt5	chr14	9.971	12.3782	-2.407	0.00259
Ap4b1	chr3	7.65384	10.0601	-2.406	0.02916
Kansl1l	chr1	8.50492	10.9099	-2.405	7.8E-05
Rps3	chr7	12.6699	15.0746	-2.405	0.00044
Bckdha	chr7	8.34424	10.7465	-2.402	0.03726
Prpf40b	chr15	8.64446	11.0403	-2.396	0.00162
Emc10	chr7	8.99523	11.39	-2.395	0.00045
Skiv2l	chr17	10.6748	13.0688	-2.394	0.00115
1810012P15Rik	chr11	7.22236	9.61621	-2.394	0.00376
Rpgr	chrX	10.4439	12.8372	-2.393	0.00064
Eef2	chr10	14.6695	17.06	-2.391	0.0001
B3galtl	chr5	7.18917	9.579	-2.390	0.00481
Cul9	chr17	8.17931	10.5675	-2.388	0.00078
Hint2	chr4	6.36678	8.75178	-2.385	0.00385
Gna11	chr10	9.23157	11.6164	-2.385	0.00172
Lyrm7	chr11	7.23993	9.62219	-2.382	0.00028
Ficd	chr5	6.69856	9.0796	-2.381	0.01013
Yrdc	chr4	7.54989	9.92975	-2.380	0.00752
Erich1	chr8	6.68971	9.06831	-2.379	0.00612
Grcc10	chr6	7.76468	10.1389	-2.374	0.00824
Ipo5	chr14	12.3876	14.76	-2.372	6.8E-06
Eci1	chr17	7.12775	9.49826	-2.371	0.03275
Serpinb6a	chr13	8.79061	11.1587	-2.368	0.0003
Btg1	chr10	8.59318	10.9593	-2.366	0.00279
Lcp1	chr14	10.5427	12.9082	-2.366	0.00068
Igfbp5	chr1	8.32909	10.6928	-2.364	0.01765
Acaca	chr11	9.54492	11.907	-2.362	0.00298
Tead1	chr7	9.01458	11.3764	-2.362	0.00137
Rnaseh2b	chr14	8.45941	10.8213	-2.362	0.00044
Mageb16	chrX	9.12915	11.4907	-2.362	0.00691
Tor1aip2	chr1	9.20754	11.5681	-2.361	2E-05
Srl	chr16	8.3719	10.7317	-2.360	0.00123
Glul	chr1	9.61386	11.9731	-2.359	0.02182
Tlk1	chr2	11.3666	13.7256	-2.359	5.3E-05
Solh	chr17	6.23619	8.59102	-2.355	0.00437
Ddost	chr4	10.7999	13.1537	-2.354	0.00027
Iqgap1	chr7	10.9793	13.3325	-2.353	0.00016
Tsn	chr1	11.4665	13.8195	-2.353	0.00113
Rbm5	chr9	9.76696	12.1165	-2.350	0.00186
Tmx1	chr12	7.51194	9.85955	-2.348	0.01184
Tfap4	chr16	8.64521	10.9914	-2.346	0.00058
Tnks2	chr19	11.4731	13.8191	-2.346	2.8E-05
Ubr3	chr2	12.0659	14.4064	-2.341	4.7E-05
BC003331	chr1	9.84897	12.1887	-2.340	0.00082
Zc3h10	chr10	7.06598	9.40325	-2.337	0.02114
Rpl27a	chr7	10.4503	12.7869	-2.337	2.6E-05
Wdfy1	chr1	9.19007	11.5265	-2.336	0.0032
Zfp746	chr6	8.7996	11.1355	-2.336	0.0032
Dcun1d3	chr7	9.19302	11.5283	-2.335	0.00035
Tars2	chr3	9.52581	11.8605	-2.335	0.00057

Denr	chr5	10.5226	12.8573	-2.335	0.00272
Atp11b	chr3	11.4494	13.7824	-2.333	3.4E-05
Commd10	chr18	7.74873	10.0802	-2.331	0.00108
Rps6	chr4	11.6373	13.9668	-2.330	1.4E-05
Ankrd24	chr10	6.84761	9.17571	-2.328	0.00221
Wscd2	chr5	7.67319	10.0012	-2.328	0.0046
Tra2a	chr6	10.3303	12.658	-2.328	0.00014
Casc4	chr2	7.89156	10.2165	-2.325	0.00614
Pim3	chr15	6.74309	9.06204	-2.319	0.03666
Rab11fip4	chr11	11.1233	13.4407	-2.317	8.6E-06
Nipa2	chr7	7.6797	9.99478	-2.315	0.00504
Jph1	chr1	7.78588	10.0966	-2.311	0.00952
Inppl1	chr7	10.2045	12.5127	-2.308	0.00274
Snapin	chr3	8.64176	10.9484	-2.307	0.01177
Nudt11	chrX	5.74714	8.05264	-2.305	0.00334
Glo1	chr17	8.40206	10.7069	-2.305	0.00238
Rps19	chr7	10.5501	12.8516	-2.302	0.00249
Neil2	chr14	8.53658	10.8373	-2.301	0.00207
Vcl	chr14	11.4616	13.7621	-2.301	0.00307
Ube2q2	chr9	10.1911	12.4912	-2.300	5.9E-05
Srpr	chr9	10.5415	12.8407	-2.299	2.1E-05
Tulp4	chr17	13.079	15.3777	-2.299	0.00045
Pten	chr19	11.2221	13.5203	-2.298	7.8E-05
Gadd45a	chr6	7.68178	9.97901	-2.297	0.02087
Kdm4b	chr17	9.08202	11.3792	-2.297	0.00283
Lage3	chrX	8.86774	11.1606	-2.293	0.01373
Tbc1d20	chr2	10.1016	12.392	-2.290	9.1E-05
Parg	chr14	11.1183	13.4079	-2.290	7.6E-05
4933433P14Rik	chr12	6.80676	9.09534	-2.289	0.01275
Cxxc5	chr18	7.18475	9.47317	-2.288	0.01194
Slc36a1	chr11	9.2365	11.5239	-2.287	0.00011
Wbp5	chrX	7.87415	10.1606	-2.286	0.00501
Pts	chr9	7.18291	9.46858	-2.286	0.01002
Tle1	chr4	9.2918	11.5756	-2.284	0.00016
Abcc5	chr16	9.4992	11.7805	-2.281	0.00295
Ano1	chr7	6.99786	9.27826	-2.280	0.01218
Thbs4	chr13	9.52148	11.8009	-2.279	0.00562
Sgms2	chr3	9.26959	11.5435	-2.274	0.0003
Ufc1	chr1	9.65368	11.9263	-2.273	0.00019
Dctn6	chr8	9.39162	11.6638	-2.272	0.00368
Ppp1r2	chr16	8.86672	11.1388	-2.272	0.00254
Heatr3	chr8	9.02274	11.2926	-2.270	0.00062
Kdm3b	chr18	10.65	12.9193	-2.269	0.00015
Pcyt2	chr11	6.72722	8.9939	-2.267	0.00095
Rnh1	chr7	7.8881	10.1544	-2.266	0.00622
Dnajc15	chr14	8.61114	10.8772	-2.266	0.00017
Vps8	chr16	11.4167	13.6822	-2.266	0.01733
Adsl	chr15	9.411	11.6758	-2.265	0.00076
Klhl20	chr1	9.07666	11.3406	-2.264	3.8E-05
Tmem181b-ps	chr17	9.78126	12.0446	-2.263	0.00018
Zzef1	chr11	9.91547	12.176	-2.261	2.6E-05
Med16	chr10	8.0085	10.2685	-2.260	0.01346
Mrfap1	chr5	11.6057	13.8633	-2.258	0.00138
4930427A07Rik	chr12	7.56373	9.82119	-2.257	0.00654
Lgr4	chr2	8.94343	11.2	-2.257	0.00395
Cdr2l	chr11	6.71746	8.97103	-2.254	0.00506
Htatsf1	chrX	12.4694	14.7227	-2.253	0.00016

Vamp3	chr4	11.2301	13.4827	-2.253	2.1E-05
Coa5	chr1	10.9836	13.236	-2.252	0.00061
Ccny	chr18	10.0898	12.3418	-2.252	0.00566
Arpc1a	chr5	10.3666	12.6184	-2.252	0.00275
Map3k9	chr12	10.1031	12.3547	-2.252	0.00635
Vma21	chrX	9.77941	12.0292	-2.250	0.00418
Tagap1	chr17	7.17762	9.42659	-2.249	0.01422
Pogz	chr3	11.6246	13.8726	-2.248	0.00079
Dcp1b	chr6	6.89029	9.1371	-2.247	0.01973
Hspa2	chr12	8.21542	10.4618	-2.246	0.00526
Cct4	chr11	11.506	13.7517	-2.246	0.00013
Cetn4	chr3	8.25127	10.4955	-2.244	0.00045
Hadhb	chr5	10.2059	12.4497	-2.244	0.00066
Sorcs3	chr19	9.17003	11.4137	-2.244	0.00041
Cog7	chr7	8.50677	10.75	-2.243	0.01098
Rpl8	chr15	11.4454	13.6852	-2.240	0.00011
Sae1	chr7	8.67681	10.9164	-2.240	0.00237
Cggbp1	chr16	9.82274	12.0592	-2.236	0.00053
Ddx54	chr5	8.90161	11.1358	-2.234	0.00032
Hspd1	chr1	12.693	14.927	-2.234	2.6E-05
Rcor3	chr1	8.05687	10.2897	-2.233	0.00972
Mrpl15	chr1	9.14932	11.3816	-2.232	0.00429
Ist1	chr8	10.2464	12.4759	-2.229	9.8E-06
Igsf9b	chr9	7.11107	9.33898	-2.228	0.00881
Lsm12	chr11	8.95388	11.1815	-2.228	0.00213
Fchsd1	chr18	6.98227	9.20573	-2.223	0.04366
Kif9	chr9	10.0222	12.2429	-2.221	0.00048
1110002N22Rik	chr11	7.45142	9.67187	-2.220	0.0003
Ctdsp2	chr10	10.5532	12.7693	-2.216	1.4E-05
Tbc1d22a	chr15	7.44312	9.65877	-2.216	0.01855
Mll5	chr5	9.98436	12.1992	-2.215	0.00016
4931414P19Rik	chr14	6.27217	8.48648	-2.214	0.03882
Gm14326	chr2	7.72221	9.93249	-2.210	0.00161
Twistnb	chr12	8.82168	11.0317	-2.210	0.0141
Ptma	chr1	13.1285	15.3377	-2.209	0.00194
Tnrc6a	chr7	10.7913	12.9991	-2.208	0.00155
Toe1	chr4	8.31362	10.5196	-2.206	0.01754
Ubtf	chr11	10.6091	12.8148	-2.206	0.00155
Pcx	chr19	9.62436	11.8296	-2.205	5.8E-05
Narfl	chr17	7.43664	9.63986	-2.203	0.01007
1700054N08Rik	chr8	8.9223	11.12	-2.198	0.00099
Dpagt1	chr9	6.70938	8.90536	-2.196	0.04971
Nat2	chr8	7.23922	9.43177	-2.193	0.01875
Mtmr12	chr15	9.00601	11.1985	-2.193	0.00415
Larp1b	chr3	10.5612	12.753	-2.192	0.00128
Rpl23	chr11	12.3554	14.5467	-2.191	0.00429
Tfam	chr10	10.9605	13.151	-2.191	8.3E-05
Ikzf5	chr7	8.31075	10.5012	-2.190	0.0017
Tfcp2l1	chr1	9.01349	11.2039	-2.190	0.00096
AA960436	chr8	6.50229	8.69265	-2.190	0.00346
Mex3d	chr10	8.29599	10.4851	-2.189	0.0008
Agbl3	chr6	6.7683	8.95698	-2.189	0.00611
Zmat3	chr3	8.04176	10.2302	-2.188	0.0169
Tox4	chr14	9.26214	11.4503	-2.188	0.00029
Pask	chr1	9.18025	11.3676	-2.187	0.0014
Rpl18	chr7	11.5877	13.7748	-2.187	0.00546
Eno4	chr19	6.34917	8.53352	-2.184	0.02075

Ttc19	chr11	8.88221	11.066	-2.184	0.00302
Ilkap	chr1	9.27135	11.4541	-2.183	0.00148
Bhlhe41	chr6	7.0492	9.23042	-2.181	0.01517
Stk19	chr17	6.84954	9.02976	-2.180	0.01315
Hnrnpul1	chr7	11.9696	14.1495	-2.180	0.00024
Sdccag8	chr1	7.32276	9.50241	-2.180	0.00332
Ppp3ca	chr3	9.14309	11.3224	-2.179	0.00131
Ell2	chr13	10.5475	12.7257	-2.178	0.00202
Trmt61a	chr12	8.56566	10.741	-2.175	0.01665
Polr3gl	chr3	8.3021	10.4763	-2.174	0.00024
Idh3b	chr2	9.69726	11.8712	-2.174	0.01154
Pip4k2a	chr2	8.30277	10.4761	-2.173	0.00055
Heca	chr10	10.9089	13.0819	-2.173	0.0001
Tceb3	chr4	9.5031	11.6759	-2.173	2.1E-05
Pkd2	chr5	8.35939	10.5313	-2.172	0.00075
Rere	chr4	12.6146	14.7863	-2.172	0.00017
Ivns1abp	chr1	10.5372	12.708	-2.171	3.9E-05
Megf6	chr4	8.39401	10.5646	-2.171	0.00113
Fmr1nb	chrX	7.99636	10.1664	-2.170	0.00104
Rbm34	chr8	8.25039	10.4179	-2.167	0.01673
Mtif2	chr11	9.06399	11.2314	-2.167	0.00016
Zfp512	chr5	10.1744	12.3377	-2.163	0.00012
Bag1	chr4	10.2321	12.3953	-2.163	0.00028
Ubr4	chr4	13.4436	15.606	-2.162	0.00043
Evi5l	chr8	6.73711	8.89882	-2.162	3.7E-05
Pcnxl3	chr19	10.4108	12.5683	-2.158	0.00103
Snip1	chr4	8.76003	10.9173	-2.157	0.00058
Pank4	chr4	6.49305	8.65028	-2.157	0.01351
Prdx6	chr1	8.86398	11.0198	-2.156	0.00217
Gga1	chr15	9.9603	12.1157	-2.155	0.00052
Nup133	chr8	11.6582	13.8127	-2.154	4E-06
Rps28	chr17	6.90528	9.05963	-2.154	0.04547
Wdr62	chr7	10.4615	12.6154	-2.154	0.00412
Arvcf	chr16	9.30094	11.454	-2.153	0.00021
Zfp316	chr5	8.05022	10.2017	-2.151	0.00706
Kif16b	chr2	12.4658	14.617	-2.151	2.9E-05
Ppp1r26	chr2	6.78285	8.93234	-2.149	3.5E-05
Atf7	chr15	9.46165	11.611	-2.149	0.00158
Naa15	chr3	11.1341	13.281	-2.147	0.00019
Rab5b	chr10	10.5577	12.7035	-2.146	0.00169
Bax	chr7	8.56736	10.7128	-2.145	0.00102
Chpt1	chr10	8.24697	10.3923	-2.145	0.00504
Atp6ap2	chrX	10.404	12.5487	-2.145	0.00056
9130401M01Rik	chr15	9.02825	11.1728	-2.145	0.00421
Glrb	chr3	6.66106	8.80452	-2.143	0.00863
Snx19	chr9	9.67788	11.8199	-2.142	4E-05
Dtd1	chr2	8.58032	10.721	-2.141	0.00461
Fam203a	chr15	6.99789	9.13643	-2.139	0.01272
Zfp141	chr7	9.41038	11.5465	-2.136	0.00265
Prkdc	chr16	10.7213	12.8564	-2.135	0.0002
Arhgef5	chr6	6.78284	8.91627	-2.133	0.00123
Rabgap1	chr2	11.7806	13.9139	-2.133	0.00036
Heg1	chr16	7.43012	9.56336	-2.133	0.01416
Tex14	chr11	9.93866	12.0691	-2.130	0.00012
Smad5	chr13	10.0284	12.1554	-2.127	0.00018
Lsm4	chr8	10.7917	12.9182	-2.127	0.00166
Mcart1	chr4	9.94414	12.0691	-2.125	0.00127

Cc2d2a	chr5	7.81869	9.94204	-2.123	0.00353
Nbeal1	chr1	11.6808	13.8034	-2.123	3.2E-05
Map4k3	chr17	7.72457	9.84671	-2.122	0.00502
Uba52	chr8	9.78646	11.9067	-2.120	0.00019
Lars2	chr9	15.1541	17.2744	-2.120	0.01001
Lonrf3	chrX	11.8749	13.9926	-2.118	0.00016
Zfp53	chr17	7.45472	9.57171	-2.117	0.00291
Nol7	chr13	9.78282	11.8986	-2.116	0.01333
Lamp2	chrX	9.58911	11.7044	-2.115	3E-05
Ablim1	chr19	12.2946	14.4077	-2.113	9.5E-06
Zfp238	chr1	12.0259	14.1365	-2.111	0.00019
Vps41	chr13	11.4928	13.6033	-2.111	0.00033
Cct7	chr6	10.9388	13.0489	-2.110	5.5E-06
Pfkfb3	chr2	9.55514	11.661	-2.106	0.00262
Hsp90ab1	chr17	14.9771	17.0811	-2.104	3.8E-06
Casp2	chr6	11.191	13.2916	-2.101	8.4E-05
Trpm7	chr2	11.7455	13.8461	-2.101	0.00086
1300018J18Rik	chr15	6.81565	8.91594	-2.100	0.02338
Macf1	chr4	13.6635	15.7619	-2.098	7.2E-06
Rhebl1	chr15	8.50425	10.5977	-2.093	0.006
Rpl7l1	chr17	9.33836	11.4302	-2.092	1.4E-05
LOC101055917	chrX	8.96327	11.0528	-2.090	0.0005
Urm1	chr2	8.37123	10.4599	-2.089	0.0011
Phkb	chr8	7.93007	10.0186	-2.089	0.00201
Dus1l	chr11	10.0812	12.1687	-2.088	0.00127
Wwtr1	chr3	8.14264	10.2291	-2.086	0.00601
Zfp949	chr9	8.46938	10.5556	-2.086	0.00115
Ndst2	chr14	7.22686	9.31259	-2.086	0.00677
Wdr74	chr19	8.90167	10.9867	-2.085	0.0073
Spata1	chr3	6.70011	8.78462	-2.085	0.00087
Deaf1	chr7	7.14807	9.23101	-2.083	0.03383
Arhgap33	chr7	7.14115	9.22365	-2.083	0.00101
Ddx23	chr15	11.0152	13.0951	-2.080	0.00013
Tsr1	chr11	9.66192	11.7418	-2.080	0.00012
Ubr5	chr15	13.6521	15.7307	-2.079	8E-05
Rangrf	chr11	7.27651	9.35393	-2.077	0.00716
Khdrbs1	chr4	9.53982	11.6168	-2.077	0.00028
Ipo4	chr14	10.4069	12.4786	-2.072	7.4E-05
Ppp5c	chr7	9.71527	11.7867	-2.071	0.00225
Wipf2	chr11	10.5641	12.6349	-2.071	0.00014
Casp9	chr4	8.90398	10.9732	-2.069	1.3E-05
2410006H16Rik	chr11	6.76413	8.83123	-2.067	0.00252
Sco1	chr11	7.49949	9.56451	-2.065	0.04521
Baiap2	chr11	7.88337	9.94738	-2.064	0.01037
Mios	chr6	10.707	12.77	-2.063	0.00112
Aldh5a1	chr13	10.0348	12.0963	-2.062	0.00042
Dlg2	chr7	8.86517	10.926	-2.061	0.00048
Chrna5	chr9	7.04351	9.10392	-2.060	0.03071
Ass1	chr2	7.7937	9.85403	-2.060	1.8E-06
Etv6	chr6	8.24477	10.3049	-2.060	0.03374
Bcs1l	chr1	7.26006	9.31839	-2.058	0.03913
Gm4724	chr2	6.64629	8.7022	-2.056	0.00221
Trim66	chr7	6.39521	8.44778	-2.053	0.00402
Itpr1	chr6	14.4023	16.4543	-2.052	8.1E-05
Ralgapa2	chr2	9.53727	11.589	-2.052	0.00074
Ipo7	chr7	9.86983	11.9212	-2.051	0.00054
Xiap	chrX	10.5771	12.627	-2.050	0.00024

Rpl12	chr2	11.3306	13.3805	-2.050	0.00522
Gm14434	chr2	6.63841	8.68747	-2.049	0.00184
Tuft1	chr3	7.71242	9.76137	-2.049	0.00221
Zfp191	chr18	8.18685	10.2353	-2.048	0.00523
Pfkfb2	chr1	8.68762	10.7355	-2.048	0.01466
Cmtm4	chr8	9.47764	11.5234	-2.046	0.00044
Nbas	chr12	10.9685	13.0108	-2.042	5.7E-06
Ube2c	chr2	11.055	13.0967	-2.042	0.00497
Pum1	chr4	11.0123	13.0512	-2.039	0.00024
H2afz	chr3	8.65483	10.6935	-2.039	0.00678
Spsb3	chr17	7.86714	9.90366	-2.037	0.00894
Trim37	chr11	12.008	14.0436	-2.036	3.5E-05
Dlc1	chr8	9.37812	11.4131	-2.035	0.00143
C8g	chr2	6.77899	8.81198	-2.033	0.02656
Gm14308	chr2	6.64926	8.68146	-2.032	0.00179
Cyp46a1	chr12	7.84962	9.88127	-2.032	0.00104
Kdm1b	chr13	13.9632	15.9943	-2.031	2.6E-05
Ints1	chr5	11.1976	13.2263	-2.029	0.00516
Neurl4	chr11	10.4402	12.4685	-2.028	0.00408
Aimp1	chr3	9.53302	11.5607	-2.028	0.00065
Slc30a6	chr17	6.69312	8.71963	-2.027	0.00838
Shq1	chr6	6.97321	8.99767	-2.024	0.03074
Hnrnpl	chr7	10.8977	12.922	-2.024	0.00069
Ube2v1	chr2	10.4814	12.5039	-2.022	0.0015
Smpd2	chr10	6.7753	8.79767	-2.022	0.01823
Exosc9	chr3	10.6802	12.7023	-2.022	0.0007
Stxbp2	chr8	10.9904	13.0112	-2.021	0.00066
Ufd1l	chr16	9.49953	11.5173	-2.018	0.00027
Gas5	chr1	7.82901	9.84636	-2.017	0.00304
Nme2	chr11	8.13154	10.1485	-2.017	0.00706
Crls1	chr2	8.20298	10.219	-2.016	0.01544
Sesn3	chr9	10.2805	12.2951	-2.015	3.6E-05
Itpa	chr2	7.75234	9.76658	-2.014	0.0028
Ifi30	chr8	7.02357	9.03633	-2.013	0.0078
Atp5f1	chr3	11.2749	13.2865	-2.012	0.00026
Csrp2	chr10	7.90946	9.9182	-2.009	0.00701
Vezt	chr10	6.44644	8.45481	-2.008	0.00643
Bbs7	chr3	8.34781	10.3558	-2.008	0.00091
Rpl22l1	chr3	8.19558	10.2032	-2.008	0.0003
Wdr83	chr8	7.19621	9.20354	-2.007	0.00113
Bre	chr5	8.08291	10.0875	-2.005	0.01055
Wdr67	chr15	7.71959	9.72323	-2.004	0.02134
Sox6	chr7	8.21843	10.2203	-2.002	0.00674
Ubxn6	chr17	9.60905	11.6106	-2.002	0.00705
Tln2	chr9	11.2119	13.2106	-1.999	2.8E-05
Lancl2	chr6	10.2081	12.2059	-1.998	4.2E-05
1110001A16Rik	chr17	7.63814	9.63529	-1.997	0.00288
Eif1ad	chr19	10.6581	12.6547	-1.997	0.00014
Rps6kc1	chr1	9.36284	11.3589	-1.996	0.00151
Tbc1d9b	chr11	9.96337	11.9584	-1.995	0.00022
Tbpl2	chr2	7.53543	9.52933	-1.994	0.00073
Slc25a39	chr11	9.89826	11.892	-1.994	0.00054
Rps26	chr10	8.9151	10.9062	-1.991	0.01322
Insig2	chr1	8.4261	10.4166	-1.990	0.00243
2810004N23Rik	chr8	9.6984	11.6884	-1.990	7.9E-05
Mapk13	chr17	7.01311	9.00184	-1.989	0.03542
Kctd7	chr5	7.88211	9.87019	-1.988	0.04993

Suclg1	chr6	9.47556	11.463	-1.987	0.00562
Adal	chr2	7.86505	9.8522	-1.987	0.00218
2210404J11Rik	chr17	7.52923	9.51584	-1.987	0.03532
Ndufa1	chrX	10.0117	11.9979	-1.986	0.00247
Alkbh6	chr7	7.78662	9.77259	-1.986	0.00827
Pkn1	chr8	8.84221	10.8281	-1.986	2.3E-05
Ogfrl1	chr1	9.0246	11.0095	-1.985	0.01472
Chuk	chr19	9.34247	11.3269	-1.984	0.00846
Erlec1	chr11	9.28035	11.2647	-1.984	0.00324
Rpl6	chr5	11.6361	13.6156	-1.980	7.7E-05
Gls	chr1	9.01945	10.9987	-1.979	0.00115
Med9	chr11	7.72016	9.6992	-1.979	0.00081
Man2c1	chr9	7.85961	9.83843	-1.979	0.00145
Dennd5a	chr7	10.3947	12.3735	-1.979	0.00014
Ccdc150	chr1	6.97703	8.95333	-1.976	0.00572
Mfsd11	chr11	6.84418	8.81997	-1.976	0.009
Tmem165	chr5	8.11577	10.0901	-1.974	0.00353
Polr3e	chr7	9.40011	11.3736	-1.973	0.00264
Pomt2	chr12	8.19512	10.1685	-1.973	0.00338
Rps25	chr9	10.8923	12.8653	-1.973	0.00137
Hk1	chr10	6.28073	8.25216	-1.971	0.04284
Tnfsf10	chr3	6.70192	8.67147	-1.970	0.02547
Ssr1	chr13	11.0302	12.9996	-1.969	0.0002
Cdk9	chr2	8.45737	10.4264	-1.969	0.00117
Bcas2	chr3	10.6398	12.6068	-1.967	0.00314
Gatad1	chr5	8.93715	10.9039	-1.967	0.00775
Asns	chr6	6.91738	8.88379	-1.966	0.00112
Fbxw5	chr2	9.57756	11.5438	-1.966	0.01236
Gdi1	chrX	9.95366	11.9198	-1.966	0.00192
Kif13a	chr13	9.45969	11.4251	-1.965	0.00065
Casc3	chr11	9.95392	11.9187	-1.965	0.00295
Dhps	chr8	7.09801	9.06215	-1.964	0.01292
Pcmtd2	chr2	8.76861	10.7306	-1.962	0.00813
Ssbp1	chr6	10.4698	12.4307	-1.961	0.00403
Adarb1	chr10	8.22987	10.1897	-1.960	0.04139
A730098P11Rik	chr16	8.95226	10.9109	-1.959	0.00063
Luc7l2	chr6	11.5686	13.5257	-1.957	4.3E-05
Slc25a46	chr18	7.54922	9.50472	-1.956	0.01835
Rpl36a	chrX	9.97479	11.9281	-1.953	0.00512
Cnnm4	chr1	9.65544	11.606	-1.951	0.00038
Gm2007	chr2	6.64165	8.59204	-1.950	0.00308
Acvr2b	chr9	8.01588	9.96558	-1.950	0.00434
1810026J23Rik	chr9	9.79331	11.7426	-1.949	0.0062
Ctsd	chr7	10.2578	12.2069	-1.949	3.5E-05
Gm11007	chr2	6.64311	8.59191	-1.949	0.00323
Tmem147	chr7	6.73596	8.68338	-1.947	0.00139
Srp72	chr5	12.2782	14.2254	-1.947	0.00017
Pdcd4	chr19	12.2192	14.1659	-1.947	7.2E-05
Srsf5	chr12	12.2125	14.1586	-1.946	0.00351
Gcc1	chr6	9.51444	11.4578	-1.943	9.3E-05
Cdc42bpg	chr19	8.56244	10.5051	-1.943	0.00101
Dnajc16	chr4	9.2634	11.2054	-1.942	3.2E-06
Exoc6b	chr6	8.30197	10.2421	-1.940	0.02347
Nomo1	chr7	11.0755	13.0155	-1.940	0.00013
Rpl31	chr1	11.6238	13.5601	-1.936	0.00422
Zfp523	chr17	8.18038	10.1158	-1.935	0.00045
Atrip	chr9	8.26556	10.1994	-1.934	0.00037

Zfr2	chr10	7.65206	9.58398	-1.932	0.01306
Nsun6	chr2	7.87524	9.80619	-1.931	0.00937
Rps5	chr7	12.1319	14.0628	-1.931	0.00094
Clec16a	chr16	9.53592	11.4634	-1.927	0.0006
Emc2	chr15	10.2075	12.1348	-1.927	0.00383
Mmd	chr11	8.4384	10.3648	-1.926	0.00281
Gm14391	chr2	7.13276	9.0561	-1.923	0.01213
Gatc	chr5	8.15097	10.0707	-1.920	0.0009
Zpld1	chr16	7.60614	9.52426	-1.918	0.03607
Cap1	chr4	9.88132	11.7994	-1.918	0.0026
Rpl35a	chr16	10.1758	12.0938	-1.918	0.0013
Flna	chrX	9.39968	11.3173	-1.918	0.01497
Zfat	chr15	8.19992	10.1156	-1.916	0.00927
Ythdf1	chr2	11.0806	12.9954	-1.915	8.5E-05
C330006K01Rik	chr5	7.2269	9.14165	-1.915	0.00343
Ddx20	chr3	9.66337	11.5769	-1.914	0.00073
Eif3f	chr7	10.3234	12.2368	-1.913	0.00073
2610002M06Rik	chrX	9.55926	11.4711	-1.912	0.0021
Phf10	chr17	8.91524	10.8267	-1.911	0.00441
Wdr3	chr3	9.20486	11.1139	-1.909	0.00011
Tmem208	chr8	7.08534	8.9943	-1.909	0.0046
Hook3	chr8	10.8705	12.779	-1.909	0.00043
Ppp4r1	chr17	9.91861	11.8259	-1.907	0.0078
Ugcg	chr4	11.1032	13.0102	-1.907	9.8E-05
Kdm3a	chr6	8.52195	10.4289	-1.907	0.01573
Ddx17	chr15	11.9022	13.8088	-1.907	4.4E-05
Cbll1	chr12	10.3982	12.3042	-1.906	0.00178
Ccdc61	chr7	9.87231	11.7776	-1.905	0.00104
Ezr	chr17	9.31566	11.2208	-1.905	0.00119
Ndufa3	chr7	8.64295	10.5466	-1.904	0.00783
Tomm6	chr17	10.235	12.1358	-1.901	0.00794
Usp32	chr11	12.2715	14.1721	-1.901	0.00044
Tnfrsf19	chr14	6.9012	8.80102	-1.900	0.01245
Cables1	chr18	7.18787	9.087	-1.899	0.00055
Ppib	chr9	9.19009	11.0889	-1.899	0.00086
Rps15	chr10	10.4227	12.3209	-1.898	0.00765
Zfp40	chr17	7.3384	9.23649	-1.898	0.03454
Snrk	chr9	9.19063	11.0884	-1.898	0.00302
Yif1b	chr7	7.92479	9.8206	-1.896	0.02302
Tfrc	chr16	9.17939	11.0749	-1.896	0.00463
Arl6ip4	chr5	9.30702	11.2016	-1.895	0.0059
Prdx3	chr19	8.35536	10.249	-1.894	0.01005
D030016E14Rik	chr8	11.1163	13.0086	-1.892	0.00895
Rab40c	chr17	7.69001	9.58165	-1.892	0.00211
Cstf2t	chr19	9.57891	11.4692	-1.890	0.0009
Sik2	chr9	8.72237	10.6106	-1.888	0.00081
Ube3b	chr5	10.2149	12.1028	-1.888	0.00315
Wsb1	chr11	9.79327	11.6771	-1.884	3.5E-05
Fam164a	chr3	8.82816	10.7101	-1.882	0.0039
Hipk3	chr2	10.6202	12.5014	-1.881	0.00019
Bet1l	chr7	7.69568	9.57523	-1.880	0.0187
Btbd7	chr12	9.41782	11.2971	-1.879	0.00011
Polr1b	chr2	9.30624	11.1854	-1.879	0.00014
BC024659	chr13	6.84006	8.71874	-1.879	0.02001
Tep1	chr14	10.2184	12.095	-1.877	0.00045
Adam22	chr5	8.67513	10.5512	-1.876	0.00931
Zfp937	chr2	9.26735	11.1432	-1.876	0.0034

Srsf7	chr17	10.0858	11.9612	-1.875	0.00026
Pisd	chr5	9.37435	11.2495	-1.875	0.00309
Stoml1	chr9	7.00049	8.87478	-1.874	0.03625
Mrpl43	chr19	8.32477	10.1978	-1.873	0.00214
Eml5	chr12	9.08756	10.9602	-1.873	0.00721
Tsga10	chr1	9.9866	11.853	-1.866	6.7E-05
Coq5	chr5	9.28967	11.153	-1.863	0.00067
Dtnb	chr12	10.3701	12.2307	-1.861	0.00155
Iqce	chr5	7.33945	9.2	-1.861	0.04553
Acin1	chr14	11.7009	13.561	-1.860	0.00265
Rpl13	chr8	11.0599	12.9199	-1.860	0.01065
Ascc3	chr10	11.5046	13.3643	-1.860	6.9E-05
Rbmxl1	chr8	10.4756	12.3346	-1.859	0.00101
Trmt10a	chr3	7.51573	9.37454	-1.859	0.01044
Tfg	chr16	10.5231	12.3819	-1.859	0.00054
Scaper	chr9	11.8457	13.7039	-1.858	0.00095
Rfx3	chr19	7.65017	9.5071	-1.857	1.4E-05
Pdk1	chr2	10.9657	12.8196	-1.854	0.00017
Cast	chr13	11.0398	12.893	-1.853	1.7E-05
Eif2ak3	chr6	8.39316	10.2462	-1.853	0.00372
Dbr1	chr9	7.45106	9.30384	-1.853	0.01593
Rrbp1	chr2	12.2164	14.0688	-1.852	0.00095
Rpl14	chr9	12.5106	14.3629	-1.852	0.00369
Aasdhppt	chr9	8.64911	10.5003	-1.851	0.00319
Rps4y2	chr6	9.17671	11.0279	-1.851	0.00106
Zfp39	chr11	7.35825	9.20937	-1.851	0.03666
Gsk3b	chr16	10.788	12.6382	-1.850	0.00045
Tmppe	chr9	8.61257	10.4623	-1.850	0.00652
Cpsf1	chr15	10.6489	12.4969	-1.848	0.00815
Tbck	chr3	10.0927	11.9403	-1.848	7.6E-05
Prcc	chr3	8.74765	10.5946	-1.847	0.01228
Bend3	chr10	8.75093	10.5936	-1.843	0.01386
Rpl15	chr14	12.5314	14.3732	-1.842	0.00477
Cox6c	chr15	12.614	14.4537	-1.840	0.00797
Fam3a	chrX	7.9994	9.83865	-1.839	0.00905
Polh	chr17	7.27325	9.11193	-1.839	0.03802
Cramp1l	chr17	10.5491	12.3872	-1.838	0.00129
Polr3d	chr14	9.37048	11.2083	-1.838	0.00086
Homez	chr14	6.82361	8.66061	-1.837	0.00427
Aplp2	chr9	13.0797	14.9157	-1.836	0.00025
Gm3414	chr5	6.72166	8.55633	-1.835	0.00086
Prmt7	chr8	10.8938	12.727	-1.833	0.0123
Myo9a	chr9	11.8823	13.715	-1.833	0.00197
Plod3	chr5	6.55725	8.38997	-1.833	0.00655
Zeb1	chr18	9.36631	11.1989	-1.833	8.2E-05
Fam174a	chr1	9.2693	11.1012	-1.832	0.00315
Lig1	chr7	11.6126	13.4441	-1.832	0.00012
Tmem110	chr14	7.57292	9.40392	-1.831	0.031
Rnf115	chr3	9.12973	10.9582	-1.828	0.00115
Zfp672	chr11	7.17181	8.99978	-1.828	0.00193
Mga	chr2	12.522	14.3499	-1.828	1.1E-05
Zbtb32	chr7	7.64678	9.47335	-1.827	0.00473
Gmfb	chr14	11.2025	13.0286	-1.826	0.00158
Bdp1	chr13	11.1357	12.9613	-1.826	0.00281
Tns3	chr11	7.83583	9.66082	-1.825	0.02377
Gm364	chrX	11.0997	12.9237	-1.824	0.00161
Zfp273	chr13	7.02851	8.85156	-1.823	0.00552

Mdc1	chr17	11.967	13.7894	-1.822	1.9E-05
Crtc2	chr3	7.64961	9.47044	-1.821	0.01573
6230416C02Rik	chr2	6.64017	8.45897	-1.819	0.0082
Nid1	chr13	7.7268	9.5435	-1.817	0.01666
Vat1	chr11	11.0596	12.8751	-1.816	0.00793
Stx7	chr10	9.64405	11.459	-1.815	0.00451
Arap1	chr7	8.37616	10.1909	-1.815	0.01183
Wee1	chr7	9.4643	11.2782	-1.814	0.01141
2210016F16Rik	chr13	9.30874	11.1202	-1.811	7.7E-05
Mars	chr10	9.73393	11.5442	-1.810	0.00194
C630016N16Rik	chr7	6.91421	8.724	-1.810	0.00839
Ppp2r1b	chr9	10.4047	12.214	-1.809	0.00013
Map3k1	chr13	9.14342	10.9528	-1.809	0.00074
Amigo1	chr3	7.71655	9.52343	-1.807	0.00202
Adad2	chr8	7.99074	9.79591	-1.805	0.00052
Gpsm2	chr3	10.3522	12.1573	-1.805	0.00105
Hhat	chr1	7.02243	8.82302	-1.801	0.00414
Pomt1	chr2	8.77884	10.5775	-1.799	0.02097
Agl	chr3	10.8224	12.6204	-1.798	0.00028
Qrich1	chr9	12.2113	14.0089	-1.798	0.00201
2310044G17Rik	chr12	9.71389	11.5113	-1.797	0.01184
Diablo	chr5	10.5781	12.3753	-1.797	7.7E-05
Foxp1	chr6	8.94084	10.7376	-1.797	0.00043
Mpp6	chr6	11.4352	13.228	-1.793	0.00195
Gabra3	chrX	10.3156	12.1081	-1.793	0.00197
Lmnb2	chr10	10.0541	11.8459	-1.792	0.00036
Dhx16	chr17	10.6579	12.447	-1.789	0.01019
2700097O09Rik	chr12	7.00596	8.79343	-1.787	0.01371
Pign	chr1	9.90858	11.6953	-1.787	0.00267
Cbfb	chr8	8.12136	9.90808	-1.787	0.00698
Mknk2	chr10	8.97311	10.7582	-1.785	0.00809
Lpp	chr16	10.3575	12.1389	-1.781	0.01264
Fn1	chr1	9.19582	10.9771	-1.781	0.00391
Phf20l1	chr15	9.73751	11.5178	-1.780	0.00063
Tcta	chr9	7.20882	8.98905	-1.780	0.00202
Tmbim4	chr10	8.40501	10.1852	-1.780	0.00478
Rps24	chr14	11.3402	13.1186	-1.778	0.00503
D5Ertd579e	chr5	11.0247	12.8021	-1.777	6.5E-05
Ttn	chr2	13.616	15.3915	-1.775	0.00015
Adck5	chr15	7.19916	8.97457	-1.775	0.00986
Aqr	chr2	11.8432	13.6178	-1.775	2.5E-05
Ndufs1	chr1	10.641	12.4155	-1.775	0.00039
Tex261	chr6	8.62817	10.4014	-1.773	0.02218
Kansl2	chr15	9.4767	11.2495	-1.773	0.00045
Ptprd	chr4	8.72773	10.4997	-1.772	0.04313
Sod2	chr17	10.3517	12.122	-1.770	0.00087
Cdk5rap3	chr11	9.53915	11.3088	-1.770	0.0001
Pi4ka	chr16	12.4984	14.2672	-1.769	0.00556
Rpl10a	chr17	11.5422	13.3107	-1.768	0.002
Rps20	chr4	9.25504	11.0215	-1.766	0.008
Bccip	chr7	9.28153	11.0439	-1.762	0.00536
Speer4a	chr5	8.34507	10.1066	-1.762	0.01105
Guf1	chr5	8.83973	10.6009	-1.761	0.00057
Kctd9	chr14	9.54797	11.3091	-1.761	4.9E-05
Rc3h1	chr1	12.0814	13.8385	-1.757	0.0001
Pdk3	chrX	10.1746	11.9312	-1.757	0.0003
Taf1d	chr9	10.427	12.1834	-1.756	0.00401

Ogdhl	chr14	9.91619	11.6705	-1.754	0.00016
Cyb561	chr11	8.63769	10.391	-1.753	0.03071
Rfk	chr19	12.103	13.8556	-1.753	9.6E-05
Gm20604	chr12	7.63179	9.38367	-1.752	0.00684
Kif3a	chr11	11.9713	13.7203	-1.749	9.9E-05
Ube2f	chr1	9.53505	11.2821	-1.747	0.00106
Atl3	chr19	12.1441	13.8898	-1.746	0.00015
Tbpl1	chr10	8.85039	10.5942	-1.744	0.00426
Pkd1	chr17	9.6298	11.3736	-1.744	0.00185
Zbtb20	chr16	8.85359	10.5971	-1.744	0.00266
Fan1	chr7	7.10365	8.84676	-1.743	0.00668
Hbp1	chr12	11.6378	13.3807	-1.743	0.00617
Rtn4	chr11	10.5026	12.243	-1.740	0.00732
Ctc1	chr11	9.20018	10.9402	-1.740	0.00655
Mtap1s	chr8	9.30922	11.0487	-1.740	0.01921
Cul3	chr1	12.7916	14.5281	-1.737	0.00108
Scamp1	chr13	12.3136	14.0489	-1.735	2.8E-05
Ssr2	chr3	9.63231	11.3668	-1.735	0.00012
Mettl1	chr10	7.80521	9.53901	-1.734	0.00291
Srp68	chr11	11.5677	13.3009	-1.733	9.3E-05
Pole	chr5	11.8242	13.5566	-1.732	0.00056
Zfp874a	chr13	7.19778	8.92999	-1.732	0.02038
Cinp	chr12	8.68704	10.4187	-1.732	0.00016
Bcdin3d	chr15	7.23414	8.96522	-1.731	0.00605
Ndufs6	chr13	10.2807	12.0101	-1.729	0.00764
Gnb2l1	chr11	12.2476	13.9764	-1.729	0.00123
LOC100503946	chr1	14.7922	16.5205	-1.728	0.0014
Hps1	chr19	7.4341	9.16217	-1.728	0.01264
Psap	chr10	12.1577	13.8844	-1.727	0.00239
Nagk	chr6	7.05226	8.77865	-1.726	0.01769
Sdk1	chr5	9.12133	10.8475	-1.726	0.00199
Parp2	chr14	10.0881	11.8136	-1.726	0.00028
Ndufc1	chr3	8.32366	10.0489	-1.725	0.02102
Ndst1	chr18	9.15417	10.879	-1.725	0.00236
Dhx15	chr5	12.0493	13.7739	-1.725	1.9E-05
Zfp667	chr7	7.70022	9.42468	-1.724	0.00631
Phf21a	chr2	9.60074	11.3249	-1.724	0.00777
Plcg2	chr8	11.3972	13.1194	-1.722	0.00105
Hist1h2bg	chr13	7.21204	8.93085	-1.719	0.00457
Fdps	chr3	9.66432	11.3821	-1.718	0.00309
Ddrgk1	chr2	10.5485	12.2656	-1.717	0.00095
Hdac4	chr1	9.23971	10.9566	-1.717	0.00394
Nsun5	chr5	8.45245	10.1692	-1.717	0.00073
Alyref	chr11	9.58864	11.3028	-1.714	0.0052
Intu	chr3	6.77375	8.48719	-1.713	0.00851
Plcxd2	chr16	7.89856	9.61133	-1.713	0.00882
Cct2	chr10	12.9881	14.6996	-1.711	0.00074
Spg21	chr9	9.93209	11.6419	-1.710	0.00706
Rabggta	chr14	9.19959	10.908	-1.708	0.00028
Gm6747	chr19	10.3448	12.053	-1.708	0.0181
Myo1b	chr1	12.4383	14.1452	-1.707	0.0001
Ift74	chr4	9.7313	11.4376	-1.706	0.00183
Rnf138	chr18	9.34403	11.0501	-1.706	0.00154
Phgdh	chr3	10.6354	12.3413	-1.706	0.00885
Slc39a7	chr17	9.16345	10.8689	-1.705	0.02595
Lasp1	chr11	7.64825	9.35259	-1.704	0.03586
Idh3g	chrX	10.7532	12.4574	-1.704	0.00096

Xlr4a	chrX	10.2373	11.9411	-1.704	0.00206
Lap3	chr5	8.69277	10.3964	-1.704	0.01927
Dbnl	chr11	9.62277	11.3262	-1.703	3.6E-05
Ubtd2	chr11	7.23816	8.94151	-1.703	0.00801
Nktr	chr9	11.1248	12.8272	-1.702	0.00017
Abcd3	chr3	11.0028	12.7034	-1.701	0.00018
Rpl29	chr9	9.73828	11.4382	-1.700	0.00142
Nipal3	chr4	7.86986	9.56969	-1.700	0.00337
Zfp397	chr18	9.07398	10.7738	-1.700	0.02714
Rpl11	chr4	13.5078	15.2066	-1.699	0.00414
Gas2l1	chr11	8.18137	9.87759	-1.696	0.00302
Mafk	chr5	7.99608	9.69159	-1.696	0.03134
Moap1	chr12	7.55543	9.2496	-1.694	0.00855
Rpn2	chr2	11.8382	13.5316	-1.693	5.4E-05
4931428F04Rik	chr8	7.47836	9.17168	-1.693	0.01381
Elavl1	chr8	10.7435	12.4368	-1.693	0.00266
Bex4	chrX	8.25813	9.95136	-1.693	0.02419
Gorab	chr1	7.11296	8.80518	-1.692	0.00494
Tarsl2	chr7	8.14142	9.83287	-1.691	0.00329
Coro1c	chr5	12.1395	13.8306	-1.691	8.7E-05
Rbm45	chr2	8.55999	10.2504	-1.690	0.01923
Spc24	chr9	11.1599	12.8472	-1.687	0.00256
Rps27a	chr11	10.0401	11.7268	-1.687	0.00277
Bop1	chr15	9.69435	11.3809	-1.687	0.00255
Acadvl	chr11	9.11869	10.805	-1.686	0.0052
Eef1a1	chr9	15.3424	17.0277	-1.685	4.2E-05
Ccdc163	chr4	7.28314	8.96812	-1.685	0.04456
Msto1	chr3	7.70395	9.38808	-1.684	0.04742
2410015M20Rik	chr17	7.24065	8.92331	-1.683	0.00682
Ankrd52	chr10	10.9091	12.591	-1.682	0.0004
Fbxo22	chr9	9.82945	11.5104	-1.681	0.00186
Abtb2	chr2	9.21498	10.8926	-1.678	0.001
Supt16h	chr14	13.9091	15.5863	-1.677	1.4E-05
Ndufb9	chr15	11.0381	12.7122	-1.674	0.004
Wbp1	chr6	7.59332	9.26682	-1.673	0.00206
Ubxn8	chr8	9.47294	11.1452	-1.672	0.00249
Zfp516	chr18	9.88488	11.5569	-1.672	0.00056
Slc25a20	chr9	8.82557	10.4975	-1.672	0.0028
Pdgfa	chr5	7.63624	9.3056	-1.669	0.01071
Gm19361	chr9	8.91178	10.5799	-1.668	0.01189
Abi3bp	chr16	10.38	12.0478	-1.668	0.00172
Myo7a	chr7	10.114	11.7803	-1.666	0.00046
Krtcap2	chr3	7.98674	9.65155	-1.665	0.02632
Morf4l1	chr9	12.3189	13.9834	-1.665	2.3E-05
Sde2	chr1	9.91869	11.5816	-1.663	0.00186
Hace1	chr10	9.32486	10.9861	-1.661	0.00355
Ggps1	chr13	8.64964	10.3089	-1.659	0.01448
Mta1	chr12	12.3685	14.0276	-1.659	0.00016
Map3k7	chr4	9.62721	11.2855	-1.658	0.00223
Plcg1	chr2	10.8497	12.5065	-1.657	0.00035
Armc1	chr3	11.6877	13.343	-1.655	0.0021
Fam114a2	chr11	10.8224	12.4761	-1.654	0.00359
Mael	chr1	10.8088	12.4621	-1.653	6.9E-05
Xdh	chr17	11.4154	13.0678	-1.652	0.00212
Azi2	chr9	9.48633	11.1384	-1.652	0.00257
Tmem206	chr1	8.39403	10.0458	-1.652	0.00096
Sacm1l	chr9	10.3405	11.9921	-1.652	0.0038

Rbck1	chr2	9.12542	10.7768	-1.651	0.00423
Acp2	chr2	6.73998	8.38822	-1.648	0.01457
Stat1	chr1	8.2106	9.85729	-1.647	0.03757
Ppp1cc	chr5	11.1649	12.8108	-1.646	6.1E-05
Dvl1	chr4	8.52997	10.1749	-1.645	0.00147
Znhit3	chr11	7.53913	9.18405	-1.645	0.02277
Cpsf6	chr10	10.7473	12.3913	-1.644	0.00099
Xpc	chr6	11.2781	12.9217	-1.644	0.00051
Depdc5	chr5	10.1921	11.8337	-1.642	0.00697
Polq	chr16	9.48481	11.1262	-1.641	0.00021
Gnl1	chr17	10.8157	12.4559	-1.640	0.00108
Ppp6c	chr2	8.83201	10.4721	-1.640	0.00958
Dcaf4	chr12	9.50277	11.1416	-1.639	0.01717
Amacr	chr15	7.34133	8.98004	-1.639	0.02978
Galnt2	chr8	9.85944	11.4977	-1.638	0.00204
Abcb1a	chr5	8.86162	10.4993	-1.638	0.00465
Dctpp1	chr7	9.71989	11.3574	-1.638	0.01032
Edem3	chr1	11.1607	12.7957	-1.635	0.00058
Sox13	chr1	7.39831	9.03327	-1.635	0.01463
Dnaaf2	chr12	9.60323	11.2359	-1.633	0.02002
Kbtbd11	chr8	8.10903	9.73823	-1.629	0.00155
Rpl26	chr11	11.256	12.8848	-1.629	0.00412
Rps21	chr2	10.4513	12.0791	-1.628	0.01129
Vcp	chr4	13.5145	15.1415	-1.627	1.2E-05
Dock6	chr9	9.87688	11.5023	-1.625	0.00478
Ndufv1	chr19	12.4928	14.1173	-1.625	0.00028
Dpp7	chr2	7.6082	9.23031	-1.622	0.00599
Orai3	chr7	7.28087	8.90204	-1.621	0.00522
Nfatc2ip	chr7	9.82758	11.4476	-1.620	0.0068
Ostc	chr3	8.4263	10.0455	-1.619	0.00074
Trmt2b	chrX	7.56607	9.18486	-1.619	0.04336
Rpl10	chrX	11.5724	13.1912	-1.619	0.00117
Nr2c2	chr6	9.5376	11.1547	-1.617	0.00467
Mif	chr10	8.78257	10.3997	-1.617	0.01031
Ccdc73	chr2	11.2385	12.8551	-1.617	0.00185
Klhl12	chr1	11.5461	13.1626	-1.616	0.00315
Hat1	chr2	11.581	13.1974	-1.616	0.00438
Gm14305	chr2	7.1637	8.77766	-1.614	0.0468
Wash	chr17	8.62808	10.2419	-1.614	0.00908
Ptar1	chr19	7.38508	8.99882	-1.614	0.04217
Mcm8	chr2	10.6501	12.2634	-1.613	0.00095
Zfp248	chr6	8.55945	10.1725	-1.613	0.00894
Zfp62	chr11	9.47152	11.0841	-1.613	0.00849
Wdsub1	chr2	9.08176	10.694	-1.612	0.00073
Xrn1	chr9	11.0256	12.6371	-1.612	0.00207
2810408M09Rik	chr2	8.67461	10.2859	-1.611	0.01669
Nkap	chrX	9.17048	10.7811	-1.611	0.00357
2310079F23Rik	chr5	7.5874	9.19762	-1.610	0.01317
Pgk1	chrX	11.0516	12.6611	-1.610	7.3E-05
Dph5	chr3	7.32133	8.92933	-1.608	0.02689
Plxnd1	chr6	7.92043	9.52808	-1.608	0.01329
Mrps26	chr2	8.82218	10.4287	-1.606	0.0294
Smek1	chr12	11.2094	12.8153	-1.606	5.6E-05
Ankfy1	chr11	11.6023	13.208	-1.606	0.00026
Rps10	chr17	11.257	12.8619	-1.605	0.00424
Cep19	chr16	7.8016	9.40584	-1.604	0.01269
Cdc23	chr18	9.77562	11.3783	-1.603	0.00927

Pip4k2b	chr11	9.29883	10.9009	-1.602	0.0047
Ap3s1	chr18	10.2861	11.8874	-1.601	0.00047
Fam168b	chr1	11.34	12.9411	-1.601	4.4E-05
Tdrd7	chr4	10.1128	11.7133	-1.601	0.01297
Hnrnpa0	chr13	9.69389	11.2939	-1.600	0.00042
Taf1	chrX	11.9663	13.5626	-1.596	0.00054
Prkcd	chr14	9.41388	11.01	-1.596	0.00343
0610009O20Rik	chr18	8.03158	9.62707	-1.595	0.0062
Mapk7	chr11	7.75307	9.34784	-1.595	0.01038
Drosha	chr15	13.1107	14.7051	-1.594	2.4E-05
Helz	chr11	11.5455	13.1398	-1.594	0.00121
Pias2	chr18	11.9957	13.5893	-1.594	0.0003
Eprs	chr1	13.4151	15.0087	-1.594	0.00339
Snhg1	chr19	8.09176	9.6853	-1.594	0.01882
Srrt	chr5	11.5377	13.1302	-1.592	0.00103
Fgfr1op	chr17	9.00363	10.5959	-1.592	0.00054
Ppp6r2	chr15	9.30494	10.8968	-1.592	0.00357
Nek8	chr11	8.61327	10.2038	-1.591	0.01719
Psmb5	chr14	10.6794	12.2686	-1.589	0.01501
Rpl4	chr9	13.8144	15.4026	-1.588	2.9E-06
Pprc1	chr19	10.0317	11.6183	-1.587	0.00161
BC003266	chr4	8.37968	9.96401	-1.584	0.00236
Zbtb10	chr3	12.4319	14.0155	-1.584	0.00034
Cox4i1	chr8	12.4153	13.9989	-1.584	0.00046
Dctn1	chr6	11.5719	13.1547	-1.583	0.00026
Gm4636	chr7	7.69353	9.27631	-1.583	0.02071
Nbeal2	chr9	8.43061	10.0121	-1.582	0.0319
Bnip3	chr7	9.43938	11.0209	-1.581	2.7E-05
Th	chr7	9.95505	11.5356	-1.581	0.0036
Pnpt1	chr11	8.98652	10.567	-1.580	0.00059
Cse1l	chr2	11.6924	13.2715	-1.579	3.3E-05
Clk4	chr11	8.59458	10.1737	-1.579	0.00434
Haus7	chrX	10.1616	11.7406	-1.579	0.00116
Ptbp2	chr3	13.6417	15.2204	-1.579	0.00204
Glg1	chr8	10.8491	12.4272	-1.578	0.0033
Tbk1	chr10	10.7014	12.279	-1.578	0.00045
Plekhh1	chr12	9.92793	11.5042	-1.576	0.00591
Uqcrb	chr13	10.4399	12.0145	-1.575	0.03665
Sbf1	chr15	11.1366	12.7081	-1.571	0.00273
Ncoa1	chr12	11.3222	12.8935	-1.571	0.00063
Acox1	chr11	10.0453	11.6159	-1.571	0.00096
Wwp1	chr4	10.6821	12.2525	-1.570	2.3E-06
Nacc2	chr2	10.7698	12.3399	-1.570	0.00204
Sec23b	chr2	8.80745	10.3773	-1.570	0.00424
Tyw3	chr3	7.43832	9.00782	-1.570	0.03002
Gnb1	chr4	11.661	13.2304	-1.569	0.00011
Abcd1	chrX	9.14814	10.717	-1.569	0.00189
Senp3	chr11	11.1502	12.7184	-1.568	0.0014
Atxn3	chr12	10.4366	12.0042	-1.568	0.00592
Pcgf3	chr5	11.0462	12.6103	-1.564	0.00257
Sqstm1	chr11	8.87646	10.4398	-1.563	0.0034
Acsl4	chrX	10.5944	12.1569	-1.562	0.00018
Exoc7	chr11	9.22687	10.7893	-1.562	0.00831
Mybbp1a	chr11	11.1882	12.7505	-1.562	0.00048
Mfn2	chr4	11.0046	12.5659	-1.561	0.00272
Opa3	chr7	8.44318	10.0024	-1.559	0.01943
Taf2	chr15	10.7507	12.3081	-1.557	0.00337

Adat1	chr8	9.06215	10.6182	-1.556	0.00083
Oraov1	chr7	8.22004	9.77101	-1.551	0.00379
Ccdc66	chr14	10.664	12.2147	-1.551	0.00073
Gm5567	chr6	9.17974	10.7302	-1.550	0.00021
Wdr90	chr17	8.20767	9.75788	-1.550	0.0088
Xrcc4	chr13	9.5646	11.1134	-1.549	0.00134
Tstd2	chr4	8.34064	9.8889	-1.548	0.00167
Chd1	chr17	10.9216	12.4682	-1.547	0.00018
Ric8b	chr10	7.63768	9.18253	-1.545	0.01694
Qsox2	chr2	7.44301	8.9878	-1.545	0.01253
Spen	chr4	11.353	12.897	-1.544	2.6E-05
Pelo	chr13	7.821	9.36379	-1.543	0.00298
Cep350	chr1	11.8394	13.3811	-1.542	0.00079
Krit1	chr5	9.62538	11.167	-1.542	0.02123
Rprd1a	chr18	11.6554	13.1945	-1.539	0.00058
Aff4	chr11	10.0737	11.6104	-1.537	0.00048
Ate1	chr7	10.5048	12.0411	-1.536	0.01456
Stxbp3a	chr3	9.82601	11.3603	-1.534	0.00282
Zdhhc4	chr5	8.03044	9.56464	-1.534	0.02718
Arfrp1	chr2	8.38391	9.9171	-1.533	7.8E-05
Rsf1	chr7	10.7326	12.2641	-1.531	7.2E-06
Zfp414	chr17	8.47065	10.0018	-1.531	0.01068
Mrpl49	chr19	7.70229	9.22979	-1.527	0.0159
Zmym3	chrX	10.1846	11.7092	-1.525	0.00014
Anks3	chr16	7.56879	9.09322	-1.524	0.02604
Taf4b	chr18	10.206	11.7303	-1.524	0.00112
Ginm1	chr10	10.7629	12.2857	-1.523	0.00025
Itga1	chr13	7.43666	8.95918	-1.523	0.03169
Rcbtb2	chr14	9.88701	11.4084	-1.521	0.0012
Ppif	chr14	7.67126	9.19121	-1.520	0.03014
Kit	chr5	14.3345	15.8544	-1.520	0.0004
Ncaph2	chr15	11.2978	12.8164	-1.519	0.00034
Ccdc22	chrX	7.59052	9.10502	-1.514	0.00628
Vapa	chr17	11.0629	12.5756	-1.513	0.00177
Gm14325	chr2	8.01584	9.5272	-1.511	0.00587
Mat2a	chr6	12.0366	13.5434	-1.507	0.00102
Coq7	chr7	9.45077	10.9574	-1.507	0.00102
Dsp	chr13	9.83304	11.3395	-1.506	0.01846
Sdhb	chr4	10.6623	12.1683	-1.506	0.00317
Aktip	chr8	10.3994	11.9049	-1.506	0.00037
Csnk1d	chr11	12.3691	13.8732	-1.504	0.0004
Mbd2	chr18	8.89144	10.3937	-1.502	0.00459
Iffo2	chr4	9.16143	10.6628	-1.501	0.00093
Snx1	chr9	9.88649	11.3872	-1.501	0.00832
Plbd2	chr5	9.71067	11.2111	-1.500	0.02584
Sympk	chr7	12.3277	13.8276	-1.500	0.00436
Rasal2	chr1	10.601	12.099	-1.498	0.00773
Twsg1	chr17	8.84512	10.3428	-1.498	0.01722
Cobra1	chr2	9.17911	10.6765	-1.497	0.03191
Harbi1	chr2	8.48252	9.97996	-1.497	0.01544
Rbm8a	chr3	11.2756	12.7673	-1.492	0.00927
Esp38	chr17	20.8139	22.3043	-1.490	0.00191
Tcp1	chr17	13.6923	15.1819	-1.490	0.0003
Park7	chr4	11.9018	13.3912	-1.489	0.0018
Rps18	chr17	11.795	13.2839	-1.489	0.00656
Mcoln1	chr8	10.9736	12.4598	-1.486	6.2E-05
Dhx34	chr7	8.8453	10.3287	-1.483	0.00966

Ints6	chr14	12.0578	13.5392	-1.481	0.00218
Dnttip1	chr2	9.46423	10.9456	-1.481	0.00015
Copa	chr1	13.414	14.8948	-1.481	0.00021
Gm11985	chr11	9.4824	10.9627	-1.480	0.00807
Csde1	chr3	15.2568	16.7358	-1.479	0.00094
Hnrpll	chr17	9.84081	11.3196	-1.479	0.00254
Ntmt1	chr2	8.79748	10.2757	-1.478	0.00354
Th1l	chr2	10.9402	12.4161	-1.476	0.00141
Ankmy2	chr12	7.41757	8.89316	-1.476	0.03108
2410076I21Rik	chr9	8.79974	10.2742	-1.474	0.00351
Hmgb3	chrX	11.4999	12.973	-1.473	0.00206
Ncor2	chr5	10.2099	11.6822	-1.472	0.00155
Cnot1	chr8	14.7801	16.2509	-1.471	0.00239
Mnt	chr11	9.87197	11.3426	-1.471	0.01006
Fam92a	chr4	8.54908	10.0193	-1.470	0.00314
Tbc1d7	chr13	8.66774	10.1367	-1.469	0.00146
Patl1	chr19	11.552	13.0194	-1.467	0.00032
Synj2bp	chr12	10.5585	12.0255	-1.467	0.00524
Slc5a6	chr5	8.89273	10.3594	-1.467	0.0002
Anks1	chr17	9.78468	11.2502	-1.466	0.00397
Klc2	chr19	10.3167	11.782	-1.465	0.00061
Cdkn1c	chr7	9.42973	10.8948	-1.465	0.00018
Ofd1	chrX	12.8485	14.3126	-1.464	3.2E-05
Srsf3	chr17	12.3811	13.8437	-1.463	5.8E-05
St7l	chr3	7.85534	9.3166	-1.461	0.01283
Nubpl	chr12	8.72717	10.1883	-1.461	0.0303
Smap1	chr1	12.3511	13.8114	-1.460	0.0024
Tbrg4	chr11	9.38061	10.8401	-1.459	0.00114
Wdr24	chr17	9.12079	10.5792	-1.458	0.01693
Pphln1	chr15	12.2241	13.6809	-1.457	0.00037
Zfp623	chr15	8.03748	9.49376	-1.456	0.01288
Pcsk7	chr9	8.3487	9.80422	-1.456	0.00131
Gss	chr2	7.89222	9.3477	-1.455	0.00914
Rabgef1	chr5	8.78424	10.2397	-1.455	0.0001
Pigs	chr11	10.4318	11.8861	-1.454	0.01026
Brpf1	chr6	9.54766	11.0009	-1.453	0.00091
Agap1	chr1	12.6785	14.13	-1.452	0.00052
Map4k2	chr19	8.57287	10.0241	-1.451	0.00071
Rpl23a	chr11	11.259	12.7092	-1.450	0.0046
Zfp809	chr9	7.58862	9.03766	-1.449	0.03869
Letmd1	chr15	8.17116	9.62012	-1.449	0.00122
Snrnp200	chr2	14.0302	15.4771	-1.447	0.00021
Arid4b	chr13	10.7544	12.2013	-1.447	0.00238
Zbtb7a	chr10	8.34974	9.7959	-1.446	0.01313
Tmtc4	chr14	11.3463	12.7924	-1.446	0.00066
Ndufa12	chr10	10.7417	12.1859	-1.444	0.01411
Chmp2a	chr7	11.618	13.0616	-1.444	0.01485
Dusp16	chr6	11.2912	12.734	-1.443	0.00121
Eif2s3x	chrX	13.0978	14.5401	-1.442	4.6E-07
Cbr4	chr8	8.14412	9.58612	-1.442	0.04915
Epn1	chr7	10.9323	12.3715	-1.439	0.01003
Rps9	chr7	11.878	13.317	-1.439	0.0195
Lsm1	chr8	10.2105	11.6489	-1.438	0.00716
Tatdn2	chr6	10.1676	11.6058	-1.438	0.01082
Fibp	chr19	9.06437	10.5021	-1.438	0.00795
Clcn7	chr17	8.87372	10.3113	-1.438	0.01036
Qdpr	chr5	10.7449	12.182	-1.437	0.0019

Zfp787	chr7	9.19863	10.6349	-1.436	0.00771
Rrp36	chr17	7.79283	9.22812	-1.435	0.00313
Gfod2	chr8	7.77591	9.21062	-1.435	0.00824
Fam58b	chr11	8.22556	9.65873	-1.433	0.00292
2610301G19Rik	chr14	9.27856	10.7114	-1.433	0.01151
Egln2	chr7	10.8532	12.2851	-1.432	0.00093
Ogt	chrX	12.0299	13.461	-1.431	0.00109
Sp2	chr11	8.88314	10.3138	-1.431	0.00351
2810021J22Rik	chr11	8.19022	9.62016	-1.430	0.02823
Mob1b	chr5	10.4786	11.9066	-1.428	0.00054
Ube2z	chr11	11.5514	12.9782	-1.427	0.00468
2900010M23Rik	chr17	9.17019	10.5963	-1.426	0.01895
Zfp329	chr7	9.45149	10.8772	-1.426	0.00119
Bach1	chr16	9.30817	10.7336	-1.425	0.00518
Zfp101	chr17	7.55313	8.97794	-1.425	0.00025
Lrrc58	chr16	11.1054	12.5297	-1.424	0.00099
Psmd2	chr16	13.6021	15.0257	-1.424	0.00464
Amot	chrX	10.3656	11.7889	-1.423	0.00431
Nop14	chr5	11.5888	13.0112	-1.422	0.0035
Pls3	chrX	8.18067	9.60161	-1.421	0.01005
Eaf1	chr14	9.54267	10.9626	-1.420	0.01172
Itm2b	chr14	11.8251	13.2446	-1.419	0.00067
Habp4	chr13	8.93389	10.3522	-1.418	0.00896
Ap4s1	chr12	7.24106	8.65923	-1.418	0.02171
Zcchc7	chr4	11.6099	13.0278	-1.418	0.00056
Eif3a	chr19	13.3316	14.7488	-1.417	0.00044
Sass6	chr3	9.00112	10.4165	-1.415	0.01852
Ndufa5	chr6	9.3867	10.8016	-1.415	0.03022
Ndufa7	chr17	6.9501	8.36475	-1.415	0.01405
Stat3	chr11	12.983	14.3971	-1.414	0.00427
Ect2	chr3	12.9642	14.3778	-1.414	0.00033
Zfp113	chr5	9.04166	10.4553	-1.414	0.00033
Cpped1	chr16	8.10342	9.51702	-1.414	0.03485
Ttc39b	chr4	10.5313	11.9444	-1.413	0.00138
Wdr59	chr8	9.30177	10.7139	-1.412	0.00445
Tma7	chr9	9.54643	10.9585	-1.412	0.0441
Rpl38	chr11	10.1148	11.5266	-1.412	0.03026
Rmnd1	chr10	9.59144	11.0026	-1.411	0.01681
Ddx5	chr11	13.9753	15.3863	-1.411	0.00338
Lrba	chr3	11.8085	13.2188	-1.410	0.00422
A230046K03Rik	chr10	10.4333	11.8412	-1.408	0.00463
Adrbk1	chr19	8.54697	9.95464	-1.408	0.00613
Dync2h1	chr9	10.4232	11.8308	-1.408	0.0046
2010011I20Rik	chr2	9.64565	11.0527	-1.407	0.0054
Fbxw11	chr11	11.7486	13.155	-1.406	0.00091
Reep6	chr10	9.06981	10.4762	-1.406	0.00081
Stard4	chr18	9.87162	11.2778	-1.406	0.0204
Rpl13a	chr7	11.92	13.3252	-1.405	0.00226
Exosc2	chr2	8.81268	10.2178	-1.405	0.00119
Cct8	chr16	13.0875	14.4924	-1.405	0.00091
Klhl15	chrX	10.9606	12.3636	-1.403	0.00389
Rnpepl1	chr1	8.01649	9.41884	-1.402	0.00239
Zbtb17	chr4	9.46935	10.8693	-1.400	0.0048
Mesdc2	chr7	8.8577	10.2565	-1.399	0.00451
41889	chr9	12.2241	13.6211	-1.397	0.00046
Acsf3	chr8	8.89052	10.2872	-1.397	0.01958
1300001I01Rik	chr11	10.5493	11.9427	-1.393	0.00679

Ddx21	chr10	11.1425	12.5359	-1.393	0.01094
Marco	chr1	8.17201	9.56464	-1.393	0.00473
Rpl22	chr4	12.1786	13.5708	-1.392	0.001
Amdhd2	chr17	9.48148	10.8734	-1.392	0.00514
Rps6ka3	chrX	10.4735	11.864	-1.391	0.02433
Hnrnpa1	chr15	13.4699	14.8591	-1.389	0.00065
Hnrnpa2b1	chr6	14.2407	15.6292	-1.389	0.00515
Gatsl2	chr5	10.4523	11.8376	-1.385	0.00202
Pdcd10	chr3	8.90865	10.2939	-1.385	0.00145
Naglu	chr11	6.97265	8.35787	-1.385	0.01638
Ranbp17	chr11	10.4644	11.8495	-1.385	0.00262
Cdkn2d	chr9	8.26259	9.64705	-1.384	0.01268
Man2a2	chr7	7.09291	8.47718	-1.384	0.01245
Gclm	chr3	10.8523	12.2364	-1.384	9.4E-05
Eme1	chr11	8.64168	10.0248	-1.383	0.00403
Phpt1	chr2	9.65957	11.0412	-1.382	0.00533
Cdca7	chr2	8.9533	10.3339	-1.381	0.04947
Ttll1	chr15	9.91124	11.2913	-1.380	0.00584
Acvr2a	chr2	9.85746	11.2365	-1.379	0.00237
Rrp1	chr10	11.5468	12.925	-1.378	0.00226
Trip13	chr13	9.25687	10.6346	-1.378	0.01413
Cog6	chr3	11.2726	12.6485	-1.376	0.00338
Ercc6	chr14	11.9503	13.3246	-1.374	0.00011
Dctn4	chr18	11.1896	12.5632	-1.374	0.0008
Ocln	chr13	9.04184	10.4153	-1.373	0.00539
Rps15a	chr7	11.3358	12.7091	-1.373	0.0092
Pafah1b3	chr7	9.99103	11.3642	-1.373	0.00128
Rps7	chr12	13.1782	14.5506	-1.372	0.0034
Rpl24	chr16	10.4355	11.8069	-1.371	0.0008
Cmip	chr8	7.676	9.04693	-1.371	0.03228
Cdc16	chr8	9.92531	11.2959	-1.371	0.00057
Npm3	chr19	9.52234	10.8906	-1.368	8.6E-05
Fam160a2	chr7	10.2936	11.6615	-1.368	0.00132
Nif3l1	chr1	8.8446	10.2112	-1.367	0.03595
Dohh	chr10	9.16725	10.5338	-1.367	0.02241
Rplp2	chr7	11.2632	12.6293	-1.366	0.00781
Rpl30	chr15	11.4912	12.8571	-1.366	0.00536
Snx2	chr18	11.8779	13.2435	-1.366	0.00079
Fbxl18	chr5	9.48643	10.85	-1.364	0.00188
Mtmr6	chr14	10.293	11.6539	-1.361	4.6E-05
Armc5	chr7	8.13903	9.49974	-1.361	0.01216
Tro	chrX	9.33095	10.691	-1.360	0.00011
Pikfyve	chr1	12.0623	13.4219	-1.360	2.7E-05
Rbfa	chr18	8.75901	10.1182	-1.359	0.02464
M6pr	chr6	11.5654	12.9245	-1.359	0.00056
Nsun2	chr13	11.4193	12.7778	-1.358	0.00117
Zkscan1	chr5	11.594	12.9513	-1.357	0.00239
Minpp1	chr19	9.09148	10.4485	-1.357	0.00411
Ttc5	chr14	9.22614	10.5814	-1.355	0.03319
Itfg1	chr8	10.9843	12.3387	-1.354	1.7E-06
R3hdm2	chr10	13.8344	15.1846	-1.350	0.00229
Pgap2	chr7	7.29845	8.6482	-1.350	0.00725
Esr1	chr10	11.8165	13.1663	-1.350	0.00071
Sik1	chr17	10.2757	11.6246	-1.349	0.01191
Ndufc2	chr7	10.5245	11.8725	-1.348	0.01948
Trove2	chr1	8.34204	9.68841	-1.346	0.00779
Dom3z	chr17	8.72711	10.0725	-1.345	0.01077

Abhd10	chr16	8.54056	9.88518	-1.345	0.00787
Slc24a2	chr4	9.49677	10.841	-1.344	0.00137
Gm5643	chrX	7.80956	9.15293	-1.343	0.0313
Ndufs8	chr19	9.32679	10.67	-1.343	0.00081
Tpp2	chr1	11.1439	12.4867	-1.343	0.00129
Ubxn1	chr19	10.6505	11.9933	-1.343	0.0024
Ddx27	chr2	12.0976	13.44	-1.342	0.00074
4930547N16Rik	chr10	8.80889	10.1511	-1.342	0.00566
5730455P16Rik	chr11	8.36443	9.70573	-1.341	0.01159
Zbtb44	chr9	12.3246	13.6658	-1.341	0.00066
Ranbp10	chr8	10.0121	11.3529	-1.341	0.00351
Anks6	chr4	8.29216	9.6323	-1.340	0.03898
Atp9b	chr18	9.30033	10.6396	-1.339	0.00321
Nt5c3	chr6	8.0475	9.38656	-1.339	0.01505
Ddt	chr10	7.99538	9.33346	-1.338	0.04295
Pitrm1	chr13	11.7383	13.0756	-1.337	0.00663
Rps2	chr17	13.2253	14.5613	-1.336	0.00288
Fech	chr18	9.35748	10.6914	-1.334	0.01635
Rps14	chr18	11.844	13.1762	-1.332	0.02422
Ppp2r4	chr2	10.4238	11.7554	-1.332	0.00318
Atp6ap1	chrX	10.8584	12.189	-1.331	0.00213
Vps11	chr9	10.8956	12.2258	-1.330	0.01764
Ppp1ca	chr19	10.9373	12.2673	-1.330	0.01613
Sirt2	chr7	9.01868	10.3484	-1.330	0.0303
Zbtb1	chr12	9.37152	10.6998	-1.328	0.02634
Cul4a	chr8	11.2321	12.5588	-1.327	0.00203
Cadm1	chr9	11.7294	13.0556	-1.326	0.00011
Phrf1	chr7	10.4655	11.791	-1.325	4E-05
Pgam1	chr19	8.76506	10.0888	-1.324	0.00873
Snd1	chr6	12.7564	14.0789	-1.323	0.00225
Dgkh	chr14	11.3572	12.6794	-1.322	2.8E-05
Pigk	chr3	10.7695	12.0902	-1.321	0.00064
Arhgap32	chr9	9.74246	11.0625	-1.320	0.02477
Caprin1	chr2	13.0211	14.3405	-1.319	6.6E-05
Sec23a	chr12	12.5208	13.8397	-1.319	0.00015
Fam178b	chr1	7.32657	8.64514	-1.319	0.00086
Esd	chr14	8.33758	9.65553	-1.318	0.03283
Rasgef1b	chr5	8.70855	10.0263	-1.318	0.01758
Timm17b	chrX	9.34861	10.6659	-1.317	0.01864
Skiv2l2	chr13	12.9493	14.2645	-1.315	0.00197
Gak	chr5	10.6379	11.9529	-1.315	0.01305
Sgcb	chr5	7.45226	8.76665	-1.314	0.00839
Recql4	chr15	9.50322	10.8172	-1.314	0.00312
Rpn1	chr6	11.1655	12.4794	-1.314	0.00125
Mmgt1	chrX	10.8453	12.1586	-1.313	0.01214
Herc3	chr6	11.3142	12.6274	-1.313	0.00669
Smg7	chr1	12.3579	13.6704	-1.313	0.00045
Ogdh	chr11	12.6135	13.9259	-1.312	0.00038
Klhl22	chr16	9.42095	10.7333	-1.312	0.00633
Rhobtb3	chr13	7.37537	8.68768	-1.312	0.00952
Tmem127	chr2	9.60226	10.9144	-1.312	0.00122
Aifm1	chrX	11.3195	12.6316	-1.312	0.00013
Nkiras2	chr11	8.53912	9.8512	-1.312	0.01765
Ost4	chr5	9.02688	10.3389	-1.312	0.03524
Amz2	chr11	10.6788	11.9878	-1.309	0.00169
Tpr	chr1	13.8734	15.1813	-1.308	0.00025
Tspyl2	chrX	10.6463	11.9532	-1.307	0.00678

Eef1b2	chr1	12.1335	13.44	-1.307	0.00777
Eral1	chr11	8.22733	9.53371	-1.306	0.0424
Exosc5	chr7	8.26832	9.57375	-1.305	0.00084
Uap1	chr1	9.24484	10.5496	-1.305	0.00914
Rsu1	chr2	9.998	11.3013	-1.303	0.01012
Grwd1	chr7	8.17156	9.47481	-1.303	0.02444
Gm16517	chr10	8.74246	10.0452	-1.303	0.01333
Atpaf1	chr4	9.89789	11.2005	-1.303	0.00463
Kdm5c	chrX	12.2517	13.5534	-1.302	0.00302
Mtrr	chr13	8.70898	10.0104	-1.301	0.01113
Peli2	chr14	8.60603	9.90729	-1.301	0.03501
Dazap1	chr10	10.8632	12.1624	-1.299	0.00541
Ankhd1	chr18	11.9183	13.2174	-1.299	0.00012
Pcdh15	chr10	11.3691	12.6671	-1.298	0.00059
Uspl1	chr5	9.3336	10.6314	-1.298	0.00765
Magt1	chrX	9.07309	10.3706	-1.298	0.00053
Ahcyl1	chr3	11.4218	12.7186	-1.297	0.00402
Anapc5	chr5	12.6885	13.9848	-1.296	0.00765
Bag6	chr17	12.3786	13.6739	-1.295	0.00389
BC056474	chr8	9.29593	10.591	-1.295	0.02458
Xpo1	chr11	13.0645	14.3581	-1.294	0.0006
Sergef	chr7	8.33739	9.63069	-1.293	0.03261
Lmf1	chr17	8.47642	9.76925	-1.293	0.01384
Ep300	chr15	12.9014	14.1943	-1.293	0.00077
1500032L24Rik	chr15	10.7815	12.0737	-1.292	0.0268
Pick1	chr15	9.32597	10.6174	-1.291	0.0001
Ptgfrn	chr3	10.3963	11.6864	-1.290	0.00451
Gm608	chr16	11.2416	12.5292	-1.288	0.00111
2410127L17Rik	chr19	10.8872	12.1737	-1.286	0.00297
Ltn1	chr16	10.9642	12.2489	-1.285	0.00017
Rngtt	chr4	9.25783	10.5424	-1.285	0.00275
Nol6	chr4	10.5369	11.8208	-1.284	0.00085
1700011J10Rik	chr2	7.59263	8.87618	-1.284	0.0451
Atp5g2	chr15	12.4791	13.7614	-1.282	0.01338
Dync2li1	chr17	7.83414	9.1137	-1.280	0.04941
Map3k11	chr19	11.0624	12.3405	-1.278	0.00703
Ndufb7	chr8	8.48115	9.75865	-1.277	0.02648
Crcp	chr5	9.81571	11.0924	-1.277	0.00466
Pfn2	chr3	11.6003	12.8763	-1.276	0.00084
Gm340	chr19	10.6709	11.9468	-1.276	0.01289
Zfp385b	chr2	7.99958	9.27272	-1.273	0.01785
Metap1	chr3	9.79658	11.0694	-1.273	0.0192
Fis1	chr5	9.07426	10.347	-1.273	0.00488
P2rx4	chr5	7.81148	9.08374	-1.272	0.00125
Tufm	chr7	10.2037	11.4754	-1.272	0.00102
Sos2	chr12	9.53903	10.8103	-1.271	0.00071
Ap1s2	chrX	9.16039	10.4313	-1.271	0.02244
Ssr4	chrX	9.07334	10.3442	-1.271	0.00924
Ppp6r1	chr7	11.8703	13.1411	-1.271	9.4E-05
Senp7	chr16	8.05052	9.32126	-1.271	0.00326
Lin9	chr1	9.05691	10.3274	-1.270	0.04152
Psmc6	chr14	11.3752	12.6456	-1.270	0.0008
Cnpy3	chr17	8.83021	10.0991	-1.269	0.02239
Lypd6	chr2	9.14781	10.4155	-1.268	0.01017
Erbb2ip	chr13	10.6418	11.9092	-1.267	0.00815
Pdhb	chr14	11.584	12.8504	-1.266	0.00087
Cdc73	chr1	10.397	11.6629	-1.266	0.0006

Dis3l2	chr1	8.85423	10.1189	-1.265	0.04091
Tcn2	chr11	10.3325	11.597	-1.264	6.5E-05
Megf8	chr7	10.2944	11.558	-1.264	0.0035
Hdac2	chr10	12.7795	14.0423	-1.263	0.00041
Twf1	chr15	10.5597	11.822	-1.262	0.00128
Cdk19	chr10	8.48909	9.74777	-1.259	0.04877
Sptlc2	chr12	10.2626	11.5211	-1.259	0.00046
Rai1	chr11	9.86567	11.1222	-1.257	0.00219
Tpk1	chr6	8.10454	9.36061	-1.256	0.04794
Apool	chrX	8.53218	9.78793	-1.256	0.04099
Fam169a	chr13	10.1356	11.3907	-1.255	0.01444
Mapre3	chr5	8.16806	9.42256	-1.254	0.01654
Scyl1	chr19	10.6751	11.9287	-1.254	0.02505
Cadps2	chr6	9.7572	11.0087	-1.252	0.00608
Rps8	chr4	13.6336	14.885	-1.251	0.00879
Gnaz	chr10	9.45694	10.7057	-1.249	0.00165
Ppm1g	chr5	10.8699	12.1181	-1.248	0.0214
Poldip2	chr11	10.289	11.5371	-1.248	0.00366
Sdccag3	chr2	10.1903	11.4372	-1.247	0.00251
Mrpl30	chr1	9.22311	10.4688	-1.246	0.00702
Rsrc1	chr3	10.7909	12.0365	-1.246	0.00086
9130011J15Rik	chr8	11.1909	12.4352	-1.244	0.00024
Gbe1	chr16	11.0703	12.3143	-1.244	0.00065
Gclc	chr9	9.72082	10.9637	-1.243	0.00499
Dera	chr6	9.07516	10.3174	-1.242	0.01306
Tmem123	chr9	11.1401	12.3822	-1.242	0.0006
Bsg	chr10	11.617	12.8581	-1.241	0.00082
Irf3	chr7	8.97624	10.217	-1.241	0.01679
Smc5	chr19	10.9341	12.1748	-1.241	0.00081
Zfp639	chr3	8.49607	9.73649	-1.240	0.00259
Prpf4b	chr13	11.3426	12.5828	-1.240	0.00319
Acly	chr11	10.8005	12.0403	-1.240	0.00328
Uros	chr7	8.94742	10.1861	-1.239	0.04962
Golt1b	chr6	9.254	10.4923	-1.238	0.00932
Igf2r	chr17	13.2933	14.5291	-1.236	0.00012
Notch2	chr3	10.1264	11.3605	-1.234	0.00351
Tbce	chr13	8.83917	10.0715	-1.232	0.00117
Naa10	chrX	8.4456	9.67652	-1.231	0.01462
Lpin1	chr12	9.53119	10.7618	-1.231	0.00241
Grik5	chr7	7.38275	8.61302	-1.230	0.02115
Rexo2	chr9	10.754	11.9828	-1.229	0.00108
Cep110	chr2	10.735	11.9634	-1.228	0.00783
Lims1	chr10	10.4014	11.6287	-1.227	0.00063
Usp8	chr2	12.6101	13.8366	-1.227	0.00123
Ctr9	chr7	11.3525	12.5782	-1.226	0.00087
Rabggtb	chr3	10.2515	11.4764	-1.225	0.00022
Cuta	chr17	9.70496	10.9299	-1.225	0.02954
Kctd2	chr11	9.32755	10.5515	-1.224	0.00621
Hnrnph2	chrX	11.4209	12.6441	-1.223	0.0013
Senp5	chr16	11.3008	12.5225	-1.222	0.00203
Dapk1	chr13	8.21595	9.43737	-1.221	0.03107
Sar1a	chr10	10.1109	11.3316	-1.221	0.01028
Cacul1	chr19	10.4623	11.6827	-1.220	0.00187
Btbd9	chr17	11.0544	12.2748	-1.220	7.9E-05
Usp33	chr3	12.0698	13.2875	-1.218	0.00714
Txndc17	chr11	9.20133	10.4183	-1.217	0.01942
Rps11	chr7	11.8796	13.0959	-1.216	0.00111

Arhgef17	chr7	7.59096	8.80517	-1.214	0.04731
Letm1	chr5	11.5605	12.7745	-1.214	0.00062
4933434E20Rik	chr3	10.1218	11.3352	-1.213	0.01541
Rad50	chr11	14.0193	15.2302	-1.211	0.0051
2700099C18Rik	chr17	7.57815	8.78886	-1.211	0.03304
Zfp770	chr2	9.95851	11.1691	-1.211	0.00179
Cox5b	chr1	11.8545	13.0646	-1.210	0.00119
Cab39	chr1	12.0931	13.303	-1.210	0.00033
Ganab	chr19	12.4983	13.708	-1.210	1.2E-05
BC030336	chr7	8.98215	10.1917	-1.210	0.01351
Nek3	chr8	8.76671	9.97575	-1.209	0.01045
Nupl1	chr14	11.9428	13.1515	-1.209	1.2E-05
Strap	chr6	11.9807	13.1893	-1.209	0.00354
Uchl5	chr1	11.229	12.4367	-1.208	0.00195
Nsa2	chr13	10.7836	11.9912	-1.208	0.00457
Birc6	chr17	13.5188	14.7252	-1.206	0.00805
LOC100862375	chr14	8.03735	9.24193	-1.205	0.01854
Klc4	chr17	8.90556	10.1099	-1.204	0.01297
Dhx35	chr2	8.22662	9.42922	-1.203	0.02239
E130308A19Rik	chr4	9.2054	10.407	-1.202	0.00546
Gmppa	chr1	8.23164	9.43301	-1.201	0.01408
Mrps15	chr4	8.45871	9.65823	-1.200	0.02986
Leng8	chr7	11.517	12.7153	-1.198	0.0107
Bnip3l	chr14	13.6749	14.8713	-1.196	0.00101
Ccdc157	chr11	8.9139	10.1099	-1.196	0.00252
Zc3h11a	chr1	11.2433	12.4388	-1.195	0.00212
Snhg5	chr9	7.80288	8.99831	-1.195	0.02122
Zbtb34	chr2	10.6336	11.8283	-1.195	0.00497
Nos1	chr5	12.7175	13.9098	-1.192	0.00018
Fktn	chr4	7.63779	8.8282	-1.190	0.03462
G3bp1	chr11	10.9503	12.1407	-1.190	0.00378
Erc1	chr6	10.2229	11.4131	-1.190	0.00754
2610507B11Rik	chr11	12.083	13.2728	-1.190	0.001
Akt3	chr1	12.7689	13.9586	-1.190	0.0002
Ankib1	chr5	10.1626	11.3507	-1.188	0.02022
Ryr2	chr13	9.38407	10.5719	-1.188	0.00211
Tmem135	chr7	11.2053	12.3931	-1.188	0.00358
Eri3	chr4	8.81578	10.0036	-1.188	0.01859
Lypla1	chr1	10.4639	11.6498	-1.186	0.00136
Entpd4	chr14	8.06317	9.24834	-1.185	0.02219
Dennd4b	chr3	7.58816	8.77233	-1.184	0.0452
Ascc2	chr11	9.69156	10.8752	-1.184	0.01204
Akap8l	chr17	10.42	11.6018	-1.182	0.00421
Cep192	chr18	12.4885	13.6692	-1.181	0.00098
Rpl34	chr3	10.7763	11.9569	-1.181	0.00337
Rspry1	chr8	10.2758	11.456	-1.180	0.00207
Aptx	chr4	10.1721	11.3521	-1.180	0.00252
Ccdc112	chr18	9.3303	10.51	-1.180	0.00082
Tyk2	chr9	9.88065	11.0601	-1.179	0.00693
Ndufb2	chr6	9.83873	11.017	-1.178	0.04575
Stau2	chr1	12.2576	13.4353	-1.178	3.3E-05
Cdkn1b	chr6	10.9947	12.1719	-1.177	0.00486
Ankrd12	chr17	11.4802	12.6556	-1.175	0.0306
9930104L06Rik	chr4	8.20797	9.38287	-1.175	0.03441
Ahcyl2	chr6	11.3283	12.5032	-1.175	0.00062
Vbp1	chrX	12.1933	13.3647	-1.171	0.00581
Sp3	chr2	10.6354	11.8067	-1.171	0.00687

Ykt6	chr11	10.3446	11.5141	-1.169	0.00579
Tspan31	chr10	9.82955	10.9946	-1.165	0.0052
Sh3glb1	chr3	10.688	11.8508	-1.163	0.01542
Zswim5	chr4	11.0358	12.1977	-1.162	0.00023
Chmp5	chr4	11.7056	12.8668	-1.161	0.01522
Itch	chr2	10.5312	11.692	-1.161	0.00191
Kifc5b	chr17	9.11689	10.2772	-1.160	0.01439
Pank3	chr11	10.2598	11.4193	-1.160	0.01142
Brd3	chr2	10.865	12.0242	-1.159	0.00822
Taf5l	chr8	10.8052	11.9642	-1.159	0.00217
Pnpo	chr11	9.46396	10.6227	-1.159	0.04599
Cd3eap	chr7	9.41111	10.5694	-1.158	0.0312
Rtkn2	chr10	9.85823	11.0151	-1.157	0.00019
Atp5c1	chr2	12.0686	13.2252	-1.157	0.00311
Nek7	chr1	10.4827	11.6393	-1.157	0.00229
Cirbp	chr10	12.7113	13.8655	-1.154	0.00151
Adprhl2	chr4	7.47762	8.63043	-1.153	0.03822
Gns	chr10	10.4578	11.6102	-1.152	0.02048
2900092E17Rik	chr7	9.8018	10.9534	-1.152	0.01099
Atp6v0b	chr4	11.0631	12.2136	-1.151	0.0024
Mrpl13	chr15	7.69099	8.84132	-1.150	0.03628
Zbtb22	chr17	8.72451	9.87339	-1.149	0.03464
Clasp1	chr1	12.934	14.0822	-1.148	0.00117
Catsper2	chr2	8.55484	9.70116	-1.146	0.01694
Edf1	chr2	9.75076	10.8963	-1.146	0.01846
Rpl36	chr17	10.9155	12.0607	-1.145	0.00988
Poldip3	chr15	11.2154	12.3606	-1.145	0.00033
Akap13	chr7	13.328	14.4728	-1.145	0.00092
Gtf2e1	chr16	9.59135	10.736	-1.145	0.03607
Med24	chr11	10.9383	12.0825	-1.144	0.04144
Mnd1	chr3	10.1469	11.2904	-1.143	0.01725
St5	chr7	9.26146	10.4037	-1.142	0.04299
Nln	chr13	11.3039	12.4446	-1.141	0.00291
Rps17	chr7	11.9263	13.0666	-1.140	0.00697
Phf7	chr14	9.58151	10.7217	-1.140	0.00453
Lats1	chr10	10.0941	11.2336	-1.140	0.00152
Ankrd50	chr3	10.6777	11.8173	-1.140	0.00645
Chd1l	chr3	11.0616	12.2002	-1.139	0.0004
C1qbp	chr11	10.6301	11.7675	-1.137	0.00293
Ash2l	chr8	11.727	12.864	-1.137	0.0002
Trim23	chr13	9.90345	11.0396	-1.136	0.01859
Ears2	chr7	7.54839	8.68414	-1.136	0.04454
Ltbp3	chr19	8.53126	9.667	-1.136	0.00428
Eif4e2	chr1	11.6119	12.747	-1.135	0.00166
Dhx40	chr11	11.4581	12.5928	-1.135	2.5E-05
Rabl3	chr16	9.47435	10.6086	-1.134	0.02639
Lifr	chr15	8.86549	9.99824	-1.133	0.00216
Slc12a2	chr18	11.856	12.9887	-1.133	0.00029
Rnpc3	chr3	9.84889	10.9815	-1.133	0.00054
Snurf	chr7	9.08878	10.2199	-1.131	0.02002
Tmem43	chr6	8.33363	9.46429	-1.131	0.00198
Cldn12	chr5	8.68891	9.81888	-1.130	0.04326
Arl2bp	chr8	8.99214	10.1196	-1.127	0.01972
Sarnp	chr10	10.4687	11.5958	-1.127	0.00585
Slc25a28	chr19	8.08507	9.21108	-1.126	0.03516
Reps2	chrX	8.89094	10.0154	-1.124	0.00172
Eea1	chr10	13.4295	14.5538	-1.124	0.00212

Tspan3	chr9	10.3848	11.5087	-1.124	0.02464
Prkaca	chr8	10.2072	11.331	-1.124	0.00141
Rps4x	chrX	13.7458	14.8695	-1.124	0.00145
Ehd1	chr19	10.4573	11.5809	-1.124	0.00957
Zfp775	chr6	9.16048	10.2831	-1.123	0.01399
Col4a1	chr8	9.62204	10.744	-1.122	0.00022
Wwox	chr8	9.37966	10.5008	-1.121	0.03117
Mtch1	chr17	10.3675	11.4883	-1.121	0.01011
Asna1	chr8	11.4853	12.6058	-1.120	0.01996
Usmg5	chr19	10.1097	11.2299	-1.120	0.03518
Ptpn9	chr9	8.73762	9.85604	-1.118	0.04196
Atad2	chr15	12.0941	13.2107	-1.117	0.00135
Rps6kb2	chr19	8.41942	9.53516	-1.116	0.00072
Ifnar1	chr16	10.9413	12.057	-1.116	0.00619
Rpl32	chr6	12.3746	13.4896	-1.115	0.01121
Ccdc127	chr13	9.54623	10.6608	-1.115	0.02914
Mgat5	chr1	10.0247	11.1391	-1.114	0.00145
Bclaf1	chr10	12.4733	13.5876	-1.114	0.00031
Cir1	chr2	11.7088	12.8228	-1.114	0.00225
Mllt4	chr17	14.0397	15.1529	-1.113	0.00026
Frmpd1	chr4	9.24924	10.3622	-1.113	0.01103
Myh9	chr15	14.1604	15.2728	-1.112	0.00023
Enah	chr1	12.2319	13.3435	-1.112	0.00161
Rpl27	chr11	9.53872	10.6497	-1.111	0.00076
Ero1lb	chr13	13.0294	14.1394	-1.110	0.0003
Ylpm1	chr12	12.122	13.231	-1.109	0.00071
Plekha5	chr6	11.5203	12.6288	-1.108	0.0031
1700029J07Rik	chr8	7.83354	8.93966	-1.106	0.01874
Zfp597	chr16	10.4538	11.559	-1.105	0.00021
Eif3g	chr9	12.9911	14.0955	-1.104	0.00482
Shkbp1	chr7	8.52618	9.6304	-1.104	0.03099
Csrp1	chr1	11.118	12.2218	-1.104	0.00777
Agpat3	chr10	10.5297	11.6329	-1.103	0.01684
Tmem194	chr10	10.0967	11.1997	-1.103	0.01537
Ergic3	chr2	11.7505	12.8534	-1.103	0.00027
Zmynd11	chr13	11.1449	12.2477	-1.103	0.00165
Rnf44	chr13	10.6659	11.7668	-1.101	0.00011
Rrp7a	chr15	10.6154	11.7137	-1.098	0.00822
Mical3	chr6	11.5857	12.6831	-1.097	0.02646
Ptprs	chr17	11.1223	12.2196	-1.097	0.00608
Dido1	chr2	11.8307	12.928	-1.097	0.00026
Erf	chr7	7.79305	8.88994	-1.097	0.04145
Aprt	chr8	8.05126	9.14676	-1.095	0.03853
Uba6	chr5	11.9294	13.0234	-1.094	4.3E-05
Slk	chr19	12.32	13.4137	-1.094	0.00427
Tcf3	chr10	10.9724	12.0653	-1.093	0.0008
Ppfibp1	chr6	11.717	12.8091	-1.092	0.00199
Eif2c2	chr15	10.4156	11.5073	-1.092	0.03491
Atf6b	chr17	10.7672	11.8585	-1.091	0.00345
Cpeb3	chr19	12.5337	13.6243	-1.091	0.0046
Ecsit	chr9	9.67737	10.7679	-1.091	0.02127
Ap4e1	chr2	9.83648	10.9261	-1.090	0.0027
Adipor2	chr6	11.1568	12.2463	-1.090	0.00595
Hdhd2	chr18	8.49818	9.58732	-1.089	0.03821
Zswim7	chr11	8.99178	10.0807	-1.089	0.02544
Tab3	chrX	12.1354	13.2242	-1.089	0.01195
2310061I04Rik	chr17	11.6386	12.7272	-1.089	0.01366

Ndufs2	chr1	12.9489	14.0365	-1.088	0.00352
Tm9sf3	chr19	12.6292	13.7161	-1.087	0.00079
Acadm	chr3	8.992	10.0786	-1.087	0.01656
Pacs1	chr19	11.7614	12.8469	-1.086	0.00055
Metap1d	chr2	9.02511	10.1106	-1.086	0.02082
Mlst8	chr17	7.93396	9.01929	-1.085	0.03944
Cbx5	chr15	12.1788	13.2637	-1.085	0.00761
Ndufa6	chr15	9.71971	10.8036	-1.084	0.00375
Svil	chr18	9.65377	10.7376	-1.084	0.01103
Ptprj	chr2	13.5472	14.631	-1.084	2.8E-05
Spata5	chr3	12.5391	13.6222	-1.083	0.00011
Man2a1	chr17	10.3312	11.4135	-1.082	0.00595
Ptges2	chr2	8.96211	10.0429	-1.081	0.0204
Dynlt3	chrX	10.707	11.7853	-1.078	0.00136
Eif4ebp2	chr10	7.58365	8.66145	-1.078	0.00565
Pdpr	chr8	10.9967	12.0745	-1.078	0.01781
Sel1l	chr12	12.4635	13.5406	-1.077	0.00063
Gtf3c3	chr1	10.3454	11.4224	-1.077	0.00366
Mdm1	chr10	8.96171	10.0383	-1.077	0.04899
Vps25	chr11	11.0129	12.0888	-1.076	0.00058
Rplp1	chr9	11.876	12.9518	-1.076	0.02069
Usp14	chr18	11.6245	12.6997	-1.075	0.00016
Klhl24	chr16	10.7151	11.789	-1.074	0.0013
Dusp23	chr1	7.69805	8.77002	-1.072	0.02565
3110043O21Rik	chr4	11.7627	12.8339	-1.071	0.00176
2310035C23Rik	chr1	11.2155	12.2867	-1.071	0.00695
Rpl21	chr5	13.2329	14.3027	-1.070	0.00813
Gdpd1	chr11	13.495	14.5637	-1.069	0.00042
Tead2	chr7	7.70533	8.77382	-1.068	0.02591
Tsta3	chr15	8.35181	9.41906	-1.067	0.03075
Hmgb1	chr5	12.7285	13.795	-1.067	0.00179
Pkig	chr2	10.6301	11.6966	-1.067	0.03233
Mcrs1	chr15	11.1995	12.2659	-1.066	0.00101
Tesk1	chr4	10.0242	11.0904	-1.066	0.02249
Acer3	chr7	8.26955	9.33496	-1.065	0.00328
Fancl	chr11	9.05366	10.1185	-1.065	0.00333
Rimklb	chr6	12.7186	13.783	-1.064	0.01325
Dcaf17	chr2	10.137	11.2014	-1.064	0.001
Rad51ap1	chr6	8.11166	9.17519	-1.064	0.04034
Dhx36	chr3	11.7844	12.8461	-1.062	0.00259
Gps2	chr11	10.6082	11.6693	-1.061	0.00352
Ube2j1	chr4	10.2199	11.281	-1.061	0.01333
Pcmtd1	chr1	10.0815	11.1423	-1.061	0.01588
Dnajc10	chr2	12.006	13.0665	-1.061	0.00085
Ttc28	chr5	10.3417	11.4016	-1.060	0.04782
Hnrnpr	chr4	11.6611	12.7201	-1.059	0.01315
Zdhhc6	chr19	9.87993	10.9387	-1.059	0.0037
Cul2	chr18	10.5987	11.6574	-1.059	0.00338
Hectd1	chr12	14.1743	15.2312	-1.057	0.00064
Fau	chr19	11.2604	12.3171	-1.057	0.01762
Pbx3	chr2	7.12897	8.18466	-1.056	0.01962
Smchd1	chr17	13.3289	14.3843	-1.055	0.0019
Ppid	chr3	11.0102	12.0647	-1.054	0.00346
Tbl1xr1	chr3	13.05	14.1042	-1.054	0.00108
Marf1	chr16	14.2229	15.2766	-1.054	0.0006
Zc3h14	chr12	13.4728	14.5265	-1.054	9.6E-05
Mrpl33	chr5	8.48698	9.54018	-1.053	0.03271

Spata13	chr14	9.38071	10.4332	-1.053	0.00861
Pnldc1	chr17	9.0353	10.0869	-1.052	0.00799
Lars	chr18	10.8707	11.9215	-1.051	0.02001
Dnmbp	chr19	8.49243	9.54253	-1.050	0.01546
Mrpl41	chr2	8.12917	9.17895	-1.050	0.02439
Ube2d1	chr10	8.02367	9.07331	-1.050	0.04698
Tbc1d23	chr16	10.6349	11.6845	-1.050	0.02694
Erc2	chr14	10.7017	11.7511	-1.049	0.00598
Ulk2	chr11	9.692	10.7411	-1.049	0.00894
Prmt1	chr7	10.8615	11.9095	-1.048	0.00169
Trmt6	chr2	9.94971	10.9973	-1.048	0.00528
Ap2m1	chr16	12.4372	13.4833	-1.046	0.0003
Dis3	chr14	9.85713	10.9015	-1.044	0.01693
Mtx2	chr2	11.1012	12.1433	-1.042	0.00687
Taf5	chr19	12.3339	13.376	-1.042	0.00501
Minos1	chr4	10.9268	11.9682	-1.041	0.03521
Pgp	chr17	8.40952	9.44841	-1.039	0.01031
Acat1	chr9	12.1541	13.1917	-1.038	0.00705
Mtpn	chr6	12.9332	13.9705	-1.037	0.00256
Dmxl2	chr9	13.288	14.3249	-1.037	0.00104
Dcun1d4	chr5	10.7999	11.8355	-1.036	0.00402
1700001L05Rik	chr15	12.0718	13.1068	-1.035	0.00661
Arhgap42	chr9	11.9978	13.0323	-1.035	0.00059
Atp5o	chr16	11.2081	12.2412	-1.033	0.02011
Vps16	chr2	10.5966	11.6282	-1.032	0.00028
Map2k7	chr8	11.0256	12.0565	-1.031	0.03704
Iqgap3	chr3	12.6456	13.6751	-1.029	0.00104
Gm5595	chr7	7.83241	8.86042	-1.028	0.01534
Utp14a	chrX	10.774	11.8017	-1.028	0.01219
Fam82b	chr4	7.88719	8.91321	-1.026	0.03636
Fbxl3	chr14	10.2869	11.3121	-1.025	0.03152
Rasa1	chr13	12.2313	13.2558	-1.025	0.00016
Sdhc	chr1	10.8388	11.8634	-1.025	0.00506
Ccnd3	chr17	9.61765	10.6415	-1.024	0.00707
Mrpl50	chr4	8.4223	9.44559	-1.023	0.01241
Man1a2	chr3	10.4339	11.4568	-1.023	0.00582
Csnk1g1	chr9	10.5423	11.565	-1.023	0.01561
Tmem126b	chr7	7.5431	8.56538	-1.022	0.02004
Zfp940	chr7	9.16603	10.1869	-1.021	0.02384
Rps6ka1	chr4	9.59672	10.617	-1.020	0.02466
Taldo1	chr7	11.5387	12.559	-1.020	0.01091
Cdk13	chr13	11.658	12.6778	-1.020	0.00043
Fam13b	chr18	10.4941	11.5136	-1.019	0.01753
Eif4a2	chr16	13.2943	14.3121	-1.018	0.00128
R3hdm1	chr1	12.4944	13.5119	-1.017	0.0024
Kdm2a	chr19	11.7596	12.7767	-1.017	0.03669
Ccdc15	chr9	10.4694	11.4864	-1.017	0.00148
Vps4b	chr1	10.7953	11.812	-1.017	9.6E-05
Gbas	chr5	11.8249	12.841	-1.016	0.00255
Wdr46	chr17	10.1629	11.1783	-1.015	0.00665
Sipa1l3	chr7	12.4782	13.4934	-1.015	0.00228
Luc7l	chr17	10.6013	11.6163	-1.015	0.00432
Mib2	chr4	10.4331	11.4465	-1.013	0.02435
Lamc1	chr1	12.2502	13.2625	-1.012	0.00828
Mdh2	chr5	10.8963	11.907	-1.011	0.00627
Reep3	chr10	8.28782	9.29852	-1.011	0.01903
Fkbp1a	chr2	11.7847	12.7942	-1.009	0.00413

Grhpr	chr4	9.64181	10.6503	-1.008	0.0128
Map1lc3b	chr8	10.9195	11.9272	-1.008	0.0041
Kdsr	chr1	10.1392	11.1458	-1.007	0.01003
Srsf1	chr11	13.0421	14.0481	-1.006	0.00078
Gca	chr2	7.3428	8.34547	-1.003	0.01678
2700060E02Rik	chr14	11.1675	12.1694	-1.002	0.01747
Pigo	chr4	9.45086	10.4513	-1.000	0.02814

Up in FGO

Gene Symbol	Chr	GV ave	ng ave	fold changes	t-test
E330011O21Rik	chr16	13.4324	0.67465	12.758	0.00012
Zfp735	chr11	12.6211	0.67465	11.946	0.00032
Gm5065	chr7	11.5419	0	11.542	0.00023
Upp1	chr11	12.8569	1.54046	11.316	0.00476
Epsti1	chr14	12.2291	1.00798	11.221	0.00255
Zar1l	chr5	13.306	2.09513	11.211	0.00967
Rgs17	chr10	12.7261	1.63731	11.089	0.00606
Pak7	chr2	12.396	1.33694	11.059	0.00109
Ceacam20	chr7	11.8481	0.89461	10.953	0.00648
Gm17751	chr1	12.869	1.93939	10.930	0.00717
Pde6b	chr5	10.9259	0	10.926	2.4E-05
Tubb3	chr8	13.5612	2.67027	10.891	0.00671
Pdlim2	chr14	12.6875	1.86526	10.822	0.00185
Gm13089	chr4	12.0819	1.32549	10.756	0.00064
Dnmt3l	chr10	12.6142	1.86526	10.749	0.002
7420461P10Rik	chr1	12.5944	1.84839	10.746	0.00791
Cndp1	chr18	10.7184	0	10.718	5.1E-05
Slc35e4	chr11	10.6772	0	10.677	0.00078
Gm16551	chr9	12.1805	1.53192	10.649	0.00084
Mettl7a3	chr15	11.572	0.96436	10.608	0.00712
Gm1965	chr6	13.4068	2.83422	10.573	0.00031
Crabp2	chr3	12.3104	1.74462	10.566	0.00805
Fam19a2	chr10	10.861	0.30836	10.553	0.00025
Slc6a15	chr10	12.5506	2.0036	10.547	0.00345
Dclk3	chr9	10.4896	0	10.490	0.0001
Gm15941	chr15	11.783	1.30207	10.481	0.01502
Mir1941	chr15	10.45	0	10.450	4.5E-05
Rhoh	chr5	11.4028	0.97065	10.432	0.00257
Gm6600	chr6	11.0966	0.67465	10.422	0.00013
Eps8l3	chr3	10.4175	0	10.418	5.2E-06
Icosl	chr10	12.3388	1.9219	10.417	0.00358
Ccdc3	chr2	10.7437	0.32896	10.415	0.00012
Ildr1	chr16	10.4011	0	10.401	0.00013
Aqp9	chr9	10.3755	0	10.376	3.9E-05
Gpr35	chr1	11.6753	1.30398	10.371	0.0024
Trp73	chr4	10.3673	0	10.367	1.5E-05
Tmprss11a	chr5	11.6569	1.33694	10.320	0.00083
4930588N13Rik	chr5	10.6097	0.32896	10.281	7.5E-05
Gjb1	chrX	10.2704	0	10.270	7.9E-05
Sebox	chr11	10.599	0.32896	10.270	0.00093
Gm17830	chr17	10.5857	0.32896	10.257	0.00049
Obox5	chr7	14.249	3.99603	10.253	6.2E-05
Nckap1l	chr15	10.5925	0.36629	10.226	0.00056
LOC100416360	chr17	11.157	0.93961	10.217	0.00139
Oosp1	chr19	15.5392	5.32936	10.210	0.00309
Nek10	chr14	10.8575	0.65125	10.206	2.3E-05
Tubal3	chr13	11.5714	1.40263	10.169	0.0008
Zfp92	chrX	11.1745	1.01388	10.161	0.00352
Gm813	chr16	13.7776	3.61772	10.160	0.0106
Dpp10	chr1	11.2379	1.08233	10.156	0.01067
Pax2	chr19	10.49	0.36629	10.124	0.00031
Gm4745	chr7	11.602	1.49897	10.103	0.00541
Gm16497	chr12	10.8012	0.69962	10.102	0.00378

Gm10466	chr11	10.1014	0	10.101	2.3E-07
Aadac	chr3	11.1077	1.00798	10.100	0.00216
Pid1	chr1	11.0684	0.97502	10.093	0.00884
Gna14	chr19	11.2152	1.12817	10.087	0.01168
Fbxw20	chr9	14.0151	3.93181	10.083	0.0003
Scin	chr12	11.0838	1.0036	10.080	2.3E-08
Slc26a9	chr1	10.38	0.30836	10.072	2.1E-05
Kcnk13	chr12	11.2894	1.22357	10.066	0.00468
Nlrp2	chr7	14.6669	4.61654	10.050	0.00039
Oog3	chr4	14.8837	4.83755	10.046	0.00296
Grm2	chr9	10.678	0.66229	10.016	0.00301
Oog2	chr4	12.9036	2.89086	10.013	0.00088
Spry4	chr18	15.7031	5.72547	9.978	0.00174
Scel	chr14	10.2724	0.30836	9.964	0.0005
Myo16	chr8	11.5568	1.6002	9.957	0.02486
Gm13242	chr4	12.4933	2.54143	9.952	0.01667
Gsdmc2	chr15	10.6505	0.71488	9.936	0.00099
Vmn1r139	chr7	10.0273	0.11223	9.915	0.00013
Cpa1	chr6	13.119	3.21339	9.906	0.00388
Wfdc3	chr2	9.89375	0	9.894	7.8E-05
S100a14	chr3	10.1915	0.30836	9.883	0.00091
Slc5a12	chr2	10.1882	0.30836	9.880	6.2E-05
Mettl7a2	chr15	12.2687	2.38952	9.879	0.00558
Gm4975	chr8	10.2431	0.36629	9.877	0.00067
C87977	chr4	14.6534	4.77815	9.875	0.00082
9130409I23Rik	chr1	11.5493	1.67729	9.872	0.00127
Vmn1r132	chr7	9.89922	0.03296	9.866	0.00063
Tll1	chr8	11.636	1.77758	9.858	0.00667
Olfr288	chr15	11.3036	1.47431	9.829	0.00766
Hoxa7	chr6	9.82427	0	9.824	0.00021
Mrc1	chr2	11.0489	1.22794	9.821	0.01495
Mfsd2a	chr4	13.7071	3.90835	9.799	0.00654
Obox2	chr7	14.8751	5.08154	9.794	0.00189
Rora	chr9	9.7806	0	9.781	1.3E-05
Nlrp9c	chr7	14.5306	4.75474	9.776	0.0003
Pabpc1l	chr2	14.363	4.6082	9.755	0.00088
AB099516	chr15	12.1033	2.35612	9.747	0.00558
Fbxw19	chr9	15.4429	5.70927	9.734	0.00147
Umodl1	chr17	14.1378	4.41918	9.719	0.00289
C87499	chr4	14.326	4.61351	9.712	0.00026
Tcl1b4	chr12	9.70523	0	9.705	0.00025
Tcstv1	chr13	9.66285	0	9.663	0.00024
Pdx1	chr5	9.65523	0	9.655	0.00026
Tfap2e	chr4	12.9436	3.2896	9.654	0.01849
Zfp616	chr11	13.2504	3.60439	9.646	0.00288
Csf1	chr3	11.6038	1.96436	9.639	0.01298
Pacsin1	chr17	12.1676	2.53192	9.636	0.0011
Gm11545	chr11	14.3916	4.75783	9.634	0.00083
D16Ertd519e	chr16	11.1583	1.53192	9.626	9.9E-06
Fbxw14	chr9	15.4149	5.79268	9.622	0.00042
Gm12130	chr11	11.1379	1.51943	9.618	0.02381
Kcnmb2	chr3	9.60562	0	9.606	0.00052
Hspa1b	chr17	9.97149	0.36629	9.605	0.00108
7420426K07Rik	chr9	10.9374	1.34131	9.596	0.00699
Gm14635	chrX	9.57354	0	9.574	0.00034
Def6	chr17	10.4613	0.89461	9.567	0.00723
Nlrp9b	chr7	15.1487	5.58878	9.560	0.0058

Clec12a	chr6	11.468	1.93961	9.528	0.02233
Sec14l3	chr11	9.88888	0.36629	9.523	0.0014
Col4a3	chr1	9.51333	0	9.513	0.00025
Evx1	chr6	10.2	0.69525	9.505	0.00083
Cmtm8	chr9	9.53651	0.03296	9.504	5.3E-05
Bmp5	chr9	12.074	2.58856	9.485	1.5E-06
Igsf23	chr7	10.7824	1.2977	9.485	0.0079
Gm7056	chr5	13.2732	3.79347	9.480	0.00011
Fgf1	chr18	9.47729	0	9.477	0.00053
Ankrd33b	chr15	10.8185	1.34131	9.477	0.0059
Cxcr3	chrX	9.47551	0	9.476	0.00012
Nanos2	chr7	11.2206	1.75189	9.469	0.01793
Tmem74	chr15	9.46799	0	9.468	8.9E-05
Gm2042	chr12	10.1852	0.71788	9.467	0.00471
Fbxw28	chr9	15.5386	6.07232	9.466	0.00311
Selp	chr1	9.45958	0	9.460	0.00012
LOC100503496	chr11	9.45573	0	9.456	0.00018
Spam1	chr6	9.80809	0.36629	9.442	0.001
Gm10436	chr12	14.3242	4.8969	9.427	2.6E-07
Gm839	chr6	12.0053	2.58387	9.421	0.01138
Ctsh	chr9	13.0941	3.7125	9.382	0.02025
Il17f	chr1	9.70742	0.32896	9.378	3.6E-05
Adm2	chr15	9.37226	0	9.372	6.9E-05
B3gat2	chr1	9.36894	0	9.369	5.9E-06
AI848285	chr15	10.3648	1.0036	9.361	3.1E-05
1810041L15Rik	chr15	10.0013	0.64169	9.360	0.00201
Oas1f	chr5	10.736	1.3768	9.359	0.00449
Tlr9	chr9	9.33317	0	9.333	8.5E-05
Obox1	chr7	13.9107	4.58631	9.324	0.00577
Cntnap1	chr11	12.8822	3.56108	9.321	0.00661
Gpr1	chr1	12.6499	3.32936	9.321	0.0141
2610034M16Rik	chr17	9.3114	0	9.311	0.00013
Trim15	chr17	9.30387	0	9.304	7.9E-05
Gm10668	chr7	9.30204	0	9.302	0.00019
Gm3143	chr3	9.65406	0.36629	9.288	0.00013
Dlgap2	chr8	9.27574	0	9.276	0.00011
A330093E20Rik	chr18	11.1205	1.85166	9.269	0.0215
Rgn	chrX	11.5969	2.33313	9.264	0.03915
Pou2f2	chr7	10.7951	1.54037	9.255	0.00724
Dsg1a	chr18	9.24293	0	9.243	7.4E-05
Gabrq	chrX	9.23443	0	9.234	6.9E-05
Gm4567	chr7	9.22741	0	9.227	0.00028
Oog4	chr4	14.8039	5.58071	9.223	0.01104
Gm5	chr5	9.54513	0.32896	9.216	3.4E-05
Gm13088	chr4	9.20972	0	9.210	0.0002
Zfp423	chr8	10.1023	0.89461	9.208	0.00924
Gm10825	chr10	9.91412	0.74108	9.173	0.00412
Mira	chr6	9.80793	0.63731	9.171	9.8E-05
Prrg1	chrX	11.5142	2.36062	9.154	0.02533
Cyp2c53-ps	chr19	9.47225	0.32896	9.143	0.00089
Accsl	chr2	15.232	6.08918	9.143	0.00463
Rcsd1	chr1	9.13305	0	9.133	0.00052
Paqr6	chr3	10.8317	1.70041	9.131	0.00364
Mllt11	chr3	11.0156	1.88894	9.127	0.01062
41896	chr5	10.3253	1.20297	9.122	0.00559
Zfp750	chr11	13.2927	4.17088	9.122	0.00931
Muc19	chr15	9.42624	0.30836	9.118	2.8E-05

Cd7	chr11	11.4927	2.38662	9.106	0.02595
Dusp1	chr17	11.3539	2.25664	9.097	0.03638
Sost	chr11	9.40259	0.32896	9.074	0.00042
Nacad	chr11	9.92478	0.85728	9.068	0.00747
Pga5	chr19	10.2222	1.16563	9.057	0.00625
Gm14164	chr2	9.05593	0	9.056	5.9E-05
Vwa3a	chr7	10.6427	1.59807	9.045	0.02886
Kcng4	chr8	10.2091	1.17001	9.039	0.01583
Vwa2	chr19	11.2001	2.16563	9.034	0.02003
Cyp19a1	chr9	10.3264	1.30207	9.024	0.01961
Gm13103	chr4	13.3996	4.37776	9.022	0.00809
Sec16b	chr1	9.98961	0.97065	9.019	0.00308
Grem1	chr2	9.01318	0	9.013	0.00091
Nlrp9a	chr7	14.4305	5.41829	9.012	0.00878
E230008N13Rik	chr4	9.31385	0.30836	9.005	8.5E-05
Fgf8	chr19	10.9359	1.94376	8.992	0.0148
Pabpn1l	chr8	12.9696	3.99888	8.971	0.01244
Ank3	chr10	11.2142	2.2459	8.968	0.01887
BC051537	chr17	9.66358	0.69962	8.964	0.00602
Ccdc114	chr7	9.70924	0.749	8.960	0.00643
Olfr1300-ps1	chr2	10.1269	1.19061	8.936	0.01705
Gm13023	chr4	14.6307	5.70572	8.925	0.0029
Chrnd	chr1	10.9557	2.07796	8.878	0.01881
Hsd17b13	chr5	8.86951	0	8.870	6.3E-06
AU021063	chr15	8.86502	0	8.865	2.3E-05
Sptssb	chr3	9.19254	0.32896	8.864	4.1E-05
Gm10857	chr2	12.8605	4.00156	8.859	0.01399
Asb15	chr6	8.84139	0	8.841	0.00022
Stk32c	chr7	11.7668	2.93939	8.827	0.01065
Lhb	chr7	8.82446	0	8.824	1.5E-05
Gnat2	chr3	8.81246	0	8.812	0.00067
Krt84	chr15	11.6771	2.86963	8.807	0.02785
Clvs2	chr10	12.4208	3.61457	8.806	0.01191
Rnase6	chr14	8.78155	0	8.782	9.5E-05
Ikzf4	chr10	11.017	2.24146	8.776	0.02233
Plac8	chr5	12.8711	4.09661	8.774	0.02884
Pla2g4e	chr2	8.77426	0	8.774	9E-05
Edn1	chr13	8.77066	0	8.771	0.00114
Gpx6	chr13	9.3922	0.63731	8.755	0.00011
Tlr3	chr8	8.7485	0	8.749	0.00137
Chn2	chr6	10.5368	1.79046	8.746	0.02306
Proc	chr18	10.2637	1.51943	8.744	0.02882
Hc	chr2	8.73414	0	8.734	0.00067
Chrng	chr1	9.0223	0.32896	8.693	4.8E-05
Slc2a13	chr15	8.69184	0	8.692	0.00038
Gm5820	chr18	9.35267	0.66826	8.684	0.00412
Slc2a12	chr10	10.209	1.53192	8.677	0.00677
Gm5946	chrX	11.6692	2.99657	8.673	0.03292
Htra4	chr8	10.3659	1.69525	8.671	0.01869
Iqck	chr7	8.66474	0	8.665	0.00017
Themis3	chr17	9.434	0.7696	8.664	0.00737
Fbxw25	chr9	10.7485	2.08911	8.659	0.02027
C87414	chr5	15.645	6.98558	8.659	0.00195
Myom2	chr8	13.3639	4.70644	8.657	0.01825
Clec2f-ps	chr6	8.65482	0	8.655	0.00072
BC068157	chr8	9.50961	0.85728	8.652	0.00739
Klf17	chr4	15.0419	6.42085	8.621	0.00055

Gm6367	chr5	14.6164	5.99699	8.619	0.00523
Gpr149	chr3	11.0068	2.39358	8.613	0.0081
Acpp	chr9	10.9407	2.33313	8.608	0.04359
Best2	chr8	8.91427	0.30836	8.606	0.0004
Ddit3	chr10	10.0399	1.43627	8.604	0.02497
Adcy8	chr15	8.60321	0	8.603	0.00035
Gpr158	chr2	10.0122	1.41129	8.601	0.01274
Slc10a6	chr5	11.0441	2.45712	8.587	0.04914
E330021D16Rik	chr6	16.4362	7.85229	8.584	0.00047
Pramef17	chr4	8.58035	0	8.580	0.00011
Gm13084	chr4	15.0699	6.48956	8.580	0.00342
Thsd7b	chr1	10.8173	2.23708	8.580	0.01878
Slc15a5	chr6	10.2486	1.67465	8.574	0.03394
Mill1	chr7	8.6017	0.03296	8.569	3.9E-05
Rnf207	chr4	8.5566	0	8.557	0.0012
Psrc1	chr3	12.4081	3.86022	8.548	0.01193
Igl	chr16	8.54449	0	8.544	0.00038
Rtn4r	chr16	8.87171	0.32896	8.543	0.00011
Trpc5	chrX	8.54158	0	8.542	0.00101
Olfr287	chr15	11.3771	2.83654	8.541	0.02677
Cyp2j12	chr4	8.53582	0	8.536	0.00035
Gsdmc3	chr15	10.224	1.69428	8.530	0.03538
Gm12633	chr4	9.71904	1.19061	8.528	0.01844
Mmp24	chr2	9.52555	1.0036	8.522	0.00012
Tnni3	chr7	9.44546	0.93141	8.514	0.00743
Gm10280	chr8	8.51344	0	8.513	0.00033
Cpne9	chr6	12.1242	3.61457	8.510	0.02238
Prss12	chr3	9.54129	1.03296	8.508	0.00946
Pde1b	chr15	11.6104	3.10334	8.507	0.00128
Adcy5	chr16	12.002	3.50089	8.501	0.02178
Tmco4	chr4	10.3152	1.81621	8.499	0.04135
Pcbd1	chr10	10.1193	1.64169	8.478	0.03216
Slamf1	chr1	8.84351	0.36629	8.477	0.0007
A1bg	chr15	9.47112	0.99562	8.475	0.01332
Trpc4	chr3	9.47237	1.0036	8.469	0.00011
Slc2a9	chr5	8.4544	0	8.454	0.0005
Bpifc	chr10	10.1216	1.66859	8.453	0.03233
9430031J16Rik	chr1	8.45025	0	8.450	4.4E-05
Npffr2	chr5	9.07314	0.63731	8.436	0.00074
Cyp4v3	chr8	8.43434	0	8.434	0.00047
Tcl1b5	chr12	8.43233	0	8.432	0.00067
Gipc2	chr3	10.3987	1.97065	8.428	0.00957
Oog1	chr12	14.7025	6.27982	8.423	0.00038
Gm97	chr19	14.2934	5.88863	8.405	0.00033
C130060K24Rik	chr6	11.4207	3.0184	8.402	0.00763
Snph	chr2	11.1792	2.77758	8.402	0.02264
7420700N18Rik	chr8	8.39081	0	8.391	0.00024
2300002M23Rik	chr17	9.41859	1.03296	8.386	0.01253
Hspa1a	chr17	8.3759	0	8.376	0.00024
Nlrp4a	chr7	15.1755	6.8051	8.370	0.00718
Fat3	chr9	10.7231	2.36062	8.362	0.02044
Mab21l1	chr3	8.36194	0	8.362	0.00015
Gm6205	chr5	14.3514	6.01029	8.341	0.00557
LOC101055820	chr8	8.65889	0.32896	8.330	6.7E-05
Hs3st5	chr10	9.83323	1.50334	8.330	0.03077
Dsc2	chr18	9.91609	1.59539	8.321	0.01673
Gm20362	chr6	10.3957	2.08522	8.310	0.01665

LOC433944	chr5	9.79073	1.4811	8.310	0.01364
Sptlc3	chr2	8.9451	0.64169	8.303	0.0035
Esr2	chr12	12.3317	4.02832	8.303	0.00548
LOC100502896	chr4	8.29803	0	8.298	0.00046
Nlrc4	chr17	10.5729	2.27569	8.297	0.0273
Lrrc4	chr6	10.9474	2.65934	8.288	0.04234
Gm18683	chr14	8.28364	0	8.284	0.00013
Susd3	chr13	11.2618	2.98544	8.276	0.03458
Serpinb8	chr1	8.68481	0.42327	8.262	0.00054
B3gnt8	chr7	8.62315	0.36629	8.257	0.00027
Tuba3b	chr6	9.75417	1.50888	8.245	0.0084
Dnajc5b	chr3	8.24488	0	8.245	0.00036
Runx2	chr17	8.56907	0.32896	8.240	0.0002
Pcdh9	chr14	13.003	4.76309	8.240	0.00131
Gm1653	chr3	8.23456	0	8.235	1.3E-05
Gabrr3	chr16	8.23072	0	8.231	0.00027
Mgat4c	chr10	8.53306	0.30836	8.225	4.3E-05
Bmp15	chrX	17.0552	8.83416	8.221	0.00275
Gm2101	chrX	8.21074	0	8.211	1.8E-05
Gm2165	chrX	8.20998	0	8.210	1.6E-05
Gm3669	chrX	8.20606	0	8.206	3.3E-05
Bcl2l10	chr9	15.7386	7.54397	8.195	0.00263
Gm6578	chr6	8.59088	0.40377	8.187	0.00043
Wnt7a	chr6	8.4853	0.30836	8.177	6.3E-05
Bhlhe40	chr6	10.7806	2.60605	8.175	0.00499
Magea10	chrX	11.9399	3.77138	8.169	0.01584
Rex2	chr4	11.4347	3.26848	8.166	0.02028
Krt71	chr15	8.16272	0	8.163	7.9E-05
Plac1l	chr19	14.0684	5.91289	8.156	0.02761
2210019I11Rik	chr5	8.14663	0	8.147	4.2E-05
Rassf5	chr1	12.1034	3.96499	8.138	0.00625
Sh2d4b	chr14	8.46219	0.32896	8.133	0.00012
Myh15	chr16	8.11611	0	8.116	7.2E-05
Ggt1	chr10	9.5526	1.44424	8.108	0.00265
Grm8	chr6	9.98287	1.89023	8.093	0.0268
Fam155a	chr8	10.8859	2.80104	8.085	0.01185
Il23r	chr6	9.29791	1.22357	8.074	0.00882
Gm4981	chr10	10.0035	1.93961	8.064	0.03048
Tcl1b3	chr12	10.4206	2.3632	8.057	0.02556
Gm5134	chr10	11.0577	3.01106	8.047	0.0171
AW495222	chr13	8.67668	0.63731	8.039	0.00013
Lrrc8e	chr8	12.9987	4.97031	8.028	0.00579
Igfbp7	chr5	9.82673	1.81543	8.011	0.0472
Mbd3l2	chr9	11.8404	3.8302	8.010	0.03136
Gnrh1	chr14	10.1454	2.13875	8.007	0.03816
Gm11202	chr11	9.98508	1.98544	8.000	0.0336
Fbxo17	chr7	8.31837	0.32896	7.989	0.00011
Zfp296	chr7	12.2246	4.24168	7.983	0.01994
Rab3b	chr4	11.6883	3.71357	7.975	0.04994
Eif4e1b	chr13	16.0378	8.07461	7.963	0.00473
Asb4	chr6	8.79419	0.86963	7.925	0.00143
Adam32	chr8	8.77727	0.85728	7.920	0.01064
4930404A10Rik	chr11	9.61102	1.69833	7.913	0.04311
0610040J01Rik	chr5	10.419	2.53192	7.887	0.0193
Car9	chr4	11.7978	3.91598	7.882	0.00649
Zfp534	chr4	9.62628	1.76009	7.866	0.00523
Spock3	chr8	9.59175	1.72878	7.863	0.04485

4931428L18Rik	chr1	11.8458	3.98444	7.861	0.01812
Ldhd	chr8	11.2196	3.38003	7.840	0.0242
Phf15	chr11	14.5317	6.69229	7.839	0.00316
Rimbp2	chr5	9.79828	1.96806	7.830	0.04219
Usp29	chr7	11.4046	3.58856	7.816	0.04082
Il2ra	chr2	9.52057	1.71893	7.802	0.04522
Vmn2r51	chr7	8.16745	0.36629	7.801	0.00169
Frmpd4	chrX	8.16315	0.36629	7.797	0.00189
Slc9a3	chr13	11.8351	4.03978	7.795	0.01858
Il7	chr3	11.3528	3.59079	7.762	0.02476
Tbx19	chr1	10.6398	2.89777	7.742	0.03886
Ccdc42	chr11	8.39497	0.66229	7.733	0.00697
Ifitm6	chr7	9.99563	2.27294	7.723	0.02026
Nlrp4e	chr7	13.6434	5.92539	7.718	0.00273
Acot3	chr12	11.0237	3.3059	7.718	0.01649
Grm1	chr10	8.57228	0.85728	7.715	0.00836
Krt76	chr15	9.30506	1.61065	7.694	0.04014
Rgs2	chr1	15.5039	7.81237	7.692	0.00158
Fbxw16	chr9	14.5894	6.91422	7.675	0.00031
Slc16a13	chr11	10.9218	3.24851	7.673	0.03731
Zfp600	chr4	10.8387	3.18495	7.654	0.02455
Chst7	chrX	9.31588	1.66859	7.647	0.04249
Rhobtb1	chr10	11.9036	4.2896	7.614	0.01008
S1pr3	chr13	11.3743	3.77	7.604	0.03915
Myo1a	chr10	8.24509	0.64169	7.603	0.00692
Psg20	chr7	9.24366	1.64361	7.600	0.01889
4921536K21Rik	chr11	9.55709	2.00798	7.549	0.02152
Rasa4	chr5	13.2522	5.72929	7.523	0.00033
Gm19865	chr13	9.11524	1.61043	7.505	0.02283
Gm5177	chr10	10.8633	3.39358	7.470	0.01966
C130073F10Rik	chr4	9.37092	1.90643	7.464	0.03449
Ccdc69	chr11	14.5455	7.08286	7.463	0.00809
Tctex1d1	chr4	9.87608	2.41855	7.458	0.01997
Adcyap1r1	chr6	10.0119	2.5698	7.442	0.01087
Epha3	chr16	9.10502	1.66859	7.436	0.04339
E330034G19Rik	chr14	15.046	7.61483	7.431	5.9E-05
Scg3	chr9	12.1949	4.7757	7.419	0.024
Abcb5	chr12	8.57936	1.16563	7.414	0.00863
Tmem169	chr1	11.8656	4.45415	7.411	0.04792
AA619741	chr1	12.3046	4.90835	7.396	5.3E-07
Prkd2	chr7	12.3853	4.99282	7.393	0.01709
Mab21l2	chr3	8.75433	1.41129	7.343	0.01475
Tmem171	chr13	9.69485	2.36609	7.329	0.03397
Txndc2	chr17	12.5191	5.19102	7.328	0.0033
Slc2a10	chr2	9.00593	1.68743	7.319	0.02452
Edar	chr10	8.8265	1.5363	7.290	0.02184
Plce1	chr19	13.6508	6.36372	7.287	0.0019
Pcdh10	chr3	9.46687	2.18172	7.285	0.04785
Ak5	chr3	8.99105	1.73129	7.260	0.02642
Zar1	chr5	15.443	8.19034	7.253	0.00692
Nr2e1	chr10	13.1596	5.90835	7.251	0.00309
Heatr7b2	chr15	9.27627	2.02729	7.249	0.02211
Gm1078	chr7	12.2212	4.97854	7.243	0.0246
Ngef	chr1	10.9344	3.69587	7.239	0.00749
Tnfsf11	chr14	9.36309	2.13875	7.224	0.04501
Otx1	chr11	10.4365	3.21339	7.223	0.03773
Rhbg	chr3	9.54508	2.32488	7.220	0.02601

Fam188b	chr6	8.83218	1.61703	7.215	0.02163
Mctp1	chr13	8.26337	1.08233	7.181	0.02141
Rapgef3	chr15	10.1135	2.9363	7.177	0.03828
Lhx2	chr2	8.88393	1.73129	7.153	0.0312
Tcea3	chr4	12.2397	5.09303	7.147	0.04009
Gm19461	chr1	9.31613	2.1723	7.144	0.00336
Gm11475	chr2	9.0717	1.92873	7.143	0.03608
Slc6a4	chr11	10.5947	3.45904	7.136	0.03705
Igh	chr12	13.1358	6.02282	7.113	0.00461
Cdo1	chr18	13.8743	6.76847	7.106	0.00215
Insm1	chr2	12.4823	5.37842	7.104	0.00618
Rfpl4	chr7	13.8513	6.76539	7.086	0.04938
Gm7694	chr1	11.1916	4.11689	7.075	0.04171
Gm13152	chr4	11.5187	4.45084	7.068	0.0166
AA792892	chr5	12.8849	5.82924	7.056	0.00953
Prr5l	chr2	12.4038	5.35139	7.052	0.00127
Ptger4	chr15	8.79208	1.74462	7.047	0.031
Fbxw21	chr9	14.9764	7.94856	7.028	0.00106
Cldn26	chr16	9.6763	2.65211	7.024	0.04639
Nat14	chr7	11.927	4.90452	7.022	0.04363
Ddit4	chr10	13.8551	6.83349	7.022	0.01511
Meis2	chr2	14.8273	7.8177	7.010	0.00089
Grasp	chr15	9.28055	2.27272	7.008	0.01305
Cd300lf	chr11	8.25069	1.26646	6.984	0.03116
Fbxw13	chr9	13.1089	6.12939	6.979	0.02794
Ehf	chr2	14.2051	7.23728	6.968	0.00213
Slc4a8	chr15	14.0246	7.08138	6.943	0.00286
Fam151a	chr4	13.0299	6.09459	6.935	0.01268
Ggta1	chr2	13.64	6.7116	6.928	4.9E-06
4933417E11Rik	chr1	8.9266	2.02162	6.905	0.04923
Gsdmc4	chr15	9.60958	2.72637	6.883	0.02211
AA536875	chr14	11.7175	4.83689	6.881	0.00335
Slc12a8	chr16	8.39045	1.52394	6.867	0.04412
Krt12	chr11	10.4947	3.63923	6.855	0.03458
Gm13247	chr4	8.30702	1.4821	6.825	0.04286
Has3	chr8	11.7392	4.93992	6.799	0.02044
Gm19784	chr18	9.87797	3.08425	6.794	0.04779
Nwd1	chr8	8.3299	1.5363	6.794	0.01324
Slc45a3	chr1	15.955	9.18204	6.773	3E-06
Mesp2	chr7	10.0496	3.3059	6.744	0.04765
Prelp	chr1	10.9673	4.22538	6.742	0.04875
Slco3a1	chr7	14.2261	7.54246	6.684	0.00245
D6Ertd527e	chr6	15.0667	8.38561	6.681	0.00016
Gm17322	chr9	8.24181	1.56128	6.681	0.04976
Gckr	chr5	9.46575	2.79038	6.675	0.04021
Lyn	chr4	9.00259	2.33313	6.669	0.04312
Tub	chr7	11.7391	5.07565	6.663	0.03148
Nlrp4b	chr7	15.0593	8.41493	6.644	0.00466
Trim60	chr8	15.2039	8.56169	6.642	0.00154
Hivep2	chr10	10.0878	3.49008	6.598	0.03803
Mro	chr18	9.45491	2.87539	6.580	0.04903
Ido2	chr8	8.65832	2.08522	6.573	0.02926
Stard13	chr5	14.5453	7.98464	6.561	0.00454
Serpinc1	chr1	12.2402	5.68084	6.559	0.01586
Gm10523	chr18	10.5717	4.0184	6.553	0.02486
Fam84a	chr12	10.9986	4.47867	6.520	0.00975
Gpx3	chr11	10.8986	4.38854	6.510	0.03875

Dppa2	chr16	11.763	5.27366	6.489	0.01107
Tspan1	chr4	9.3022	2.82779	6.474	0.04421
Tcl1b1	chr12	11.3323	4.88684	6.445	0.00882
Rfx4	chr10	8.92903	2.49393	6.435	0.0499
Usp46	chr5	15.1873	8.75866	6.429	0.00036
Tmbim1	chr1	13.9762	7.55062	6.426	7.3E-06
Gcm2	chr13	9.35698	2.93939	6.418	0.04503
Rab38	chr7	13.0728	6.6621	6.411	0.0321
Lrch1	chr14	11.5565	5.14709	6.409	0.03748
Parp8	chr13	11.0284	4.62911	6.399	0.02735
Clip2	chr5	12.7495	6.35068	6.399	0.00076
D6Ertd474e	chr6	13.2895	6.903	6.387	0.0239
Fbxl13	chr5	8.22183	1.84917	6.373	0.04556
Miox	chr15	13.869	7.5016	6.367	0.00533
Pla2g4c	chr7	16.2987	9.93451	6.364	0.00138
Gm15698	chr11	16.7141	10.3596	6.355	0.00248
Hsf5	chr11	10.2544	3.90606	6.348	0.04889
Cmklr1	chr5	10.8939	4.55035	6.344	0.0314
Dab2	chr15	12.8031	6.47055	6.333	0.00073
Syde1	chr10	9.44779	3.11689	6.331	0.02474
Kpna7	chr5	14.82	8.49497	6.325	0.00201
Pstpip1	chr9	12.2612	5.9369	6.324	0.0001
Sp110	chr1	14.5807	8.2675	6.313	3.8E-05
AU018091	chr7	10.5327	4.26406	6.269	0.00893
Jam2	chr16	10.456	4.19102	6.265	1.6E-05
Diras2	chr13	13.0641	6.80161	6.262	0.01098
Prr18	chr17	12.0875	5.83196	6.256	0.02888
Gm17575	chr3	12.9759	6.72266	6.253	0.00296
Gm17580	chr3	12.9418	6.69681	6.245	0.00277
A530040E14Rik	chr1	11.4068	5.16614	6.241	0.00388
Tmem229b	chr12	10.7916	4.56635	6.225	0.01333
Elovl7	chr13	11.498	5.27439	6.224	0.00813
Aurkc	chr7	12.9706	6.75032	6.220	0.01234
2510049J12Rik	chr6	9.00176	2.80658	6.195	0.04906
Apba2	chr7	10.3589	4.16755	6.191	0.01858
Nebl	chr2	8.4633	2.27294	6.190	0.03588
Tubb6	chr18	14.6552	8.5128	6.142	0.00369
Dnase1l3	chr14	8.91887	2.78297	6.136	0.00136
Tacc2	chr7	15.2329	9.13	6.103	0.00231
Morn4	chr19	8.5178	2.41958	6.098	0.04649
Fbxw22	chr9	13.7907	7.71833	6.072	0.00362
Dnm3	chr1	8.65398	2.58856	6.065	0.01296
Kif17	chr4	13.9641	7.92201	6.042	0.00374
Gm19757	chr6	12.4726	6.43344	6.039	0.00062
Gng3	chr19	12.0696	6.03416	6.035	0.0163
Fam84b	chr15	13.5043	7.48032	6.024	0.0015
Fbxw24	chr9	16.2818	10.267	6.015	6.8E-05
Me1	chr9	14.6954	8.68219	6.013	0.00029
Ccdc28b	chr4	11.3243	5.31329	6.011	0.02324
Zfp108	chr7	13.4574	7.45854	5.999	0.01203
Bace2	chr16	10.0817	4.08624	5.995	0.01199
Casp8	chr1	10.2108	4.27486	5.936	0.01165
Fbxw26	chr9	14.247	8.31809	5.929	0.00272
Fam161a	chr11	10.7097	4.78188	5.928	0.00032
Palm3	chr8	12.2203	6.29788	5.922	0.00613
Iqgap2	chr13	14.0838	8.19775	5.886	0.00112
Otub2	chr12	11.763	5.88005	5.883	0.02749

Atxn1	chr13	12.0304	6.15659	5.874	0.00602
Cxcl14	chr13	8.92704	3.06024	5.867	0.02784
Pld2	chr11	11.4884	5.63565	5.853	0.02036
Abcb1b	chr5	12.5831	6.74908	5.834	3.8E-05
Sh2d3c	chr2	11.383	5.5535	5.830	0.02204
Ephb4	chr5	11.0762	5.24766	5.829	0.04797
Gm1070	chr6	14.7877	8.96627	5.821	0.00399
Slc18a2	chr19	11.0959	5.30175	5.794	0.04745
Slc25a48	chr13	12.8577	7.06579	5.792	0.00121
Ninj2	chr6	12.4589	6.67092	5.788	0.00727
Calr4	chr4	13.6889	7.9084	5.780	3.5E-06
Trim61	chr8	15.775	10.0011	5.774	0.00213
Bub1b	chr2	17.1263	11.3534	5.773	1.6E-06
Rnf43	chr11	10.3902	4.62033	5.770	0.04842
Gm6150	chr10	11.2859	5.55024	5.736	0.00851
Kcnh3	chr15	10.9935	5.26207	5.731	3.7E-07
Elavl3	chr9	12.0985	6.38796	5.711	0.01331
Gbx2	chr1	12.5862	6.89007	5.696	0.00835
Slc6a6	chr6	13.0151	7.32073	5.694	0.00051
Serpinb12	chr1	10.9559	5.26406	5.692	0.03154
Cd160	chr3	11.6572	5.98927	5.668	0.00046
2610305D13Rik	chr4	12.2357	6.58189	5.654	0.02835
Lypd3	chr7	11.8961	6.25765	5.638	0.01315
Stxbp6	chr12	13.0303	7.39282	5.638	0.00388
Rph3a	chr5	13.0656	7.44807	5.617	0.00012
H1foo	chr6	16.3407	10.7321	5.609	0.00018
Rgs10	chr7	10.5553	4.96039	5.595	0.04103
Omt2a	chr9	14.4268	8.8382	5.589	0.00017
Egfr	chr11	12.8391	7.2567	5.582	0.00262
Maob	chrX	9.80274	4.22419	5.579	0.00012
Sh3rf1	chr8	12.8116	7.24211	5.569	0.00894
Ptprb	chr10	9.97315	4.40488	5.568	0.01036
Stbd1	chr5	12.5905	7.02647	5.564	0.00138
Gata3	chr2	9.38902	3.85385	5.535	0.02605
BC021767	chr3	10.0195	4.48871	5.531	0.01659
Eps8	chr6	13.9117	8.39484	5.517	0.00144
1300002K09Rik	chr4	10.5135	5.03198	5.482	0.0419
Smoc2	chr17	12.0266	6.54612	5.480	0.02637
Gdf9	chr11	17.4722	12.0127	5.459	0.00064
Prlr	chr15	11.9942	6.54538	5.449	0.0221
6720489N17Rik	chr13	10.2433	4.79906	5.444	0.00876
Omt2b	chr9	14.5971	9.15593	5.441	0.00254
Vwc2	chr11	8.50053	3.06024	5.440	0.04558
Nr5a2	chr1	11.5347	6.1042	5.431	0.00163
Robo3	chr9	10.2806	4.8599	5.421	0.00729
Bscl2	chr19	13.1631	7.77504	5.388	0.01558
Glis1	chr4	10.0147	4.63501	5.380	0.01866
St6gal1	chr16	12.2745	6.89536	5.379	0.00471
Dhrs3	chr4	10.4792	5.10162	5.378	0.04523
Btg4	chr9	15.2904	9.92887	5.361	7.4E-05
Birc3	chr9	15.2151	9.8577	5.357	0.0013
Tgfb2	chr1	13.6902	8.33338	5.357	0.00079
Ipcef1	chr10	13.587	8.2316	5.355	0.00736
2210408I21Rik	chr13	9.45482	4.15401	5.301	0.00315
Dnahc7a	chr1	9.94701	4.65976	5.287	0.00087
Nlgn1	chr3	10.4438	5.15998	5.284	0.0208
Fbxw15	chr9	15.415	10.1408	5.274	0.00395

Tbx4	chr11	12.9582	7.69384	5.264	0.00229
Sulf2	chr2	12.4419	7.1825	5.259	0.01598
Slc6a7	chr18	12.853	7.5982	5.255	7.4E-07
Zfp608	chr18	10.1628	4.91404	5.249	0.02298
Col13a1	chr10	9.75672	4.51923	5.237	0.00025
Prkg2	chr5	8.78255	3.54672	5.236	0.02166
Zfp109	chr7	10.7269	5.4994	5.228	0.03624
Pdlim1	chr19	11.8748	6.64743	5.227	0.01479
Prickle3	chrX	11.6409	6.42616	5.215	0.00052
Lmo1	chr7	12.1149	6.91125	5.204	0.01307
Vrtn	chr12	13.5269	8.32892	5.198	0.00457
D5Ertd577e	chr5	12.9699	7.78616	5.184	0.00548
Sap30	chr8	12.0895	6.90823	5.181	0.00317
Mest	chr6	13.1272	7.95093	5.176	0.00118
AU018829	chr5	9.20936	4.04907	5.160	0.00998
Fabp5	chr3	11.3901	6.23476	5.155	0.00699
Gm3183	chr5	9.19871	4.04907	5.150	0.00999
Efcab3	chr11	10.464	5.31972	5.144	0.00525
Cachd1	chr4	12.2736	7.13001	5.144	1.7E-05
Emp2	chr16	11.7937	6.65142	5.142	0.00458
Ampd2	chr3	9.85184	4.74534	5.107	0.00283
Ptpn21	chr12	15.7156	10.6145	5.101	7.7E-05
Sspn	chr6	12.6768	7.57626	5.101	0.0108
Trim36	chr18	11.5386	6.43875	5.100	0.04314
Gm10509	chr17	10.392	5.29315	5.099	0.00021
Nsun4	chr4	13.9628	8.86447	5.098	0.00598
Cdc42se1	chr3	11.8881	6.79666	5.091	0.00118
Mb21d1	chr9	12.5096	7.43868	5.071	0.00171
Stom	chr2	14.293	9.22423	5.069	0.01035
Sall4	chr2	15.4901	10.4239	5.066	2.4E-05
A430033K04Rik	chr5	15.5206	10.4559	5.065	0.00028
Gprc5c	chr11	11.1118	6.05448	5.057	0.0025
Speer5-ps1	chr10	13.4935	8.43757	5.056	0.00438
AU022751	chrX	14.533	9.48488	5.048	4.6E-06
E330014E10Rik	chr5	12.7892	7.74446	5.045	0.00182
Gucy2c	chr6	10.2913	5.2502	5.041	0.01153
AU016765	chr17	11.3886	6.35571	5.033	0.02509
A4galt	chr15	9.22996	4.20341	5.027	0.00354
Bmp6	chr13	13.5379	8.51427	5.024	0.00645
Fank1	chr7	10.9745	5.95111	5.023	0.01742
Rimkla	chr4	13.9148	8.89465	5.020	1.7E-06
BC017612	chr9	11.2355	6.21592	5.020	0.00084
Dnajb4	chr3	14.3406	9.3377	5.003	0.00134
Mos	chr4	12.9657	7.97366	4.992	9.7E-06
Gm2104	chr14	12.3594	7.3677	4.992	0.00146
Cphx	chr14	12.3531	7.36418	4.989	0.00151
Gm2135	chr14	12.3599	7.3712	4.989	0.00143
Rad51ap2	chr12	9.58095	4.59739	4.984	0.02457
Slc7a8	chr14	9.31434	4.3319	4.982	0.00017
C86187	chr7	16.4267	11.4654	4.961	0.00026
Fam46b	chr4	14.6991	9.74683	4.952	0.00066
Oas1e	chr5	15.0497	10.1166	4.933	0.00037
Akap2	chr4	13.4954	8.56528	4.930	0.00358
Mpzl1	chr1	13.3601	8.4326	4.928	0.00689
Orai1	chr5	9.68402	4.76771	4.916	0.00557
Tmem132b	chr5	12.1567	7.24407	4.913	0.01062
Dgkk	chrX	12.1183	7.21111	4.907	0.00172

Pcgf1	chr6	13.4524	8.54678	4.906	0.0001
Plat	chr8	17.278	12.3818	4.896	0.00032
Gm10248	chr14	10.0356	5.14289	4.893	0.01177
Afap1l2	chr19	13.5116	8.63882	4.873	0.00079
BB557941	chr2	9.53239	4.67622	4.856	9.2E-06
Npm2	chr14	15.7163	10.8624	4.854	0.013
Spint1	chr2	11.4309	6.57901	4.852	0.00032
Cdh3	chr8	11.6714	6.83251	4.839	0.00051
Ppap2b	chr4	12.604	7.76615	4.838	0.01252
Caln1	chr5	11.2793	6.44462	4.835	0.00768
Cmya5	chr13	12.8555	8.02871	4.827	0.00019
Tuba3a	chr6	13.529	8.70387	4.825	0.0023
Sytl2	chr7	12.5421	7.72495	4.817	1.2E-06
Tek	chr4	10.4314	5.62299	4.808	0.02702
Gm3259	chr5	10.8727	6.0669	4.806	0.00637
Gm3286	chr5	10.8725	6.0669	4.806	0.00637
Obscn	chr11	11.0179	6.22255	4.795	0.00346
Kif26b	chr1	9.30599	4.51406	4.792	0.03935
Cdr2	chr7	13.7975	9.00947	4.788	0.0016
Sox8	chr17	11.9096	7.1252	4.784	0.00689
Spata21	chr4	10.8426	6.06727	4.775	0.00558
Fam46c	chr3	15.8147	11.0401	4.775	0.00018
Dok4	chr8	11.3232	6.5626	4.761	0.00437
Eef2k	chr7	14.9946	10.2588	4.736	0.00012
AA545190	chr6	11.3041	6.5707	4.733	0.00033
B4galnt3	chr6	10.7994	6.07177	4.728	3.1E-05
Eogt	chr6	9.26319	4.54184	4.721	0.01784
Ccdc82	chr9	19.7362	15.0222	4.714	2.2E-05
Ubash3b	chr9	12.5613	7.8528	4.708	0.0017
Ntn1	chr11	10.8887	6.18718	4.701	0.01189
Dpp6	chr5	10.91	6.2109	4.699	0.0181
4933431E20Rik	chr3	10.3322	5.63584	4.696	0.01383
Zbed3	chr13	17.9856	13.2895	4.696	3.3E-08
Unc5c	chr3	14.0199	9.32546	4.694	0.00159
Ets2	chr16	12.2061	7.51875	4.687	0.0208
Arg2	chr12	12.8987	8.21905	4.680	0.00532
Fbxw18	chr9	15.1061	10.4267	4.679	5.5E-05
Cnpy4	chr5	14.3018	9.62527	4.676	0.0044
Pin1-ps1	chr2	11.4903	6.83128	4.659	0.01487
Arntl2	chr6	10.842	6.18719	4.655	0.00084
Pld1	chr3	15.1929	10.5409	4.652	2E-06
Fbxw27	chr9	11.6585	7.00676	4.652	0.0121
Abca9	chr11	9.27026	4.62396	4.646	0.02033
Eif4e3	chr6	13.9645	9.32868	4.636	0.00249
Pdpn	chr4	12.5871	7.95347	4.634	0.00968
BC030307	chr10	10.7249	6.09152	4.633	0.03843
E330017A01Rik	chr16	14.0025	9.38082	4.622	0.00376
Gpd2	chr2	10.505	5.88863	4.616	0.0273
Rnf152	chr1	10.0289	5.43171	4.597	0.00099
Ier5l	chr2	10.9599	6.37279	4.587	0.0119
Pfkp	chr13	10.1845	5.60427	4.580	0.0339
Gm7609	chr1	10.0863	5.51119	4.575	0.02145
Paqr4	chr17	11.1203	6.56482	4.556	0.00928
Neil3	chr8	13.3083	8.7534	4.555	0.008
Gm7104	chr12	10.697	6.14561	4.551	0.0411
Lama5	chr2	12.3131	7.77296	4.540	0.00336
Rdh12	chr12	10.5872	6.05762	4.530	0.04313

Nin	chr12	15.3771	10.8487	4.528	4.8E-05
E030030I06Rik	chr10	9.59558	5.06801	4.528	0.01214
Gm3139	chr5	8.85378	4.32989	4.524	0.00315
Tdrd12	chr7	13.4945	8.9741	4.520	0.00062
Fgfr2	chr7	12.3346	7.81422	4.520	0.00391
Nedd9	chr13	11.1307	6.61654	4.514	0.0181
Armc2	chr10	10.0995	5.58633	4.513	0.04428
Nrp1	chr8	11.7093	7.20033	4.509	0.00434
Egf	chr3	11.1827	6.68705	4.496	8.8E-05
Ermp1	chr19	16.0289	11.545	4.484	0.00026
Tshz2	chr2	11.419	6.93539	4.484	0.00549
Fzd7	chr1	10.2988	5.82281	4.476	0.04389
Cdh2	chr18	11.4227	6.94722	4.475	0.00646
Khdc1b	chr1	16.3565	11.8826	4.474	0.00125
Glrx	chr13	13.8858	9.41407	4.472	0.00064
Slc25a35	chr11	11.7818	7.31703	4.465	0.02863
Arhgef39	chr4	13.074	8.61047	4.464	0.00126
Btk	chrX	10.8073	6.34899	4.458	0.01125
Twf2	chr9	11.0172	6.56202	4.455	0.01233
Rapgef5	chr12	13.9284	9.474	4.454	0.00127
Cdk20	chr13	13.4622	9.02098	4.441	3.7E-06
Myh11	chr16	14.2105	9.76992	4.441	0.00134
Samd7	chr3	12.2722	7.83203	4.440	0.00186
1190002N15Rik	chr9	13.8909	9.45334	4.438	3.6E-05
Gm7120	chr13	9.45033	5.01587	4.434	0.03942
Trib2	chr12	10.5981	6.16588	4.432	0.00022
Khdc3	chr9	14.0715	9.63945	4.432	0.0024
Parp12	chr6	15.9491	11.5252	4.424	0.00012
Plekha2	chr8	10.2315	5.81141	4.420	0.02204
Mat1a	chr14	10.8205	6.40108	4.419	0.00614
B3gnt4	chr5	9.37949	4.96097	4.419	0.00646
Rapgef4	chr2	10.5974	6.19018	4.407	0.01074
Kif23	chr9	15.7504	11.3432	4.407	1.4E-07
Nab2	chr10	11.5062	7.09921	4.407	0.00463
Gdap1	chr1	14.2783	9.87541	4.403	0.00058
LOC101055764	chr13	8.3835	3.98736	4.396	0.01619
Slc38a8	chr8	10.7046	6.31187	4.393	0.00761
Plekhg3	chr12	14.1717	9.78902	4.383	0.00417
Rnf185	chr11	14.6911	10.3175	4.374	2.5E-05
Pfn4	chr12	9.87837	5.51345	4.365	0.01403
Siah2	chr3	13.9365	9.58437	4.352	0.00826
6330411D24Rik	chr4	12.3481	7.99751	4.351	1.1E-05
Dusp18	chr11	11.2012	6.85474	4.346	0.00975
Rbm38	chr2	13.8688	9.53335	4.335	0.00607
Zfp957	chr14	13.6032	9.27574	4.327	2.3E-05
Dclre1a	chr19	13.6184	9.3145	4.304	1.6E-05
Tubb2a	chr13	15.3292	11.0285	4.301	9.4E-06
Vwde	chr6	10.3627	6.06859	4.294	0.03677
Gli3	chr13	12.5917	8.31382	4.278	0.00055
Ltc4s	chr11	8.60353	4.32642	4.277	0.00651
Thsd7a	chr6	12.5387	8.26577	4.273	0.00851
Wdr65	chr4	8.65326	4.38473	4.269	0.0456
Arrdc1	chr2	13.2172	8.94918	4.268	3.9E-05
Ramp3	chr11	9.94949	5.68158	4.268	2.8E-05
Wdr20a	chr12	14.7824	10.5191	4.263	0.00154
Grn	chr11	11.5158	7.2656	4.250	0.03189
Stk32b	chr5	11.6176	7.36905	4.249	0.01721

Rnf24	chr2	13.6132	9.37336	4.240	6E-06
Ddx11	chr17	13.6102	9.37652	4.234	0.00042
Mpped1	chr15	11.536	7.30848	4.228	0.00533
Hecw2	chr1	10.6942	6.46708	4.227	0.02522
AU023762	chr9	9.11737	4.89234	4.225	0.00486
Tmtc1	chr6	14.4135	10.1897	4.224	0.00061
Tnfaip8	chr18	13.442	9.22416	4.218	1.9E-06
Gtf2a1l	chr17	10.6854	6.47055	4.215	0.01195
Cpsf4l	chr11	14.0884	9.87468	4.214	0.00232
Bach2	chr4	13.098	8.89026	4.208	0.00055
Slc23a3	chr1	9.01127	4.80567	4.206	0.04605
Vamp8	chr6	11.6366	7.43638	4.200	0.01368
Aspm	chr1	16.2896	12.1045	4.185	0.00059
Cpxm2	chr7	10.0525	5.86993	4.183	0.03921
0610040F04Rik	chr6	10.1375	5.97784	4.160	0.01473
BC067074	chr13	9.93988	5.79803	4.142	0.02336
Tet3	chr6	16.9945	12.8528	4.142	2.2E-05
Trim75	chr8	14.5059	10.3644	4.141	0.00084
Prex2	chr1	10.7062	6.56521	4.141	0.01675
Dusp10	chr1	11.8876	7.74767	4.140	0.02291
Esyt1	chr10	12.7053	8.56823	4.137	0.00168
Ahdc1	chr4	12.3767	8.24052	4.136	5.9E-05
Tubg2	chr11	12.6709	8.54154	4.129	0.0117
4831426I19Rik	chr12	12.4519	8.32913	4.123	0.00677
Tulp3	chr6	12.7874	8.66566	4.122	0.00412
Magi2	chr5	10.5503	6.43002	4.120	0.00596
Tspan9	chr6	12.9943	8.87917	4.115	0.00167
Kdm2b	chr5	15.0893	10.9751	4.114	0.00089
Ttll11	chr2	12.7252	8.61752	4.108	0.00842
Ica1	chr6	12.0871	7.98111	4.106	0.00185
Fam63a	chr3	13.65	9.54448	4.106	0.0005
Cnst	chr1	14.7849	10.6796	4.105	8.5E-07
Styk1	chr6	12.7761	8.67086	4.105	0.00918
Calr3	chr8	11.003	6.90149	4.102	0.00175
Folr4	chr9	12.8179	8.71865	4.099	0.00264
Hhex	chr19	11.9002	7.80981	4.090	0.00501
Cldn8	chr16	10.4087	6.31881	4.090	0.02631
Lamb1	chr12	16.8872	12.8013	4.086	0.00138
Fmo5	chr3	9.85342	5.77101	4.082	0.02088
Svopl	chr6	10.8982	6.8203	4.078	0.03684
Gfra1	chr19	11.7306	7.65542	4.075	0.01372
Bdh1	chr16	11.4401	7.36533	4.075	0.00657
Ankrd44	chr1	15.0349	10.9608	4.074	0.00216
Pcyt1b	chrX	11.9906	7.91811	4.072	0.00106
Nudt7	chr8	12.19	8.11968	4.070	0.02827
Fam132a	chr4	9.82493	5.75852	4.066	0.01369
Itprip	chr19	11.2206	7.15871	4.062	0.01999
Gm4907	chrX	9.66418	5.60299	4.061	0.01595
Tmem164	chrX	13.2596	9.19965	4.060	3.8E-05
Egfl6	chrX	10.2194	6.16041	4.059	0.00029
Tuba4a	chr1	13.5822	9.52502	4.057	0.00095
Fhod3	chr18	14.622	10.5752	4.047	5E-05
Kdm6b	chr11	14.4798	10.4344	4.045	9.6E-06
Pde3b	chr7	12.8936	8.85667	4.037	0.01958
Fgf10	chr13	10.1919	6.16011	4.032	0.0138
Trpm6	chr19	8.88248	4.85768	4.025	0.01134
Rgs3	chr4	12.6805	8.65829	4.022	0.00498

Ppt2	chr17	12.6464	8.62552	4.021	4.1E-05
Chst11	chr10	11.2726	7.25255	4.020	0.01009
Rhpn2	chr7	16.0156	11.9959	4.020	7.3E-05
Rassf3	chr10	13.4392	9.42162	4.018	0.00151
Ern1	chr11	15.7314	11.714	4.017	0.00036
Gm5434	chr12	12.5938	8.57789	4.016	0.00216
Astl	chr2	13.9776	9.96287	4.015	0.00011
Tspan14	chr14	13.7838	9.7754	4.008	0.00024
Plk1	chr7	13.4803	9.47474	4.006	0.00025
Grhl1	chr12	12.9141	8.91315	4.001	0.00185
Camk4	chr18	8.30151	4.3059	3.996	0.00413
Noxo1	chr17	10.5609	6.56678	3.994	0.03075
1700021K19Rik	chr16	15.3229	11.3295	3.993	4.7E-06
Smagp	chr15	13.4041	9.4141	3.990	0.00191
Pik3cd	chr4	14.7544	10.7682	3.986	2.1E-05
Hectd2	chr19	11.8289	7.85325	3.976	0.00102
Tiam1	chr16	16.2691	12.2949	3.974	6.6E-06
Mfng	chr15	11.1764	7.20366	3.973	1.5E-05
Txndc5	chr13	13.1904	9.23462	3.956	0.00075
Gm13752	chr2	11.5901	7.64001	3.950	0.01058
Adcy1	chr11	10.0732	6.12639	3.947	0.00486
Cep57l1	chr10	12.1624	8.22115	3.941	3.1E-06
Fsd1	chr17	11.4141	7.47434	3.940	0.00577
Slc41a2	chr10	11.9669	8.02968	3.937	0.00773
Galnt6	chr15	12.1935	8.26012	3.933	0.00363
Slco5a1	chr1	10.4955	6.56464	3.931	0.01024
Il10rb	chr16	10.185	6.25523	3.930	0.02536
Gpd1l	chr9	13.5475	9.62282	3.925	0.00049
Gse1	chr8	14.723	10.7998	3.923	0.00014
Kifc3	chr8	12.2037	8.28099	3.923	6.8E-05
Xrcc3	chr12	11.545	7.6287	3.916	0.01023
Pttg1	chr11	13.1518	9.24373	3.908	0.01089
Shcbp1	chr8	11.5937	7.68693	3.907	0.02604
Gpr68	chr12	11.6106	7.70732	3.903	0.00273
E2f2	chr4	12.5037	8.60344	3.900	0.00018
Galm	chr17	12.8947	9.00221	3.892	0.00734
Fbxo40	chr16	12.1915	8.30361	3.888	0.00055
D2Ertd750e	chr2	13.7798	9.89609	3.884	1.3E-05
Bcas1	chr2	11.4227	7.54059	3.882	9E-05
Gpr63	chr4	9.92463	6.04498	3.880	0.01292
Rspo2	chr15	14.7882	10.9089	3.879	2.5E-05
Dnmt1	chr9	17.9072	14.0451	3.862	4.1E-05
Fam72a	chr1	10.5525	6.69774	3.855	0.00913
Klf16	chr10	12.0195	8.16582	3.854	0.00829
Tmem117	chr15	11.621	7.77292	3.848	0.03204
Midn	chr10	13.4464	9.60368	3.843	0.00594
Flnb	chr14	14.3643	10.5243	3.840	0.0006
Itga8	chr2	9.9901	6.15125	3.839	0.00053
Avpi1	chr19	9.45083	5.61325	3.838	0.00649
Fbxo5	chr10	12.4438	8.61818	3.826	0.00061
BC021891	chr8	9.08042	5.25648	3.824	0.01258
LOC100503720	chr5	11.4249	7.61038	3.815	0.01129
Prkg1	chr19	11.1634	7.34921	3.814	0.00902
Fam123b	chrX	10.7488	6.93839	3.810	0.04804
Cacna1h	chr17	12.6353	8.83049	3.805	0.00578
Cplx2	chr13	10.7718	6.96942	3.802	0.00141
2700029M09Rik	chr8	14.9197	11.1175	3.802	4.3E-05

Serpini1	chr3	12.0772	8.27542	3.802	0.00953
Ccnb1	chr13	15.6037	11.8054	3.798	0.00044
Dclk2	chr3	15.4806	11.6843	3.796	0.00018
Zfp78	chr7	10.8502	7.056	3.794	0.02475
Angptl2	chr2	11.3573	7.56485	3.792	0.01418
Depdc7	chr2	14.6144	10.8246	3.790	0.00097
Sorbs1	chr19	13.8285	10.0501	3.778	0.00319
Sgsm1	chr5	12.7103	8.94195	3.768	0.0019
Flrt1	chr19	8.85587	5.09534	3.761	0.00288
Tmem38a	chr8	10.4889	6.73685	3.752	0.0239
Slain1	chr14	13.6915	9.94042	3.751	3.5E-07
Ggct	chr6	13.0464	9.2968	3.750	0.00054
Ptgis	chr2	10.0577	6.31916	3.739	0.01667
Tbc1d14	chr5	15.506	11.7718	3.734	5.3E-05
Inhba	chr13	9.85019	6.11837	3.732	0.00842
Elovl1	chr4	11.3287	7.59712	3.732	0.03091
Ppap2a	chr13	11.0375	7.3091	3.728	0.02471
AI987944	chr7	13.6161	9.89238	3.724	7.7E-05
Usp43	chr11	11.1234	7.40843	3.715	0.00533
Tax1bp3	chr11	11.2012	7.49878	3.702	0.02587
Klhdc1	chr12	11.8797	8.19255	3.687	0.00275
Emilin2	chr17	12.5858	8.9009	3.685	0.0085
Yap1	chr9	14.8301	11.1496	3.681	0.00108
Rab32	chr10	11.2447	7.56451	3.680	0.03009
Nod2	chr8	11.1576	7.48032	3.677	0.02383
Gata4	chr14	10.2643	6.59367	3.671	0.01794
Zfp57	chr17	16.3479	12.6775	3.670	0.00107
Nav3	chr10	13.2271	9.56596	3.661	0.00106
Slc39a10	chr1	15.0365	11.3765	3.660	0.00031
Lrp1	chr10	14.2037	10.5481	3.656	5.2E-05
Card6	chr15	11.0333	7.38184	3.651	0.0477
Cmpk2	chr12	12.1492	8.49786	3.651	3.4E-05
Onecut1	chr9	9.12832	5.47701	3.651	0.00431
Heatr2	chr5	12.8636	9.21323	3.650	0.02058
Plxnc1	chr10	12.4378	8.78828	3.650	0.00278
Fbp1	chr13	10.4644	6.82413	3.640	0.00481
Fa2h	chr8	11.4142	7.77555	3.639	0.0058
Fpgs	chr2	12.8556	9.21747	3.638	0.00477
BC030476	chr15	13.0849	9.45107	3.634	0.00428
Tmem109	chr19	12.433	8.80575	3.627	0.00077
Fbxo43	chr15	12.9978	9.37281	3.625	0.00077
Mmp2	chr8	11.045	7.42133	3.624	0.02296
Spin1	chr13	18.814	15.1907	3.623	9.1E-07
Efhb	chr17	9.53816	5.92218	3.616	0.03245
Slbp	chr5	16.1494	12.5369	3.613	1E-05
Elof1	chr9	13.4564	9.85363	3.603	0.00651
Tenc1	chr15	11.1594	7.5592	3.600	0.01845
Ccndbp1	chr2	13.0562	9.45925	3.597	0.0006
Wee2	chr6	15.4274	11.8334	3.594	0.00044
Disc1	chr8	11.6192	8.02552	3.594	0.02769
Abca13	chr11	12.0988	8.50589	3.593	0.0217
Slco1a5	chr6	8.89098	5.29883	3.592	0.01688
Fbxw4	chr19	12.0723	8.48622	3.586	0.01174
Arpc1b	chr5	13.017	9.43573	3.581	0.00356
Prdx2	chr8	14.1595	10.5934	3.566	0.0001
2010002M12Rik	chr19	13.9899	10.4261	3.564	3.5E-06
Runx1t1	chr4	13.1154	9.55308	3.562	0.00297

Whamm	chr7	13.4683	9.90748	3.561	0.00313
Tgds	chr14	11.9379	8.37757	3.560	0.0269
Lrp1b	chr2	9.30772	5.75252	3.555	0.0426
D030018L15Rik	chr15	11.7279	8.1746	3.553	0.03065
Papd7	chr13	15.0645	11.5128	3.552	4.9E-06
Mthfd2	chr6	13.2585	9.70762	3.551	0.00035
Tmem72	chr6	12.5514	9.00069	3.551	0.00401
Gm11426	chr11	11.1663	7.61987	3.546	0.02138
Polm	chr11	11.7916	8.24796	3.544	0.00478
Katnal2	chr18	11.3814	7.8398	3.542	2.4E-06
1700067K01Rik	chr8	9.70954	6.1841	3.525	0.03089
Atp2c2	chr8	13.226	9.70459	3.521	0.00073
Arxes1	chrX	8.84573	5.32721	3.519	0.00023
Cgn	chr3	13.6188	10.1046	3.514	5.1E-05
Gpr180	chr14	10.8793	7.37038	3.509	0.01237
Mlxipl	chr5	11.8058	8.2983	3.508	0.00276
Uxs1	chr1	12.6431	9.1374	3.506	0.01515
Epha10	chr4	9.21623	5.71551	3.501	0.03545
Fbxo34	chr14	14.8582	11.36	3.498	1.1E-06
Carhsp1	chr16	13.8849	10.3868	3.498	1.7E-05
Fam167a	chr14	11.7411	8.24817	3.493	0.0091
Htr5a	chr5	12.3352	8.84471	3.490	0.00021
Patl2	chr2	11.2903	7.80067	3.490	0.00948
Slc23a2	chr2	15.5475	12.0584	3.489	0.00037
Clvs1	chr4	11.7427	8.25472	3.488	0.00255
Macrod2	chr2	12.8797	9.39425	3.485	0.00752
C1qb	chr4	8.50652	5.02417	3.482	0.00523
Rnf25	chr1	11.5026	8.02469	3.478	0.00222
Slc30a3	chr5	14.7531	11.2757	3.477	3.5E-05
Loh12cr1	chr6	10.7231	7.24828	3.475	0.03888
8430410A17Rik	chr6	13.7253	10.2549	3.470	0.00331
AY512915	chr6	12.1373	8.66778	3.470	0.00119
Lsm10	chr4	11.559	8.09088	3.468	0.00029
Gm13157	chr4	9.67418	6.20835	3.466	0.04918
Zp3	chr5	15.9633	12.4976	3.466	0.00191
2010107G23Rik	chr10	14.1485	10.685	3.464	0.00138
Gys1	chr7	12.2433	8.78208	3.461	0.00022
Ube2t	chr1	12.3546	8.90842	3.446	0.01743
Efna5	chr17	11.963	8.5194	3.444	0.0072
Oas1d	chr5	14.7649	11.3281	3.437	0.00763
Fgd1	chrX	11.499	8.06273	3.436	0.02223
Lpin2	chr17	15.3478	11.912	3.436	8.1E-05
Kcnq1	chr7	10.4451	7.00946	3.436	0.00235
Hist1h2aa	chr13	10.4947	7.06212	3.433	0.00662
Rassf1	chr9	10.4093	6.97882	3.431	8.9E-05
Fmn2	chr1	14.3165	10.8937	3.423	0.00015
Ksr1	chr11	11.6533	8.23286	3.420	0.03301
Efhc2	chrX	11.3276	7.90902	3.419	0.00068
Lnx2	chr5	13.9004	10.4823	3.418	2.2E-05
Creb3l4	chr3	11.1686	7.75196	3.417	0.01594
Slc15a4	chr5	12.7686	9.35217	3.416	0.00524
Gch1	chr14	11.1343	7.71829	3.416	0.00061
Bmpr1b	chr3	14.1119	10.701	3.411	0.00641
Dock5	chr14	13.9488	10.5384	3.410	0.00081
Ugt8a	chr3	9.58371	6.17497	3.409	0.00115
Itgb4	chr11	9.1999	5.80017	3.400	0.02233
Arhgap15	chr2	8.32571	4.92935	3.396	0.00807

E330012B07Rik	chr6	10.988	7.59209	3.396	0.00025
2310022B05Rik	chr8	9.71871	6.32401	3.395	0.00119
Kctd17	chr15	8.7812	5.40103	3.380	0.01931
Csrnp3	chr2	10.5896	7.21164	3.378	0.03058
4933427D06Rik	chr6	15.1517	11.7765	3.375	7.3E-05
Lzts1	chr8	10.7269	7.35593	3.371	0.00042
Gpnmb	chr6	9.70939	6.34091	3.368	0.04545
Kank2	chr9	11.6981	8.33309	3.365	0.0234
Gm13051	chr4	9.4961	6.13552	3.361	0.04963
Gpr56	chr8	12.3799	9.02448	3.355	0.00033
Sbds	chr5	13.3295	9.9808	3.349	7.4E-05
Plekhg1	chr10	13.9557	10.6131	3.343	0.00066
Plxnb1	chr9	11.6316	8.29338	3.338	0.01653
Mfsd10	chr5	10.1813	6.84396	3.337	0.00037
Tob2	chr15	13.8049	10.4687	3.336	8.1E-06
Serpine2	chr1	12.8161	9.48006	3.336	0.00473
Bcar3	chr3	14.5685	11.2382	3.330	0.00256
Hirip3	chr7	14.0408	10.7108	3.330	0.00011
Tiam2	chr17	13.8244	10.4952	3.329	0.00535
Rhd	chr4	12.0032	8.67466	3.329	0.01231
Cyp1b1	chr17	8.49273	5.16755	3.325	0.01588
Nceh1	chr3	13.0595	9.73523	3.324	1.5E-05
Vps37b	chr5	11.7063	8.3839	3.322	0.01899
Tcl1	chr12	13.8731	10.5547	3.318	0.00102
Zdhhc18	chr4	12.407	9.09019	3.317	0.0041
Cenpo	chr12	13.3742	10.0644	3.310	4.7E-06
Mtap1b	chr13	14.7708	11.462	3.309	0.00024
Phc2	chr4	13.4708	10.1636	3.307	0.0006
Adamts3	chr5	10.0029	6.70271	3.300	0.03173
Foxred2	chr15	12.3572	9.05928	3.298	0.00454
Ctbp2	chr7	13.3052	10.0076	3.298	0.00186
Camk2g	chr14	14.659	11.362	3.297	0.0011
Ccdc155	chr7	11.8158	8.51894	3.297	0.0149
Gm8801	chr17	13.6048	10.3083	3.296	0.00017
Axin2	chr11	14.5309	11.2365	3.294	0.00039
Atxn2l	chr7	15.8499	12.5602	3.290	0.00011
Bpgm	chr6	17.5782	14.2888	3.289	1.4E-05
Pstpip2	chr18	11.8744	8.58681	3.288	0.00528
Sytl4	chrX	13.4989	10.2129	3.286	0.00824
Rbm47	chr5	9.09261	5.80955	3.283	0.00191
Sfmbt1	chr14	16.0954	12.8127	3.283	1.3E-06
Trps1	chr15	14.1426	10.8607	3.282	2.9E-05
Cd177	chr7	13.7208	10.4407	3.280	9.4E-06
5830473C10Rik	chr5	8.92722	5.64728	3.280	0.01233
Fam49a	chr12	10.9426	7.664	3.279	0.00375
Dpcd	chr19	11.8234	8.54813	3.275	0.00573
Cttnbp2	chr6	9.79846	6.52756	3.271	0.002
Vav2	chr2	9.66262	6.39415	3.268	0.02247
AU040320	chr4	12.3129	9.04507	3.268	0.00446
Cttnbp2nl	chr3	14.1825	10.9168	3.266	0.00018
Ppm1e	chr11	9.58974	6.3256	3.264	0.00704
Angel2	chr1	13.3726	10.1151	3.258	0.00129
Zfp939	chr7	10.7972	7.54044	3.257	8E-05
Pecr	chr1	14.2887	11.0322	3.256	0.00013
Gyg	chr3	15.254	11.9994	3.255	0.00032
Itga9	chr9	13.2594	10.0055	3.254	0.00104
5730419I09Rik	chr6	15.1173	11.8645	3.253	0.0001

Hpcal1	chr12	11.6474	8.39958	3.248	0.00209
2810002D19Rik	chr2	8.4386	5.19102	3.248	0.00064
C230081A13Rik	chr9	15.3373	12.0904	3.247	1.9E-05
Cenpj	chr14	13.3647	10.1223	3.242	0.00186
Ptpre	chr7	12.6137	9.37324	3.240	0.00149
Lpl	chr8	11.0834	7.84354	3.240	0.00047
Cflar	chr1	10.6829	7.44341	3.240	0.01702
Rell1	chr5	12.8434	9.60635	3.237	0.01786
Ddx28	chr8	10.7038	7.46714	3.237	0.03752
Stxbp5l	chr16	11.3141	8.08538	3.229	0.02718
A730082K24Rik	chr7	12.7593	9.53087	3.228	0.00428
Dcp1a	chr14	16.6656	13.4411	3.224	9.1E-05
1700026J04Rik	chr13	11.6557	8.43323	3.222	0.01767
Sult4a1	chr15	9.86597	6.64484	3.221	0.004
Cdh1	chr8	14.2014	10.9817	3.220	0.00017
Slc44a2	chr9	13.9944	10.7758	3.219	0.00014
Laptm4b	chr15	14.8078	11.5919	3.216	0.00035
Nup35	chr2	13.3408	10.1251	3.216	0.0054
Ppargc1b	chr18	10.7474	7.53203	3.215	0.00802
BC018507	chr13	15.0663	11.8554	3.211	4.1E-05
Tspyl4	chr10	12.5615	9.36173	3.200	0.00026
Slc12a9	chr5	11.6488	8.44999	3.199	0.00027
Gm10391	chr14	9.95684	6.76086	3.196	0.01052
Mageh1	chrX	10.4369	7.24111	3.196	0.00899
Ubqln2	chrX	12.9205	9.72919	3.191	0.01141
Cdc42se2	chr11	15.264	12.0769	3.187	1.1E-05
Ezh2	chr6	14.9888	11.8032	3.186	0.00314
Tk1	chr11	11.1395	7.95491	3.185	0.02682
Gpr3	chr4	11.5856	8.40396	3.182	0.00875
Gm10394	chr14	9.94921	6.76881	3.180	0.0109
2700049A03Rik	chr12	14.6508	11.4734	3.177	8.1E-05
Bora	chr14	11.9131	8.73733	3.176	0.00437
B4galt6	chr18	13.3182	10.1459	3.172	0.00884
Aass	chr6	14.2044	11.0371	3.167	0.00034
Ivd	chr2	10.9656	7.80119	3.164	0.02957
Sufu	chr19	12.2501	9.08692	3.163	0.00121
Rdh11	chr12	13.8847	10.7244	3.160	0.00117
Creld2	chr15	13.8182	10.6611	3.157	1.7E-05
Gm13125	chr4	12.0687	8.91237	3.156	0.01087
Zfand2a	chr5	13.359	10.204	3.155	0.00347
Kbtbd7	chr14	15.1393	11.9891	3.150	0.00171
Epc1	chr18	13.6761	10.5266	3.149	0.00174
H13	chr2	12.9736	9.82545	3.148	0.01007
1700010I14Rik	chr17	10.0421	6.89529	3.147	0.0464
Zfp345	chr2	12.1484	9.0073	3.141	0.01921
Mxra8	chr4	8.17373	5.03803	3.136	0.01084
Atp6v0a2	chr5	9.6576	6.52239	3.135	0.00341
Ftsjd1	chr8	12.3275	9.19917	3.128	0.00533
Akr1b3	chr6	14.2655	11.1392	3.126	0.00025
Socs7	chr11	15.602	12.4778	3.124	8.1E-06
Rasl11b	chr5	10.8531	7.73239	3.121	0.00069
Lrrc17	chr5	13.566	10.4455	3.120	0.00222
Plekhb1	chr7	10.3683	7.25346	3.115	0.04531
Gm14085	chr2	9.14157	6.02851	3.113	0.00741
Phyhipl	chr10	10.4452	7.33398	3.111	0.02395
Gucy2e	chr11	12.8081	9.70962	3.099	0.00129
Baiap2l1	chr5	14.1476	11.0497	3.098	0.00051

Nhsl1	chr10	13.3325	10.2346	3.098	1.9E-05
Gsg2	chr11	12.802	9.70598	3.096	0.00293
G6pdx	chrX	15.0152	11.9225	3.093	0.00245
Fbxo16	chr14	8.50323	5.41226	3.091	0.00847
Lrrfip1	chr1	13.7781	10.6897	3.088	2.2E-06
Efcab6	chr15	12.0724	8.98401	3.088	0.00816
Mylip	chr13	8.92262	5.83607	3.087	0.02425
Pgs1	chr11	13.6418	10.5598	3.082	3E-05
Dnaja4	chr9	12.8481	9.767	3.081	0.00066
Hvcn1	chr5	11.7185	8.64073	3.078	0.00051
Oas1c	chr5	15.3751	12.2993	3.076	0.00187
Cmtm6	chr9	12.3504	9.27471	3.076	0.00319
Duxbl	chr14	9.8135	6.74078	3.073	0.00889
3230401D17Rik	chr2	15.3539	12.283	3.071	0.00092
Thap11	chr8	10.8411	7.77364	3.067	0.00872
Nfya	chr17	15.134	12.0678	3.066	1.5E-07
Cpm	chr10	9.45009	6.38457	3.066	0.01348
Dem1	chr4	11.8427	8.78003	3.063	0.00348
Itpr2	chr6	13.6112	10.5525	3.059	0.00148
Myo19	chr11	11.1647	8.10603	3.059	0.00599
Nrap	chr19	12.819	9.76067	3.058	0.00909
Nlrp14	chr7	17.8907	14.8335	3.057	0.00298
Ppme1	chr7	12.9454	9.89019	3.055	0.00013
Taf3	chr2	14.449	11.3966	3.052	0.00105
Mdm4	chr1	16.6037	13.556	3.048	3.3E-06
Dlg5	chr14	13.0411	9.99433	3.047	0.00872
Rhbdf1	chr11	11.4757	8.43953	3.036	0.00476
Nup214	chr2	15.5244	12.4932	3.031	1.6E-05
Klhl8	chr5	15.6529	12.6232	3.030	0.00086
Nlrp4g	chr9	13.2196	10.19	3.030	1.6E-06
Golm1	chr13	13.3251	10.2956	3.029	0.00172
Nap1l3	chrX	9.39785	6.37198	3.026	0.00391
Prr14	chr7	12.096	9.07216	3.024	0.00023
Usp28	chr9	14.8036	11.7855	3.018	1.4E-05
Pde3a	chr6	13.9061	10.8893	3.017	0.00024
Hist1h2bk	chr13	11.2606	8.24409	3.017	0.00028
Rwdd2b	chr16	11.3706	8.35656	3.014	0.00231
Sh3tc2	chr18	8.63405	5.62744	3.007	0.03705
Ticrr	chr7	14.5616	11.5569	3.005	1.6E-05
Gm4566	chr17	10.2062	7.20352	3.003	0.01135
Trnp1	chr4	9.07778	6.07585	3.002	0.00626
Sugt1	chr14	12.9328	9.93736	2.995	0.00293
5730469M10Rik	chr14	9.56581	6.57071	2.995	0.02294
Akap1	chr11	15.1666	12.1725	2.994	0.0002
Trim13	chr14	13.455	10.472	2.983	0.00365
Slc26a6	chr9	10.9942	8.01197	2.982	0.00296
Chaf1a	chr17	15.6181	12.6364	2.982	4.5E-05
Itgb3	chr11	13.5543	10.5809	2.973	0.04399
Gyk	chrX	12.9023	9.93253	2.970	0.00043
Anxa6	chr11	12.894	9.92566	2.968	0.00018
Gadd45gip1	chr8	13.2135	10.2474	2.966	0.00035
Antxr2	chr5	11.4747	8.51154	2.963	0.01301
Ddr2	chr1	10.8081	7.84779	2.960	0.00076
Kazn	chr4	10.4512	7.49507	2.956	0.00477
Slc16a10	chr10	13.1502	10.195	2.955	0.0041
Ptprr	chr10	11.4015	8.45071	2.951	0.00886
Fam131a	chr16	12.1151	9.16605	2.949	0.005

Zc3h3	chr15	12.741	9.79233	2.949	0.00462
Zp1	chr19	15.0386	12.0911	2.947	0.00102
Irf1	chr11	13.1881	10.2459	2.942	0.00179
Setd4	chr16	12.871	9.93421	2.937	0.00074
Etl4	chr2	12.4664	9.53103	2.935	0.00078
Shroom4	chrX	12.2315	9.29932	2.932	0.00285
Gnb4	chr3	13.2196	10.2936	2.926	0.00297
Mospd1	chrX	12.9775	10.0546	2.923	7.1E-06
Fbxl6	chr15	10.9357	8.0155	2.920	0.03339
Epn2	chr11	15.372	12.452	2.920	6.5E-06
Slco4a1	chr2	11.3531	8.43348	2.920	0.00526
Shank2	chr7	10.8892	7.96959	2.920	0.00461
Zcchc10	chr11	13.0097	10.0912	2.918	0.00014
Cited2	chr10	12.7307	9.81397	2.917	0.0029
Spire2	chr8	10.5101	7.59417	2.916	0.02531
Arl15	chr13	11.6132	8.70114	2.912	0.00091
Ano6	chr15	10.7148	7.80711	2.908	0.00129
Rai14	chr15	12.0044	9.09731	2.907	0.02103
Znrf2	chr6	14.1222	11.2163	2.906	0.00043
Med21	chr6	11.1111	8.20733	2.904	0.04915
Crnkl1	chr2	14.9471	12.0434	2.904	5.2E-07
Kcnn2	chr18	11.042	8.13924	2.903	0.01632
Dennd3	chr15	10.9537	8.05294	2.901	0.00535
Ankrd13a	chr5	13.958	11.0576	2.900	0.00042
Lrrc8b	chr5	12.2451	9.34628	2.899	0.0073
Rraga	chr4	12.4391	9.54569	2.893	0.00127
Sh3gl1	chr17	12.0813	9.18925	2.892	0.00035
Nfil3	chr13	12.0979	9.20742	2.890	0.00195
Brdt	chr5	13.6968	10.8064	2.890	0.00121
Smad3	chr9	14.3558	11.4654	2.890	0.00029
F13a1	chr13	8.99809	6.11096	2.887	0.04248
Bod1	chr11	14.5622	11.6753	2.887	4.9E-05
Csrnp2	chr15	13.8867	11	2.887	5.7E-05
Arntl	chr7	12.5168	9.63072	2.886	0.00027
Tmem232	chr17	11.7531	8.86789	2.885	0.00298
Abcc4	chr14	12.0812	9.198	2.883	0.00066
Dnajc3	chr14	14.5078	11.6335	2.874	9.2E-06
Fmnl3	chr15	15.3536	12.4872	2.866	0.00053
Dnahc2	chr11	8.99142	6.12556	2.866	0.00018
Dse	chr10	11.8753	9.01459	2.861	0.01319
Pofut2	chr10	12.4712	9.61157	2.860	0.00107
Aurka	chr2	13.7221	10.8641	2.858	0.00142
Lass5	chr15	12.5592	9.70391	2.855	0.00422
2810453I06Rik	chr5	13.8595	11.0047	2.855	0.00013
Pim1	chr17	9.10214	6.24782	2.854	0.00255
Mastl	chr2	13.7162	10.8633	2.853	0.00103
Ccnb3	chrX	11.5319	8.68021	2.852	0.01132
Gm904	chr13	8.47032	5.6214	2.849	0.00562
Htr7	chr19	9.0109	6.1688	2.842	0.00066
Tiparp	chr3	15.4763	12.6367	2.840	0.00126
Map3k6	chr4	13.3404	10.5046	2.836	0.00057
Dusp14	chr11	12.5223	9.6908	2.831	0.02462
Arrdc2	chr8	11.782	8.95077	2.831	0.00496
Tmem241	chr18	10.2216	7.39288	2.829	0.0248
Lrp11	chr10	10.7811	7.95254	2.829	0.00156
Zfp592	chr7	15.0476	12.2197	2.828	6.8E-06
Ephb6	chr6	11.9672	9.14224	2.825	0.00596

Nkd1	chr8	14.0475	11.2274	2.820	0.00092
Srd5a3	chr5	11.5007	8.68093	2.820	0.00718
Mxd1	chr6	12.8682	10.0486	2.820	4.7E-05
Txnrd1	chr10	13.8467	11.0279	2.819	0.01235
Glce	chr9	15.296	12.479	2.817	0.00022
Gja4	chr4	14.8122	12.0001	2.812	1.2E-05
Wwc1	chr11	10.8439	8.03194	2.812	0.00016
Mfsd9	chr1	10.9593	8.14778	2.811	0.00027
Rln3	chr8	9.50571	6.69487	2.811	0.00776
Fkbp5	chr17	14.6376	11.8302	2.807	0.00102
Cry1	chr10	13.9004	11.0954	2.805	0.00228
Amfr	chr8	14.7261	11.9219	2.804	0.00045
Bcl2l1	chr2	13.0357	10.2321	2.804	0.00114
Jhdm1d	chr6	14.0908	11.2877	2.803	2.3E-05
Snx22	chr9	11.0655	8.26304	2.802	0.00015
Chga	chr12	10.8248	8.02431	2.800	0.02172
Kcnh1	chr1	15.3582	12.5613	2.797	0.00015
Skap1	chr11	11.7679	8.97251	2.795	0.00807
Arl8a	chr1	10.6845	7.88943	2.795	0.02321
Eif2ak4	chr2	12.7544	9.96132	2.793	0.00295
Hspa12a	chr19	11.8784	9.08723	2.791	0.00071
Cnot7	chr8	14.9306	12.1396	2.791	1.2E-05
Asgr2	chr11	11.6383	8.84796	2.790	0.00861
Spag1	chr15	12.998	10.2103	2.788	0.00245
Atg5	chr10	14.1787	11.3951	2.784	0.00037
Rims2	chr15	9.19043	6.41062	2.780	0.00696
Smarcd2	chr11	13.5484	10.7686	2.780	8.4E-05
Fndc3a	chr14	14.6181	11.8419	2.776	1.9E-06
Snx9	chr17	13.8119	11.039	2.773	3.7E-05
Ccdc6	chr10	16.7024	13.93	2.772	0.0002
Klhl32	chr4	11.3294	8.55726	2.772	0.00839
Fndc3b	chr3	13.0337	10.2631	2.771	7.5E-05
Brox	chr1	12.8571	10.0866	2.770	0.01386
Bai3	chr1	9.75909	6.99111	2.768	0.03503
Prkab1	chr5	12.4653	9.69828	2.767	0.00017
Scyl2	chr10	13.3753	10.6095	2.766	0.00076
Foxj3	chr4	16.1149	13.3515	2.763	2.7E-06
Rbbp7	chrX	16.5487	13.7859	2.763	1.1E-05
Syngr3	chr17	9.59403	6.83155	2.762	0.04183
Slc25a32	chr15	11.2795	8.51892	2.761	0.00161
Pla2g15	chr8	12.5744	9.81396	2.760	0.00142
Rsl1d1	chr16	14.4754	11.7153	2.760	0.00026
Slc22a23	chr13	11.8535	9.09372	2.760	0.00245
Sdf2	chr11	12.3048	9.54645	2.758	0.01719
Agpat2	chr2	10.0642	7.30753	2.757	0.04766
Tor1a	chr2	13.4777	10.7215	2.756	8.1E-05
Ctnnal1	chr4	11.7706	9.01527	2.755	0.00523
Tsc22d1	chr14	14.813	12.0587	2.754	1.8E-05
Lig4	chr8	12.8838	10.1306	2.753	0.03239
Pfdn4	chr2	12.6333	9.88103	2.752	0.01148
Arfip1	chr3	14.1923	11.4407	2.752	0.00902
Kbtbd8	chr6	16.043	13.2927	2.750	6.1E-05
Lzts2	chr19	10.7239	7.97518	2.749	0.0138
Smurf2	chr11	14.0764	11.3327	2.744	8.8E-06
Actn1	chr12	12.5893	9.84589	2.743	0.00494
Lrp2	chr2	9.97727	7.23497	2.742	0.01719
Ptpn13	chr5	12.0564	9.31427	2.742	0.00519

Fgd6	chr10	14.204	11.4633	2.741	3.7E-05
Lrr1	chr12	12.4165	9.67746	2.739	0.00134
Taok3	chr5	12.6489	9.91027	2.739	0.00112
Kif26a	chr12	9.07502	6.33659	2.738	0.00298
Lbr	chr1	13.6725	10.9352	2.737	0.0022
Gm9766	chr10	10.7095	7.9754	2.734	0.00746
Gypc	chr18	8.3227	5.58982	2.733	0.001
Ckap2	chr8	13.2619	10.5334	2.729	0.0004
1700030K09Rik	chr8	11.0041	8.27581	2.728	0.00463
Pycrl	chr15	11.6802	8.95219	2.728	0.00663
Zbtb8b	chr4	11.2346	8.50727	2.727	0.00103
Ndfip2	chr14	12.5638	9.83811	2.726	0.00259
Gtf2ird1	chr5	11.6787	8.955	2.724	0.02245
Ube2u	chr4	9.02116	6.29961	2.722	0.00644
Paip2b	chr6	15.5427	12.8234	2.719	0.00027
Hmgxb4	chr8	12.8457	10.128	2.718	0.00794
Raf1	chr6	13.589	10.8717	2.717	1.4E-05
Slc15a2	chr16	10.443	7.72679	2.716	0.03245
Fam83g	chr11	14.1268	11.413	2.714	0.00404
Unc50	chr1	14.9967	12.285	2.712	0.00022
Slc9a1	chr4	13.0198	10.3134	2.706	0.02063
Unc5a	chr13	11.3815	8.67664	2.705	0.02357
Acbd3	chr1	15.1612	12.4577	2.703	3.5E-05
Tbc1d30	chr10	10.8475	8.14614	2.701	0.00116
Klhl18	chr9	13.2545	10.554	2.700	0.0006
Nus1	chr10	14.3938	11.6936	2.700	0.00157
2700050L05Rik	chr7	14.8009	12.1009	2.700	9.9E-06
1700018B24Rik	chr3	11.2022	8.50373	2.699	0.01154
H1f0	chr15	12.5693	9.87089	2.698	0.0008
Npas2	chr1	11.0059	8.30774	2.698	0.00683
Icmt	chr4	13.8532	11.1569	2.696	0.00149
Ncoa3	chr2	14.6353	11.9393	2.696	2.4E-05
Mcc	chr18	13.7972	11.1036	2.694	1E-05
Ska1	chr18	12.5638	9.87049	2.693	0.00125
Elovl5	chr9	13.8716	11.1785	2.693	0.00431
Trim27	chr13	14.251	11.5589	2.692	6.8E-05
Tmem102	chr11	9.01406	6.32518	2.689	0.0216
C77370	chrX	10.7216	8.03452	2.687	0.00573
Osbpl10	chr9	12.633	9.9471	2.686	0.00119
Abcg1	chr17	11.9023	9.21702	2.685	0.01138
Limd2	chr11	10.0946	7.41181	2.683	0.00028
Dnahc5	chr15	10.0362	7.35563	2.681	0.00023
Pdhx	chr2	13.5702	10.8917	2.678	0.00027
Fbxl7	chr15	10.422	7.74551	2.676	0.01804
Diap1	chr18	14.3129	11.642	2.671	3.9E-05
Pde10a	chr17	9.84889	7.18074	2.668	0.02872
Zfp708	chr13	11.5024	8.83452	2.668	0.01973
Dnahc8	chr17	12.7178	10.0501	2.668	0.00015
8430427H17Rik	chr2	12.3405	9.67359	2.667	0.04501
Rragc	chr4	15.2824	12.6204	2.662	3E-05
Zfp791	chr8	11.5261	8.86512	2.661	0.00449
Myt1	chr2	10.6711	8.01013	2.661	0.00427
Usp10	chr8	11.9872	9.32709	2.660	0.00102
Pard6b	chr2	12.9727	10.3152	2.658	0.00047
Wdr91	chr6	11.8802	9.22293	2.657	0.00375
Acox3	chr5	13.2588	10.6025	2.656	3.2E-05
Tacc1	chr8	12.1902	9.53413	2.656	0.0015

Gemin7	chr7	11.3267	8.67092	2.656	0.00646
Nedd4l	chr18	13.0351	10.3804	2.655	0.00046
Map4k5	chr12	14.3679	11.7146	2.653	0.00185
Echdc3	chr2	11.4703	8.81711	2.653	0.00021
Klf4	chr4	9.98794	7.33564	2.652	4.2E-05
Zdhhc24	chr19	12.3152	9.66331	2.652	5.9E-05
Madd	chr2	13.4941	10.8439	2.650	0.00034
Foxr1	chr9	10.3778	7.72807	2.650	0.00891
Ccdc92	chr5	12.9211	10.2743	2.647	0.00965
Ctif	chr18	11.2922	8.64678	2.645	0.00345
Rph3al	chr11	13.4751	10.8333	2.642	0.00014
Zdhhc8	chr16	12.7314	10.0905	2.641	0.00046
Ptges3l	chr11	9.53312	6.89764	2.635	0.01449
Pcnt	chr10	15.9935	13.3591	2.634	0.00021
Dedd	chr1	14.0199	11.3857	2.634	0.00098
Tom1l1	chr11	11.3433	8.71036	2.633	0.00557
Tmem132d	chr5	9.60625	6.97363	2.633	0.00434
Tgfb3	chr12	11.9747	9.34204	2.633	4.8E-07
Nup85	chr11	13.2306	10.6009	2.630	0.00275
Polr2c	chr8	14.1425	11.514	2.628	0.00088
Khdc1a	chr1	13.2995	10.6729	2.627	0.00846
Zfp334	chr2	12.4191	9.79447	2.625	0.00333
Adamtsl1	chr4	13.2882	10.6664	2.622	0.00022
Tcte2	chr17	10.2112	7.59377	2.617	0.01663
Derl3	chr10	11.4294	8.81233	2.617	0.00208
Fam13c	chr10	12.9317	10.3148	2.617	0.00179
Zfp560	chr9	11.4846	8.86788	2.617	0.00241
Ophn1	chrX	12.4895	9.87471	2.615	0.00025
Ctbs	chr3	9.66138	7.04705	2.614	7.3E-06
Sema5a	chr15	13.1829	10.5733	2.610	2E-05
Stk35	chr2	15.6737	13.0656	2.608	0.00015
Naip1	chr13	10.842	8.23647	2.606	0.00036
Cnot6l	chr5	14.85	12.2484	2.602	9.2E-05
Aldh1a2	chr9	11.6285	9.02767	2.601	0.00274
Helq	chr5	12.9049	10.3053	2.600	9.9E-06
Cdc6	chr11	11.2676	8.66898	2.599	0.0018
Zfp541	chr7	11.1832	8.58585	2.597	0.00177
Caskin2	chr11	11.1312	8.5345	2.597	0.00334
Pou4f1	chr14	12.9716	10.3761	2.596	0.00209
Lysmd4	chr7	12.1669	9.57279	2.594	0.00011
Scn10a	chr9	12.2672	9.67376	2.593	0.00961
Pms1	chr1	12.2807	9.68973	2.591	0.00073
Taf9b	chrX	13.0484	10.4632	2.585	0.00281
Mapre2	chr18	13.1602	10.5765	2.584	0.00017
Trp53bp1	chr2	13.9978	11.4163	2.581	0.00072
Fgf7	chr2	8.89995	6.32317	2.577	0.01971
Rab3il1	chr19	11.5815	9.00504	2.576	0.00295
Slc7a6os	chr8	13.1517	10.576	2.576	0.00384
Zim3	chr7	8.51102	5.9375	2.574	0.017
Dpysl3	chr18	10.9672	8.39427	2.573	0.00029
Ttyh3	chr5	12.8632	10.2922	2.571	5.3E-05
Dyrk1a	chr16	14.6556	12.0864	2.569	7.3E-05
Srcrb4d	chr5	9.99392	7.42722	2.567	0.00065
Ubiad1	chr4	12.5524	9.98725	2.565	0.0035
Rab3gap1	chr1	14.6468	12.0819	2.565	0.0003
Dcbld2	chr16	14.5448	11.98	2.565	1.5E-05
Phf19	chr2	9.21723	6.65362	2.564	0.00234

Cdc45	chr16	11.8863	9.32609	2.560	0.0039
Dusp7	chr9	14.3814	11.8223	2.559	4.6E-06
0610009B22Rik	chr11	11.2624	8.70346	2.559	0.00048
Sh3d19	chr3	12.6967	10.1428	2.554	0.00403
Mbl2	chr19	12.3675	9.8169	2.551	0.00486
Srek1ip1	chr13	11.2694	8.72072	2.549	0.00488
Vil1	chr1	13.4772	10.9288	2.548	0.00023
Riok1	chr13	14.8662	12.3184	2.548	1.1E-05
Prickle1	chr15	14.2975	11.7499	2.548	0.00131
Jag1	chr2	15.6427	13.0991	2.544	2.8E-05
Dnajc11	chr4	15.3672	12.825	2.542	2.5E-05
Nek2	chr1	13.4729	10.9308	2.542	0.00033
Rac3	chr11	10.8807	8.33878	2.542	9.4E-05
Zswim4	chr8	12.0526	9.51121	2.541	4.1E-05
Chek1	chr9	14.5061	11.9655	2.541	0.00177
Pak1	chr7	14.352	11.8123	2.540	0.00027
Pole4	chr6	14.0528	11.5165	2.536	0.00024
Prdm10	chr9	10.9612	8.42516	2.536	0.03276
Sh3rf2	chr18	11.0604	8.52622	2.534	0.005
Tbc1d15	chr10	15.9849	13.4509	2.534	0.0003
Palld	chr8	12.4144	9.88127	2.533	0.00892
Cnn3	chr3	12.8481	10.3152	2.533	0.00592
Pgbd5	chr8	12.8023	10.2706	2.532	0.0001
Cage1	chr13	12.1232	9.59276	2.530	0.00013
Ubqln1	chr13	14.7277	12.1989	2.529	0.0003
Csrp2bp	chr2	14.28	11.753	2.527	3.4E-05
Btbd10	chr7	13.8282	11.3068	2.521	0.00011
Myo18a	chr11	13.8658	11.3457	2.520	0.00332
Zbtb7b	chr3	11.7303	9.21291	2.517	0.00838
Gcnt4	chr13	14.4256	11.9114	2.514	6.2E-05
Bcorl1	chrX	11.8588	9.34709	2.512	0.00156
Alg10b	chr15	14.7675	12.256	2.512	0.00045
Kpna3	chr14	14.0178	11.5063	2.511	0.00051
Rrm2b	chr15	14.3515	11.8412	2.510	0.00185
Llgl2	chr11	10.9987	8.4937	2.505	0.0455
1810037I17Rik	chr3	11.1426	8.63984	2.503	0.00458
Mrps25	chr6	12.4911	9.9894	2.502	0.00056
Taf1a	chr1	13.793	11.2928	2.500	0.00229
Cftr	chr6	9.27237	6.77393	2.498	0.02458
Tspan5	chr3	12.3862	9.88965	2.497	0.0035
Isl2	chr9	10.1542	7.65829	2.496	7.9E-05
Mtf1	chr4	14.1704	11.6769	2.494	0.00014
Piwil1	chr5	11.518	9.02608	2.492	0.01268
6430548M08Rik	chr8	11.49	9.00108	2.489	0.00019
Irf8	chr8	10.1019	7.61321	2.489	0.0203
Bnc1	chr7	13.2485	10.7608	2.488	0.00109
Zfp820	chr17	9.22435	6.73674	2.488	0.03812
Tgfbrap1	chr1	12.575	10.0874	2.488	0.00529
Osbp2	chr11	12.0985	9.61104	2.487	0.00222
Apitd1	chr4	12.4511	9.96489	2.486	0.00521
Suz12	chr11	14.6967	12.211	2.486	8.9E-06
Osbpl9	chr4	14.2195	11.7339	2.486	0.00357
Cd99l2	chrX	10.4407	7.95548	2.485	0.01068
Brf2	chr8	12.3313	9.8469	2.484	0.00255
Pop1	chr15	12.6463	10.162	2.484	0.00555
Ranbp3	chr17	13.7889	11.3066	2.482	0.00083
AI846148	chr19	9.96412	7.48716	2.477	0.00354

Orc5	chr5	13.3857	10.9105	2.475	0.00893
Pwp2	chr10	12.119	9.64437	2.475	0.00029
Ampd3	chr7	12.2581	9.78767	2.470	0.00531
Ccdc117	chr11	14.8797	12.4103	2.469	0.00203
Fbxo18	chr2	14.0679	11.5999	2.468	0.00011
Bmi1	chr2	15.1387	12.675	2.464	0.00102
Ppfia1	chr7	15.0865	12.6244	2.462	7.7E-05
Fam160a1	chr3	15.1551	12.6941	2.461	4.6E-05
Nyx	chrX	9.13304	6.67274	2.460	0.019
Ccdc14	chr16	13.5956	11.1355	2.460	0.0004
Rcn2	chr9	12.164	9.70634	2.458	0.00527
Tacc3	chr5	15.974	13.5166	2.457	0.00031
Timm9	chr12	12.3589	9.90247	2.456	0.00045
Ece1	chr4	10.7799	8.32743	2.452	0.00088
Pacrgl	chr5	12.7241	10.2717	2.452	0.00569
Hist1h2ba	chr13	9.5697	7.11756	2.452	0.00359
Mtus1	chr8	17.2839	14.8318	2.452	6E-06
Tubb4b	chr2	16.0086	13.5572	2.451	0.00028
Fam35a	chr14	11.977	9.52619	2.451	4.7E-05
Litaf	chr16	11.0081	8.55844	2.450	0.00823
Fam187b	chr7	9.80342	7.35402	2.449	0.01297
Fam110c	chr12	13.3762	10.9276	2.449	0.00314
Zfp277	chr12	12.1733	9.72619	2.447	0.00214
Gng12	chr6	12.866	10.4219	2.444	0.00267
Chsy1	chr7	13.9513	11.5127	2.439	0.00139
Phf3	chr1	15.7058	13.2735	2.432	0.00012
Pam16	chr16	12.0957	9.66376	2.432	0.00057
Il17rd	chr14	13.5449	11.113	2.432	0.00161
Usp27x	chrX	12.3623	9.93348	2.429	0.0015
Hras1	chr7	11.5868	9.1604	2.426	0.01911
Ccdc47	chr11	14.6856	12.2592	2.426	2.5E-05
Prmt2	chr10	12.4688	10.0433	2.426	4.9E-06
Inpp5b	chr4	11.9912	9.566	2.425	0.01359
Dnajb1	chr8	13.4306	11.0055	2.425	2.4E-05
Srprb	chr9	12.3846	9.96082	2.424	0.00244
Gulo	chr14	9.94378	7.52283	2.421	0.02545
Trak1	chr9	13.865	11.4443	2.421	0.00018
Cdk17	chr10	12.1398	9.71956	2.420	0.0023
D1Bwg0212e	chr1	12.9355	10.5199	2.416	0.00326
Nefh	chr11	10.7491	8.33555	2.414	0.02408
Kdm6a	chrX	14.4609	12.0478	2.413	0.00389
Atp13a3	chr16	14.7457	12.3333	2.412	0.00403
Rab27a	chr9	12.7071	10.2948	2.412	0.00735
Srgap1	chr10	11.1189	8.70694	2.412	0.00828
Cul1	chr6	15.8281	13.4204	2.408	0.00019
Stxbp5	chr10	12.6936	10.2932	2.400	0.00207
Naa11	chr5	12.3234	9.92451	2.399	0.00251
Tmcc2	chr1	13.3648	10.9662	2.399	0.00178
Fam69b	chr2	10.7466	8.34808	2.399	0.00673
Tnfrsf21	chr17	9.59674	7.20222	2.395	0.00496
Hs2st1	chr3	12.6573	10.2653	2.392	0.00021
Otud3	chr4	11.0618	8.67218	2.390	0.03715
Hyls1	chr9	10.5228	8.13444	2.388	0.01681
Ttc37	chr13	13.9103	11.5244	2.386	0.00067
Bcl2l13	chr6	12.987	10.6012	2.386	0.00138
Atxn1l	chr8	14.376	11.9914	2.385	0.00114
Cdc42bpb	chr12	14.3845	12.0023	2.382	0.0025

Ldlrad3	chr2	11.1127	8.73131	2.381	0.03967
Tctn2	chr5	10.2547	7.87341	2.381	0.00391
Gm20234	chr11	9.11495	6.73404	2.381	0.0006
Aldh2	chr5	13.763	11.3855	2.378	3.6E-06
Gk5	chr9	10.7423	8.36588	2.376	0.00238
Acvr1b	chr15	12.0949	9.71992	2.375	0.00032
Gramd1c	chr16	11.1904	8.81555	2.375	0.00272
Nadsyn1	chr7	9.74038	7.36781	2.373	0.02819
Clcc1	chr3	13.3502	10.9777	2.373	0.00011
Eya1	chr1	11.115	8.74366	2.371	0.00023
Gtdc1	chr2	11.3349	8.96483	2.370	0.00302
Ccdc109a	chr10	12.4959	10.1265	2.369	0.00145
Dmrtb1	chr4	10.8422	8.47634	2.366	0.01636
Ube2k	chr5	14.3855	12.0225	2.363	0.00189
Hps6	chr19	11.4727	9.11007	2.363	0.01552
Rab7	chr6	13.3092	10.9479	2.361	0.00012
Pkd2l2	chr18	15.4159	13.0555	2.360	3.9E-05
Ust	chr10	9.54232	7.1822	2.360	0.04284
B4galt4	chr16	13.8252	11.4686	2.357	0.00842
Rufy1	chr11	14.584	12.2301	2.354	3.7E-05
Mphosph6	chr8	14.7229	12.3695	2.353	2E-05
Ank	chr15	9.93969	7.58678	2.353	0.02568
Cyb5	chr18	11.5432	9.19056	2.353	0.00325
P4ha1	chr10	13.1959	10.8434	2.353	9E-05
Lsr	chr7	11.7568	9.40631	2.350	0.00094
Henmt1	chr3	12.7049	10.3556	2.349	0.00419
Bms1	chr6	16.3339	13.9845	2.349	0.00012
A630007B06Rik	chr19	15.0966	12.7475	2.349	2.7E-05
Capn1	chr19	10.0982	7.74967	2.349	0.0139
Zfp710	chr7	10.9497	8.6039	2.346	0.00045
Zkscan17	chr11	11.9886	9.64398	2.345	0.0164
Zswim3	chr2	12.6986	10.3562	2.342	0.00728
Nsf	chr11	14.6291	12.2868	2.342	0.00026
Syt11	chr3	11.5473	9.20585	2.341	0.00347
Zfp449	chrX	11.6061	9.26874	2.337	0.00817
Hmg20a	chr9	12.7244	10.3878	2.337	0.00501
Zkscan14	chr5	12.5846	10.2485	2.336	0.00457
Zc3h6	chr2	13.7188	11.3833	2.335	2.7E-05
Ube2d2a	chr18	14.1222	11.7921	2.330	0.0015
Pecam1	chr11	11.8805	9.55165	2.329	2.5E-06
Itga6	chr2	14.6882	12.3596	2.329	0.00066
Tmtc3	chr10	12.3503	10.0222	2.328	0.00173
Zfp68	chr5	13.1706	10.8434	2.327	0.00057
Fyn	chr10	13.7397	11.4141	2.326	0.00485
Brsk2	chr7	11.5918	9.26632	2.325	0.01285
Npc1	chr18	14.692	12.3698	2.322	0.0002
Osbpl11	chr16	13.875	11.5555	2.319	0.00155
Sh3yl1	chr12	11.2506	8.93376	2.317	0.015
Glrx5	chr12	13.551	11.2344	2.317	0.00042
Usp49	chr17	12.3047	9.99122	2.314	0.00015
Htra2	chr6	11.0644	8.75191	2.312	0.00192
Tmem150a	chr6	10.3573	8.04489	2.312	0.03956
Adam17	chr12	14.9039	12.5963	2.308	2.2E-06
Mpdz	chr4	13.5598	11.2542	2.306	0.0056
Usp38	chr8	13.4776	11.1729	2.305	3.8E-05
Ccdc84	chr9	10.4321	8.12754	2.305	0.00056
Zfp385a	chr15	10.8828	8.57855	2.304	0.00015

Cdca5	chr19	13.612	11.3087	2.303	0.01479
H2afx	chr9	10.871	8.57286	2.298	0.0007
Arhgap20	chr9	9.44446	7.14846	2.296	0.02294
Samd4	chr14	9.00182	6.70738	2.294	0.0013
Mark2	chr19	15.6168	13.3231	2.294	0.00035
Ehd4	chr2	13.6451	11.353	2.292	0.00053
Tekt2	chr4	8.50728	6.21611	2.291	0.02547
Cltb	chr13	12.8682	10.5775	2.291	0.00672
Elk3	chr10	10.2863	7.99593	2.290	0.0189
Bet3l	chr10	10.8984	8.61358	2.285	0.00232
Brip1	chr11	13.2282	10.9442	2.284	0.00083
Srgn	chr10	11.1047	8.82253	2.282	0.00139
Ddi2	chr4	13.8714	11.5894	2.282	0.00147
Pdzrn4	chr15	10.3718	8.09022	2.282	5.8E-05
Abhd4	chr14	13.1072	10.8319	2.275	0.00302
Dnalc4	chr15	11.307	9.03238	2.275	0.0077
Hist1h1a	chr13	8.46922	6.19476	2.274	0.00992
Got1	chr19	9.5163	7.24715	2.269	0.00435
Rbms2	chr10	11.8376	9.56883	2.269	0.01468
Tert	chr13	10.8909	8.6242	2.267	0.01361
Lrriq3	chr3	9.91159	7.64525	2.266	0.00344
Esco1	chr18	13.3096	11.0438	2.266	0.00767
Ldb1	chr19	12.8468	10.5829	2.264	0.00021
Kdm4a	chr4	13.6943	11.4305	2.264	1.8E-05
Tril	chr6	9.83991	7.57646	2.263	0.00742
Top1	chr2	15.2751	13.0131	2.262	0.00634
Sart3	chr5	14.862	12.6011	2.261	0.0001
Nup98	chr7	15.4331	13.1765	2.257	0.00011
Etv1	chr12	11.1814	8.92619	2.255	0.02621
Mep1b	chr18	8.8339	6.57938	2.255	0.02897
Pigx	chr16	9.68851	7.43567	2.253	0.04497
Ctps	chr4	14.4086	12.1559	2.253	0.0036
Dennd2a	chr6	11.4393	9.1873	2.252	0.00414
Abhd13	chr8	14.3804	12.1291	2.251	0.00179
Bcl7b	chr5	11.2208	8.97148	2.249	0.00042
Fam116a	chr14	13.3289	11.0803	2.249	0.01561
Gm5465	chr14	11.8461	9.59823	2.248	0.01111
1700017B05Rik	chr9	11.3416	9.09369	2.248	0.00144
Lrp6	chr6	15.0717	12.8286	2.243	0.00028
Ccdc19	chr1	10.586	8.34584	2.240	0.01449
Tfe3	chrX	13.0997	10.8607	2.239	7.8E-05
Slc30a5	chr13	12.4818	10.2442	2.238	0.0081
Pex13	chr11	12.6842	10.4472	2.237	9.3E-05
Mapk1ip1	chr7	12.2532	10.0165	2.237	0.00258
Gtf2b	chr3	14.6151	12.3807	2.234	0.00026
Zmynd8	chr2	11.3244	9.09017	2.234	0.01839
Samd10	chr2	11.1028	8.86919	2.234	0.0072
Pkhd1	chr1	9.21993	6.98743	2.232	0.02822
Ccdc120	chrX	10.6456	8.4141	2.232	0.00891
Suv39h2	chr2	13.4264	11.1979	2.229	0.00706
Fbxo8	chr8	12.6221	10.3961	2.226	9.5E-05
Fads3	chr19	8.71632	6.49076	2.226	0.01195
Pithd1	chr4	10.3422	8.11677	2.225	0.03224
Atg16l1	chr1	12.935	10.7098	2.225	0.0006
Nfrkb	chr9	13.7928	11.5689	2.224	0.00187
Dctd	chr8	11.6674	9.44384	2.224	0.04181
Pde6d	chr1	12.0699	9.85042	2.220	0.00086

Ddr1	chr17	10.2826	8.06329	2.219	0.00985
Tmbim6	chr15	14.1591	11.9413	2.218	0.00015
Nt5dc1	chr10	9.99758	7.78374	2.214	0.02804
Prss44	chr9	9.19185	6.97979	2.212	0.04464
Ctnna1	chr18	13.6946	11.4844	2.210	1.6E-06
Wdr47	chr3	12.8756	10.6671	2.209	0.00362
Poln	chr5	14.3378	12.1303	2.207	6.4E-05
Tmem231	chr8	11.7276	9.52025	2.207	5.3E-05
Fam54a	chr10	12.4487	10.2445	2.204	0.01549
Reep2	chr18	12.8399	10.6365	2.203	0.00521
Zfp598	chr17	12.5898	10.3867	2.203	0.00024
Mfap3	chr11	12.1709	9.9689	2.202	0.00525
Mrpl48	chr7	13.0731	10.8715	2.202	2.7E-05
Rbm18	chr2	13.9276	11.7272	2.200	0.00118
Foxo1	chr3	14.5015	12.3019	2.200	0.00514
Mid1	chrX	13.0423	10.8432	2.199	0.00023
Ctnnbl1	chr2	14.409	12.2113	2.198	0.00122
Scoc	chr8	11.6721	9.47534	2.197	0.00032
Gspt2	chrX	12.7648	10.5687	2.196	0.00036
Phospho2	chr2	13.9809	11.7866	2.194	0.00343
Camsap1	chr2	14.4635	12.2698	2.194	0.00046
Polr2d	chr18	12.6855	10.4936	2.192	0.02515
Sycp2	chr2	12.0261	9.83525	2.191	0.0008
E2f4	chr8	11.9164	9.72563	2.191	0.00149
Rbpms	chr8	11.7073	9.51706	2.190	0.00956
Rnf38	chr4	16.2546	14.0658	2.189	9.3E-05
Fbxo28	chr1	13.7552	11.5666	2.189	0.00101
Zfp956	chr6	11.5279	9.34068	2.187	0.0196
Syne2	chr12	16.0273	13.8415	2.186	4.9E-05
Lrp5	chr19	14.2968	12.112	2.185	0.00037
Cntn2	chr1	11.2151	9.03103	2.184	0.01059
Psmf1	chr2	12.7052	10.5212	2.184	0.00652
Dppa5a	chr9	12.1756	9.99212	2.184	0.00125
B4galt5	chr2	13.2222	11.039	2.183	0.0004
BC057022	chr5	11.5965	9.4163	2.180	0.00727
Ss18	chr18	12.5258	10.3468	2.179	0.0003
Cacna1i	chr15	10.8233	8.64669	2.177	0.04743
Zfp513	chr5	12.1525	9.97929	2.173	0.00571
Znfx1	chr2	14.0666	11.8948	2.172	0.00222
Nsmce2	chr15	12.951	10.7806	2.170	0.00054
D9Ertd402e	chr9	11.858	9.68769	2.170	0.02364
Rcl1	chr19	11.4338	9.26419	2.170	0.00087
Ncoa7	chr10	9.16881	7.00106	2.168	0.00936
Rbl1	chr2	11.3483	9.18057	2.168	0.00291
Aim1	chr10	13.123	10.9574	2.166	0.00209
Ctdspl	chr9	10.8096	8.64818	2.161	1.3E-05
Lipt1	chr1	11.5402	9.37888	2.161	0.01063
Dip2b	chr15	13.5622	11.4015	2.161	0.01909
Birc5	chr11	14.4203	12.2606	2.160	0.00527
Coq10b	chr1	11.6285	9.47059	2.158	0.01276
Lrrc1	chr9	10.3143	8.1574	2.157	0.00871
Slc7a1	chr5	11.8472	9.6906	2.157	1E-05
Gli2	chr1	10.5139	8.35741	2.157	0.00013
Tmem201	chr4	14.1593	12.0044	2.155	0.00042
Fam70b	chr8	12.8711	10.7167	2.154	0.00279
Arih1	chr9	14.6872	12.5357	2.152	1.4E-05
4930402H24Rik	chr2	12.9637	10.8124	2.151	9.5E-05

Tyw1	chr5	14.3737	12.2226	2.151	0.00066
Ppard	chr17	11.2463	9.09516	2.151	0.03067
Rybp	chr6	12.9128	10.7632	2.150	0.01354
Ccdc45	chr11	12.4705	10.3213	2.149	0.0038
Fcgbp	chr7	11.1565	9.00913	2.147	0.00088
Stx5a	chr19	14.3405	12.1959	2.145	0.00138
Ap3m2	chr8	11.8909	9.74724	2.144	0.00058
Snap29	chr16	12.412	10.2697	2.142	0.00122
Ccdc141	chr2	9.54841	7.40958	2.139	0.00064
Ablim2	chr5	11.9053	9.76687	2.138	0.00094
AW146154	chr7	10.85	8.71158	2.138	0.01715
Arid4a	chr12	12.4818	10.3441	2.138	0.0084
Senp1	chr15	13.4324	11.2956	2.137	0.00236
Mrpl12	chr11	11.2192	9.08245	2.137	0.01328
Mbtd1	chr11	14.4777	12.341	2.137	2.4E-05
Zfp964	chr8	8.68525	6.54914	2.136	0.01757
Zbtb2	chr10	10.6322	8.49635	2.136	0.0128
Abhd12	chr2	11.7136	9.57805	2.136	7.8E-05
Kif22	chr7	14.0375	11.902	2.136	0.00025
Gdap2	chr3	12.4867	10.3513	2.135	0.00591
Atrx	chrX	16.1668	14.0329	2.134	0.0001
4933411K20Rik	chr8	13.5952	11.4684	2.127	0.00054
Arap2	chr5	13.6445	11.5195	2.125	0.00016
Tusc3	chr8	12.1032	9.98009	2.123	0.00938
C87436	chr6	11.1096	8.98788	2.122	0.02496
Rasa2	chr9	14.132	12.0105	2.121	0.00153
Nfkbiz	chr16	11.1699	9.04955	2.120	0.04129
Fam172a	chr13	11.1992	9.07946	2.120	0.00208
Efnb2	chr8	12.6277	10.5091	2.119	7E-05
Pkn2	chr3	13.755	11.6384	2.117	0.00606
Nfxl1	chr5	14.7186	12.6025	2.116	1.9E-06
Sft2d1	chr17	11.6454	9.52966	2.116	0.00825
Mllt10	chr2	14.5218	12.4076	2.114	0.00127
Atp2a2	chr5	15.6886	13.5766	2.112	0.00057
Rp9	chr9	10.9315	8.82021	2.111	0.02874
Ppm1b	chr17	14.8804	12.7713	2.109	0.00092
Gxylt1	chr15	14.2224	12.1144	2.108	0.00542
Cacnb2	chr2	9.98548	7.87815	2.107	0.00022
Sgsm3	chr15	12.5952	10.4883	2.107	0.02606
Rps6ka5	chr12	12.0528	9.94638	2.106	0.00037
Eif2b4	chr5	13.0989	10.9964	2.103	0.0048
Paip1	chr13	13.5503	11.4485	2.102	0.00453
Acsl3	chr1	13.2865	11.1893	2.097	0.003
Foxk2	chr11	13.4099	11.3153	2.095	0.0016
1500015A07Rik	chr18	8.52926	6.43563	2.094	0.03799
Cybasc3	chr19	10.7761	8.68268	2.093	0.00019
Patz1	chr11	12.1127	10.0194	2.093	3.2E-05
Gm19299	chr9	10.8558	8.76394	2.092	0.01424
Dennd4c	chr4	13.7047	11.6138	2.091	0.0008
Lrriq1	chr10	9.12124	7.03046	2.091	0.00268
Zmiz1	chr14	11.5263	9.43647	2.090	0.00154
Ccdc58	chr16	12.0275	9.94042	2.087	0.00284
Pias1	chr9	14.1338	12.0502	2.084	3.7E-05
Med28	chr5	13.1854	11.1025	2.083	0.0001
Txndc11	chr16	11.9394	9.85712	2.082	0.02221
Tceb1	chr1	13.1471	11.067	2.080	0.00117
Slain2	chr5	13.6625	11.5842	2.078	0.0002

Cdc27	chr11	14.4559	12.3777	2.078	0.00086
Pdss2	chr10	10.038	7.96217	2.076	0.02186
Enox1	chr14	12.2876	10.2146	2.073	0.0005
Oas1h	chr5	13.8034	11.7318	2.072	0.00244
Nup50	chr15	14.0481	11.9772	2.071	0.00047
Amotl1	chr9	10.5654	8.49508	2.070	0.04452
Slc35b3	chr13	10.1004	8.03031	2.070	0.02184
Mus81	chr19	12.2249	10.1553	2.070	0.00017
Pard6g	chr18	10.7086	8.63919	2.069	0.03989
Tmem60	chr5	12.9039	10.8348	2.069	0.00211
Fbxo33	chr12	12.2859	10.2176	2.068	0.00037
BC031353	chr9	13.302	11.2341	2.068	0.00047
Suco	chr1	14.4603	12.3926	2.068	0.00117
Ibtk	chr9	14.9843	12.9175	2.067	0.00038
1110003E01Rik	chr5	13.3928	11.327	2.066	0.00456
Pde8a	chr7	11.6514	9.58564	2.066	0.00917
Nusap1	chr2	13.5405	11.4781	2.062	0.00012
Gpr125	chr5	13.4235	11.362	2.062	0.00034
Inpp5a	chr7	11.6278	9.56858	2.059	0.02582
Eepd1	chr9	11.2329	9.17541	2.058	0.00763
Tbc1d8	chr1	13.3763	11.3198	2.057	0.00197
Arhgap12	chr18	14.4305	12.3781	2.052	5.7E-05
Hmgb2	chr8	15.108	13.0565	2.051	0.00117
Snrnp27	chr6	11.0079	8.96167	2.046	0.02043
Foxm1	chr6	13.5742	11.529	2.045	0.01179
Txn1	chr4	9.3191	7.27396	2.045	0.01363
Map2k1	chr9	13.8175	11.7728	2.045	0.00036
Cox10	chr11	13.248	11.2072	2.041	0.00794
Usp3	chr9	12.427	10.3868	2.040	0.00221
Pxt1	chr17	10.4512	8.41106	2.040	0.00023
Gm4349	chr3	8.91758	6.87874	2.039	0.01807
Ino80e	chr7	11.2628	9.22606	2.037	0.00675
Fbxo30	chr10	13.8746	11.8403	2.034	0.00101
Zfp143	chr7	11.2033	9.16907	2.034	0.00773
Cept1	chr3	12.9503	10.9178	2.032	0.01032
AW554918	chr18	12.3723	10.3404	2.032	0.01508
Ooep	chr9	14.5036	12.4718	2.032	0.00091
Cdk2ap2	chr19	10.8557	8.82558	2.030	0.02669
Ing1	chr8	13.2615	11.2315	2.030	2.5E-06
Fam168a	chr7	12.2077	10.1804	2.027	0.00234
Sdf2l1	chr16	9.66472	7.63817	2.027	0.0019
Slc31a2	chr4	11.3753	9.34942	2.026	0.00817
Zfp383	chr7	11.6975	9.67255	2.025	0.03599
Rab4a	chr8	11.3644	9.34025	2.024	0.00172
Syt7	chr19	11.4227	9.39956	2.023	0.0096
Zfp61	chr7	12.8184	10.7965	2.022	0.00038
Fryl	chr5	14.0038	11.982	2.022	0.00215
N4bp1	chr8	13.7	11.6787	2.021	0.00608
2700062C07Rik	chr18	11.6924	9.67208	2.020	0.0009
Rpa3	chr6	10.638	8.6179	2.020	0.00115
Klhl13	chrX	12.5747	10.5548	2.020	6.3E-05
Uvrag	chr7	13.7858	11.7659	2.020	2.8E-05
Cotl1	chr8	9.6944	7.67692	2.017	0.00159
Utp20	chr10	13.8052	11.7893	2.016	0.00111
Stk40	chr4	11.775	9.76469	2.010	0.00265
Pag1	chr3	10.5181	8.50812	2.010	0.04715
Zbtb37	chr1	12.0536	10.0442	2.009	3.3E-05

Igf2bp2	chr16	15.5825	13.5748	2.008	7.4E-06
Anln	chr9	13.2376	11.2301	2.008	3.1E-05
Tube1	chr10	11.273	9.26565	2.007	0.04151
Exosc3	chr4	11.2936	9.28622	2.007	0.00757
Tnks1bp1	chr2	14.4673	12.4611	2.006	0.00067
Myo5b	chr18	15.6465	13.6418	2.005	0.00277
Elmod2	chr8	14.7615	12.7572	2.004	2.6E-05
Kif2c	chr4	13.6244	11.6205	2.004	4.6E-05
Snupn	chr9	12.4007	10.3986	2.002	0.00167
Cdc20	chr4	11.5514	9.55117	2.000	0.00477
Dbndd1	chr8	12.4921	10.492	2.000	0.00026
Abcf3	chr16	15.1208	13.122	1.999	6.9E-05
Rdh10	chr1	13.1291	11.1304	1.999	0.00029
Zc3h8	chr2	10.0711	8.07316	1.998	0.0223
Lrrfip2	chr9	13.9032	11.9057	1.998	6.1E-05
Sh3rf3	chr10	11.3692	9.37206	1.997	0.00537
Trim11	chr11	11.8076	9.81231	1.995	0.0182
Fam83d	chr2	9.78762	7.79372	1.994	0.02025
Gm20605	chr5	13.121	11.1278	1.993	0.00053
Dhx57	chr17	12.3191	10.3269	1.992	0.00153
Maea	chr5	14.4323	12.4408	1.991	0.00046
Gpn2	chr4	11.213	9.22207	1.991	0.00723
Nhej1	chr1	9.52007	7.52937	1.991	0.04451
Rnf141	chr7	11.3226	9.3319	1.991	0.00982
Fxyd6	chr9	9.48031	7.49118	1.989	0.00222
Pold3	chr7	14.4758	12.4884	1.987	0.00154
Hnrnpul2	chr19	16.0231	14.0373	1.986	1.5E-05
Armcx5	chrX	11.424	9.44034	1.984	0.0139
Cryl1	chr14	11.593	9.61025	1.983	0.00323
Rnf220	chr4	14.483	12.5004	1.983	5.7E-06
Phf12	chr11	13.6345	11.6539	1.981	0.00041
Naa40	chr19	13.2014	11.2253	1.976	0.00065
Ak2	chr4	13.9529	11.9784	1.974	0.00803
Anks4b	chr7	11.6946	9.72023	1.974	0.00055
Scfd2	chr5	10.7913	8.81753	1.974	7.2E-06
Tm9sf2	chr14	15.2584	13.2852	1.973	2.3E-05
Esyt2	chr12	13.8979	11.9266	1.971	0.00482
Lsm14a	chr7	15.1783	13.211	1.967	0.00459
Zp2	chr7	15.6609	13.6937	1.967	0.0052
Lin37	chr7	11.3147	9.3476	1.967	0.02015
Rttn	chr18	13.2921	11.3266	1.965	0.00324
Blcap	chr2	12.9629	10.9978	1.965	0.00936
Ccnd2	chr6	11.6317	9.66696	1.965	0.005
Ambra1	chr2	13.6053	11.6417	1.964	0.00822
Mzt2	chr16	9.50638	7.54596	1.960	0.02644
Strn	chr17	15.1533	13.1929	1.960	0.00181
Timm8a2	chr14	9.59782	7.63794	1.960	0.00015
Dna2	chr10	11.1597	9.20005	1.960	0.02047
Iffo1	chr6	10.6853	8.72673	1.959	0.01954
Zfp280b	chr10	15.0574	13.0993	1.958	3.2E-05
E2f1	chr2	13.5168	11.5588	1.958	0.00014
Dcp2	chr18	13.5458	11.5883	1.958	0.00526
Nxt1	chr2	11.3027	9.34897	1.954	0.00239
Cnot8	chr11	13.2774	11.3272	1.950	0.00158
Cdc25a	chr9	12.9133	10.9636	1.950	0.00787
Btrc	chr19	14.3818	12.4347	1.947	0.00036
2610002J02Rik	chr4	8.47097	6.52387	1.947	0.00051

Tbc1d22b	chr17	13.5884	11.6421	1.946	0.00149
Hax1	chr3	13.315	11.3697	1.945	0.0002
Kremen1	chr11	12.541	10.5959	1.945	0.00173
Dtx2	chr5	14.5383	12.5948	1.944	3.2E-05
St6galnac3	chr3	9.00549	7.06275	1.943	0.04352
Nrcam	chr12	10.25	8.30795	1.942	0.01849
Ldhb	chr6	16.992	15.0512	1.941	0.0003
Cbx2	chr11	12.5255	10.5856	1.940	0.00287
Gatad2b	chr3	14.2466	12.3086	1.938	0.00267
Tspan33	chr6	12.3197	10.3824	1.937	0.00442
Rab6a	chr7	13.305	11.3686	1.936	0.00021
A030009H04Rik	chr11	9.41348	7.47731	1.936	0.01141
Rpap2	chr5	13.0309	11.0955	1.935	0.00132
BC067068	chr10	8.91758	6.98233	1.935	0.0409
Wdr82	chr9	13.7106	11.7756	1.935	0.0002
Hmgcs1	chr13	15.5432	13.6085	1.935	2.7E-05
Smpd1	chr7	11.9738	10.0394	1.934	0.00998
Daam1	chr12	14.66	12.7263	1.934	2E-06
Abcd4	chr12	9.50607	7.5727	1.933	0.03241
Klhdc2	chr12	14.5965	12.664	1.933	0.00249
Npc2	chr12	12.1435	10.2126	1.931	0.0008
Stat6	chr10	11.4266	9.49783	1.929	0.01982
Haus4	chr14	11.5435	9.61481	1.929	0.01854
Abhd6	chr14	10.685	8.75765	1.927	0.01184
Zfp160	chr17	11.5275	9.60067	1.927	1.1E-05
Apex2	chrX	10.0985	8.17299	1.926	0.00687
Sap30bp	chr11	12.7277	10.8025	1.925	0.00942
Mbtps2	chrX	12.4506	10.5277	1.923	0.00188
Hus1	chr11	12.6968	10.7754	1.921	0.00268
N4bp2	chr5	13.7573	11.8361	1.921	0.00088
Emb	chr13	9.49582	7.57605	1.920	0.01293
Troap	chr15	11.2498	9.3324	1.917	0.00106
Ints9	chr14	12.9487	11.0321	1.917	0.00021
Tmcc3	chr10	14.5842	12.6677	1.916	0.00036
Fam126b	chr1	13.189	11.273	1.916	0.00585
Mcm10	chr2	12.7704	10.8561	1.914	1.6E-06
Mrps27	chr13	11.7368	9.82247	1.914	0.00304
Tfpt	chr7	12.8783	10.9649	1.913	0.00158
Brd1	chr15	14.0704	12.1577	1.913	0.00046
Slc3a2	chr19	13.5256	11.6131	1.913	0.00647
Aph1c	chr9	10.5927	8.68118	1.912	0.01805
Fbf1	chr11	11.1999	9.28839	1.912	2.8E-05
Plekha3	chr2	12.4558	10.5465	1.909	0.00712
Ippk	chr13	14.0678	12.1604	1.907	0.0013
Gfod1	chr13	11.6673	9.76015	1.907	0.00973
Emc8	chr8	13.1746	11.2693	1.905	0.00445
Mfap1b	chr2	13.4493	11.5455	1.904	0.00493
Lpcat1	chr13	13.6994	11.7962	1.903	0.00096
Ptprn2	chr12	9.17235	7.27	1.902	0.00018
Srm	chr4	11.5114	9.60968	1.902	0.0059
D7Ertd443e	chr7	12.7816	10.88	1.902	0.00045
Shroom3	chr5	12.0974	10.196	1.901	0.00777
Adprm	chr11	10.3795	8.47961	1.900	0.00587
Zc3h7b	chr15	12.537	10.6377	1.899	0.00025
Fip1l1	chr5	15.5077	13.6086	1.899	0.00065
Mrpl18	chr17	11.7606	9.86159	1.899	0.00119
Atp2b3	chrX	10.5768	8.67813	1.899	0.0378

Atp10a	chr7	9.54436	7.64646	1.898	0.00054
Tesc	chr5	12.0471	10.1504	1.897	0.01547
Eif2c3	chr4	12.8737	10.9775	1.896	0.00059
Rnf181	chr6	12.3596	10.4637	1.896	0.01307
Heatr6	chr11	11.6761	9.78114	1.895	0.04793
Esrrg	chr1	11.6842	9.79004	1.894	0.01392
Snx16	chr3	11.6805	9.78779	1.893	0.01023
Lrrc14	chr15	11.096	9.20332	1.893	0.00377
D10Wsu102e	chr10	16.6571	14.7646	1.893	2.3E-05
Eps15l1	chr8	13.6942	11.8019	1.892	6.6E-05
Ell	chr8	13.1047	11.2123	1.892	2.6E-05
Abcb8	chr5	11.3995	9.50802	1.891	0.00907
Med30	chr15	10.4735	8.583	1.890	0.00467
Zc3h12c	chr9	11.9714	10.0838	1.888	0.00341
Senp8	chr9	12.8434	10.9587	1.885	0.00468
Pou5f1	chr17	13.1857	11.3026	1.883	0.00011
Rnaseh1	chr12	12.6258	10.7459	1.880	0.00771
Strn4	chr7	11.9018	10.0222	1.880	0.00212
Zkscan6	chr11	11.8628	9.98446	1.878	0.00304
Errfi1	chr4	12.246	10.3702	1.876	0.00185
Slc39a6	chr18	14.7657	12.8905	1.875	0.00584
Rhno1	chr6	10.7073	8.83251	1.875	0.02375
Abat	chr16	9.346	7.47148	1.875	0.01332
Cenpa	chr5	11.2212	9.34699	1.874	0.00853
Fam134c	chr11	12.8617	10.989	1.873	0.00094
Srebf1	chr11	11.6027	9.73322	1.870	0.01328
Slc7a11	chr3	15.0953	13.2261	1.869	9.2E-05
Lrrc61	chr6	10.4314	8.5625	1.869	0.02892
Hmgn1	chr16	14.4524	12.5857	1.867	0.00098
Ttc23	chr7	11.5661	9.70016	1.866	0.00069
Cnppd1	chr1	14.3617	12.4957	1.866	0.0059
Ripk1	chr13	11.4384	9.57254	1.866	0.00706
Nudc	chr4	15.9981	14.1328	1.865	0.00087
Diexf	chr1	12.5091	10.6437	1.865	6.5E-05
Zfp60	chr7	12.7534	10.8934	1.860	0.00029
Eed	chr7	13.4242	11.5643	1.860	0.00792
Gpr137b	chr13	11.1035	9.24384	1.860	0.00434
Mras	chr9	8.35283	6.49331	1.860	0.01585
Pak4	chr7	12.8655	11.0065	1.859	0.00306
Nr6a1	chr2	13.5049	11.6458	1.859	0.00049
Nrg4	chr9	11.6026	9.74374	1.859	0.00062
Phf23	chr11	12.5849	10.7264	1.859	0.00257
Ndnl2	chr7	12.8406	10.9823	1.858	0.00331
Dnajc8	chr4	14.0073	12.149	1.858	0.00018
Fat1	chr8	15.1661	13.3085	1.858	0.00396
Fam53c	chr18	14.921	13.0641	1.857	0.00474
Pigf	chr17	12.7407	10.8852	1.856	0.00188
Usp19	chr9	15.7879	13.9356	1.852	0.0005
Tada3	chr6	11.5213	9.67249	1.849	0.02347
Cycs	chr6	11.8276	9.97916	1.848	0.00253
Bcl7c	chr7	12.4918	10.6434	1.848	0.02071
Cdc25b	chr2	12.5895	10.7417	1.848	0.00028
Fbxo42	chr4	12.654	10.8069	1.847	0.00886
Polr3a	chr14	13.9423	12.0956	1.847	0.00517
Ltbp2	chr12	9.65182	7.80533	1.846	0.02865
Pdcd2l	chr7	13.2122	11.3673	1.845	0.00447
Mat2b	chr11	15.5755	13.7334	1.842	7.9E-05

B3galnt2	chr13	12.3398	10.4983	1.841	0.0007
Pptc7	chr5	12.8264	10.9865	1.840	0.00471
Abl2	chr1	15.8476	14.0083	1.839	7.2E-06
Tmem38b	chr4	11.3409	9.50199	1.839	0.00502
Tle6	chr10	15.8871	14.0488	1.838	0.00026
D1Ertd622e	chr1	12.0611	10.2234	1.838	0.00124
Mxd4	chr5	9.97574	8.13862	1.837	0.01278
Ptprm	chr17	10.4835	8.64694	1.837	0.00121
Wwc2	chr8	15.0686	13.2327	1.836	0.00071
Dnmt3a	chr12	14.1618	12.326	1.836	0.00046
Sirt4	chr5	9.24964	7.41496	1.835	0.03244
Lrrc8d	chr5	12.4428	10.6084	1.834	0.00057
Xpo4	chr14	11.2984	9.46421	1.834	0.00285
3110040N11Rik	chr7	9.66057	7.82648	1.834	0.00872
Timm17a	chr1	12.1586	10.3251	1.833	0.007
Thap1	chr8	12.0999	10.2678	1.832	0.00135
Tusc3	chr8	10.4494	8.61737	1.832	0.00086
Lin52	chr12	11.2284	9.39795	1.830	0.01294
Sema6d	chr2	12.4599	10.6319	1.828	0.00145
Zfp821	chr8	12.0392	10.2115	1.828	0.01247
Pycr2	chr1	10.0987	8.27147	1.827	0.00131
Tmem159	chr7	8.43616	6.6092	1.827	0.01718
Map3k12	chr15	10.6431	8.81664	1.827	0.00325
Tnik	chr3	10.4382	8.61254	1.826	0.00142
Sun1	chr5	12.602	10.7768	1.825	4.2E-05
Nsmce1	chr7	11.8722	10.0471	1.825	0.02487
Ccno	chr13	14.9453	13.1209	1.824	0.00059
D19Ertd737e	chr19	11.5919	9.76803	1.824	0.00235
Ada	chr2	10.8014	8.97974	1.822	0.04367
Edc4	chr8	14.3286	12.5075	1.821	0.00241
Wtap	chr17	13.675	11.8544	1.821	3.5E-05
Nhedc1	chr3	10.936	9.11768	1.818	0.00138
Luzp1	chr4	13.4225	11.6051	1.817	6.6E-05
Pde4dip	chr3	12.7359	10.9189	1.817	6.2E-05
Stau1	chr2	14.7665	12.9498	1.817	0.00046
Abcb9	chr5	11.9051	10.0895	1.816	0.00182
Rap2b	chr3	10.5467	8.73109	1.816	0.03506
AW146020	chr6	10.7571	8.94253	1.815	0.00216
Elp2	chr18	14.915	13.1005	1.815	9.8E-05
Sms	chrX	13.9004	12.0865	1.814	0.00047
1300018I17Rik	chr8	11.6383	9.82581	1.812	0.00885
Ppp1r12a	chr10	14.5168	12.7046	1.812	5.4E-05
Gon4l	chr3	13.9129	12.1008	1.812	0.00027
Mff	chr1	14.0242	12.2126	1.812	7.9E-05
Ddx19a	chr8	14.7724	12.9612	1.811	0.0004
Nphp3	chr9	8.65962	6.85119	1.808	0.02519
Ext1	chr15	12.994	11.1872	1.807	0.00128
Elp6	chr9	8.98545	7.1817	1.804	0.01448
Ppp1r14b	chr19	9.13315	7.32995	1.803	0.04674
Tug1	chr11	11.9265	10.1246	1.802	0.00065
Tirap	chr9	9.76802	7.96647	1.802	0.02282
Inadl	chr4	14.505	12.7042	1.801	0.00025
Glmn	chr5	12.5757	10.7776	1.798	0.01031
Yipf4	chr17	12.337	10.5393	1.798	0.02561
Zfp276	chr8	10.2809	8.48536	1.796	0.01569
Ccrn4l	chr3	13.2049	11.4098	1.795	0.00175
Osbpl8	chr10	14.8733	13.0791	1.794	1.9E-05

Cactin	chr10	13.1634	11.3692	1.794	0.00054
Phf1	chr17	13.87	12.0771	1.793	0.0032
41704	chr15	15.584	13.7941	1.790	0.00178
Col6a3	chr1	11.6868	9.89943	1.787	0.00068
Dhx8	chr11	13.1623	11.3776	1.785	1.1E-05
E130008D07Rik	chr17	8.41217	6.62902	1.783	0.01588
Celsr1	chr15	12.3986	10.6159	1.783	0.00175
Gle1	chr2	13.8652	12.0841	1.781	0.00244
Sult2a4	chr7	8.37728	6.5999	1.777	0.00482
Taz	chrX	12.0657	10.2893	1.776	0.00046
Slc25a13	chr6	10.6167	8.84134	1.775	0.00588
Mapkbp1	chr2	12.5632	10.7883	1.775	0.0127
Aven	chr2	12.339	10.565	1.774	0.02869
Armc10	chr5	12.9712	11.1972	1.774	0.00219
Ppa1	chr10	13.119	11.3474	1.772	0.00067
Tmem66	chr8	13.5261	11.7561	1.770	0.00158
Zdhhc7	chr8	10.5636	8.795	1.769	0.00691
Spty2d1	chr7	12.9731	11.2045	1.769	0.0002
Ctnnd1	chr2	13.2697	11.5026	1.767	0.00204
Ahsa2	chr11	10.5181	8.75148	1.767	0.03399
Degs1	chr1	14.1035	12.3375	1.766	0.00022
Ube2h	chr6	14.2932	12.5272	1.766	8.6E-06
Arhgap18	chr10	9.96665	8.20131	1.765	0.00028
Spg20	chr3	13.125	11.3608	1.764	0.00266
Ccdc101	chr7	12.2701	10.5066	1.763	0.00029
Snrnp40	chr4	12.6316	10.8681	1.763	0.00061
Galk2	chr2	10.5052	8.742	1.763	0.00241
Mif4gd	chr11	9.40428	7.6412	1.763	0.00046
Ipo8	chr6	15.7207	13.9578	1.763	0.00023
Fam188a	chr2	12.1999	10.4387	1.761	0.00379
Dis3l	chr9	13.2174	11.4566	1.761	0.00028
Tmco6	chr18	9.84665	8.08608	1.761	0.00276
Atf4	chr15	13.0899	11.3301	1.760	0.00055
Yipf5	chr18	12.5584	10.7989	1.760	0.00066
Efna4	chr3	9.44086	7.684	1.757	0.01193
Fam179b	chr12	13.5841	11.8275	1.757	0.00071
Ccdc99	chr11	13.5224	11.7683	1.754	6.4E-05
Spred2	chr11	10.2634	8.51314	1.750	0.00019
1110012L19Rik	chrX	10.4242	8.67417	1.750	0.0019
Cml2	chr6	10.8927	9.14312	1.750	0.00318
41705	chr2	14.0172	12.2679	1.749	0.00279
Gm16515	chr11	13.8263	12.0789	1.747	0.00175
Zfp341	chr2	11.7077	9.9612	1.747	0.00054
Tom1l2	chr11	13.1682	11.4221	1.746	0.00232
Map2k4	chr11	13.6249	11.8791	1.746	0.00012
Ppp2r2b	chr18	10.2145	8.47075	1.744	0.00057
Apc	chr18	14.1355	12.393	1.743	3.6E-06
Gas6	chr8	9.67447	7.93218	1.742	0.00306
Zfp574	chr7	11.7868	10.045	1.742	0.0122
Tjp2	chr19	12.3805	10.6403	1.740	0.00039
Clhc1	chr4	12.4792	10.7398	1.739	0.0011
E4f1	chr17	10.4038	8.66456	1.739	0.02785
Zfp781	chr10	10.9006	9.16184	1.739	0.00174
Atl2	chr17	14.4051	12.6694	1.736	0.00041
4930432K21Rik	chr8	12.3002	10.5645	1.736	0.00134
Brca1	chr11	13.6398	11.9061	1.734	0.00074
Smu1	chr4	14.1586	12.4257	1.733	0.00403

Os9	chr10	14.3978	12.6661	1.732	0.00382
Camsap2	chr1	14.3392	12.6076	1.732	1E-05
Rapgefl1	chr11	12.7245	10.9939	1.731	0.003
Nedd1	chr10	12.6072	10.8778	1.729	0.00402
BC030867	chr11	12.0123	10.2834	1.729	0.00044
Zfp952	chr17	11.0233	9.29522	1.728	0.00437
Dand5	chr8	10.4553	8.72938	1.726	0.00557
Bmp2k	chr5	13.6867	11.9612	1.726	8.5E-07
Lrrc16a	chr13	14.4042	12.6795	1.725	0.00021
Zfp212	chr6	13.7496	12.0251	1.724	0.01459
Cdk7	chr13	12.1963	10.4754	1.721	0.00639
Rnf34	chr5	14.545	12.8246	1.720	0.00309
Rnf14	chr18	13.3707	11.6515	1.719	0.00066
Dtl	chr1	12.9269	11.2089	1.718	0.00107
Lsp1	chr7	10.784	9.06715	1.717	0.02698
Txndc15	chr13	9.41071	7.6972	1.714	0.00572
Cyfip2	chr11	12.0294	10.3192	1.710	0.0018
Cs	chr10	14.1936	12.4836	1.710	0.00042
Uqcc	chr2	12.2095	10.4997	1.710	0.00019
Nmnat2	chr1	9.15736	7.44896	1.708	0.00471
Furin	chr7	14.5686	12.861	1.708	3.4E-05
Msh4	chr3	10.827	9.11977	1.707	0.04421
Ski	chr4	15.258	13.5509	1.707	0.00255
Jak2	chr19	15.0531	13.3463	1.707	0.00022
Smc3	chr19	14.5257	12.8196	1.706	0.00058
Dynlt1b	chr17	10.198	8.49231	1.706	0.0025
Haus8	chr8	11.7562	10.0508	1.705	1.4E-05
Taf4a	chr2	13.0203	11.3151	1.705	0.00073
Pdia5	chr16	10.1851	8.47982	1.705	0.02436
Tnpo1	chr13	15.4632	13.7599	1.703	0.0002
Ergic2	chr6	12.788	11.0849	1.703	0.00268
Nuf2	chr1	13.5763	11.8732	1.703	2.3E-05
Zfp830	chr11	12.1744	10.4716	1.703	0.00444
Arhgap26	chr18	12.3303	10.6294	1.701	0.00408
Atp6v0a2	chr5	11.3543	9.65447	1.700	0.02312
8430429K09Rik	chr11	9.411	7.71135	1.700	0.00122
B3gntl1	chr11	10.8643	9.16474	1.700	0.00236
Nfatc2	chr2	9.30106	7.60299	1.698	0.0245
Thop1	chr10	12.0147	10.3178	1.697	0.01396
Slc35f2	chr9	11.2176	9.52109	1.696	0.0003
Mier2	chr10	13.8805	12.1841	1.696	0.00929
Magi3	chr3	12.2075	10.5133	1.694	0.0012
Olfm1	chr2	10.9463	9.2567	1.690	0.00073
Dynlt1a	chr17	8.25817	6.56868	1.689	0.00172
Ralgps2	chr1	13.1626	11.4736	1.689	3.7E-05
Qtrt1	chr9	8.62112	6.93298	1.688	0.03702
Trpa1	chr1	10.4574	8.77197	1.685	0.00275
Arhgap21	chr2	13.9877	12.3024	1.685	0.00014
Brix1	chr15	13.9263	12.2417	1.685	0.00463
Slc25a19	chr11	11.8136	10.1292	1.684	0.0007
Zfp532	chr18	12.4111	10.727	1.684	0.00103
Galnt9	chr5	9.55644	7.87317	1.683	0.01569
AI118078	chr9	8.95113	7.26855	1.683	0.01889
Fchsd2	chr7	13.2778	11.5978	1.680	0.0002
Cxx1a	chrX	9.63771	7.95795	1.680	0.02033
Cdt1	chr8	12.5336	10.8543	1.679	0.00082
Pan2	chr10	13.2918	11.6129	1.679	4.9E-05

Zfp97	chr17	11.1832	9.50467	1.679	0.01175
Papolg	chr11	12.5748	10.8981	1.677	0.00079
Myh2	chr11	10.9001	9.22527	1.675	0.01556
Arhgap11a	chr2	14.3297	12.6551	1.675	0.00037
Adck2	chr6	9.94282	8.26923	1.674	0.00155
Arhgef1	chr7	11.453	9.77941	1.674	0.01136
Ncoa4	chr14	13.8774	12.204	1.673	0.00211
Wdr5	chr2	14.217	12.5445	1.672	6.4E-05
Gstcd	chr3	9.73918	8.06735	1.672	0.00322
Fam125b	chr2	12.7102	11.0383	1.672	0.00229
9330102E08Rik	chr6	11.1603	9.48918	1.671	0.037
Gpr137b-ps	chr13	11.5587	9.88792	1.671	0.01004
BC016423	chr13	13.8122	12.1418	1.670	2.3E-05
Ywhag	chr5	14.8525	13.1823	1.670	3.8E-06
Ireb2	chr9	14.9908	13.3217	1.669	0.00057
Mrpl22	chr11	8.93164	7.2637	1.668	0.01973
Lass6	chr2	13.866	12.1981	1.668	0.00136
Rras2	chr7	13.081	11.4137	1.667	0.00298
C330018D20Rik	chr18	10.8521	9.185	1.667	0.00266
Fam60a	chr6	12.6534	10.9864	1.667	0.02162
Mtmr2	chr9	12.5989	10.9321	1.667	0.00046
Dnajb2	chr1	11.8533	10.1875	1.666	0.00014
Akirin2	chr4	14.8771	13.2131	1.664	0.00024
Plxna2	chr1	12.8227	11.1589	1.664	0.0062
Lrig1	chr6	12.9024	11.2385	1.664	0.00038
Polr1d	chr5	13.5155	11.8534	1.662	0.01114
Robo1	chr16	11.4203	9.75898	1.661	0.00029
Sipa1l1	chr12	14.0649	12.4039	1.661	0.00016
Tsga10ip	chr19	9.58083	7.91992	1.661	0.04197
Dnaja1	chr4	15.6452	13.9843	1.661	7.9E-05
Gins3	chr8	10.4316	8.77135	1.660	0.01277
Tesk2	chr4	10.5712	8.91139	1.660	0.0399
Gm4961	chr5	11.4426	9.78299	1.660	0.00124
Msrb3	chr10	12.571	10.9119	1.659	0.03056
Rpusd4	chr9	10.9067	9.24882	1.658	0.00011
Hmgcll1	chr9	11.7109	10.0548	1.656	0.00051
Egln1	chr8	13.8474	12.1942	1.653	0.00149
Ubp1	chr9	14.2502	12.5979	1.652	0.00043
Ska3	chr14	12.2507	10.599	1.652	0.01084
Mthfd1	chr12	13.3912	11.7402	1.651	0.00115
Plagl1	chr10	10.3363	8.68633	1.650	0.01701
Smarcal1	chr1	11.9433	10.2935	1.650	0.00367
Fnbp1l	chr3	14.8641	13.2147	1.649	0.00051
Ccnyl1	chr1	12.7171	11.0687	1.648	0.0003
Rnf219	chr14	12.0711	10.4236	1.648	0.02336
Ercc6l	chrX	12.7961	11.1491	1.647	0.00228
Plk4	chr3	13.6202	11.9736	1.647	0.00075
Il17rb	chr14	11.0219	9.37768	1.644	0.03059
Uck2	chr1	11.9329	10.2901	1.643	0.00365
Ptpn2	chr18	13.4402	11.7975	1.643	0.00075
Zcchc8	chr5	14.6434	13.0014	1.642	0.00335
Tbc1d1	chr5	14.08	12.4389	1.641	0.00048
Pcyt1a	chr16	12.9229	11.283	1.640	0.01624
Zfhx3	chr8	14.3044	12.6648	1.640	0.00024
Mfap1a	chr2	13.9392	12.2998	1.639	0.00526
Ankrd17	chr5	16.4146	14.7754	1.639	4.6E-05
Atp2c1	chr9	14.9524	13.314	1.638	0.00098

Stk10	chr11	11.8141	10.1759	1.638	0.01194
Fam118a	chr15	12.1852	10.5471	1.638	0.00292
Hist2h2aa2	chr3	11.5178	9.8804	1.637	0.01394
Cnot3	chr7	14.9395	13.3027	1.637	4.5E-05
Sptlc1	chr13	12.4059	10.7692	1.637	1.1E-05
Surf4	chr2	14.7949	13.1583	1.637	4.5E-05
Rit1	chr3	10.6865	9.0504	1.636	0.03433
Usp39	chr6	11.6598	10.0242	1.636	0.01001
Sh3bp2	chr5	12.2991	10.6678	1.631	0.00064
5730494M16Rik	chr18	13.4061	11.7756	1.630	0.0032
Net1	chr13	14.117	12.4872	1.630	7.3E-05
Slc2a1	chr4	11.1035	9.47377	1.630	0.00112
AI481877	chr4	10.9435	9.31876	1.625	0.00195
Spag5	chr11	12.1227	10.4983	1.624	0.00016
Timeless	chr10	13.3082	11.6842	1.624	0.00961
Pdcd6	chr13	10.7369	9.11366	1.623	0.01844
Nol10	chr12	13.1032	11.4801	1.623	0.00203
Arid1a	chr4	15.291	13.6684	1.623	0.00351
Trp53inp2	chr2	13.5806	11.958	1.623	0.00017
Lrrc42	chr4	12.4585	10.8362	1.622	0.01676
Timm8b	chr9	10.1287	8.50764	1.621	0.01886
Pfkl	chr10	10.6188	8.99843	1.620	0.02472
Padi6	chr4	17.0582	15.4383	1.620	0.00682
Ttc39c	chr18	11.0823	9.46256	1.620	0.00491
Daglb	chr5	12.7533	11.1369	1.616	0.0019
Arhgap28	chr17	13.7216	12.1054	1.616	0.00017
Rfx5	chr3	12.4452	10.829	1.616	0.00025
Zbtb45	chr7	9.38319	7.76722	1.616	0.02496
Stk30	chr12	10.1831	8.56889	1.614	0.04526
Arpc3	chr5	12.5095	10.8965	1.613	0.00275
Trit1	chr4	11.3194	9.70749	1.612	0.01164
Kif18a	chr2	11.3434	9.73185	1.612	0.01029
Ddx25	chr9	10.6814	9.07007	1.611	0.04527
Slc25a38	chr9	10.466	8.85508	1.611	0.01111
Atad1	chr19	14.41	12.7992	1.611	6.4E-06
Cobl	chr11	10.5466	8.93648	1.610	0.02852
Pdcd7	chr9	14.4882	12.8785	1.610	4.5E-06
Zfp3	chr11	9.13316	7.52355	1.610	0.01757
Zfp58	chr13	11.81	10.2026	1.607	0.00073
Mavs	chr2	10.4508	8.84363	1.607	0.0385
Adpgk	chr9	11.8963	10.2895	1.607	0.00117
Flcn	chr11	12.2176	10.611	1.607	0.00076
Taf8	chr17	11.7299	10.1238	1.606	0.00549
Tcea1	chr1	14.0652	12.4599	1.605	0.00154
Epb4.1	chr4	13.8617	12.2567	1.605	0.00035
Cd2ap	chr17	13.5544	11.9497	1.605	0.00031
Casp8ap2	chr4	13.7075	12.1049	1.603	0.00012
Fam40b	chr6	10.55	8.94961	1.600	0.00763
Ruvbl2	chr7	13.6725	12.0725	1.600	0.01154
Lrrn4	chr2	9.81046	8.21151	1.599	0.00117
Mast4	chr13	13.492	11.8954	1.597	0.00548
Limk2	chr11	13.1255	11.5299	1.596	0.00468
Nrf1	chr6	12.6984	11.1033	1.595	0.00058
Ccdc130	chr8	11.4394	9.84555	1.594	0.01378
Ccdc90a	chr13	11.6007	10.0076	1.593	0.01583
Hipk2	chr6	12.6349	11.042	1.593	0.0004
Cep164	chr9	12.5708	10.9784	1.592	0.00049

Wdr17	chr8	9.46847	7.87618	1.592	0.00259
Atg2a	chr19	12.3017	10.7096	1.592	0.0027
Nexn	chr3	14.3881	12.796	1.592	0.00572
Zfp420	chr7	10.6904	9.09901	1.591	0.03668
Ankrd28	chr14	12.308	10.7179	1.590	0.00121
Ythdf3	chr3	13.7416	12.1517	1.590	0.01525
Pop7	chr5	9.93276	8.34361	1.589	0.00228
Herc2	chr7	15.3687	13.78	1.589	0.00054
Ints3	chr3	13.5437	11.9569	1.587	0.00046
Pik3r1	chr13	14.2734	12.6873	1.586	0.00412
Mphosph9	chr5	11.4984	9.91264	1.586	0.04594
Tmcc1	chr6	12.8916	11.3075	1.584	0.02284
Uhmk1	chr1	11.2953	9.71293	1.582	0.00409
Trim24	chr6	14.1133	12.5326	1.581	0.00021
Cdca8	chr4	12.4395	10.8599	1.580	0.00557
Smug1	chr15	12.5058	10.9264	1.579	0.00019
Golph3	chr15	14.3053	12.7259	1.579	0.0096
Hif3a	chr7	10.7465	9.16722	1.579	0.00082
Esco2	chr14	13.108	11.5317	1.576	0.00206
Dsn1	chr2	13.6468	12.0712	1.576	3.1E-05
Uso1	chr5	13.6636	12.0895	1.574	0.00224
Rbm7	chr9	13.1382	11.5653	1.573	0.00094
Ablim3	chr18	10.3455	8.77542	1.570	0.00039
Gm16039	chr6	12.8431	11.2747	1.568	0.00292
Atpbd4	chr2	12.6058	11.038	1.568	0.00176
Hnmt	chr2	8.75751	7.19021	1.567	0.01526
3110082I17Rik	chr5	11.99	10.4239	1.566	0.02175
Specc1l	chr10	14.7611	13.1967	1.564	0.00347
8430410K20Rik	chr9	10.6215	9.05725	1.564	1.7E-05
Slc25a16	chr10	11.3083	9.7451	1.563	0.0195
Pramef12	chr4	15.4494	13.8884	1.561	0.00537
2810025M15Rik	chr1	9.69758	8.14148	1.556	0.0229
41703	chr19	14.2995	12.7435	1.556	8.1E-05
Wls	chr3	8.62332	7.06799	1.555	0.04165
Zfp235	chr7	10.3654	8.81084	1.555	0.00537
Herpud2	chr9	13.7844	12.2332	1.551	0.003
Tsc22d4	chr5	12.4254	10.8742	1.551	0.00081
Aph1a	chr3	11.2973	9.74776	1.549	0.02251
Hiatl1	chr13	12.4898	10.9406	1.549	0.00991
Mknk1	chr4	12.7497	11.2006	1.549	0.02951
Sin3a	chr9	15.748	14.1997	1.548	3.2E-05
Alas1	chr9	11.1374	9.58911	1.548	0.00681
Cep55	chr19	12.592	11.044	1.548	0.0145
9430038I01Rik	chr7	10.1323	8.58492	1.547	0.00052
Fbxo11	chr17	13.7882	12.2409	1.547	0.01671
Dag1	chr9	12.5837	11.0366	1.547	0.00643
Gba2	chr4	10.5677	9.02071	1.547	0.04685
Emg1	chr6	10.5844	9.03941	1.545	0.0287
Zfp960	chr17	10.4241	8.87938	1.545	0.03398
Adamts17	chr7	10.033	8.48856	1.544	0.02062
Dap3	chr3	13.4435	11.8991	1.544	0.00482
Taf6l	chr19	11.7218	10.1795	1.542	0.03561
BC017647	chr11	13.2089	11.6668	1.542	0.00011
Fam184a	chr10	11.7633	10.2216	1.542	0.0159
Setd8	chr5	12.816	11.2745	1.541	9.4E-05
Zfp951	chr5	11.3973	9.85694	1.540	0.02165
Hyal2	chr9	10.5239	8.98387	1.540	0.00279

Kcmf1	chr6	14.7129	13.1738	1.539	0.00037
Scap	chr9	14.6354	13.0976	1.538	5.7E-05
Ndel1	chr11	13.2579	11.7201	1.538	0.00331
Gmpr	chr13	12.1727	10.6352	1.537	0.00055
Cdc37l1	chr19	12.6388	11.1017	1.537	0.00338
Noc4l	chr5	12.9896	11.453	1.537	0.0006
Mtpap	chr18	12.2041	10.669	1.535	0.03298
Cdca3	chr6	10.4151	8.88024	1.535	0.0119
Klhl2	chr8	11.8723	10.338	1.534	0.00051
Fam192a	chr8	13.8417	12.3081	1.534	0.00387
Lix1l	chr3	8.29625	6.76274	1.534	0.04252
Sec13	chr6	12.254	10.7215	1.532	0.00043
Siae	chr9	10.5899	9.05753	1.532	0.01067
Fut8	chr12	12.744	11.212	1.532	0.00327
Nkiras1	chr14	12.173	10.6414	1.532	0.00617
Ube2s	chr7	13.5785	12.047	1.532	0.0002
Mtmr7	chr8	12.6857	11.1547	1.531	0.00052
Ryk	chr9	11.9649	10.4343	1.531	0.02024
Kif20a	chr18	13.2568	11.7263	1.531	0.00986
Gas2l3	chr10	12.6766	11.1466	1.530	0.0045
Dstyk	chr1	13.5207	11.991	1.530	0.00066
Trim59	chr3	11.8624	10.3331	1.529	0.01328
Fdft1	chr14	15.487	13.9578	1.529	0.00012
Mfsd6	chr1	10.7201	9.19109	1.529	0.04538
Zscan20	chr4	11.3841	9.85568	1.528	0.01455
Scn8a	chr15	10.1111	8.583	1.528	0.03573
Zmynd19	chr2	13.7913	12.2633	1.528	0.00053
Itgb1bp1	chr12	11.0546	9.52685	1.528	0.00774
Arl6ip1	chr7	15.2068	13.6798	1.527	0.00144
Leprot	chr4	12.6914	11.1648	1.527	0.01295
Zfp709	chr8	10.358	8.83301	1.525	0.04231
Gria4	chr9	9.67394	8.14994	1.524	0.00906
Zfp568	chr7	11.9103	10.3864	1.524	0.00637
Slc20a1	chr2	12.8884	11.3646	1.524	0.00851
Tex19.1	chr11	11.4313	9.90849	1.523	0.0343
Mterfd2	chr1	12.2879	10.7665	1.521	0.00104
Dmwd	chr7	11.9319	10.4119	1.520	0.01832
Asf1b	chr8	11.8715	10.3515	1.520	0.0006
Mutyh	chr4	8.54011	7.02022	1.520	0.02319
Dcaf12	chr4	14.0147	12.4952	1.519	0.00017
1810013D10Rik	chr5	9.81645	8.29711	1.519	0.00139
Pigyl	chr9	10.7408	9.22323	1.518	0.00226
Gnptg	chr17	10.2322	8.71509	1.517	0.01259
Romo1	chr2	8.78176	7.26557	1.516	0.02939
Il17ra	chr6	11.6072	10.0925	1.515	0.00273
Bbs4	chr9	13.3407	11.8262	1.514	0.01367
Limd1	chr9	11.8827	10.3694	1.513	0.00176
Clic4	chr4	14.0603	12.5475	1.513	0.01771
Upf1	chr8	14.1911	12.6786	1.513	0.00694
Ncapd3	chr9	12.0875	10.5752	1.512	0.02279
Heatr5b	chr17	13.204	11.6925	1.511	0.00024
Siah1a	chr8	11.9307	10.4229	1.508	0.00141
2310033P09Rik	chr11	11.5583	10.0507	1.508	0.00154
Ube2g1	chr11	14.4065	12.9004	1.506	0.00124
Imp4	chr1	12.3674	10.8633	1.504	0.00317
Zfp955b	chr17	12.3943	10.8909	1.503	0.00016
Hist2h3c1	chr3	11.6424	10.1405	1.502	0.0219

Ppfibp2	chr7	13.9962	12.4956	1.501	0.00187
Zfp653	chr9	11.4481	9.94757	1.501	0.00418
Hsf1	chr15	11.4856	9.98542	1.500	0.0427
Ptk2b	chr14	8.66713	7.16702	1.500	0.02931
C2cd3	chr7	12.8584	11.3589	1.500	0.00729
Tprn	chr2	10.5107	9.01174	1.499	0.01983
Tmem161b	chr13	11.0233	9.52513	1.498	0.00266
Snrpd1	chr18	12.7334	11.2357	1.498	0.01113
Syce2	chr8	13.4343	11.9369	1.497	0.00057
Phldb3	chr7	9.63902	8.14183	1.497	0.03424
Brwd3	chrX	13.3676	11.8705	1.497	2.9E-05
Steap2	chr5	12.7094	11.2123	1.497	0.00448
Cenpq	chr17	12.4309	10.9344	1.497	0.0281
Arhgef11	chr3	12.6293	11.1334	1.496	0.00027
Grip1	chr10	10.167	8.67277	1.494	0.02549
Tmem209	chr6	13.2092	11.7152	1.494	0.00885
Racgap1	chr15	12.9758	11.4833	1.493	0.00418
Kif5c	chr2	12.7762	11.2853	1.491	0.00065
Tbp	chr17	12.0832	10.5962	1.487	0.03466
Psmd8	chr7	12.6801	11.1937	1.486	0.00193
Mcm4	chr16	13.7934	12.308	1.485	0.00074
Orc4	chr2	12.0663	10.5811	1.485	0.01445
D10Jhu81e	chr10	11.09	9.60491	1.485	0.01269
Gm3055	chr10	10.8312	9.34614	1.485	0.0099
Fancm	chr12	12.4105	10.9262	1.484	0.02267
Qars	chr9	14.824	13.3397	1.484	0.00057
Papss1	chr3	12.6767	11.1928	1.484	0.00496
Syf2	chr4	13.6212	12.1379	1.483	0.00206
Brap	chr5	13.2653	11.7823	1.483	0.00088
Trafd1	chr5	13.5539	12.0713	1.483	0.0005
Epha4	chr1	12.6873	11.2051	1.482	0.01253
Slc35a1	chr4	11.3244	9.84239	1.482	0.02264
Auh	chr13	12.8085	11.3268	1.482	0.0011
Usp1	chr4	13.8323	12.3518	1.481	0.00012
Olfr976	chr9	13.0281	11.5485	1.480	0.00018
Hn1l	chr17	14.491	13.012	1.479	0.00059
Prr5	chr15	11.831	10.3535	1.478	9.5E-05
Neo1	chr9	13.4381	11.9609	1.477	0.00324
Glcci1	chr6	12.6033	11.1266	1.477	0.00122
Rbm22	chr18	12.1354	10.6598	1.476	0.02084
Atp1a1	chr3	14.4267	12.9519	1.475	0.00206
Wdr53	chr16	12.6198	11.1457	1.474	0.00309
C78339	chr13	12.4987	11.0272	1.471	0.00392
Manf	chr9	12.4717	11.0007	1.471	0.00137
Cisd1	chr10	11.6931	10.2234	1.470	0.00192
Scaf4	chr16	12.5878	11.1198	1.468	0.00099
Zfp46	chr4	12.393	10.9252	1.468	0.001
Surf1	chr2	11.4792	10.0114	1.468	0.03422
Ift46	chr9	12.4081	10.9405	1.468	0.00283
Ralgds	chr2	11.4481	9.98123	1.467	0.00805
Ubac2	chr14	11.9732	10.5067	1.467	0.00044
Spata7	chr12	10.4591	8.99327	1.466	0.00275
Erh	chr12	10.9231	9.45955	1.464	0.00516
Ralbp1	chr17	15.2577	13.7945	1.463	1.8E-05
Prpf3	chr3	11.7906	10.329	1.462	0.00097
Psen2	chr1	11.6328	10.173	1.460	1.4E-05
Mapkap1	chr2	12.9544	11.4947	1.460	0.00099

Tmem132c	chr5	9.57049	8.1121	1.458	0.01258
Ubxn2a	chr12	11.7696	10.3115	1.458	0.01447
Chd8	chr14	14.5399	13.0818	1.458	0.00066
Banp	chr8	12.0761	10.6179	1.458	0.00016
D430042O09Rik	chr7	11.3278	9.87313	1.455	0.02097
Tmem39a	chr16	12.1261	10.6718	1.454	0.00841
Srf	chr17	11.3953	9.94307	1.452	0.04035
Hspb11	chr4	8.30385	6.85178	1.452	0.00867
Rnf19b	chr4	12.4753	11.0243	1.451	5.6E-05
Thap7	chr16	11.1671	9.71664	1.450	0.00103
Taf12	chr4	11.777	10.3279	1.449	0.00616
Tm2d3	chr7	12.1036	10.657	1.447	0.00646
Mrs2	chr13	9.28007	7.83349	1.447	0.04324
Rad52	chr6	10.5008	9.05527	1.446	0.04509
Immp1l	chr2	12.0334	10.588	1.445	0.00243
Eif4enif1	chr11	15.9909	14.5494	1.442	0.00012
Usf1	chr1	10.6639	9.22242	1.441	0.04713
E330009J07Rik	chr6	13.7359	12.2966	1.439	0.00389
Atf6	chr1	12.8545	11.4167	1.438	0.00082
Exo1	chr1	13.0673	11.6302	1.437	0.00312
9330182L06Rik	chr5	11.5151	10.0818	1.433	0.00048
Rnf113a2	chr12	11.376	9.94343	1.433	0.00286
Tubb5	chr17	13.0659	11.6337	1.432	0.00588
Ush2a	chr1	10.5173	9.08551	1.432	0.01169
Zhx3	chr2	12.259	10.8272	1.432	0.0251
Ddhd1	chr14	13.859	12.4284	1.431	0.00011
Dpf3	chr12	12.005	10.5743	1.431	0.03118
Brwd1	chr16	15.6239	14.1938	1.430	2.7E-05
Cit	chr5	14.5642	13.1374	1.427	0.00028
Ttk	chr9	13.4754	12.0493	1.426	0.00397
Rnf168	chr16	13.6314	12.2077	1.424	9.8E-05
Zscan21	chr5	11.7257	10.3022	1.423	0.0279
Caml	chr13	11.0231	9.59975	1.423	0.00238
Strada	chr11	11.965	10.5442	1.421	0.00014
B9d1	chr11	10.1998	8.77917	1.421	0.031
Abhd2	chr7	10.3954	8.97513	1.420	0.00027
Bspry	chr4	10.153	8.7331	1.420	0.0136
Txnip	chr3	15.3127	13.8943	1.418	0.00027
Psmd9	chr5	12.2861	10.868	1.418	0.0007
Rfx2	chr17	11.2337	9.81618	1.417	0.0122
Arhgap29	chr3	12.0887	10.673	1.416	0.0212
Eif5b	chr1	14.7107	13.295	1.416	0.0124
Dock3	chr9	12.9373	11.5226	1.415	0.01029
Gpcpd1	chr2	12.2247	10.8101	1.415	0.00502
Polr2g	chr19	13.1003	11.6872	1.413	0.01739
Wdr92	chr11	10.9988	9.58574	1.413	0.02658
Rbpms2	chr9	14.9048	13.4921	1.413	0.00524
Med20	chr17	12.4167	11.0045	1.412	0.00034
Mitd1	chr1	9.4867	8.07458	1.412	0.02282
Nsd1	chr13	15.3653	13.9533	1.412	0.00077
Dnahc11	chr12	12.0551	10.6436	1.411	0.00462
Mrpl34	chr8	10.3799	8.96948	1.410	0.02213
Gorasp2	chr2	13.522	12.1121	1.410	0.00428
Mgat4b	chr11	10.6428	9.23305	1.410	0.01215
Srpk1	chr17	14.5962	13.1869	1.409	0.00055
Mllt3	chr4	13.9784	12.5695	1.409	0.00237
Dbf4	chr5	13.6155	12.2081	1.407	0.0011

Gbf1	chr19	14.0778	12.6705	1.407	0.0004
Psma7	chr2	12.2495	10.8423	1.407	0.00165
Tmem128	chr5	8.73372	7.32815	1.406	0.01815
Fbxl12	chr9	10.3336	8.9293	1.404	0.00672
Kctd10	chr5	13.4691	12.0651	1.404	0.00187
Top3a	chr11	12.0083	10.6054	1.403	0.00497
Eif2b1	chr5	12.326	10.9239	1.402	0.00158
Iqsec2	chrX	10.8659	9.46655	1.399	0.00023
Papd5	chr8	13.4512	12.0522	1.399	0.0023
Hivep1	chr13	14.9115	13.5137	1.398	0.00194
Amd1	chr10	13.5413	12.1454	1.396	0.00033
Zcchc9	chr13	10.305	8.9104	1.395	0.01202
Lman2	chr13	11.9964	10.6025	1.394	0.02262
Yipf6	chrX	13.7124	12.3187	1.394	1.4E-06
Zcchc17	chr4	12.3053	10.9123	1.393	0.00698
Igsf3	chr3	14.6208	13.2284	1.392	0.00057
Txndc9	chr1	13.9192	12.527	1.392	0.00027
Sc4mol	chr8	9.44981	8.05851	1.391	0.04069
Noa1	chr5	10.3467	8.9568	1.390	0.01319
Jagn1	chr6	11.1942	9.80617	1.388	0.00636
Dhodh	chr8	10.1306	8.74294	1.388	0.04504
Fam185a	chr5	9.92474	8.53744	1.387	0.0236
Gpr137c	chr14	10.5332	9.14664	1.387	0.0258
Mapre1	chr2	14.1551	12.7692	1.386	0.00049
Usp30	chr5	13.8896	12.5045	1.385	0.00061
Pradc1	chr6	11.1905	9.80537	1.385	0.02814
Prune	chr3	13.0261	11.6425	1.384	0.00338
E130303B06Rik	chr8	9.23214	7.84881	1.383	0.00318
Rfwd2	chr1	14.7571	13.3762	1.381	0.00146
Ttc27	chr17	12.8875	11.5074	1.380	6.8E-05
Traf4	chr11	10.9613	9.58156	1.380	0.00031
Git2	chr5	12.5797	11.2006	1.379	0.00317
Ncaph	chr2	12.8927	11.5145	1.378	0.00192
Mtap4	chr9	15.1637	13.7855	1.378	0.00014
Pigc	chr1	11.7747	10.3987	1.376	0.00581
Piga	chrX	11.8082	10.4324	1.376	0.01343
Ddb1	chr19	15.9612	14.5858	1.375	0.00107
Gm527	chr12	11.0726	9.69806	1.375	0.00994
Supt6h	chr11	15.0943	13.7202	1.374	6.6E-05
Rab11fip5	chr6	11.6353	10.2619	1.373	0.00349
Tada1	chr1	10.5752	9.20357	1.372	0.02564
Ccdc115	chr1	13.4584	12.0878	1.371	0.00179
1110037F02Rik	chr4	13.823	12.4524	1.371	0.00011
Fbxl2	chr9	10.9625	9.59227	1.370	0.01929
Mphosph8	chr14	14.7696	13.3994	1.370	0.00052
Atad5	chr11	12.1459	10.7768	1.369	0.00181
Mafg	chr11	10.6894	9.32047	1.369	0.00632
Tmem199	chr11	11.1295	9.76055	1.369	0.03154
Prpf6	chr2	13.2224	11.8553	1.367	0.00171
Grb10	chr11	11.3854	10.0188	1.367	0.04461
4921524J17Rik	chr8	13.103	11.7374	1.366	0.00151
Rnf114	chr2	16.6305	15.2664	1.364	0.00013
Atl1	chr12	13.0897	11.7264	1.363	0.00224
Taf13	chr3	12.1034	10.7401	1.363	0.00656
Extl3	chr14	13.1223	11.7593	1.363	0.00801
Anapc16	chr10	12.2708	10.9079	1.363	0.01313
Mpp5	chr12	12.591	11.2283	1.363	0.00698

Rce1	chr19	9.87059	8.50797	1.363	0.00409
Nde1	chr16	10.1019	8.74106	1.361	0.00968
Lpcat4	chr2	10.7831	9.42255	1.361	0.004
Supv3l1	chr10	12.0736	10.7146	1.359	0.00524
Pom121	chr5	13.6141	12.2552	1.359	0.00662
Stx18	chr5	11.0447	9.68622	1.359	0.00094
Prepl	chr17	12.0834	10.7258	1.358	0.00222
D11Wsu99e	chr11	11.6229	10.2658	1.357	0.00397
Prex1	chr2	12.6671	11.3101	1.357	0.00078
Med11	chr11	8.82926	7.47236	1.357	0.00227
Tollip	chr7	12.5444	11.1885	1.356	0.00245
Arl13b	chr16	10.3324	8.97685	1.356	0.01363
Clk1	chr1	13.0824	11.7275	1.355	1.7E-05
Zbtb46	chr2	11.3129	9.95866	1.354	0.00741
1810030N24Rik	chr4	10.1767	8.82302	1.354	0.00034
Lamtor3	chr3	13.3317	11.9799	1.352	0.0021
9230115E21Rik	chr9	14.0651	12.7135	1.352	0.00218
Mmab	chr5	10.6227	9.27189	1.351	0.00774
Scmh1	chr4	12.5113	11.1608	1.351	0.01477
Clcn5	chrX	12.2275	10.8779	1.350	0.00283
Akt1s1	chr7	11.5637	10.2151	1.349	0.00342
Pacsin2	chr15	14.7995	13.451	1.349	0.00015
Gtf2h1	chr7	11.5598	10.2147	1.345	0.02504
Galnt3	chr2	9.33423	7.98999	1.344	0.04661
Plekhj1	chr10	10.8648	9.52173	1.343	0.002
Wdr85	chr2	8.95688	7.61562	1.341	0.04469
Cenpf	chr1	16.0255	14.6853	1.340	0.00304
Mad2l2	chr4	10.5575	9.21722	1.340	0.00769
Foxk1	chr5	13.5061	12.1662	1.340	0.00031
Usp21	chr1	11.5617	10.2224	1.339	0.00133
Hook2	chr8	10.9442	9.60498	1.339	6.8E-05
Ipo9	chr1	13.1647	11.8271	1.338	0.0042
Atmin	chr8	13.8145	12.4774	1.337	0.00041
Zfp28	chr7	9.82295	8.48683	1.336	0.00409
Erp44	chr4	11.9922	10.6572	1.335	0.00172
Unk	chr11	12.4444	11.1095	1.335	0.00068
Setdb1	chr3	14.8751	13.5432	1.332	0.0002
Rnf166	chr8	11.4681	10.1369	1.331	0.00159
Zfp963	chr8	9.71519	8.3849	1.330	0.00326
Zcchc3	chr2	12.994	11.6639	1.330	0.00065
Slc25a44	chr3	10.78	9.45044	1.330	0.03105
D19Ertd386e	chr19	11.9853	10.6596	1.326	0.00021
Tpd52	chr3	14.5344	13.2091	1.325	0.00603
Zfp828	chr8	13.5689	12.2441	1.325	0.00085
Akt1	chr12	12.6306	11.3059	1.325	0.00275
Ptov1	chr7	11.6266	10.3027	1.324	0.00651
Srfbp1	chr18	11.2694	9.94598	1.323	0.00047
Cep57	chr9	10.2133	8.89057	1.323	0.04503
Actn3	chr19	12.0358	10.7135	1.322	0.01668
Tmem39b	chr4	12.2174	10.896	1.321	0.00489
Swt1	chr1	13.006	11.6849	1.321	0.00389
Eif2ak1	chr5	13.5082	12.1886	1.320	0.00141
Nudt5	chr2	13.0682	11.7491	1.319	0.00014
Phf6	chrX	13.1499	11.8327	1.317	0.00302
Tbc1d2b	chr9	10.5489	9.233	1.316	0.00999
Abhd16a	chr17	11.7924	10.4767	1.316	0.00808
Sephs2	chr7	11.9528	10.6375	1.315	0.00239

Yipf1	chr4	11.485	10.1703	1.315	0.01535
Tmem116	chr5	8.49451	7.18131	1.313	0.01266
Prkrip1	chr5	12.7216	11.4095	1.312	0.00827
Calr	chr8	15.2878	13.9772	1.311	8.2E-05
Pnma5	chrX	11.496	10.1856	1.310	0.01166
Mtus2	chr5	12.1394	10.8298	1.310	0.00235
Adcy9	chr16	11.3455	10.0379	1.308	0.00836
4930471M23Rik	chr5	10.8215	9.51428	1.307	0.00024
1600027N09Rik	chr2	10.523	9.21608	1.307	0.01769
Zfand5	chr19	13.6579	12.3518	1.306	0.00352
Ip6k2	chr9	12.2013	10.8955	1.306	0.00918
Dnahc7b	chr1	10.9237	9.61867	1.305	0.00135
Tmem50a	chr4	12.4258	11.1208	1.305	0.00083
Sgol2	chr1	12.0085	10.7039	1.305	0.00185
Btbd2	chr10	11.9592	10.6552	1.304	0.02947
Pvrl3	chr16	13.1212	11.8185	1.303	0.00017
Cep76	chr18	10.7234	9.42163	1.302	0.01768
Msh3	chr13	12.6204	11.3185	1.302	0.00684
Unc13b	chr4	13.024	11.7224	1.302	0.0004
Actr1a	chr19	13.3521	12.0514	1.301	0.00101
Rad23b	chr4	15.4728	14.1734	1.299	0.00016
9630033F20Rik	chr6	12.1277	10.8285	1.299	0.0005
Clspn	chr4	13.627	12.328	1.299	0.00068
Zfyve1	chr12	11.0099	9.71085	1.299	0.0052
Phtf2	chr5	13.9306	12.6321	1.299	0.00041
Entpd1	chr19	10.5931	9.29498	1.298	0.00573
Mrpl14	chr17	11.1424	9.84504	1.297	0.01111
Csad	chr15	12.316	11.0195	1.297	0.01631
Scamp2	chr9	11.1283	9.83211	1.296	0.04142
6720456H20Rik	chr14	9.69821	8.40236	1.296	0.01741
Rbm4	chr19	9.84073	8.54544	1.295	0.01879
Sh3kbp1	chrX	13.1126	11.8175	1.295	1.3E-05
Myrip	chr9	12.484	11.1895	1.295	0.00264
Rhot1	chr11	14.4983	13.204	1.294	0.00022
Sgcd	chr11	10.0797	8.78569	1.294	0.00887
Psma1	chr7	12.6732	11.3792	1.294	0.0008
Mcm3ap	chr10	14.2062	12.9126	1.294	0.00042
Commd2	chr3	10.8922	9.60104	1.291	0.02115
Ankrd6	chr4	12.4545	11.1636	1.291	0.0005
Ppp2r2d	chr7	12.8174	11.5264	1.291	0.00147
Mettl9	chr7	11.9621	10.6731	1.289	0.00316
Uhrf1	chr17	15.9703	14.6838	1.287	0.00372
1500003O03Rik	chr2	12.0853	10.7991	1.286	0.03947
Ranbp9	chr13	14.9752	13.689	1.286	5.4E-05
Gm14420	chr2	10.4789	9.19357	1.285	0.00972
Zfyve21	chr12	11.4325	10.1475	1.285	0.00651
Rhbdf2	chr11	11.4595	10.1749	1.285	0.00398
Mtfmt	chr9	9.37572	8.09137	1.284	0.01071
Cab39l	chr14	11.7395	10.456	1.283	0.01812
Gnai2	chr9	12.9515	11.6692	1.282	0.00621
Paip2	chr18	14.765	13.4838	1.281	0.0007
Samd8	chr14	13.1412	11.8602	1.281	0.00172
Dock7	chr4	13.3345	12.0539	1.281	0.00113
Hdac8	chrX	9.64387	8.36352	1.280	0.035
Med10	chr13	11.2298	9.95067	1.279	0.03199
Lrrc28	chr7	11.863	10.584	1.279	0.01029
Zfp654	chr16	12.1326	10.8549	1.278	0.0018

Cdkl2	chr5	10.7143	9.43698	1.277	0.01867
Uba3	chr6	12.4472	11.1699	1.277	0.00051
Prkaa1	chr15	11.8016	10.5246	1.277	0.03231
Ppapdc2	chr19	9.09797	7.82199	1.276	0.0287
Pdia6	chr12	14.1689	12.8966	1.272	0.00022
Zfp936	chr7	10.6084	9.33928	1.269	0.00418
Noto	chr6	10.5906	9.32175	1.269	0.02034
D17Wsu92e	chr17	13.7932	12.5246	1.269	0.00051
Tpd52l2	chr2	12.8318	11.5634	1.268	0.00107
Trnau1ap	chr4	9.77437	8.50646	1.268	0.03715
Ssb	chr2	14.9101	13.643	1.267	0.00071
9430023L20Rik	chr15	11.1773	9.91107	1.266	0.01743
Snx13	chr12	11.9639	10.6978	1.266	0.02757
Ap1m2	chr9	12.4178	11.1519	1.266	0.00669
Pcid2	chr8	11.097	9.83242	1.265	0.00377
Tmem5	chr10	11.9856	10.7214	1.264	0.01262
Lin28b	chr10	10.9616	9.69831	1.263	0.00499
Dctn5	chr7	13.1216	11.859	1.263	0.0055
Psen1	chr12	13.5604	12.2983	1.262	0.00324
Nbr1	chr11	13.7283	12.4664	1.262	0.01297
Itgb5	chr16	9.27155	8.01097	1.261	0.01828
Sdc1	chr12	13.9609	12.7006	1.260	0.00065
Dcaf5	chr12	13.4884	12.2288	1.260	8.7E-06
Trim32	chr4	11.2078	9.94999	1.258	0.00176
Dopey1	chr9	13.8278	12.5703	1.258	0.00122
Lsm14b	chr2	15.9646	14.7071	1.257	0.00063
Pxn	chr5	11.1642	9.90675	1.257	0.00318
Hn1	chr11	12.3973	11.1426	1.255	0.01465
3200002M19Rik	chr7	12.3711	11.1189	1.252	0.00581
Ston2	chr12	12.8759	11.6241	1.252	0.00096
Smc6	chr12	13.7413	12.4908	1.251	8.8E-05
Rpap3	chr15	11.5819	10.3318	1.250	0.01384
Ptbp1	chr10	13.5714	12.3214	1.250	0.00035
Psme3	chr11	14.8408	13.5924	1.248	0.00015
Bend5	chr4	10.0811	8.83367	1.247	0.01782
Ciapin1	chr8	12.1873	10.9413	1.246	0.00893
Plag1	chr4	11.5526	10.3077	1.245	0.0452
Enpp5	chr17	12.0173	10.7734	1.244	0.00216
Tshz1	chr18	13.1664	11.9227	1.244	0.00041
Skp2	chr15	10.4272	9.18403	1.243	0.00952
Siva1	chr12	12.81	11.5678	1.242	0.0205
Gpatch8	chr11	13.4608	12.2189	1.242	0.00665
Prc1	chr7	14.7055	13.4637	1.242	0.00389
Lpgat1	chr1	12.8075	11.566	1.241	0.00324
Wrn	chr8	11.9122	10.6715	1.241	0.00111
Kif20b	chr19	13.4223	12.1822	1.240	0.00713
Rnf26	chr9	12.6699	11.4318	1.238	0.00038
Zufsp	chr10	11.7507	10.513	1.238	0.03209
Plekhg5	chr4	10.4198	9.18205	1.238	0.01871
Rhobtb2	chr14	11.5044	10.2676	1.237	0.0473
Rbm3	chrX	11.3686	10.1322	1.236	0.02669
Pard6a	chr8	9.86232	8.62599	1.236	0.02397
Ddx3x	chrX	16.3069	15.0708	1.236	2.9E-05
Cdc40	chr10	11.7464	10.5115	1.235	0.01254
Desi2	chr1	13.4317	12.1987	1.233	0.00159
Szrd1	chr4	11.075	9.8424	1.233	0.02985
Sec16a	chr2	14.326	13.0935	1.233	0.00158

Mms22l	chr4	12.2222	10.9901	1.232	0.00051
Bicd2	chr13	12.8415	11.6094	1.232	0.00464
Vcpip1	chr1	14.274	13.0422	1.232	0.01111
Kpna2	chr11	15.2285	14.0006	1.228	9.1E-05
Elk4	chr1	12.6737	11.448	1.226	0.00115
Nrg1	chr8	10.7242	9.5003	1.224	0.00974
Casc1	chr6	12.1418	10.9184	1.223	5E-05
Tsc22d2	chr3	11.8923	10.669	1.223	0.00424
Emc3	chr6	10.8862	9.6636	1.223	0.00891
9530068E07Rik	chr11	12.0807	10.8592	1.221	0.00036
Cdk5	chr5	12.0082	10.7872	1.221	0.00101
D230025D16Rik	chr8	11.7363	10.5169	1.219	0.00507
Secisbp2l	chr2	13.5847	12.3655	1.219	0.00046
Rrp8	chr7	11.7213	10.5028	1.218	0.03137
Tmem242	chr17	9.41014	8.19217	1.218	0.00952
Mis12	chr11	12.5279	11.3101	1.218	0.02023
Hif1a	chr12	14.346	13.1284	1.218	0.00046
Mrps33	chr6	11.9134	10.6964	1.217	0.0097
Med6	chr12	10.9239	9.70759	1.216	0.04861
Lmo7	chr14	15.5118	14.2959	1.216	0.00309
Mctp2	chr7	13.8235	12.6077	1.216	0.00014
Arhgef7	chr8	13.6027	12.387	1.216	0.00553
Csnk1e	chr15	15.0264	13.8107	1.216	0.00173
Stim2	chr5	11.1933	9.97799	1.215	0.0016
Mrps22	chr9	11.3939	10.1798	1.214	0.0029
Btd	chr14	9.68492	8.47102	1.214	0.03546
Cwf19l2	chr9	12.0533	10.8416	1.212	0.00261
Ccnf	chr17	12.1781	10.9669	1.211	0.02095
Elf3	chr1	10.0862	8.87605	1.210	0.00368
Lmf2	chr15	10.8491	9.64043	1.209	0.02756
Wdr75	chr1	12.588	11.3803	1.208	0.00635
Gda	chr19	13.6404	12.433	1.207	0.01513
Ppp1r37	chr7	11.4016	10.1946	1.207	0.00618
Myo10	chr15	15.5682	14.3639	1.204	0.0003
Fam65a	chr8	10.4224	9.2189	1.203	0.01208
Cyth2	chr7	12.0345	10.8316	1.203	0.01616
Pex14	chr4	11.2362	10.034	1.202	0.01344
Cirh1a	chr8	14.2572	13.057	1.200	0.00358
Top2a	chr11	13.1586	11.9585	1.200	0.00333
1700020O03Rik	chr12	12.1377	10.9382	1.200	0.00292
Srp54c	chr12	13.921	12.7222	1.199	0.00043
Tgfbr3	chr5	11.2416	10.0429	1.199	0.03218
Bod1l	chr5	14.3115	13.1128	1.199	0.00422
Cpeb1	chr7	15.4562	14.2579	1.198	0.00132
Ino80	chr2	12.8534	11.6564	1.197	0.00061
Pced1b	chr15	10.4769	9.2808	1.196	0.00411
Tmem29	chrX	11.3929	10.1972	1.196	0.00839
Med27	chr2	12.54	11.3446	1.195	0.00156
Apip	chr2	10.7125	9.51717	1.195	0.0311
Sap30l	chr11	11.1356	9.94075	1.195	0.01656
Chdh	chr14	13.303	12.1093	1.194	0.00346
Fxr2	chr11	14.2183	13.0262	1.192	0.00125
Tmem104	chr11	10.4266	9.23517	1.191	0.00053
Gga3	chr11	12.5668	11.3777	1.189	0.01802
Tox	chr4	9.82691	8.6391	1.188	0.01876
Max	chr12	13.5634	12.3756	1.188	1.8E-05
Csda	chr6	15.0618	13.8747	1.187	0.00218

Mybl2	chr2	14.1862	12.9999	1.186	0.00082
Tspyl1	chr10	12.859	11.6731	1.186	0.00164
2010321M09Rik	chr9	11.187	10.0014	1.186	0.00288
Smurf1	chr5	12.7565	11.571	1.186	0.00605
Bcl2l11	chr2	11.0148	9.82942	1.185	0.00248
Pigy	chr6	8.46337	7.27934	1.184	0.00973
6030443J06Rik	chr5	9.52113	8.33711	1.184	0.03341
Sec22a	chr16	13.0408	11.8568	1.184	0.00015
Smg8	chr11	13.0958	11.9145	1.181	0.01006
Mkln1	chr6	13.0741	11.8933	1.181	0.00052
Rnf8	chr17	14.5437	13.3638	1.180	0.00119
Rusc2	chr4	10.1886	9.00923	1.179	0.00173
Ss18l1	chr2	12.602	11.4245	1.178	0.01339
Ttc33	chr15	11.8619	10.6844	1.177	0.00399
Rcor1	chr12	11.2756	10.0984	1.177	0.02593
Grhl2	chr15	8.31379	7.13667	1.177	0.0027
Gnpnat1	chr14	11.1828	10.0091	1.174	0.0193
Pik3r4	chr9	14.1589	12.9856	1.173	0.00046
Slc6a20a	chr9	9.40372	8.23109	1.173	0.00175
Ppm1l	chr3	10.7145	9.5432	1.171	0.02844
Tpra1	chr6	12.8564	11.6852	1.171	0.00843
Zfp748	chr13	10.7822	9.61275	1.169	0.00593
2210009G21Rik	chr2	11.0403	9.87184	1.168	0.03109
Zkscan5	chr5	11.8181	10.6499	1.168	0.01614
Gltpd1	chr4	10.115	8.94785	1.167	0.0236
Nav2	chr7	14.6431	13.4771	1.166	0.00722
Zdhhc5	chr2	12.8021	11.6366	1.166	0.0151
Ccnl2	chr4	12.0673	10.9024	1.165	0.00428
Add3	chr19	12.283	11.119	1.164	0.02701
Cdca4	chr12	12.372	11.2084	1.164	0.00239
Ppp1r15a	chr7	12.1732	11.0114	1.162	0.04015
Pdcl3	chr1	12.9349	11.7738	1.161	0.00195
Mll2	chr15	15.2346	14.0739	1.161	0.00553
Aff1	chr5	12.7995	11.6389	1.161	0.00037
Atg4b	chr1	12.1888	11.0295	1.159	0.00926
Anapc4	chr5	13.397	12.2388	1.158	0.00459
Bcar1	chr8	11.9143	10.7564	1.158	0.00637
Jmjd6	chr11	11.348	10.1906	1.157	0.01772
Rnf213	chr11	11.3292	10.1721	1.157	0.00591
Mcm9	chr10	11.0944	9.93751	1.157	0.00461
Pcbp1	chr6	12.4047	11.2483	1.156	0.04303
Pex1	chr5	11.6999	10.5439	1.156	0.00063
4931406P16Rik	chr7	13.7089	12.5542	1.155	0.00026
2810474O19Rik	chr6	13.9266	12.7725	1.154	0.00026
Acd	chr8	11.816	10.6637	1.152	0.01295
Cd164l2	chr4	12.9355	11.7835	1.152	0.00195
Sepw1	chr7	11.8489	10.6971	1.152	0.01276
Psd3	chr8	12.7155	11.5649	1.151	0.00024
Fiz1	chr7	11.7425	10.5923	1.150	0.01544
Rbm12b1	chr4	10.5038	9.35369	1.150	0.0185
Ccdc34	chr2	10.9517	9.80169	1.150	0.01912
Agps	chr2	12.7227	11.5728	1.150	0.00794
Cd55	chr1	13.7182	12.5685	1.150	0.03004
Orc6	chr8	12.1784	11.0288	1.150	0.0016
Tmem41b	chr7	13.3828	12.2333	1.149	0.00484
Nol9	chr4	12.1031	10.9541	1.149	0.00658
Akap10	chr11	12.1868	11.038	1.149	0.02175

Nfyc	chr4	12.8376	11.6889	1.149	3.9E-06
Arid1b	chr17	14.5253	13.3769	1.148	0.00457
E2f5	chr3	14.1609	13.0134	1.147	0.00053
Atf7ip	chr6	13.459	12.3124	1.147	0.00159
Trpc2	chr7	11.2241	10.0779	1.146	0.03332
Nosip	chr7	11.3496	10.2039	1.146	0.0138
Gm6792	chr7	8.4493	7.30484	1.144	0.00042
Dda1	chr8	11.4473	10.3034	1.144	0.01485
Terf1	chr1	13.0849	11.9428	1.142	0.00128
Ahr	chr12	11.1612	10.0197	1.142	0.00851
Tubb2b	chr13	14.1357	12.9944	1.141	0.01532
Ttc7	chr17	9.60668	8.4662	1.140	0.00074
Gm6086	chr1	9.3153	8.17485	1.140	0.04516
Ccdc174	chr6	12.238	11.0978	1.140	0.01181
Psme4	chr11	14.3341	13.1943	1.140	0.0011
Ing5	chr1	11.585	10.4453	1.140	0.0006
Ube2w	chr1	12.9333	11.794	1.139	0.00621
Sgms1	chr19	11.0076	9.86978	1.138	0.0165
Zbtb48	chr4	10.2793	9.14159	1.138	0.04092
Mgat1	chr11	11.5129	10.3755	1.137	0.00601
Phtf1	chr3	11.1834	10.0462	1.137	0.02478
Pcyox1	chr6	12.2328	11.0959	1.137	0.0026
Rnf4	chr5	14.1545	13.018	1.136	0.00155
Asf1a	chr10	12.935	11.7986	1.136	0.00084
Tm2d1	chr4	11.7675	10.6317	1.136	0.02614
H3f3a	chr1	14.0013	12.8658	1.135	0.00443
Trpc4ap	chr2	13.0234	11.888	1.135	0.00907
Dgcr14	chr16	12.249	11.1141	1.135	0.00236
Reep4	chr14	10.1528	9.01937	1.133	0.00931
Farsa	chr8	11.7192	10.5865	1.133	0.00342
Kidins220	chr12	13.8312	12.6989	1.132	5.3E-05
C1galt1c1	chrX	9.20067	8.06835	1.132	0.01827
Qtrtd1	chr16	10.5719	9.44024	1.132	0.0174
Dynlt1c	chr17	9.21925	8.08767	1.132	0.01113
Acp6	chr3	11.4976	10.3672	1.130	0.00788
Zc3h4	chr7	13.0463	11.916	1.130	0.00049
Trio	chr15	13.6981	12.5688	1.129	0.00148
Zc3h18	chr8	13.3058	12.1776	1.128	0.00341
Srp54b	chr12	13.9129	12.785	1.128	0.00754
Nfkbib	chr7	10.9942	9.86739	1.127	0.00967
Gpt2	chr8	10.6808	9.55421	1.127	0.0164
Hist1h2bc	chr13	10.47	9.34483	1.125	0.00177
Trip12	chr1	16.4734	15.3486	1.125	0.00799
Sar1b	chr11	11.8764	10.752	1.124	0.00308
Slc6a9	chr4	14.8394	13.7157	1.124	0.00111
Pcgf6	chr19	13.6778	12.5548	1.123	0.00134
Tdrkh	chr3	12.3609	11.2405	1.120	0.00422
Srp54a	chr12	14.2853	13.1663	1.119	5.8E-05
Rab35	chr5	12.1438	11.0249	1.119	0.00342
Kdelr1	chr7	12.0976	10.9788	1.119	0.00483
Raver1-fdx1l	chr9	12.0914	10.9729	1.119	0.0003
Pde8b	chr13	9.27172	8.15327	1.118	0.00112
Sdhd	chr9	13.2266	12.1099	1.117	0.00283
Ngly1	chr14	12.2794	11.1635	1.116	0.00642
Recql5	chr11	12.3292	11.2139	1.115	0.03871
Pak1ip1	chr13	13.6693	12.5547	1.115	4.7E-05
Nlrp4f	chr13	16.3652	15.2514	1.114	0.00097

Trmt12	chr15	11.0325	9.91891	1.114	0.01981
Bmx	chrX	8.59903	7.48566	1.113	0.00128
Utp11l	chr4	11.4986	10.3853	1.113	0.00049
Slc52a2	chr15	11.4602	10.347	1.113	0.00383
Spire1	chr18	14.2027	13.0899	1.113	0.0009
Ankrd26	chr6	12.109	10.9975	1.112	0.04423
Msl1	chr11	13.4915	12.3801	1.111	0.00301
Smarca2	chr19	14.3342	13.2232	1.111	0.00431
Ino80b	chr6	10.3641	9.25366	1.110	0.01921
Ssna1	chr2	10.7012	9.59096	1.110	0.00524
Mtmr4	chr11	12.9753	11.865	1.110	0.00306
Msl2	chr9	12.51	11.4009	1.109	0.00534
Trnt1	chr6	12.3142	11.206	1.108	0.00207
Rmi1	chr13	12.5423	11.435	1.107	0.00218
2410012M07Rik	chr9	10.1419	9.03593	1.106	0.01805
Psip1	chr4	14.6595	13.5535	1.106	0.01419
9130023H24Rik	chr7	9.84784	8.74359	1.104	0.01038
Synj1	chr16	13.7974	12.6936	1.104	0.00163
Psenen	chr7	12.084	10.981	1.103	0.00153
Acacb	chr5	12.7773	11.6755	1.102	0.00509
Myst4	chr14	13.6523	12.5519	1.100	0.00976
Slc25a15	chr8	13.3181	12.2182	1.100	0.01256
Lrrc49	chr9	9.81041	8.71203	1.098	0.04963
Ninj1	chr13	12.6177	11.52	1.098	0.00706
Cdca7l	chr12	11.5774	10.4802	1.097	0.00189
1500011B03Rik	chr5	10.8282	9.73119	1.097	0.00017
2310036O22Rik	chr8	10.6773	9.5807	1.097	0.00109
Dph3	chr14	10.6908	9.59458	1.096	0.02752
Bfar	chr16	11.8871	10.7919	1.095	0.0081
Qsox1	chr1	10.8853	9.79084	1.094	0.01035
Imp3	chr9	10.7117	9.61729	1.094	0.01734
Rnaset2b	chr17	12.908	11.8141	1.094	0.00461
Erlin1	chr19	11.8954	10.8027	1.093	0.02005
1110004E09Rik	chr16	12.6242	11.5322	1.092	0.00383
Narg2	chr9	12.6746	11.5827	1.092	0.01477
Tanc1	chr2	13.8286	12.7381	1.091	0.0026
Hmgxb3	chr18	12.2462	11.1564	1.090	0.00365
Tbc1d10a	chr11	10.0195	8.93054	1.089	0.04563
Aes	chr10	10.2827	9.19393	1.089	0.03512
Gtf2a1	chr12	14.3463	13.2578	1.088	0.00755
Lin28a	chr4	12.9434	11.8553	1.088	0.0027
Ddb2	chr2	11.6704	10.5825	1.088	0.00321
Mapk8	chr14	13.6487	12.561	1.088	0.00039
Zdhhc12	chr2	8.55844	7.47193	1.087	0.03916
Fam122a	chr19	10.5985	9.51253	1.086	0.01155
Mon2	chr10	13.6675	12.5818	1.086	0.00145
C030046E11Rik	chr19	13.909	12.8239	1.085	0.00134
Phf20	chr2	14.2892	13.2041	1.085	0.00013
Zmpste24	chr4	12.7311	11.6461	1.085	0.00024
Smn1	chr13	12.4078	11.3231	1.085	0.00556
Fbrs	chr7	11.4486	10.3641	1.084	0.02782
Trappc5	chr8	12.6315	11.5475	1.084	0.00194
Ralgapb	chr2	13.672	12.5883	1.084	0.00053
Ccdc60	chr5	10.7934	9.7103	1.083	0.00094
Nup54	chr5	12.6225	11.5403	1.082	0.01525
Plrg1	chr3	13.4147	12.3335	1.081	2.6E-05
Lrig2	chr3	12.8078	11.7271	1.081	0.00324

Fem1a	chr17	10.4846	9.40461	1.080	0.01333
Zfp839	chr12	13.0257	11.946	1.080	0.02777
Hmgcr	chr13	12.8119	11.7322	1.080	0.00756
Hexim2	chr11	11.593	10.5133	1.080	0.00223
Sap18	chr14	14.3902	13.3106	1.080	0.00416
Nup107	chr10	14.2405	13.1621	1.078	0.00057
Tpi1	chr6	11.2352	10.1572	1.078	0.03331
Acad11	chr9	11.3558	10.278	1.078	0.01351
Ahctf1	chr1	13.7111	12.6335	1.078	0.00207
Zbtb39	chr10	11.3749	10.2974	1.077	0.00232
Cds1	chr5	13.5308	12.454	1.077	0.00331
Rltpr	chr8	9.94126	8.86447	1.077	0.0184
Zfp87	chr13	11.8196	10.7437	1.076	0.00339
D330028D13Rik	chr6	9.79259	8.71778	1.075	0.04836
Tbc1d10b	chr7	13.1408	12.0677	1.073	0.00193
Skp1a	chr11	16.0826	15.0098	1.073	0.00011
Pcnxl4	chr12	10.7925	9.72031	1.072	0.00343
Tpmt	chr13	10.6974	9.62638	1.071	0.00901
Golga2	chr2	13.3242	12.2535	1.071	0.02294
Dennd4a	chr9	11.8867	10.8171	1.070	0.01359
Gstp1	chr19	9.40922	8.3403	1.069	0.02188
Cpsf4	chr5	11.2457	10.1768	1.069	0.02115
Ddx56	chr11	11.7842	10.7153	1.069	0.01249
Lrrc40	chr3	13.3268	12.2589	1.068	8.5E-05
Taok1	chr11	14.3928	13.3255	1.067	0.00875
Mtif3	chr5	12.084	11.0173	1.067	0.00936
Ppwd1	chr13	12.2361	11.1695	1.067	0.0239
Rae1	chr2	12.3548	11.2883	1.067	0.0001
U2af1l4	chr7	11.2534	10.188	1.065	0.0111
Rdx	chr9	17.7155	16.6506	1.065	3.2E-05
Mepce	chr5	13.3536	12.2889	1.065	0.01282
Abi1	chr2	13.685	12.6237	1.061	0.0007
Wdr76	chr2	13.9873	12.9273	1.060	0.00011
Brca2	chr5	14.6709	13.6115	1.059	0.0022
Tmco3	chr8	10.7604	9.70124	1.059	0.0043
Slc7a2	chr8	14.0292	12.9705	1.059	0.01576
Stk36	chr1	13.9496	12.8913	1.058	0.02599
Pex26	chr6	11.6739	10.6159	1.058	0.0004
Inpp5d	chr1	10.8828	9.82524	1.058	0.00978
Actr2	chr11	13.804	12.75	1.054	0.00925
Tubd1	chr11	10.4961	9.44565	1.050	0.00079
Txlng	chrX	12.9753	11.9256	1.050	0.00362
Sema4d	chr13	12.0089	10.9603	1.049	0.00043
Wdr25	chr12	11.0776	10.0293	1.048	0.00201
Rad51c	chr11	11.8477	10.8004	1.047	0.02672
Cyb561d2	chr9	10.638	9.59418	1.044	0.01088
Llgl1	chr11	10.2156	9.17218	1.043	0.04229
Men1	chr19	11.9528	10.9098	1.043	0.02949
Erp29	chr5	10.9223	9.87995	1.042	0.00632
C1qtnf5	chr9	11.3917	10.3505	1.041	0.0083
Pnpla2	chr7	10.5297	9.48969	1.040	0.00919
Rab23	chr1	11.9712	10.9333	1.038	0.01882
Ksr2	chr5	10.8858	9.84812	1.038	0.00683
Bub3	chr7	12.3925	11.3556	1.037	0.01021
Scml2	chrX	13.2917	12.2551	1.037	0.00556
Samd1	chr8	11.7131	10.6773	1.036	0.01286
Lrrc57	chr2	11.0818	10.0462	1.036	0.04596

Pdia4	chr6	13.2797	12.2449	1.035	0.00309
Hmgn5	chrX	10.7457	9.71215	1.034	0.00604
Shb	chr4	11.3376	10.3055	1.032	0.01036
Mis18a	chr16	12.1584	11.1272	1.031	0.00884
Socs6	chr18	12.6836	11.6534	1.030	0.02528
Ankrd54	chr15	8.46257	7.43396	1.029	0.0096
Ttll4	chr1	12.7892	11.7607	1.028	0.00361
Sf3a1	chr11	13.9939	12.9655	1.028	0.02333
Nup62-il4i1	chr7	14.0955	13.0683	1.027	0.00681
Emc6	chr11	10.7243	9.697	1.027	0.01216
Khsrp	chr17	14.1737	13.1467	1.027	0.0218
Lama4	chr10	11.1263	10.0995	1.027	0.01745
Tank	chr2	10.3535	9.32707	1.026	0.00643
Asxl1	chr2	15.0569	14.0307	1.026	6.7E-05
Med4	chr14	12.7861	11.7601	1.026	0.01664
Psma2	chr13	13.7847	12.7588	1.026	0.01029
Lipe	chr7	9.2999	8.27457	1.025	0.02215
Ctcf	chr8	13.3361	12.3115	1.025	0.0003
Tdeanc2	chr4	11.4166	10.3923	1.024	0.00487
Alms1	chr6	13.2803	12.2564	1.024	0.00478
Mki67	chr7	14.7285	13.7053	1.023	0.01245
Tti1	chr2	12.6708	11.6486	1.022	0.02967
Vti1a	chr19	11.1703	10.1505	1.020	0.00842
Mphosph10	chr7	13.555	12.5354	1.020	0.00025
Rbm6	chr9	13.8655	12.8475	1.018	0.00154
Gnai3	chr3	13.3405	12.3229	1.018	0.00036
Dynlt1f	chr17	9.15154	8.13412	1.017	0.01789
Klc1	chr12	11.687	10.67	1.017	0.01105
Fance	chr17	10.3233	9.30799	1.015	0.01047
Sgol1	chr17	11.3505	10.3356	1.015	0.03103
Nlk	chr11	12.3111	11.2964	1.015	0.01681
Amn1	chr6	11.6284	10.6139	1.015	0.00091
Fbxl19	chr7	13.2869	12.2725	1.014	0.04757
Atxn7l2	chr3	9.97171	8.95808	1.014	0.03837
Usp15	chr10	14.7516	13.7389	1.013	0.00024
Osbpl5	chr7	8.26511	7.25273	1.012	0.02814
Rheb	chr5	11.6713	10.6591	1.012	0.00066
Rbm12b2	chr4	10.9946	9.983	1.012	0.00282
Stt3b	chr9	15.337	14.3257	1.011	0.01009
Prpsap2	chr11	12.2322	11.2212	1.011	0.01122
6030458C11Rik	chr15	13.2215	12.211	1.010	0.00692
Dguok	chr6	10.1147	9.10458	1.010	0.04752
Tm7sf3	chr6	13.3728	12.3636	1.009	0.00148
Gm12942	chr4	10.4684	9.45984	1.009	0.01685
Rbfox2	chr15	13.541	12.5345	1.006	0.00452
Zfp526	chr7	10.6688	9.66342	1.005	0.00958
Dnajc21	chr15	15.0848	14.0801	1.005	0.02676
Spats2	chr15	13.3231	12.3199	1.003	0.00637
Gde1	chr7	9.95793	8.95483	1.003	0.0089
AI314180	chr4	15.4113	14.4091	1.002	0.00487
Baz1b	chr5	14.815	13.8131	1.002	0.00011
Tjap1	chr17	11.6028	10.6009	1.002	0.0277
Drap1	chr19	13.4425	12.4408	1.002	0.0015
Tmem149	chr7	11.3126	10.3111	1.001	0.02073
Tex264	chr9	12.2981	11.2969	1.001	0.00188
Klhl28	chr12	11.1509	10.1498	1.001	0.03223

Supplementary Table 2.

GO	Fold	Term	PValue	Bonferroni	Benjamini	FDR
Biological process	> 10 fold	GO:0045449~regulation of transcription	4.35E-07	9.29E-04	9.29E-04	7.53E-04
		GO:0006350~transcription	5.59E-06	0.0118858	0.00596066	0.00969
	4-10 fold	GO:0030163~protein catabolic process	8.74E-07	0.0017858	0.00178583	0.00151
		GO:0009057~macromolecule catabolic process	2.00E-06	0.0040774	0.00204076	0.00345
		GO:0019941~modification-dependent protein cata	2.02E-06	0.0041139	0.00137317	0.00348
		GO:0043632~modification-dependent macromole	2.02E-06	0.0041139	0.00137317	0.00348
		GO:0044265~cellular macromolecule catabolic pr	2.51E-06	0.0051073	0.00127928	0.00432
		GO:0051603~proteolysis involved in cellular prote	3.45E-06	0.0070228	0.00140852	0.00594
		GO:0044257~cellular protein catabolic process	3.98E-06	0.0080969	0.00135405	0.00686
		GO:0015031~protein transport	3.32E-05	0.0656738	0.0096573	0.05727
		GO:0045184~establishment of protein localization	4.04E-05	0.0793113	0.010276	0.06966
		GO:0019318~hexose metabolic process	4.67E-05	0.0910572	0.01055205	0.08048
	2-4 fold	GO:0006412~translation	3.69E-23	8.43E-20	8.43E-20	6.44E-20
		GO:0006091~generation of precursor metabolites	1.72E-09	3.93E-06	1.96E-06	3.00E-06
		GO:0006396~RNA processing	1.26E-08	2.88E-05	9.61E-06	2.20E-05
		GO:0022900~electron transport chain	1.98E-07	4.52E-04	1.13E-04	3.45E-04
		GO:0046907~intracellular transport	3.30E-06	0.0075052	0.00150557	0.00576
		GO:0016197~endosome transport	4.43E-06	0.010073	0.00168592	0.00774
		GO:0034660~ncRNA metabolic process	3.48E-05	0.0763558	0.01128278	0.06068
		GO:0006796~phosphate metabolic process	5.38E-05	0.1156312	0.01524277	0.09385
		GO:0006793~phosphorus metabolic process	5.38E-05	0.1156312	0.01524277	0.09385

GO	Fold	Term	PValue	Bonferroni	Benjamini	FDR
Cellular component	> 10 fold	GO:0005739~mitochondrion	3.51E-05	0.012716	0.01271596	0.04824
		GO:0031967~organelle envelope	4.06E-05	0.0147036	0.007379	0.05583
		GO:0031975~envelope	4.54E-05	0.0164174	0.00550268	0.06239
	4-10 fold	GO:0043228~non-membrane-bounded organelle	6.38E-09	2.54E-06	2.54E-06	8.89E-06
		GO:0043232~intracellular non-membrane-bounde	6.38E-09	2.54E-06	2.54E-06	8.89E-06
		GO:0070013~intracellular organelle lumen	7.00E-09	2.79E-06	1.40E-06	9.76E-06
		GO:0043233~organelle lumen	7.89E-09	3.15E-06	1.05E-06	1.10E-05
		GO:0031974~membrane-enclosed lumen	1.67E-08	6.67E-06	1.67E-06	2.33E-05
		GO:0031981~nuclear lumen	3.24E-07	1.29E-04	2.58E-05	4.51E-04
		GO:0030529~ribonucleoprotein complex	5.93E-07	2.37E-04	3.95E-05	8.27E-04
		GO:0005840~ribosome	5.11E-06	0.0020359	2.91E-04	0.00712
		GO:0005654~nucleoplasm	6.64E-06	0.0026453	3.31E-04	0.00926
		GO:0005739~mitochondrion	1.70E-05	0.0067721	7.55E-04	0.02374
		GO:0005730~nucleolus	2.73E-05	0.0108456	0.00108989	0.0381
	2-4 fold	GO:0030529~ribonucleoprotein complex	4.27E-32	1.90E-29	1.90E-29	6.05E-29
		GO:0005840~ribosome	9.96E-27	4.43E-24	2.22E-24	1.41E-23
		GO:0005739~mitochondrion	4.00E-20	1.78E-17	5.93E-18	5.66E-17
		GO:0043228~non-membrane-bounded organelle	1.91E-17	8.51E-15	2.13E-15	2.71E-14
		GO:0043232~intracellular non-membrane-bounde	1.91E-17	8.51E-15	2.13E-15	2.71E-14
		GO:0044429~mitochondrial part	2.55E-15	1.14E-12	2.27E-13	3.62E-12
		GO:0031974~membrane-enclosed lumen	7.79E-14	3.47E-11	5.78E-12	1.10E-10
		GO:0005740~mitochondrial envelope	1.70E-13	7.58E-11	1.08E-11	2.41E-10
		GO:0031090~organelle membrane	2.73E-13	1.21E-10	1.52E-11	3.87E-10
		GO:0031967~organelle envelope	1.07E-12	4.76E-10	5.29E-11	1.52E-09
		GO:0031975~envelope	1.29E-12	5.73E-10	5.73E-11	1.82E-09
		GO:0070013~intracellular organelle lumen	1.92E-12	8.54E-10	7.76E-11	2.72E-09
		GO:0043233~organelle lumen	2.30E-12	1.02E-09	8.53E-11	3.26E-09
		GO:0031966~mitochondrial membrane	4.98E-12	2.22E-09	1.71E-10	7.06E-09
		GO:0031981~nuclear lumen	2.57E-09	1.14E-06	8.16E-08	3.64E-06
		GO:0005743~mitochondrial inner membrane	2.07E-08	9.23E-06	6.15E-07	2.94E-05
		GO:0019866~organelle inner membrane	3.34E-08	1.48E-05	9.28E-07	4.73E-05
		GO:0070469~respiratory chain	6.12E-08	2.72E-05	1.60E-06	8.67E-05
		GO:0033279~ribosomal subunit	2.56E-06	0.0011372	6.32E-05	0.00362
		GO:0005829~cytosol	7.89E-06	0.0035037	1.85E-04	0.01117
		GO:0005654~nucleoplasm	1.34E-05	0.0059496	2.98E-04	0.01899
		GO:0005730~nucleolus	5.54E-05	0.0243321	0.00117231	0.07838

GO:0012505~endomembrane system	6.28E-05	0.0275632	0.00126966	0.08893

GO	Fold	Term	PValue	Bonferroni	Benjamini	FDR
Molecular function	> 10 fold	GO:0008270~zinc ion binding	1.48E-06	9.93E-04	9.93E-04	0.00222
		GO:0043169~cation binding	5.89E-06	0.0039454	0.00197463	0.00883
		GO:0046914~transition metal ion binding	6.47E-06	0.0043315	0.00144591	0.0097
		GO:0043167~ion binding	1.50E-05	0.0099905	0.00250702	0.02244
		GO:0046872~metal ion binding	1.56E-05	0.010429	0.00209457	0.02343
		GO:0003677~DNA binding	3.84E-05	0.025456	0.00428839	0.05761
	4-10 fold	GO:0008270~zinc ion binding	3.27E-07	2.18E-04	2.18E-04	4.89E-04
		GO:0046914~transition metal ion binding	1.19E-06	7.91E-04	3.95E-04	0.00178
		GO:0000166~nucleotide binding	2.54E-06	0.001691	5.64E-04	0.00381
		GO:0016879~ligase activity, forming carbon-nitrog	3.96E-06	0.0026347	6.59E-04	0.00593
		GO:0003735~structural constituent of ribosome	9.74E-06	0.0064685	0.00129706	0.0146
		GO:0005524~ATP binding	1.00E-05	0.0066453	0.00111063	0.015
		GO:0032559~adenyl ribonucleotide binding	1.64E-05	0.0108714	0.00156034	0.02458
		GO:0032553~ribonucleotide binding	2.09E-05	0.0138285	0.00173911	0.03132
		GO:0032555~purine ribonucleotide binding	2.09E-05	0.0138285	0.00173911	0.03132
		GO:0030554~adenyl nucleotide binding	2.64E-05	0.0174044	0.00194895	0.03948
		GO:0017076~purine nucleotide binding	3.06E-05	0.0201919	0.00203777	0.04587
		GO:0019787~small conjugating protein ligase acti	3.17E-05	0.0209141	0.0019196	0.04753
		GO:0001883~purine nucleoside binding	3.78E-05	0.0248309	0.00209317	0.05654
		GO:0016881~acid-amino acid ligase activity	3.97E-05	0.0260829	0.00203094	0.05943
		GO:0001882~nucleoside binding	4.75E-05	0.0311126	0.00225509	0.07107
	2-4 fold	GO:0003735~structural constituent of ribosome	1.38E-24	1.11E-21	1.11E-21	2.12E-21
		GO:0000166~nucleotide binding	3.75E-15	3.05E-12	1.52E-12	5.80E-12
		GO:0003723~RNA binding	1.32E-12	1.06E-09	3.54E-10	2.02E-09
		GO:0001882~nucleoside binding	4.26E-10	3.44E-07	8.60E-08	6.55E-07
		GO:0030554~adenyl nucleotide binding	4.95E-10	4.00E-07	7.99E-08	7.61E-07
		GO:0001883~purine nucleoside binding	5.11E-10	4.13E-07	6.88E-08	7.86E-07
		GO:0005524~ATP binding	1.60E-09	1.29E-06	1.85E-07	2.47E-06
		GO:0032559~adenyl ribonucleotide binding	1.93E-09	1.55E-06	1.94E-07	2.96E-06
		GO:0017076~purine nucleotide binding	2.16E-09	1.74E-06	1.94E-07	3.32E-06
		GO:0005198~structural molecule activity	3.17E-09	2.56E-06	2.56E-07	4.88E-06
		GO:0032555~purine ribonucleotide binding	8.55E-09	6.90E-06	6.27E-07	1.31E-05
		GO:0032553~ribonucleotide binding	8.55E-09	6.90E-06	6.27E-07	1.31E-05
		GO:0004674~protein serine/threonine kinase activ	1.97E-05	0.0157426	0.00132144	0.03021
		GO:0004386~helicase activity	4.94E-05	0.0390602	0.00306019	0.07584

Supplementary Table 3.

GO	Fold	Term	PValue	Bonferroni	Benjamini	FDR
Biological process	> 10 fold	GO:0009968~negative regulation of signal transduction	2.40E-06	0.0057305	0.00573	0.00422
		GO:0010648~negative regulation of cell communication	3.31E-06	0.0078865	0.003951	0.00581
	4-10 fold	GO:0007049~cell cycle	4.01E-06	0.0080937	0.008094	0.00691
		GO:0007017~microtubule-based process	4.87E-05	0.0940077	0.048164	0.08387
	2-4 fold	GO:0007049~cell cycle	1.01E-13	2.60E-10	2.60E-10	1.78E-10
		GO:0000279~M phase	8.72E-13	2.25E-09	1.13E-09	1.54E-09
		GO:0022403~cell cycle phase	9.02E-13	2.33E-09	7.77E-10	1.60E-09
		GO:0000087~M phase of mitotic cell cycle	1.56E-12	4.03E-09	1.01E-09	2.76E-09
		GO:0000278~mitotic cell cycle	1.67E-12	4.31E-09	8.61E-10	2.95E-09
		GO:0051301~cell division	2.33E-12	6.02E-09	1.00E-09	4.13E-09
		GO:0048285~organelle fission	2.67E-12	6.91E-09	9.87E-10	4.73E-09
		GO:0000280~nuclear division	3.23E-12	8.34E-09	1.04E-09	5.72E-09
		GO:0007067~mitosis	3.23E-12	8.34E-09	1.04E-09	5.72E-09
		GO:0022402~cell cycle process	8.16E-12	2.11E-08	2.34E-09	1.45E-08
		GO:0006259~DNA metabolic process	2.06E-11	5.33E-08	5.33E-09	3.65E-08
		GO:0051276~chromosome organization	1.64E-09	4.24E-06	3.85E-07	2.90E-06
		GO:0006350~transcription	2.34E-09	6.05E-06	5.04E-07	4.15E-06
		GO:0045449~regulation of transcription	1.27E-08	3.28E-05	2.52E-06	2.25E-05
		GO:0006974~response to DNA damage stimulus	1.70E-08	4.38E-05	3.13E-06	3.00E-05
		GO:0033554~cellular response to stress	2.84E-08	7.34E-05	4.89E-06	5.03E-05
		GO:0009057~macromolecule catabolic process	1.03E-07	2.65E-04	1.66E-05	1.82E-04
		GO:0006281~DNA repair	1.97E-07	5.09E-04	2.99E-05	3.49E-04
		GO:0044265~cellular macromolecule catabolic process	2.32E-07	5.98E-04	3.33E-05	4.10E-04
		GO:0030163~protein catabolic process	6.50E-07	0.00168	8.85E-05	0.00115
		GO:0051603~proteolysis involved in cellular protein catabo	6.89E-07	0.0017806	8.91E-05	0.00122
		GO:0044257~cellular protein catabolic process	8.39E-07	0.0021676	1.03E-04	0.00149
		GO:0019941~modification-dependent protein catabolic pro	2.51E-06	0.0064684	2.95E-04	0.00445
		GO:0043632~modification-dependent macromolecule cata	2.51E-06	0.0064684	2.95E-04	0.00445
		GO:0008104~protein localization	6.94E-06	0.0177755	7.79E-04	0.01229
		GO:0007059~chromosome segregation	8.75E-06	0.0223697	9.42E-04	0.0155
		GO:0006396~RNA processing	1.98E-05	0.0499944	0.002049	0.03513
		GO:0006325~chromatin organization	2.32E-05	0.058256	0.002306	0.04111
		GO:0046907~intracellular transport	2.82E-05	0.0702281	0.002693	0.04987
		GO:0016071~mRNA metabolic process	4.52E-05	0.1102963	0.004165	0.08003

GO	Fold	Term	PValue	Bonferroni	Benjamini	FDR
Celluar component	> 10 fold	GO:0005886~plasma membrane	7.21E-07	2.49E-04	2.49E-04	9.85E-04
		GO:0044459~plasma membrane part	1.11E-05	0.0038067	0.001905	0.01509
	4-10 fold	GO:0005874~microtubule	3.93E-06	0.0014939	0.001494	0.00545
		GO:0005856~cytoskeleton	2.65E-05	0.0100137	0.005019	0.03666
	2-4 fold	GO:0043232~intracellular non-membrane-bounded organe	3.00E-13	1.32E-10	1.32E-10	4.24E-10
		GO:0043228~non-membrane-bounded organelle	3.00E-13	1.32E-10	1.32E-10	4.24E-10
		GO:0031974~membrane-enclosed lumen	1.99E-12	8.78E-10	4.39E-10	2.81E-09
		GO:0070013~intracellular organelle lumen	8.09E-12	3.57E-09	1.19E-09	1.14E-08
		GO:0005694~chromosome	8.58E-12	3.78E-09	9.46E-10	1.21E-08
		GO:0031981~nuclear lumen	9.32E-12	4.11E-09	8.22E-10	1.32E-08
		GO:0043233~organelle lumen	9.69E-12	4.27E-09	7.12E-10	1.37E-08
		GO:0000793~condensed chromosome	8.17E-09	3.60E-06	5.14E-07	1.16E-05
		GO:0015630~microtubule cytoskeleton	8.39E-09	3.70E-06	4.62E-07	1.19E-05
		GO:0000775~chromosome, centromeric region	1.76E-08	7.76E-06	8.62E-07	2.49E-05
		GO:0044427~chromosomal part	2.60E-08	1.14E-05	1.14E-06	3.67E-05
		GO:0000779~condensed chromosome, centromeric region	1.20E-06	5.29E-04	4.81E-05	0.0017
		GO:0005654~nucleoplasm	3.26E-06	0.0014353	1.20E-04	0.00461
		GO:0005815~microtubule organizing center	4.67E-06	0.0020583	1.58E-04	0.00661
		GO:0005813~centrosome	5.77E-06	0.0025402	1.82E-04	0.00816
		GO:0044451~nucleoplasm part	8.00E-06	0.0035206	2.35E-04	0.01131
		GO:0000776~kinetochore	9.83E-06	0.0043271	2.71E-04	0.01391
		GO:0005783~endoplasmic reticulum	1.28E-05	0.0056302	3.32E-04	0.01811
		GO:0030529~ribonucleoprotein complex	1.62E-05	0.0071048	3.96E-04	0.02287
		GO:0000777~condensed chromosome kinetochore	4.11E-05	0.0179522	9.53E-04	0.05809
		GO:0005856~cytoskeleton	4.27E-05	0.0186598	9.41E-04	0.0604
		GO:0005794~Golgi apparatus	5.36E-05	0.0233625	0.001125	0.0758

GO	Fold	Term	PValue	Bonferroni	Benjamini	FDR
Molecular function	> 10 fold	GO:0046872~metal ion binding	1.72E-04	1.11E-01	1.11E-01	2.58E-01
	4-10 fold	GO:0008270~zinc ion binding	4.15E-06	0.0025742	0.002574	0.00616
		GO:0046914~transition metal ion binding	6.98E-06	0.0043268	0.002166	0.01036
		GO:0046872~metal ion binding	8.77E-06	0.0054289	0.001813	0.01301
		GO:0043169~cation binding	1.08E-05	0.006687	0.001676	0.01603
		GO:0043167~ion binding	1.69E-05	0.0104682	0.002102	0.02514
	2-4 fold	GO:0008270~zinc ion binding	5.37E-10	4.18E-07	4.18E-07	8.21E-07
		GO:0003677~DNA binding	2.24E-08	1.74E-05	8.72E-06	3.42E-05
		GO:0046914~transition metal ion binding	3.45E-07	2.68E-04	8.95E-05	5.27E-04
		GO:0032555~purine ribonucleotide binding	1.37E-06	0.0010636	2.66E-04	0.00209
		GO:0032553~ribonucleotide binding	1.37E-06	0.0010636	2.66E-04	0.00209
		GO:0005524~ATP binding	2.21E-06	0.0017215	3.45E-04	0.00338
		GO:0017076~purine nucleotide binding	2.38E-06	0.0018539	3.09E-04	0.00364
		GO:0032559~adenyl ribonucleotide binding	3.90E-06	0.0030297	4.33E-04	0.00596
		GO:0000166~nucleotide binding	4.70E-06	0.0036526	4.57E-04	0.00719
		GO:0030554~adenyl nucleotide binding	7.34E-06	0.0056994	6.35E-04	0.01122
		GO:0001882~nucleoside binding	9.76E-06	0.0075756	7.60E-04	0.01493
		GO:0001883~purine nucleoside binding	1.16E-05	0.0090059	8.22E-04	0.01776
